# Development and validation of an immune-related gene prognostic model for stomach adenocarcinoma

**DOI:** 10.1042/BSR20201012

**Published:** 2020-10-28

**Authors:** Ming Wu, Yu Xia, Yadong Wang, Fei Fan, Xian Li, Jukun Song, Jie Ding

**Affiliations:** 1 Medical College, Guizhou University, Guiyang, China; 2 Department of Medicine Emergency, Guizhou Provincial People’s Hospital, Guiyang, China; 3 Department of Stomatology, Guizhou Provincial People’s Hospital, Guiyang, China; 4 Department of Oral and Maxillofacial Surgery, Guizhou Provincial People’s Hospital, Guiyang, China; 5 Graduate School of Guizhou Medical University, Guiyang, China; 6 Department of Gastrointestinal Surgery, Guizhou Provincial People’s Hospital, Guiyang, China; 7 Graduate School of Zunyi Medical University, Zunyi, China

**Keywords:** bioinformatics, immune-related gene, prognosis, stomach adenocarcinoma, TCGA

## Abstract

Purpose**:** Stomach adenocarcinoma (STAD) is one of the most common malignant tumors, and its occurrence and prognosis are closely related to inflammation. The aim of the present study was to identify gene signatures and construct an immune-related gene (IRG) prognostic model in STAD using bioinformatics analysis.

Methods: RNA sequencing data from healthy samples and samples with STAD, IRGs, and transcription factors were analyzed. The hub IRGs were identified using univariate and multivariate Cox regression analyses. Using the hub IRGs, we constructed an IRG prognostic model. The relationships between IRG prognostic models and clinical data were tested.

Results: A total of 289 differentially expressed IRGs and 20 prognostic IRGs were screened with a threshold of *P*<0.05. Through multivariate stepwise Cox regression analysis, we developed a prognostic model based on seven IRGs. The prognostic model was validated using a GEO dataset (GSE 84437). The IRGs were significantly correlated with the clinical outcomes (age, histological grade, N, and M stage) of STAD patients. The infiltration abundances of dendritic cells and macrophages were higher in the high-risk group than in the low-risk group.

Conclusions: Our results provide novel insights into the pathogenesis of STAD. An IRG prognostic model based on seven IRGs exhibited the predictive value, and have potential application value in clinical decision-making and individualized treatment.

## Introduction

Stomach adenocarcinoma (STAD) is the fifth most common malignancy in the world. It is characterized by high mortality, high degree of malignancy, and strong heterogeneity [1]. In China, more than half of STAD patients when first seen clinically are diagnosed with stage III or IV disease [2], which excludes them as candidates for surgery. The prognosis of patients with metastatic STAD is poor with a median overall survival (OS) of 1 year [3]. For these patients, the treatment options are usually limited to chemo-/radiotherapy. Thus, immunotherapy has attracted considerable attention from clinicians and patients.

To date, an increasing number of studies have shown that genetic biomarkers can predict patient outcomes and guide their treatments. Previous studies have reported that tyrosine-protein kinase Met, epidermal growth factor receptor, and human epidermal growth factor receptor 2 could be potential targets for the targeted immunotherapy in STAD. However, targeted drugs have a limited clinical impact except for trastuzumab [4]. Given the heterogeneity of STAD, many studies have shown significant differences in the relationships between PD-L1 expression and outcomes [4]. Only some patients with metastatic or recurrent PD-L1-positive STAD who received intravenous pembrolizumab as an anti-PD-1 antibody drug were judged to have had a good response in a phase 1b trial [5]. Moreover, many current genetic biomarkers ignore clinicopathological features and cannot reflect the overall immune function of patients.

To provide new insights for improving clinical diagnosis and evaluation of patients’ immune status and outcomes, the differential expression of immune-related genes (IRGs) in STAD tissue samples should be studied. Thus, in the present study, we hypothesized that IRGs could assist in the assessment of prognosis in patients with STAD. We combined IRGs with clinical data of patients with STAD and constructed an IRG prognostic model that can accurately predict the outcomes of patients with STAD.

## Materials and methods

### Clinical samples and data acquisition

The gene expression profiles were downloaded from three publicly available datasets: TCGA STAD cohort (https://portal.gdc.cancer.gov/), Genotype-Tissue Expression (GTEx) project (https://commonfund.nih.gov/GTEx/), and Gene Expression Omnibus (GEO, https://www.ncbi.nlm.nih.gov/geo/). The tumor samples were obtained from TCGA STAD cohort, and the healthy samples were acquired from the GTEx project. We merged the mRNA data from TCGA STAD cohort and GTEx into one gene expression profile using batch normalization. TCGA STAD cohort included 32 normal tissues and 375 tumor tissues. A total of 145 healthy samples were obtained from the GTEx project. Another GEO dataset (GSE84437) was also downloaded for the external validation, which included 433 samples. Additionally, the clinical data of these patients, including survival time, TNM stage, gender, age, and histological grade, were also downloaded. The detailed clinical features are listed in Supplementary Tables S1 and 2.

### Differentially expressed analysis

First, we downloaded 2499 IRGs from the Immunology Database and Analysis Portal (ImmPort) [6] (Supplementary Table S3). The genes were confirmed to be involved in immune activities. Second, we conducted differentially expressed analysis on the IRGs using the Limma package with a false discovery rate (FDR) < 0.05 and a log2 |fold change| > 1.0.

### Survival analysis

OS was selected as the primary endpoint. To identify prognosis-related IRGs, univariate COX regression analysis and Kaplan–Meier (KM) survival analysis were performed using the survival package of R software. The prognosis-related IRGs were identified with threshold of *P*<0.05 and |hazard ratio| > 1.0. The results were exhibited using a forest plot. We also explored the association between IRGs and other clinical information, such as age, TNM stage, and histological grade.

### Construction of prognostic prediction model using the hub IRGs

The prognosis-related hub IRGs were further screened using a multivariate COX stepwise regression analysis. Additionally, an IRG prognostic model was constructed using the hub IRGs. The formula of the prognostic model was as follows: the coefficient was multiplied by the gene expression for each patient. According to the median prognostic index value, the patients were divided into high- and low-risk groups. Then, the KM survival curve, receiver operating characteristic (ROC) curve, and risk curve were analyzed according to the established models. Univariate and multivariate COX prognostic analyses were used to determine whether the established IRG prognostic model was prognostic factor for STAD.

### Validation of the prognostic prediction model

The established prognostic prediction model was validated using a GEO dataset (GSE84437), and the KM survival curve, ROC curve, and risk curve were plotted. We also evaluated whether the established IRG prognostic model was prognostic factor for STAD in another dataset.

### Exploration of the indicators associated with IRGs and clinical prognosis

Molecular characterization of tumor–immune interactions was conducted using the Tumor Immune Estimation Resource (TIMER; cistrome.shinyapps.io/timer) [7]. As a public resource, TIMER uses RNA sequencing (RNA-seq) expression profile data to detect the infiltration of immune cells in tumor tissues and estimates the proportion of six subtypes of TIICs (B cells, CD4 T cells, CD8 T cells, neutrophils, macrophages, and dendritic cells). We downloaded immune infiltration levels of STAD patients and calculated the associations between the IRG-based prognostic index and immune cell infiltration.

### Statistical analysis

The Wilcoxon test was utilized to test the statistical significance between the two groups. For comparisons of more than two groups, Kruskal–Wallis tests were used to examine the difference among groups [8]. Cox regression analysis was applied to determine the association between IRGs and OS. The log-rank statistic was used to test the differences in OS survival between groups using KM plots. In the univariate Cox regression analysis, variables with *P*<0.05 were considered as prognostic factors, and the included prognostic factors were used to construct the risk model for OS. Statistical analysis was performed using R v3.4.1 (https://www.r-project.org/) and packages obtained through the Bioconductor project (www.bioconductor.org). Statistical significance was considered at *P*<0.05.

## Results

### Identification of prognosis-related differentially expressed IRGs

**Figure 1:**
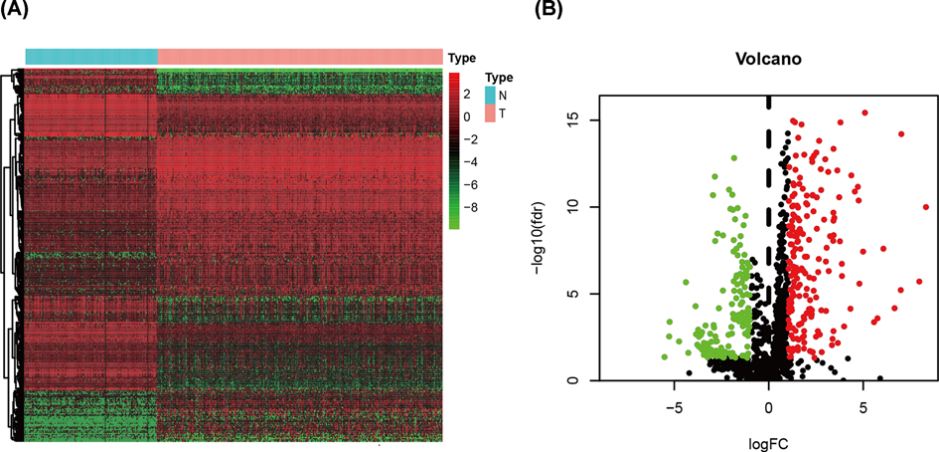
Differentially expressed immune-related genes (**A**) and volcano plot (**B**) demonstrate differentially expressed immune-related genes that are shown. Red dots represent up-regulated differentially expressed genes, green dots represent down-regulated differentially expressed genes, and black dots represent no differentially expressed genes.

Data from 174 healthy tissues and 375 STAD tumor tissues were considered eligible in the analysis, within which 289 differentially expressed IRGs were identified, including 126 up-regulated and 163 down-regulated genes with the cutoff point of *P*<0.05 and log2 |fold change| > 1.0 (Figure 1).

### Identification of prognosis-related IRGs

**Figure 2:**
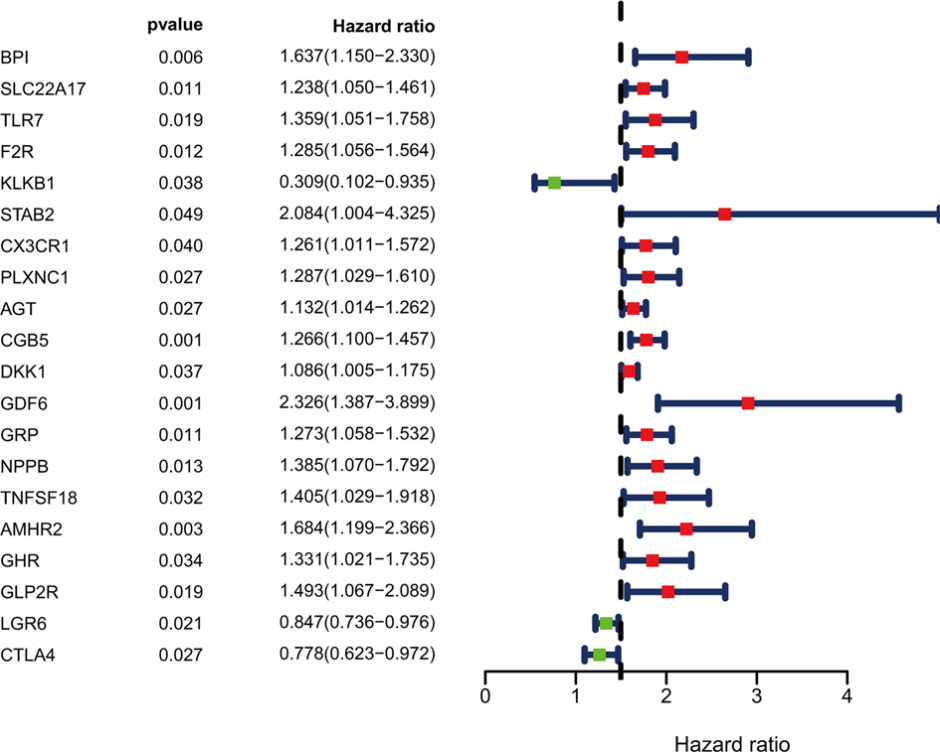
Expression profiles and prognostic values of prognosis-related immune-related genes Forest plot of hazard ratios showing the prognostic values of genes.

**Figure 3:**
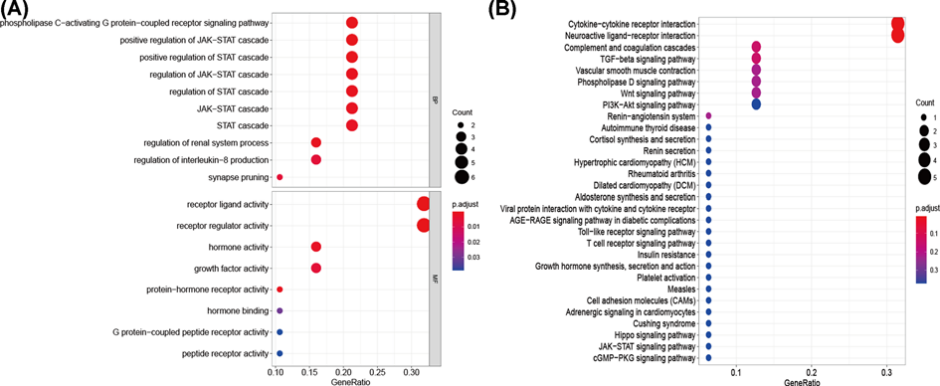
Functional enrichment of prognosis-related immune-related genes (**A**) Gene ontology analysis. (**B**) Kyoto Encyclopedia of Genes and Genomes pathways analysis.

Twenty prognosis-related differentially expressed IRGs were identified as potential prognostic molecular biomarkers. The forest plot is shown in Figure 2. We found that these prognosis-related IRGs were mostly enriched in several gene ontology terms associated with the regulation of the JAK–STAT cascade, receptor regulator activity, and receptor ligand activity. The cytokine–cytokine receptor interaction was one of the most frequently identified KEGG pathways (Figure 3).

### Establishment of IRG-related prognostic prediction model

**Figure 4:**
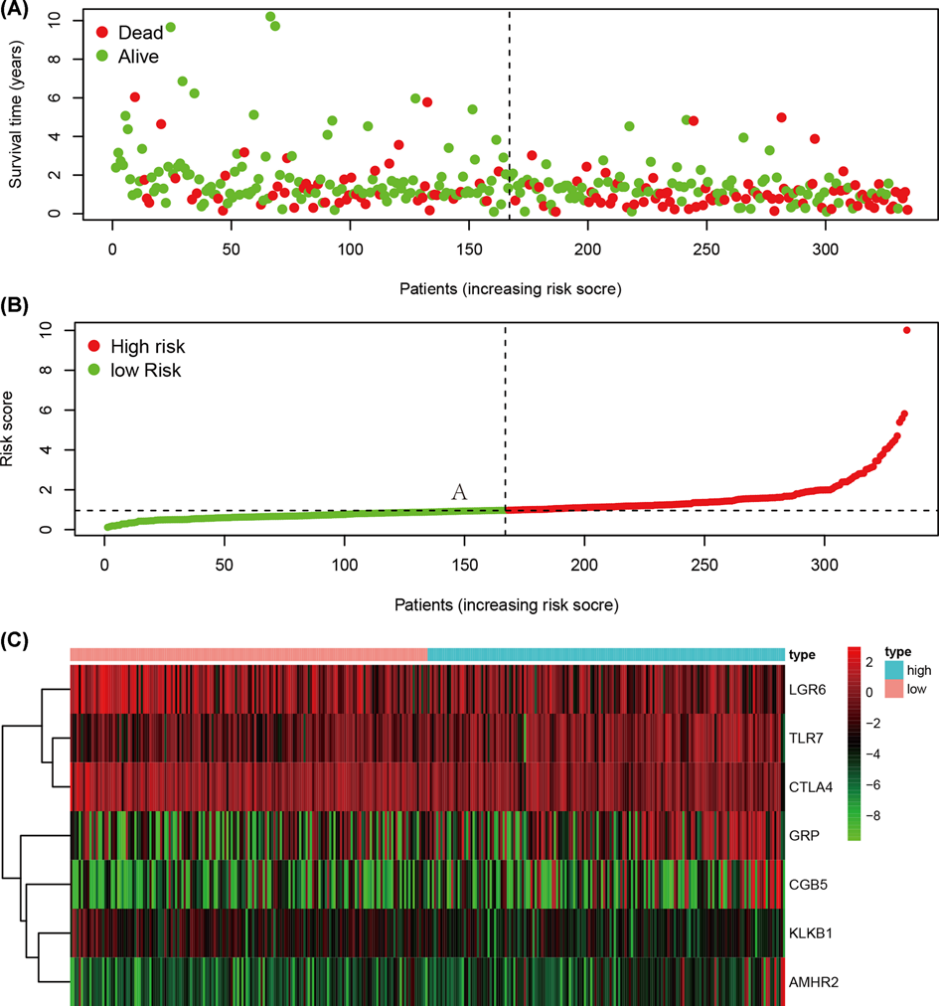
Development of the prognostic index based on immune-related genes (**A**) Survival status of patients in different groups. (**B**) Rank of prognostic index and distribution of groups. (**C**) Heatmap of expression profiles of included genes.

**Figure 5:**
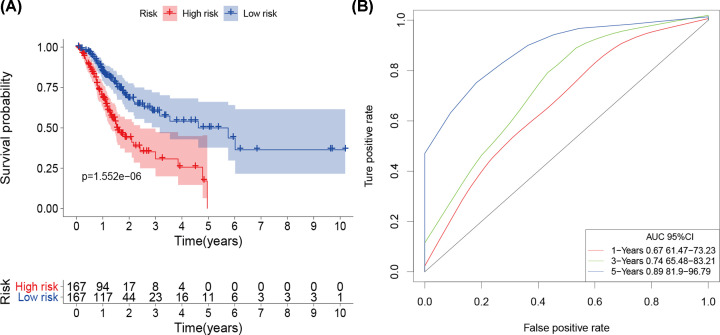
The prognostic value of prognostic index (**A**) Patients in the high-risk group suffered a lower survival probability. (**B**) Survival-dependent receiver operating characteristic curve validation of prognostic value of the prognostic index.

**Figure 6:**
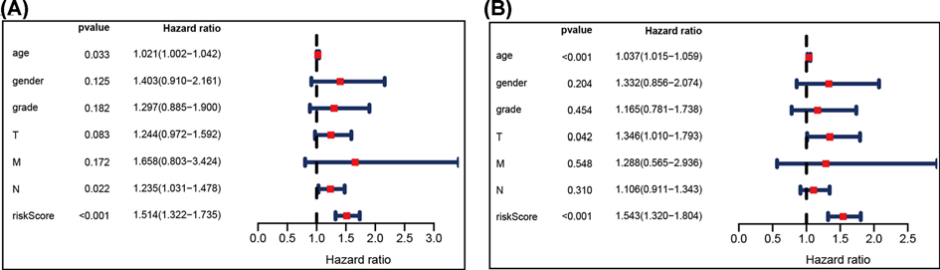
Univariate and multivariate Cox regression analysis of hub immune-related genes and clinical indicators (**A**) univariate Cox regression analysis and (**B**) multivariate Cox regression analysis.

We used the multivariate stepwise Cox analysis to construct a prediction model and identify the optimal mRNA set and used the selected mRNA to construct an mRNA-based feature classifier to predict survival status. Seven prognostic IRGs were selected to establish the prognostic prediction model (Table 1). With the seven hub IRGs, the risk score model was constructed as follows: [Expression level of TLR7 * 0.444] + [Expression level of KLKB1 * (-1.267)] + [Expression level of CGB5 * 0.151] + [Expression level of GRP * 0.197] + [Expression level of AMHR2 * 0.572] + [Expression level of LGR6 * (-0.169)] + [Expression level of CTLA4 * (-0.1370)]. Based on the results of the IRG prognostic model, the patients with STAD were divided into the high-risk and low-risk groups (Figure 4). Patients in the high-risk group had a lower survival probability (Figure 5A)**.** The area under the curve of the ROC curve for 1, 3, and 5 years reached 0.673, 0.743, and 0.893, respectively. The result indicated that the model had a medium effect in monitoring survival (Figure 5B). Univariate and multivariate Cox regression analyses suggested that the IRG prognostic model could become an independent predictor, including age, gender, histological grade, TNM stage, and risk score (Figure 6).

**Table 1:** Multivariate COX STEP stepwise regression analysis of hub immune-related genes

ID	Coef	HR	HR.95L	HR.95H	*P* value
TLR7	0.444	1.560	1.180	2.060	0.002
KLKB1	-1.267	0.282	0.090	0.883	0.030
CGB5	0.151	1.163	0.993	1.362	0.062
GRP	0.197	1.218	0.996	1.489	0.054
AMHR2	0.572	1.772	1.261	2.490	0.001
LGR6	-0.169	0.845	0.727	0.981	0.027
CTLA4	-0.370	0.691	0.532	0.896	0.005

### Validation of the constructed IRG prognostic model

**Figure 7:**
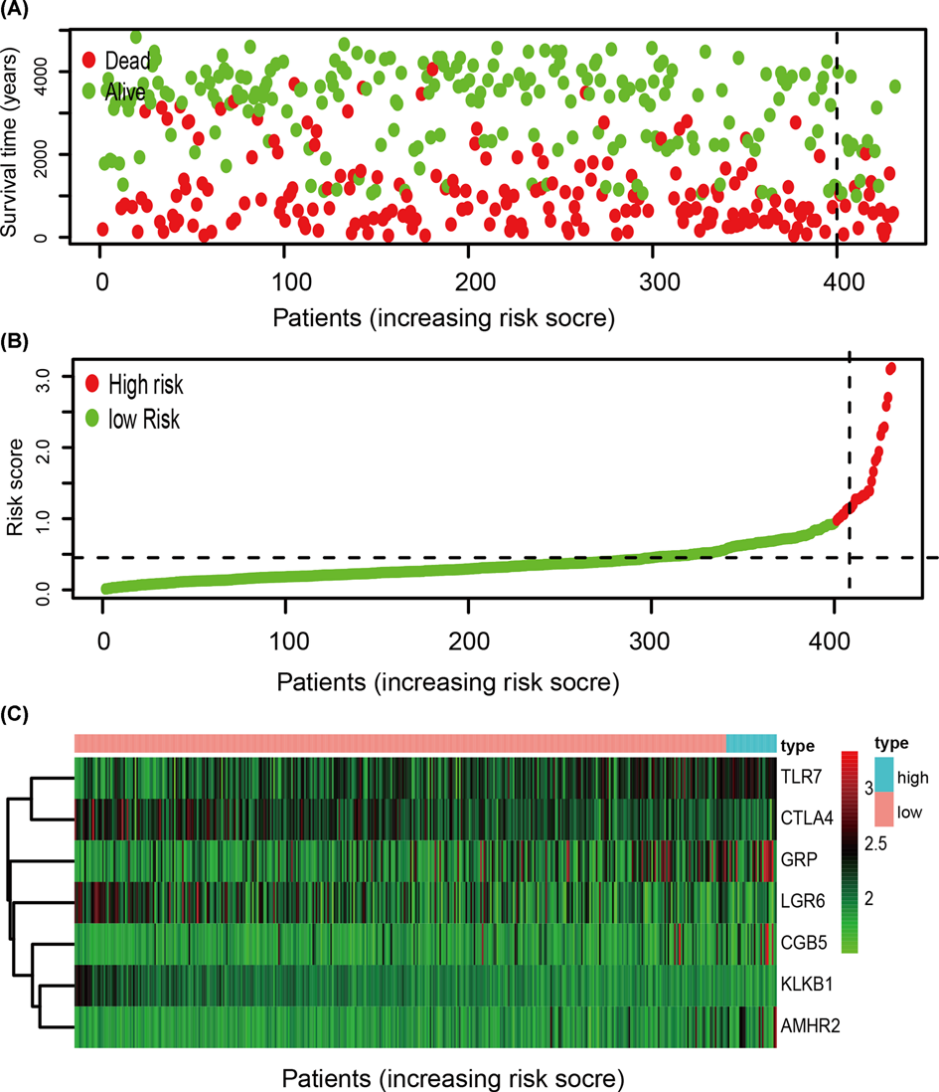
Validation of the prognostic index based on immune-related genes using a GEO dataset (GSE84437) (**A**) Survival status of patients in different groups. (**B**) Rank of prognostic index and distribution of groups. (**C**) Heatmap of expression profiles of included genes.

**Figure 8:**
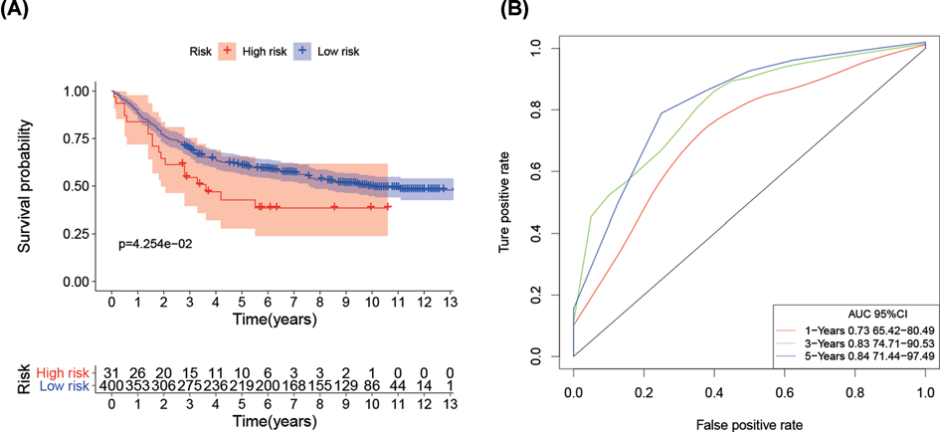
The prognostic risk index based on GEO dataset (GSE84437) (**A**) Patients in the high-risk group suffered a lower survival probability. (**B**) Survival-dependent receiver operating characteristic curve shown prognostic value of the prognostic index.

**Figure 9:**
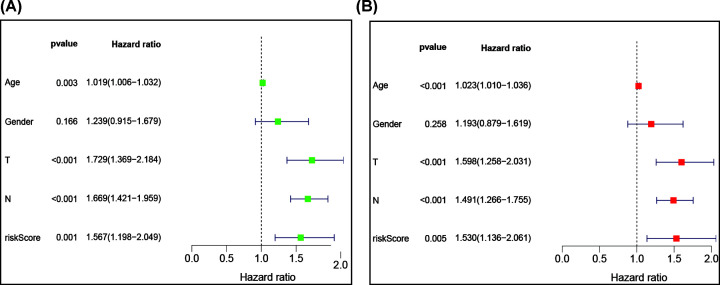
Univariate and multivariate Cox regression analysis of hub immune-related genes and clinical indicators based on GEO dataset (GSE84437) (**A**) Univariate Cox regression analysis and (**B**) multivariate Cox regression analysis.

The IRG prognostic model based on the seven IRGs was validated using a GEO dataset (GSE84437), and the result indicated that the model exhibited good predictive ability (Figure 7). Patients in the high-risk group had a lower survival probability (Figure 8A). The area under the curve of the ROC curve for 1, 3, and 5 years reached 0.729, 0.826, and 0.845, respectively (Figure 8B). Univariate and multivariate Cox regression analyses suggested that the IRG prognostic model could become an independent predictor, including age, gender, histological grade, TNM stage, and risk score (Figure 9).

### Predictive models play an important role in clinical indicators

**Figure 10:**
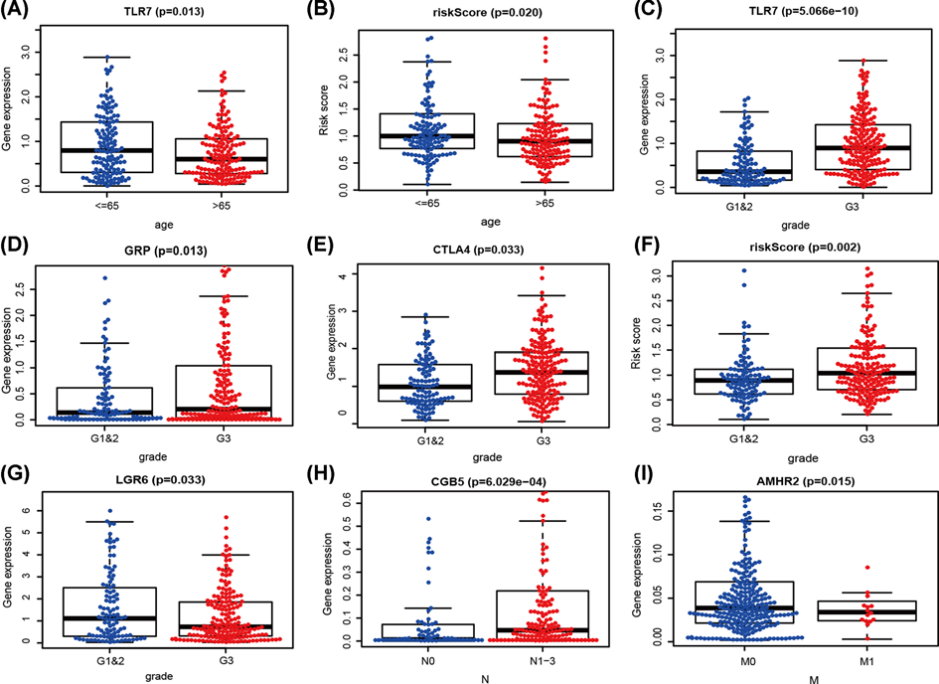
The relationships between the hub immune-related genes and clinical indicators (**A,B**) age, (**C–G**) pathological stage, (**H**) N stage, and (**I**) M stage.

**Figure 11:**
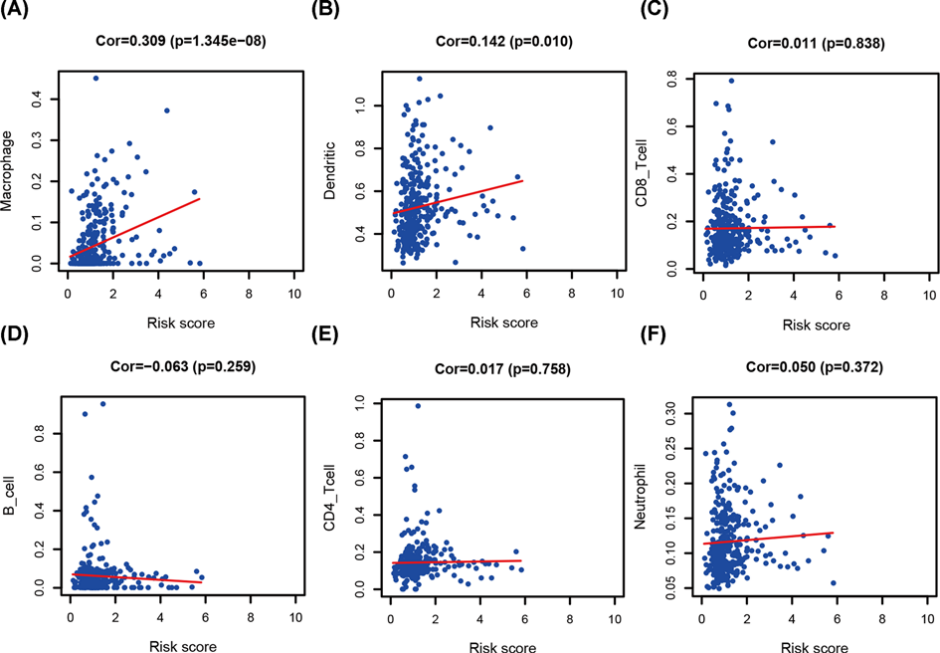
Relationships between the immune-related gene prognostic model and infiltration abundances of six types of immune cells The correlation was performed using Pearson correlation analysis. (**A**) macrophages, (**B**) dendritic cells, (**C**) CD8 T cells, (**D**) B cells, (**E**) CD4 T cells, and (**F**) neutrophils.

To predict clinical outcomes more accurately, the relationships were analyzed between seven hub IRGs and IRG prognostic model (Table 2). In the STAD population, TLR7 was significantly higher in patients with age less than or equal to 65 years (Figure 10A). For the high-risk group, the relationship was the same (Figure 10B). TLR7, GRP, and CTLA4 were significantly higher in G3 than in G1 and 2 (Figure 10C–E). For the high-risk group, the relationship was the same (Figure 10F). However, LGR6 was lower in G3 than in G1 and 2 (Figure 10G). CGB5 was significantly expressed in N1-3 (Figure 10H). AMHR2 was significantly higher in M0 than in M1 (Figure 10I). To determine whether models effectively reflected the status of the tumor–immune microenvironment, the relationships between the IRG prognostic model and infiltration abundances of six types of immune cells were analyzed. The high-risk group was significantly positively correlated with dendritic cell and macrophages (Figure 11).

**Table 2:** Relationships between the expressions of the immune-related genes and the clinicopathological factors in stomach adenocarcinoma

Gene	Age (>65/ ≤65)		Gender (male/female)		Grade (G3/G1-2)		T stage (T3–T4/ T1–T2)		N stage (N1–3/ N0)		M stage (M1/ M0)	
	*t*	*P*	*t*	*P*	*t*	*P*	*t*	*P*	*t*	*P*	*t*	*P*
TLR7	2.503	**0.013**	1.483	0.140	-6.458	**5.066 e-10**	-0.225	0.171	-1.705	0.090	-0.997	0.334
KLKB1	0.175	0.861	1.413	0.160	0.007	0.994	0.610	0.543	0.034	0.973	0.360	0.722
CGB5	0.519	0.604	-0.552	0.582	-1.671	0.096	0.090	0.928	-3.472	**6.029e-04**	-1.565	0.138
GRP	0.239	0.811	0.659	0.511	-2.497	**0.013**	-1.151	0.252	-0.564	0.573	-0.290	0.775
AMHR2	1.291	0.198	-0.098	0.922	-0.137	0.891	1.202	0.233	1.138	0.258	2.466	**0.015**
LGR6	-1.040	0.299	-0.482	0.630	2.154	**0.033**	-1.012	0.313	1.982	0.050	0.557	0.585
CTLA4	-0.471	0.638	0.028	0.978	-2.148	**0.033**	-0.661	0.510	0.217	0.828	1.076	0.296
Risk Score	2.347	**0.020**	0.331	0.741	-3.071	**0.002**	1.022	0.309	-1.157	0.249	-1.526	0.147

Note: *t*: *t* value of Student’s *t*-test; *P*: *P*‐value of Student’s *t-*test.

## Discussion

In the present study, we focused on immunogenomic profiles and their corresponding clinical significance and found that some IRGs are closely related to the progression of STAD through bioinformatic analysis. These IRGs may be used as valuable clinical biomarkers and provide a basis for further exploration of their biological functions. Most importantly, an IRG prognostic model based on seven hub IRGs in STAD patients was constructed. The model could measure immune cell infiltration and could be used to assess potential clinical outcomes.

To explore a simple and effective method for monitoring the immune status and predicting the outcomes in STAD patients, we constructed an IRG prognostic model. Previously, Yang et al. [9] collected RNA-seq data of IRGs of 372 STAD patients from TCGA database, which integrated the clinicopathological data. They established a prognostic model and found 10 prognostic genes. Wang et al. [10] also collected protein-coding genes and clinical data of STAD patients from TCGA database and established a stromal–immune score-based gene signature and risk stratification model. Wu et al. [11] used the CIBERSORT algorithm and integrated clinical data to calculate the differences in the composition of immune-infiltrating cells in STAD tissues and predict the prognosis. Compared with previous studies, our study combined healthy population and TIICs to identify seven hub IRGs and establish the IRG prognostic model, which could reflect the patient’s immune status in order to guide and adjust treatment regimens more quickly and was closely related to age, pathological grade, and metastasis of STAD patients. Therefore, the IRG prognostic model could be used not only as an immune status indicator but also as a prognostic indicator.

Our analysis revealed the relationship between the IRG prognostic model and immune cell infiltration to reflect the infiltration status of the immune microenvironment of STAD patients. The infiltration levels of dendritic cells and macrophages were positively correlated with the IRG prognostic model. These results indicated that higher infiltration levels of dendritic cells and macrophages might be observed in the high-risk group of STAD patients. In STAD, tumor-related macrophages are one of the most abundant immune components. Macrophages can promote tumor angiogenesis and metastasis [12–14] and are positively correlated with poor prognosis [15]. Additionally macrophages are also the major sources of chemokines and chemokine receptors in the gastric cancer microenvironment, such as CXCL1 and CXCL5, and promote migration through activating the CXCR2/STAT3 feed-forward circuit [16]. Conversely, dendritic cells can predict poor prognosis of STAD [17,18]. The above results are consistent with our analysis. Our preliminary observation suggested that the hub IRGs and IRG prognostic model could be used as predictors of increased immune cell infiltration and prognostic indicators of immune status.

Our findings may provide novel insights and an exploratory perspective for further studies that may lead to new immunotherapies in STAD. However, this study has some limitations because the findings have not yet been confirmed *in vitro, in vivo*, or in a prospective clinical cohort.

## Supplementary Material

Supplementary Tables S1-S3

## Data Availability

The data used to perform the analyses described herein are publically available from official TCGA, GEO, GTEx, and ImmPort data portals.

## Abbreviations

IRG: immune-related gene
OS: overall survival
ROC: receiver operating characteristic
STAD: stomach adenocarcinoma
