# Supplementary material for: Development and validation of an immune-related gene prognostic model for stomach adenocarcinoma
Source: Biosci Rep. 2020 Oct 28;40(10):BSR20201012. doi: 10.1042/BSR20201012 (PMC7593539; doi:10.1042/BSR20201012)
Supplement: Supplementary Tables S1-S3 [file BSR-2020-1012_supp.pdf]

Supplementary Table 1| The detailed clinical feature of each sample obtained from the TCGA STAD cohort.

ID	Age (Y)	Gender	Grade	Stage	T	M	N
TCGA-CD-5800	51	0	1	Stage II	T3	M0	N0
TCGA-CG-5719	54	0	1	Stage IV	T4	M1	N0
TCGA-BR-4201	66	0	1	unknow	T2a	M0	NX
TCGA-BR-4371	71	0	1	unknow	T2	M0	NX
TCGA-BR-4292	73	0	1	unknow	T1	M0	NX
TCGA-HU-8244	77	0	1	Stage IA	T1a	M0	N0
TCGA-KB-A93H	79	0	1	Stage IIB	T3	M0	N1
TCGA-CD-A4MI	62	1	1	Stage IIIA	T3	M0	N1
TCGA-VQ-A91A	67	1	1	Stage IIIB	T3	M0	N3a
TCGA-KB-A93G	68	1	1	Stage IB	T2	M0	N0
TCGA-B7-A5TJ	74	1	1	Stage IIB	T4a	M0	NX
TCGA-VQ-A927	81	1	1	Stage IIIB	T3	M0	N3
TCGA-VQ-A8PY	47	0	2	Stage IIIB	T3	M0	N2
TCGA-VQ-A8PQ	50	0	2	Stage IV	T4	M1	N1
TCGA-VQ-AA68	52	0	2	Stage IIIC	T4a	M0	N3
TCGA-VQ-AA6D	52	0	2	Stage IIIA	T4a	M0	N1
TCGA-D7-5577	53	0	2	Stage IIIA	T2	M0	N3
TCGA-BR-8690	54	0	2	Stage IIIB	T3	M0	N3a
TCGA-BR-8077	58	0	2	Stage IIIB	T4b	M0	N1

TCGA-HU-A4H2	58	0	2	Stage IIIB	T3	M0	N3b
TCGA-VQ-A91N	59	0	2	Stage IV	T4	M0	N3
TCGA-D7-A74A	61	0	2	Stage IIIA	T3	M0	N2
TCGA-HU-A4GP	62	0	2	Stage IIA	T2	M0	N1
TCGA-BR-7716	62	0	2	Stage IIB	T3	M0	N1
TCGA-BR-8679	63	0	2	Stage IB	T2	M0	N0
TCGA-BR-8373	65	0	2	Stage IIIA	T4a	M0	N1
TCGA-VQ-A8PB	65	0	2	Stage II	T3	M0	N0
TCGA-R5-A7ZF	65	0	2	Stage IV	T4a	M1	N1
TCGA-CG-4460	66	0	2	Stage IV	T4	M1	N1
TCGA-D7-8579	66	0	2	Stage IIB	T2	M0	N2
TCGA-R5-A7ZE	66	0	2	Stage IIIA	T3	M0	N2
TCGA-VQ-A91Z	67	0	2	Stage IIIA	T3	M0	N1
TCGA-HU-A4G9	67	0	2	Stage IA	T1a	M0	N0
TCGA-D7-6526	67	0	2	Stage IIIA	T3	M0	N2
TCGA-BR-A4PE	68	0	2	Stage IB	T2	M0	N0
TCGA-VQ-A8DL	69	0	2	Stage IIA	T3	M0	N0
TCGA-BR-A4J6	69	0	2	Stage IIA	T3	M0	N0
TCGA-R5-A7ZR	70	0	2	Stage III	T3	M0	NX
TCGA-D7-6528	70	0	2	Stage IB	T2	M0	N0
TCGA-D7-6815	70	0	2	Stage IIB	T2	M0	N2

TCGA-BR-8081	71	0	2	Stage IIB	T4a	M0	N0
TCGA-IN-A6RN	72	0	2	Stage IIA	T1b	M0	N2
TCGA-VQ-A8P8	72	0	2	Stage IIB	T4a	M0	N0
TCGA-CD-8527	72	0	2	Stage II	T2	M0	N1
TCGA-HU-A4H6	72	0	2	Stage IIIA	T3	M0	N2
TCGA-BR-4370	74	0	2	unknow	TX	M0	NX
TCGA-BR-6456	74	0	2	Stage IIB	T3	M0	N1
TCGA-BR-A4QL	75	0	2	Stage IIIB	T3	M0	N3a
TCGA-CG-5718	78	0	2	Stage II	T2b	M0	N1
TCGA-BR-4280	78	0	2	Stage III	T2b	M0	N1
TCGA-BR-4257	79	0	2	unknow	TX	M0	NX
TCGA-CG-5730	80	0	2	Stage IV	T2	M1	N1
TCGA-CG-4466	81	0	2	Stage IB	T2a	M0	N0
TCGA-HU-8604	82	0	2	Stage IIA	T3	M0	N0
TCGA-CD-A4MH	86	0	2	Stage IIA	T3	M0	N0
TCGA-EQ-5647	86	0	2	Stage IV	T4	M1	N3
TCGA-BR-8486	90	0	2	Stage IA	T1b	M0	N0
TCGA-VQ-A91W	30	1	2	Stage IIIA	T3	M0	N1
TCGA-VQ-AA69	35	1	2	Stage IIIA	T3	M0	N2
TCGA-BR-6710	41	1	2	Stage IB	T2	M0	N0
TCGA-BR-8680	45	1	2	Stage IV	T4b	M1	N2

TCGA-IN-A6RI	45	1	2	Stage I	T1b	M0	N0
TCGA-SW-A7EB	45	1	2	Stage IIIA	T3	M0	N2
TCGA-VQ-AA6B	48	1	2	Stage IIIB	T3	M0	N2
TCGA-VQ-A8DV	48	1	2	Stage IB	T2	M0	N0
TCGA-VQ-A928	49	1	2	Stage IV	T3	M0	N3
TCGA-BR-4267	51	1	2	Stage IB	T2a	M0	N0
TCGA-R5-A7O7	51	1	2	Stage IV	T3	M1	N1
TCGA-VQ-A8PX	51	1	2	Stage IA	T1	M0	N0
TCGA-CD-8532	52	1	2	Stage II	T3	M0	N0
TCGA-HU-A4G3	54	1	2	Stage IIB	T2	M0	N2
TCGA-D7-A4YT	56	1	2	Stage IIIA	T2	M0	N3a
TCGA-VQ-AA6A	56	1	2	Stage IIIC	T4	M0	N3a
TCGA-HU-8238	56	1	2	unknow	T3	M0	unknow
TCGA-CG-4436	57	1	2	Stage IB	T2	M0	N0
TCGA-D7-8572	57	1	2	Stage IIB	T2	M0	N2
TCGA-CD-A48A	57	1	2	Stage IIA	T3	M0	N0
TCGA-VQ-AA6F	57	1	2	Stage IIB	T3	M0	N1
TCGA-VQ-A8E2	57	1	2	Stage IIIB	T3	M0	N2
TCGA-VQ-A91V	58	1	2	Stage IIIA	T3	M0	N2
TCGA-VQ-A8PK	58	1	2	Stage IIIB	T3	M0	N3a
TCGA-CD-5802	58	1	2	Stage II	T3	M0	N0

TCGA-BR-4357	58	1	2	unknow	T3	MX	NX
TCGA-BR-8483	59	1	2	Stage IIIA	T3	M0	N2
TCGA-VQ-A8E7	59	1	2	Stage IV	T3	M1	N1
TCGA-CD-A4MJ	60	1	2	Stage IB	T2	M0	N0
TCGA-B7-A5TN	60	1	2	Stage IIB	T4a	M0	NX
TCGA-MX-A666	61	1	2	Stage IIA	T2	MX	NX
TCGA-BR-8484	61	1	2	Stage IIIA	T4a	M0	N1
TCGA-HU-A4GN	61	1	2	Stage IIA	T2	M0	N1
TCGA-VQ-A8PH	62	1	2	Stage IIIB	T3	M0	N3
TCGA-D7-6527	62	1	2	Stage II	T2	M0	N1
TCGA-FP-8211	62	1	2	Stage IIB	T3	MX	N1
TCGA-VQ-A8DU	63	1	2	Stage IIIA	T3	M0	N2
TCGA-BR-7717	63	1	2	Stage IV	T4	M0	N1
TCGA-VQ-A94R	63	1	2	unknow	T4a	M0	N2
TCGA-IN-AB1V	63	1	2	Stage IA	T1b	M0	N0
TCGA-D7-6820	64	1	2	Stage IIB	T2	M0	N1
TCGA-CD-8529	65	1	2	Stage IV	T4	M1	N0
TCGA-BR-7715	65	1	2	Stage IIA	T3	M0	N0
TCGA-BR-4294	65	1	2	unknow	T2a	M0	NX
TCGA-VQ-A925	66	1	2	Stage IIIA	T3	M0	N2
TCGA-CG-5732	66	1	2	Stage IV	T2	M0	N3

TCGA-HU-8243	66	1	2	Stage IIIC	T4a	M0	N3b
TCGA-BR-8360	66	1	2	Stage IIA	T3	M0	N0
TCGA-VQ-A92D	67	1	2	Stage IB	T2b	M0	N0
TCGA-BR-6565	67	1	2	Stage IIB	T4a	M0	N0
TCGA-VQ-AA64	68	1	2	Stage IIIB	T3	M0	N2
TCGA-CG-4443	68	1	2	Stage IA	T1	M0	N0
TCGA-CD-A486	68	1	2	Stage IIA	T3	M0	N0
TCGA-VQ-AA6G	68	1	2	Stage IIA	T3	M0	N0
TCGA-FP-8631	68	1	2	Stage IIIA	T3	MX	N2
TCGA-F1-A72C	68	1	2	Stage IIA	T3	M0	N0
TCGA-VQ-A8P2	68	1	2	Stage IIIA	T4a	M0	N1
TCGA-HU-A4GF	69	1	2	Stage IIA	T3	M0	N0
TCGA-FP-7829	69	1	2	Stage IIB	T3	M0	N1
TCGA-VQ-A924	69	1	2	Stage II	T3	M0	N0
TCGA-VQ-A8PD	69	1	2	Stage IIIC	T4a	M0	N3
TCGA-VQ-A91K	69	1	2	Stage IIIA	T3	M0	N1
TCGA-VQ-A91D	70	1	2	Stage IIIC	T4b	M0	N2
TCGA-BR-4184	70	1	2	Stage IIIA	T3	MX	N1
TCGA-BR-6801	70	1	2	Stage IIA	T3	M0	N0
TCGA-VQ-A922	70	1	2	Stage IV	T4	M0	N1
TCGA-3M-AB46	70	1	2	Stage IB	T2b	MX	N0

TCGA-IN-A6RO	70	1	2	Stage IA	T1b	M0	N0
TCGA-EQ-A4SO	70	1	2	Stage IIIB	T4b	MX	N1
TCGA-HU-A4GQ	71	1	2	Stage IIIC	T4b	M0	N2
TCGA-ZA-A8F6	71	1	2	Stage IB	T2	MX	N0
TCGA-D7-8578	72	1	2	Stage IB	T2	M0	N0
TCGA-BR-4191	72	1	2	unknow	TX	M0	N1
TCGA-VQ-A8P3	72	1	2	Stage IIIA	T4	M0	N1
TCGA-CG-5726	73	1	2	Stage IA	T1	M0	N0
TCGA-HU-A4HD	73	1	2	Stage IIIA	T3	M0	N2
TCGA-VQ-A94O	74	1	2	Stage IIIC	T4a	M0	N3
TCGA-IP-7968	74	1	2	Stage IIIB	T3	MX	N2
TCGA-BR-7901	74	1	2	Stage IIB	T3	M0	N1
TCGA-HU-A4GH	75	1	2	Stage IA	T1b	M0	N0
TCGA-HU-8610	75	1	2	Stage IA	T1b	M0	N0
TCGA-VQ-A8PS	76	1	2	Stage IIIA	T3	M0	N1
TCGA-VQ-A8PP	76	1	2	Stage IV	T4	M0	N1
TCGA-HU-8249	76	1	2	Stage IIIA	T3	M0	N2
TCGA-IN-A6RS	76	1	2	Stage IA	T1b	M0	N0
TCGA-BR-8678	76	1	2	Stage IB	T2	M0	N0
TCGA-D7-A6ET	76	1	2	Stage IIIB	T3	M0	N3a
TCGA-FP-A9TM	77	1	2	unknow	T1b	M0	N1

TCGA-HU-A4H8	77	1	2	Stage IB	T1b	M0	N1
TCGA-FP-7735	77	1	2	Stage IB	T2	MX	N0
TCGA-D7-6822	77	1	2	Stage IB	T2	M0	N0
TCGA-RD-A8N5	78	1	2	Stage IIIA	T3	M0	N1
TCGA-VQ-A91U	78	1	2	Stage IIIA	T3	M0	N1
TCGA-CG-4300	79	1	2	Stage IIIB	T3	M0	N2
TCGA-FP-8099	79	1	2	Stage IIA	T3	MX	N0
TCGA-F1-6875	79	1	2	Stage IA	T2	M0	N0
TCGA-BR-4256	80	1	2	unknow	TX	M0	N1
TCGA-IN-8462	80	1	2	Stage IIB	T2	M0	N1
TCGA-CG-4449	81	1	2	Stage II	T2	M0	N1
TCGA-BR-7703	81	1	2	Stage IA	T1b	M0	N0
TCGA-CD-5798	82	1	2	Stage II	T3	M0	N0
TCGA-CG-4441	83	1	2	Stage IIIA	T2	M0	N2
TCGA-CG-4437	83	1	2	Stage II	T2	M0	N1
TCGA-CG-4304	84	1	2	Stage IB	T2a	M0	N0
TCGA-IN-A6RL	84	1	2	unknow	T2	M0	N1
TCGA-CG-5716	86	1	2	Stage IV	T4a	M1	N2
TCGA-BR-4366	87	1	2	unknow	TX	M0	NX
TCGA-F1-6177	90	1	2	Stage I	T1b	MX	N1
TCGA-CD-5804	90	1	2	unknow	T3	M0	N1

TCGA-BR-A4IU	34	0	3	Stage IIIA	T4a	M0	N1
TCGA-D7-A748	41	0	3	Stage IV	T4a	M1	N3
TCGA-BR-8364	42	0	3	Stage IIIC	T4b	M0	N2
TCGA-CD-8528	43	0	3	Stage IIIA	T4	M0	N0
TCGA-R5-A7ZI	44	0	3	Stage IV	T4	M1	N1
TCGA-BR-A4IZ	45	0	3	Stage IIIB	T4a	M0	N2
TCGA-BR-6564	46	0	3	Stage IIIA	T3	M0	N2
TCGA-B7-5816	51	0	3	Stage IIB	T4a	M0	N0
TCGA-D7-A74B	52	0	3	Stage IIIB	T3	M0	N3
TCGA-BR-8058	53	0	3	Stage IIIB	T4a	M0	N2
TCGA-HU-A4H4	53	0	3	Stage IIB	T2	M0	N2
TCGA-BR-4188	53	0	3	unknow	TX	M0	N1
TCGA-RD-A8N0	53	0	3	Stage IIIB	T3	M0	N2
TCGA-BR-A44T	53	0	3	Stage IIA	T3	M0	N0
TCGA-KB-A6F7	54	0	3	Stage IB	T1	M0	N1
TCGA-D7-8576	54	0	3	Stage IIIB	T3	M0	N3a
TCGA-BR-6803	54	0	3	Stage IIA	T3	M0	N0
TCGA-BR-8588	55	0	3	Stage IIB	T4a	M0	N0
TCGA-BR-4183	55	0	3	Stage IIIA	T3	M0	N1
TCGA-HU-A4H3	56	0	3	Stage IIIC	T4a	M0	N3b
TCGA-VQ-A8PZ	56	0	3	Stage II	T3	M0	N0

TCGA-BR-8285	57	0	3	Stage IIIC	T4a	M0	N3a
TCGA-BR-6458	57	0	3	Stage IIB	T3	M0	N1
TCGA-BR-6709	57	0	3	Stage IIIB	T3	M0	N3a
TCGA-RD-A8N4	58	0	3	Stage IIIA	T3	M0	N1
TCGA-BR-8296	58	0	3	Stage IIIC	T4b	M0	N2
TCGA-CG-4477	58	0	3	Stage IB	T2a	M0	N0
TCGA-HU-8602	58	0	3	Stage IIB	T4a	M0	N0
TCGA-RD-A8N2	59	0	3	Stage IB	T2	M0	N0
TCGA-BR-4363	60	0	3	unknow	T3	MX	NX
TCGA-HU-A4GJ	60	0	3	Stage IIIC	T4	M0	N3a
TCGA-D7-A4Z0	60	0	3	Stage IIB	T2	M0	N2
TCGA-BR-8295	60	0	3	Stage IIB	T4a	M0	N0
TCGA-CD-8524	61	0	3	Stage II	T3	M0	N0
TCGA-BR-8592	63	0	3	Stage IIIB	T4b	M0	N1
TCGA-RD-A8N9	63	0	3	Stage II	T2	M0	N1
TCGA-BR-6852	64	0	3	Stage IIA	T3	M0	N0
TCGA-D7-6519	64	0	3	unknow	T2	M0	N1
TCGA-BR-8487	64	0	3	Stage IIA	T3	M0	N0
TCGA-BR-6566	64	0	3	Stage IIA	T3	M0	N0
TCGA-IN-A7NR	64	0	3	Stage IV	T3	M1	N3
TCGA-BR-4361	66	0	3	Stage IIIA	T4	M0	N0

TCGA-CD-8531	66	0	3	Stage IIIA	T3	M0	N1
TCGA-BR-8382	67	0	3	Stage IIIC	T4b	M0	N3a
TCGA-CG-4474	67	0	3	Stage IV	T4a	M1	N2
TCGA-VQ-A91E	67	0	3	Stage IIIB	T4b	M0	N0
TCGA-CG-5722	67	0	3	Stage IV	T3	M1	N2
TCGA-BR-8687	67	0	3	Stage IIIC	T4b	M0	N2
TCGA-BR-8485	68	0	3	Stage IIIC	T4	M0	N3a
TCGA-RD-A7BW	68	0	3	Stage IB	T2	M0	N0
TCGA-CG-4440	68	0	3	Stage IV	T3	M1	N3
TCGA-BR-6705	68	0	3	Stage IIIB	T3	M0	N3a
TCGA-D7-A4YV	69	0	3	Stage IIB	T3	M0	N1
TCGA-CG-4465	69	0	3	Stage IV	T4	M1	N3
TCGA-BR-7704	69	0	3	Stage II	T3	M0	N0
TCGA-BR-7707	69	0	3	Stage IB	T2	M0	N0
TCGA-BR-8078	70	0	3	Stage IIB	T4a	M0	N0
TCGA-BR-A4QI	70	0	3	Stage IIA	T3	M0	N0
TCGA-BR-A4CR	70	0	3	Stage IIIC	T4b	M0	N3a
TCGA-BR-8365	70	0	3	Stage IIA	T3	M0	N0
TCGA-HU-A4GX	70	0	3	Stage IIIC	T4b	M0	N2
TCGA-BR-8361	71	0	3	Stage IIIC	T4b	M0	N2
TCGA-D7-A6EV	71	0	3	Stage IIB	T2	M0	N2

TCGA-HU-A4GT	71	0	3	Stage IIA	T3	M0	N0
TCGA-BR-A4J8	71	0	3	Stage IIIB	T3	M0	N3a
TCGA-HJ-7597	71	0	3	Stage IB	T2b	MX	N0
TCGA-EQ-8122	71	0	3	Stage IIB	T3	MX	N1
TCGA-HU-A4G8	71	0	3	Stage IIB	T3	M0	N1
TCGA-D7-A6EX	72	0	3	Stage IIIA	T3	M0	N2
TCGA-D7-A6EY	72	0	3	Stage IIIB	T3	M0	N3a
TCGA-BR-A4PD	72	0	3	Stage IIB	T4a	M0	N0
TCGA-BR-8284	72	0	3	Stage IIIC	T4a	M0	N3a
TCGA-VQ-A8PU	72	0	3	Stage IIIA	T4a	M0	N1
TCGA-BR-8080	72	0	3	Stage IIIC	T4	M0	N3a
TCGA-CG-4462	72	0	3	Stage IV	T3	M1	N3
TCGA-BR-8363	73	0	3	Stage IB	T2	M0	N0
TCGA-CD-8526	73	0	3	Stage IIIA	T3	M0	N1
TCGA-IN-A7NT	73	0	3	Stage IIB	T3	M0	N1
TCGA-BR-8677	74	0	3	Stage IIIB	T3	M0	N3a
TCGA-BR-4362	74	0	3	unknow	TX	M0	N1
TCGA-CG-4301	75	0	3	Stage IV	T4	M1	N1
TCGA-BR-8060	75	0	3	Stage IIB	T2	M0	N2
TCGA-HF-A5NB	75	0	3	Stage IIIC	T4a	M0	N3a
TCGA-HU-A4GY	76	0	3	Stage IIIA	T4	M0	N1

TCGA-BR-4255	76	0	3	Stage IIIA	T3	M0	N1
TCGA-BR-8369	76	0	3	Stage IIIB	T3	M0	N3a
TCGA-BR-4368	78	0	3	Stage IV	T4	M0	N2
TCGA-IN-AB1X	78	0	3	Stage IIA	T3	M0	N0
TCGA-CD-5803	78	0	3	Stage II	T3	M0	N0
TCGA-BR-6452	78	0	3	Stage IIA	T3	M0	N0
TCGA-RD-A8N6	78	0	3	Stage IIIA	T2	M0	N2
TCGA-D7-A6F0	79	0	3	Stage IB	T2	M0	N0
TCGA-CD-A48C	79	0	3	Stage IIB	T3	M0	N1
TCGA-ZQ-A9CR	79	0	3	Stage IIIC	T4a	MX	N3
TCGA-BR-8366	80	0	3	Stage IIA	T3	M0	N0
TCGA-BR-4253	80	0	3	Stage IIIA	T3	M0	N1
TCGA-RD-A8NB	80	0	3	Stage IIIA	T3	M0	N1
TCGA-CD-8525	82	0	3	Stage IIIA	T3	M0	N1
TCGA-CG-5733	83	0	3	Stage IIIA	T2	M0	N2
TCGA-BR-8368	84	0	3	Stage IB	T2	M0	N0
TCGA-MX-A5UJ	86	0	3	Stage IIIA	T3	M0	N2
TCGA-CG-5728	88	0	3	Stage IB	T2	M0	N0
TCGA-BR-A4J4	39	1	3	Stage IIIB	T4a	M0	N2
TCGA-CD-8534	41	1	3	Stage II	T3	M0	N0
TCGA-VQ-A8DT	43	1	3	Stage IIIB	T3	M0	N3a

TCGA-BR-4279	43	1	3	Stage II	T2a	M0	N1
TCGA-D7-8570	44	1	3	Stage IIIB	T3	M0	N3a
TCGA-CD-5799	45	1	3	Stage II	T2	M0	N1
TCGA-HU-A4G2	45	1	3	Stage IIB	T3	M0	N1
TCGA-RD-A7BS	46	1	3	Stage IIIA	T3	M0	N1
TCGA-BR-A4IV	47	1	3	Stage IIIB	T4a	M0	N2
TCGA-CD-8533	48	1	3	Stage II	T3	M0	N0
TCGA-FP-8210	48	1	3	Stage IIIA	T3	M0	N1
TCGA-BR-8286	49	1	3	Stage II	T3	M0	N0
TCGA-CG-4472	49	1	3	Stage IV	T4	M0	N1
TCGA-BR-A4J7	49	1	3	Stage IIB	T4a	M0	NX
TCGA-FP-8209	49	1	3	Stage IB	T2a	M0	N0
TCGA-IN-7806	50	1	3	Stage IIB	T3	M0	N1
TCGA-BR-8381	51	1	3	Stage IIB	T3	M0	N1
TCGA-B7-A5TK	51	1	3	Stage IIIA	T4	M0	unknow
TCGA-CD-8530	51	1	3	Stage II	T3	M0	N0
TCGA-BR-A453	51	1	3	Stage IV	T4a	M1	N3a
TCGA-3M-AB47	51	1	3	Stage IIIB	T3	MX	N2
TCGA-CD-A487	51	1	3	Stage IIB	T3	M0	N1
TCGA-B7-A5TI	52	1	3	Stage IIIC	T4	M0	N3
TCGA-BR-8682	52	1	3	Stage IIB	T4a	M0	N0

TCGA-D7-6524	53	1	3	Stage II	T2	M0	N1
TCGA-D7-6520	53	1	3	Stage IIIA	T2b	M0	N2
TCGA-D7-6818	53	1	3	Stage IIIA	T2	M0	N3
TCGA-BR-6453	54	1	3	Stage IIA	T2	M0	N1
TCGA-R5-A804	54	1	3	Stage IV	T3	M1	N2
TCGA-BR-8380	55	1	3	Stage IIIC	T4a	M0	N3a
TCGA-BR-A4J9	55	1	3	Stage IIA	T3	M0	N0
TCGA-BR-8367	55	1	3	Stage IIIB	T3	M0	N3a
TCGA-FP-A4BE	55	1	3	Stage IIA	T3	M0	N0
TCGA-CG-4438	56	1	3	Stage IV	T4	M0	N2
TCGA-VQ-A8PM	56	1	3	Stage IV	T4	M0	N3
TCGA-BR-A4J5	56	1	3	Stage IIIA	T4a	M0	N1
TCGA-BR-4187	56	1	3	unknow	TX	M0	NX
TCGA-HU-A4GD	56	1	3	Stage IIB	T3	M0	N1
TCGA-RD-A8MV	56	1	3	Stage IIIB	T3	M0	N2
TCGA-BR-8589	56	1	3	Stage IIIB	T4b	M0	N1
TCGA-D7-A747	57	1	3	Stage IIB	T3	M0	N1
TCGA-D7-8573	57	1	3	Stage IIA	T3	M0	N0
TCGA-BR-A452	57	1	3	Stage IIIA	T4a	M0	N1
TCGA-BR-8289	57	1	3	Stage IV	T4	M1	N3a
TCGA-D7-6522	58	1	3	Stage IB	T2	M0	N0

TCGA-D7-6525	58	1	3	Stage IIIA	T2	M0	N2
TCGA-BR-A4IY	58	1	3	Stage IIB	T4a	M0	N0
TCGA-BR-A4CQ	58	1	3	Stage IIIA	T4a	M0	N1
TCGA-CG-5717	58	1	3	Stage II	T2b	M0	N1
TCGA-BR-6454	58	1	3	Stage IIA	T3	M0	N0
TCGA-CD-A489	58	1	3	Stage IIA	T3	M0	N0
TCGA-CG-5721	58	1	3	Stage IV	T4	M0	N1
TCGA-BR-8676	59	1	3	Stage IIIB	T3	M0	N3a
TCGA-BR-6455	59	1	3	Stage IIB	T3	M0	N1
TCGA-CD-8535	59	1	3	Stage IIIA	T3	M0	N1
TCGA-VQ-AA6K	59	1	3	Stage IIIC	T4a	M0	N3a
TCGA-BR-7723	59	1	3	Stage IIIB	T3	M0	N3a
TCGA-BR-7959	59	1	3	Stage IIIA	T4a	M0	N1
TCGA-CG-5724	59	1	3	Stage IV	T3	M1	N3
TCGA-IN-7808	59	1	3	unknow	T3	M1	N3
TCGA-BR-6563	60	1	3	Stage IIB	T3	M0	N1
TCGA-CD-5813	60	1	3	Stage II	T3	M0	N0
TCGA-FP-A8CX	60	1	3	Stage IIIC	T4b	MX	N3a
TCGA-BR-7958	60	1	3	Stage IIIB	T4b	M0	N0
TCGA-D7-A4YY	61	1	3	Stage IIIB	T3	M0	N3a
TCGA-VQ-A91Q	61	1	3	Stage IV	T3	M0	N3

TCGA-BR-8371	62	1	3	Stage IIIB	T3	M0	N3a
TCGA-D7-A6F2	62	1	3	Stage IB	T2	M0	N0
TCGA-BR-8590	62	1	3	Stage IIIC	T4b	M0	N2
TCGA-BR-7722	62	1	3	Stage IIB	T3	M0	N1
TCGA-B7-5818	62	1	3	Stage IB	T2	M0	N0
TCGA-D7-6817	63	1	3	Stage IIIA	T2b	M0	N3
TCGA-IN-A6RP	63	1	3	unknow	T1b	M0	N3
TCGA-BR-6706	63	1	3	Stage IIIA	T3	M0	N1
TCGA-BR-A4J1	63	1	3	Stage IIIA	T4a	M0	N1
TCGA-BR-8372	63	1	3	Stage IIIC	T4b	M0	N3a
TCGA-D7-A4YX	63	1	3	Stage IIB	T3	M0	N1
TCGA-IN-A6RJ	64	1	3	Stage IA	T1b	M0	N0
TCGA-BR-8370	64	1	3	Stage IIIB	T3	M0	N3a
TCGA-BR-7196	64	1	3	Stage IV	T3	M1	N3a
TCGA-BR-6802	65	1	3	Stage IIIA	T3	M0	N2
TCGA-BR-8362	65	1	3	Stage IIIC	T4b	M0	N3a
TCGA-BR-A4QM	65	1	3	Stage IIIA	T3	M0	N2
TCGA-VQ-A8PT	65	1	3	Stage IIIB	T4b	M0	N0
TCGA-VQ-A8PC	65	1	3	Stage IIIA	T3	M0	N1
TCGA-D7-6521	65	1	3	unknow	T2b	M0	N2
TCGA-CG-5727	66	1	3	Stage II	T3	M0	N0

TCGA-RD-A7BT	66	1	3	Stage IV	T3	M0	N3
TCGA-MX-A663	66	1	3	Stage IIA	T3	M0	N0
TCGA-D7-A6EZ	66	1	3	Stage IIIA	T3	M0	N2
TCGA-VQ-A8P5	67	1	3	Stage IIA	T3	M0	N0
TCGA-VQ-A91Y	67	1	3	Stage IIIC	T4a	M0	N3b
TCGA-FP-A4BF	68	1	3	Stage IIIA	T3	M0	N2
TCGA-VQ-A8E0	68	1	3	Stage IIIA	T3	M0	N2
TCGA-CG-5734	68	1	3	Stage IIIA	T3	M0	N1
TCGA-IN-8663	68	1	3	Stage IIB	T2	M0	N2
TCGA-VQ-AA6I	68	1	3	Stage IIIB	T3	M0	N3
TCGA-HU-A4HB	68	1	3	Stage IIB	T2	M0	N2
TCGA-BR-8686	69	1	3	Stage IIIB	T4b	M0	N1
TCGA-BR-8384	69	1	3	Stage IIIC	T4b	M0	N2
TCGA-CG-4476	69	1	3	Stage IIIC	T4b	M0	N3a
TCGA-IN-A7NU	69	1	3	Stage IIIB	T3	M0	N3
TCGA-CD-5801	69	1	3	Stage IIIA	T3	M0	N1
TCGA-BR-6457	69	1	3	Stage IIA	T3	M0	N0
TCGA-CG-4305	69	1	3	Stage II	T2a	M0	N1
TCGA-CG-4469	70	1	3	Stage IV	T3	M0	N3
TCGA-RD-A8N1	70	1	3	Stage IIIB	T3	M0	N2
TCGA-BR-A4J2	70	1	3	Stage IIB	T4a	M0	N0

TCGA-VQ-A94U	70	1	3	Stage IIB	T4a	M0	N0
TCGA-VQ-A8DZ	70	1	3	Stage IV	T4	M1	N3
TCGA-BR-A44U	70	1	3	Stage IIIB	T3	M0	N3a
TCGA-F1-A448	70	1	3	Stage IIIB	T3	M0	N3b
TCGA-HU-8608	70	1	3	Stage IIIB	T4a	M0	N2
TCGA-CG-5720	71	1	3	Stage IB	T2	M0	N0
TCGA-R5-A805	71	1	3	Stage IIIB	T3	M0	N2
TCGA-CG-4455	72	1	3	Stage II	T2b	M0	N1
TCGA-CG-5725	72	1	3	Stage IB	T2	M0	N0
TCGA-BR-A4PF	72	1	3	Stage IIIB	T4a	M0	N2
TCGA-D7-8574	72	1	3	Stage IIIA	T2	M0	N3a
TCGA-RD-A8MW	72	1	3	Stage IIIB	T3	M0	N2
TCGA-HU-8245	72	1	3	Stage IIA	T3	M0	N0
TCGA-HU-A4H0	72	1	3	Stage IIIC	T4a	M0	N3b
TCGA-BR-8059	72	1	3	Stage III	T4	M0	N1
TCGA-HU-A4GU	73	1	3	Stage IIB	T3	M0	N1
TCGA-VQ-A94T	73	1	3	Stage IIIB	T3	M0	N2
TCGA-KB-A6F5	73	1	3	Stage IIIA	T3	M0	N2
TCGA-D7-A4YU	73	1	3	Stage IIIB	T3	M0	N3a
TCGA-CD-8536	74	1	3	Stage II	T3	M0	N0
TCGA-BR-7851	74	1	3	Stage IIB	T4a	M0	N0

TCGA-HU-A4GC	74	1	3	Stage IIIB	T4a	M0	N2
TCGA-D7-5579	74	1	3	Stage IIIA	T3	M0	N2
TCGA-HU-A4G6	74	1	3	Stage IA	T1b	M0	N0
TCGA-VQ-A91X	74	1	3	Stage IIIB	T3	M0	N2
TCGA-VQ-A8PO	74	1	3	Stage IIB	T4a	M0	N0
TCGA-D7-6518	75	1	3	Stage IIIA	T2	M0	N3
TCGA-BR-6707	75	1	3	Stage IIA	T3	M0	N0
TCGA-BR-4369	75	1	3	unknow	TX	M0	NX
TCGA-BR-8683	75	1	3	Stage IIIB	T4a	M0	N2
TCGA-D7-8575	75	1	3	Stage IIIA	T3	M0	N2
TCGA-VQ-AA6J	75	1	3	Stage IIIB	T4a	M0	N2
TCGA-CG-4475	76	1	3	Stage IIB	T3	M0	N1
TCGA-CD-A4MG	76	1	3	Stage IIA	T3	M0	N0
TCGA-CG-4444	76	1	3	Stage IIIA	T2b	M0	N2
TCGA-VQ-A8PF	76	1	3	Stage IIIB	T3	M0	N3b
TCGA-FP-7916	77	1	3	Stage IIIC	T4a	MX	N3a
TCGA-BR-A4CS	77	1	3	Stage IIIC	T4b	M0	N3a
TCGA-FP-7998	77	1	3	Stage IIIC	T4a	MX	N3a
TCGA-MX-A5UG	78	1	3	Stage IIIA	T3	M0	N1
TCGA-BR-4367	78	1	3	unknow	TX	M0	N1
TCGA-KB-A93J	78	1	3	Stage II	T2b	M0	N1

TCGA-VQ-A8PE	78	1	3	Stage IIIB	T3	M0	N3a
TCGA-BR-8591	79	1	3	Stage IIIC	T4a	M0	N3a
TCGA-F1-6874	79	1	3	Stage IB	T2	M0	N0
TCGA-VQ-A8E3	79	1	3	Stage IIA	T3	M0	N0
TCGA-D7-5578	80	1	3	Stage IIIA	T3	M0	N2
TCGA-RD-A7C1	82	1	3	Stage IB	T2	M0	N0
TCGA-CG-5723	83	1	3	Stage II	T2	M0	N1
TCGA-IN-A6RR	84	1	3	unknow	T3	M0	N1
TCGA-CG-4442	85	1	3	Stage IB	T2	M0	N0
TCGA-VQ-A923	90	1	3	Stage IIIA	T3	M0	N1
TCGA-CG-4306	90	1	3	Stage IV	T3	M1	N2

Supplementary Table 2| The detailed clinical feature of each sample obtained from GEO dataset (GSE84437).

ID	Age (Y)	Gender	T	N
GSM2235936	63	0	1	0
GSM2235938	62	0	1	0
GSM2235988	54	0	1	0
GSM2235720	56	1	1	0
GSM2235781	56	1	1	0
GSM2235802	60	1	1	0
GSM2235851	81	1	1	0
GSM2235942	67	1	1	0
GSM2235709	54	0	2	0
GSM2235712	70	0	2	0
GSM2235837	56	0	2	0
GSM2235939	42	0	2	0
GSM2236023	61	0	2	0
GSM2235784	80	1	2	0
GSM2235788	56	1	2	0
GSM2235840	71	1	2	0
GSM2235968	52	1	2	0
GSM2236039	56	1	2	0
GSM2235785	64	0	3	0

GSM2235797	79	0	3	0
GSM2235908	32	0	3	0
GSM2235973	59	0	3	0
GSM2235697	49	1	3	0
GSM2235701	61	1	3	0
GSM2235803	46	1	3	0
GSM2235813	64	1	3	0
GSM2235944	47	1	3	0
GSM2236014	68	1	3	0
GSM2236028	68	1	3	0
GSM2236033	69	1	3	0
GSM2236048	64	1	3	0
GSM2236091	77	1	3	0
GSM2236093	59	1	3	0
GSM2235565	49	0	4	0
GSM2235577	32	0	4	0
GSM2235625	53	0	4	0
GSM2235631	66	0	4	0
GSM2235637	75	0	4	0
GSM2235725	65	0	4	0
GSM2235790	67	0	4	0

GSM2235795	73	0	4	0
GSM2235907	77	0	4	0
GSM2235933	57	0	4	0
GSM2236020	70	0	4	0
GSM2236031	52	0	4	0
GSM2236054	49	0	4	0
GSM2236082	53	0	4	0
GSM2236086	36	0	4	0
GSM2236088	61	0	4	0
GSM2236089	69	0	4	0
GSM2235560	58	1	4	0
GSM2235570	77	1	4	0
GSM2235582	65	1	4	0
GSM2235593	56	1	4	0
GSM2235710	54	1	4	0
GSM2235713	59	1	4	0
GSM2235776	80	1	4	0
GSM2235779	63	1	4	0
GSM2235804	64	1	4	0
GSM2235806	55	1	4	0
GSM2235825	69	1	4	0

GSM2235829	52	1	4	0
GSM2235862	66	1	4	0
GSM2235863	67	1	4	0
GSM2235881	72	1	4	0
GSM2235884	44	1	4	0
GSM2236030	63	1	4	0
GSM2236032	44	1	4	0
GSM2236034	55	1	4	0
GSM2236055	55	1	4	0
GSM2236056	54	1	4	0
GSM2236064	70	1	4	0
GSM2236068	44	1	4	0
GSM2236078	60	1	4	0
GSM2236080	62	1	4	0
GSM2236085	55	1	4	0
GSM2236090	86	1	4	0
GSM2236092	58	1	4	0
GSM2236094	64	1	4	0
GSM2236095	51	1	4	0
GSM2235730	68	0	1	1
GSM2235794	55	1	1	1

GSM2236036	62	1	1	1
GSM2235707	63	0	2	1
GSM2235731	63	0	2	1
GSM2235853	58	0	2	1
GSM2235886	76	0	2	1
GSM2235941	36	0	2	1
GSM2235969	72	0	2	1
GSM2235979	54	0	2	1
GSM2236013	67	0	2	1
GSM2236059	75	0	2	1
GSM2236061	68	0	2	1
GSM2236063	48	0	2	1
GSM2235597	68	1	2	1
GSM2235613	68	1	2	1
GSM2235714	61	1	2	1
GSM2235721	41	1	2	1
GSM2235732	74	1	2	1
GSM2235801	50	1	2	1
GSM2235843	60	1	2	1
GSM2235850	51	1	2	1
GSM2235855	68	1	2	1

GSM2236029	44	1	2	1
GSM2236057	62	1	2	1
GSM2236058	62	1	2	1
GSM2236074	62	1	2	1
GSM2236079	67	1	2	1
GSM2235568	58	0	3	1
GSM2235571	85	0	3	1
GSM2235602	61	0	3	1
GSM2235879	68	0	3	1
GSM2235901	66	0	3	1
GSM2235954	39	0	3	1
GSM2235976	67	0	3	1
GSM2235994	42	0	3	1
GSM2236000	64	0	3	1
GSM2236008	64	0	3	1
GSM2236025	68	0	3	1
GSM2236040	71	0	3	1
GSM2235556	61	1	3	1
GSM2235564	61	1	3	1
GSM2235575	59	1	3	1
GSM2235579	74	1	3	1

GSM2235585	71	1	3	1
GSM2235596	63	1	3	1
GSM2235598	74	1	3	1
GSM2235604	75	1	3	1
GSM2235608	55	1	3	1
GSM2235623	56	1	3	1
GSM2235624	59	1	3	1
GSM2235627	68	1	3	1
GSM2235628	43	1	3	1
GSM2235632	60	1	3	1
GSM2235716	46	1	3	1
GSM2235733	72	1	3	1
GSM2235740	69	1	3	1
GSM2235746	53	1	3	1
GSM2235751	40	1	3	1
GSM2235770	85	1	3	1
GSM2235783	71	1	3	1
GSM2235800	62	1	3	1
GSM2235822	55	1	3	1
GSM2235839	63	1	3	1
GSM2235965	58	1	3	1

GSM2235983	46	1	3	1
GSM2235993	27	1	3	1
GSM2236024	54	1	3	1
GSM2236026	65	1	3	1
GSM2236043	48	1	3	1
GSM2236050	65	1	3	1
GSM2236062	69	1	3	1
GSM2236069	69	1	3	1
GSM2236070	71	1	3	1
GSM2236076	63	1	3	1
GSM2236087	68	1	3	1
GSM2235572	68	0	4	1
GSM2235603	60	0	4	1
GSM2235605	54	0	4	1
GSM2235619	55	0	4	1
GSM2235634	64	0	4	1
GSM2235695	42	0	4	1
GSM2235702	74	0	4	1
GSM2235704	53	0	4	1
GSM2235728	40	0	4	1
GSM2235734	45	0	4	1

GSM2235743	64	0	4	1
GSM2235744	38	0	4	1
GSM2235754	37	0	4	1
GSM2235755	62	0	4	1
GSM2235761	63	0	4	1
GSM2235763	74	0	4	1
GSM2235777	49	0	4	1
GSM2235786	50	0	4	1
GSM2235791	36	0	4	1
GSM2235792	60	0	4	1
GSM2235805	61	0	4	1
GSM2235817	68	0	4	1
GSM2235823	32	0	4	1
GSM2235841	84	0	4	1
GSM2235847	53	0	4	1
GSM2235885	73	0	4	1
GSM2235888	36	0	4	1
GSM2235906	70	0	4	1
GSM2235923	77	0	4	1
GSM2235928	56	0	4	1
GSM2235945	59	0	4	1

GSM2235951	61	0	4	1
GSM2235952	56	0	4	1
GSM2235953	58	0	4	1
GSM2235996	53	0	4	1
GSM2236002	68	0	4	1
GSM2236007	76	0	4	1
GSM2236027	80	0	4	1
GSM2236037	64	0	4	1
GSM2236049	44	0	4	1
GSM2236066	63	0	4	1
GSM2236071	54	0	4	1
GSM2235558	72	1	4	1
GSM2235569	79	1	4	1
GSM2235586	71	1	4	1
GSM2235587	72	1	4	1
GSM2235588	56	1	4	1
GSM2235595	74	1	4	1
GSM2235616	73	1	4	1
GSM2235629	78	1	4	1
GSM2235630	65	1	4	1
GSM2235696	60	1	4	1

GSM2235705	67	1	4	1
GSM2235708	66	1	4	1
GSM2235711	65	1	4	1
GSM2235715	58	1	4	1
GSM2235718	46	1	4	1
GSM2235750	70	1	4	1
GSM2235752	42	1	4	1
GSM2235753	72	1	4	1
GSM2235765	68	1	4	1
GSM2235768	46	1	4	1
GSM2235771	53	1	4	1
GSM2235774	47	1	4	1
GSM2235775	60	1	4	1
GSM2235808	59	1	4	1
GSM2235809	58	1	4	1
GSM2235810	54	1	4	1
GSM2235819	68	1	4	1
GSM2235820	75	1	4	1
GSM2235821	67	1	4	1
GSM2235828	55	1	4	1
GSM2235864	54	1	4	1

GSM2235865	44	1	4	1
GSM2235882	55	1	4	1
GSM2235889	66	1	4	1
GSM2235899	48	1	4	1
GSM2235900	46	1	4	1
GSM2235902	34	1	4	1
GSM2235903	50	1	4	1
GSM2235904	62	1	4	1
GSM2235905	63	1	4	1
GSM2235910	56	1	4	1
GSM2235922	68	1	4	1
GSM2235925	64	1	4	1
GSM2235927	65	1	4	1
GSM2235931	34	1	4	1
GSM2235943	65	1	4	1
GSM2235947	62	1	4	1
GSM2235948	36	1	4	1
GSM2235949	43	1	4	1
GSM2235950	45	1	4	1
GSM2235960	66	1	4	1
GSM2235961	55	1	4	1

GSM2235962	64	1	4	1
GSM2235980	66	1	4	1
GSM2235981	69	1	4	1
GSM2235987	69	1	4	1
GSM2235992	59	1	4	1
GSM2235997	74	1	4	1
GSM2235998	68	1	4	1
GSM2235999	60	1	4	1
GSM2236003	55	1	4	1
GSM2236004	66	1	4	1
GSM2236006	42	1	4	1
GSM2236009	66	1	4	1
GSM2236010	61	1	4	1
GSM2236041	50	1	4	1
GSM2236045	63	1	4	1
GSM2236046	64	1	4	1
GSM2236052	61	1	4	1
GSM2236075	44	1	4	1
GSM2235635	73	1	2	2
GSM2235921	57	1	2	2
GSM2236044	48	1	2	2

GSM2235563	60	0	3	2
GSM2235576	78	0	3	2
GSM2235957	69	0	3	2
GSM2235995	57	0	3	2
GSM2236084	59	0	3	2
GSM2235559	78	1	3	2
GSM2235599	62	1	3	2
GSM2235607	65	1	3	2
GSM2235615	68	1	3	2
GSM2235620	65	1	3	2
GSM2235622	50	1	3	2
GSM2235723	52	1	3	2
GSM2235735	53	1	3	2
GSM2235739	66	1	3	2
GSM2235769	64	1	3	2
GSM2235793	83	1	3	2
GSM2235826	79	1	3	2
GSM2235887	81	1	3	2
GSM2235959	61	1	3	2
GSM2235971	45	1	3	2
GSM2235982	51	1	3	2

GSM2235991	77	1	3	2
GSM2236011	64	1	3	2
GSM2236019	77	1	3	2
GSM2235561	33	0	4	2
GSM2235562	81	0	4	2
GSM2235609	73	0	4	2
GSM2235611	78	0	4	2
GSM2235618	73	0	4	2
GSM2235636	32	0	4	2
GSM2235722	63	0	4	2
GSM2235738	59	0	4	2
GSM2235747	53	0	4	2
GSM2235756	48	0	4	2
GSM2235787	58	0	4	2
GSM2235815	71	0	4	2
GSM2235824	49	0	4	2
GSM2235844	54	0	4	2
GSM2235849	61	0	4	2
GSM2235866	79	0	4	2
GSM2235867	69	0	4	2
GSM2235880	67	0	4	2

GSM2235883	66	0	4	2
GSM2235932	71	0	4	2
GSM2235937	54	0	4	2
GSM2235956	68	0	4	2
GSM2235963	58	0	4	2
GSM2235970	66	0	4	2
GSM2236001	48	0	4	2
GSM2236005	72	0	4	2
GSM2236042	69	0	4	2
GSM2236047	49	0	4	2
GSM2236053	41	0	4	2
GSM2236083	64	0	4	2
GSM2235566	44	1	4	2
GSM2235567	51	1	4	2
GSM2235573	52	1	4	2
GSM2235574	72	1	4	2
GSM2235578	68	1	4	2
GSM2235580	61	1	4	2
GSM2235584	71	1	4	2
GSM2235589	66	1	4	2
GSM2235600	53	1	4	2

GSM2235601	79	1	4	2
GSM2235606	38	1	4	2
GSM2235610	76	1	4	2
GSM2235612	69	1	4	2
GSM2235614	27	1	4	2
GSM2235617	54	1	4	2
GSM2235621	63	1	4	2
GSM2235626	46	1	4	2
GSM2235633	63	1	4	2
GSM2235700	59	1	4	2
GSM2235703	35	1	4	2
GSM2235706	71	1	4	2
GSM2235719	68	1	4	2
GSM2235726	68	1	4	2
GSM2235729	67	1	4	2
GSM2235736	61	1	4	2
GSM2235737	51	1	4	2
GSM2235741	49	1	4	2
GSM2235742	62	1	4	2
GSM2235745	53	1	4	2
GSM2235748	80	1	4	2

GSM2235757	44	1	4	2
GSM2235758	42	1	4	2
GSM2235759	54	1	4	2
GSM2235760	64	1	4	2
GSM2235762	65	1	4	2
GSM2235764	58	1	4	2
GSM2235766	61	1	4	2
GSM2235767	53	1	4	2
GSM2235772	60	1	4	2
GSM2235773	30	1	4	2
GSM2235782	62	1	4	2
GSM2235789	64	1	4	2
GSM2235798	76	1	4	2
GSM2235811	55	1	4	2
GSM2235827	47	1	4	2
GSM2235830	75	1	4	2
GSM2235845	51	1	4	2
GSM2235846	67	1	4	2
GSM2235848	52	1	4	2
GSM2235909	63	1	4	2
GSM2235924	54	1	4	2

GSM2235926	47	1	4	2
GSM2235929	74	1	4	2
GSM2235930	63	1	4	2
GSM2235934	63	1	4	2
GSM2235935	65	1	4	2
GSM2235940	63	1	4	2
GSM2235946	55	1	4	2
GSM2235955	48	1	4	2
GSM2235958	55	1	4	2
GSM2235964	64	1	4	2
GSM2235967	61	1	4	2
GSM2235974	63	1	4	2
GSM2235977	72	1	4	2
GSM2235978	65	1	4	2
GSM2236015	66	1	4	2
GSM2236016	53	1	4	2
GSM2236017	61	1	4	2
GSM2236018	36	1	4	2
GSM2236038	65	1	4	2
GSM2236051	34	1	4	2
GSM2236065	58	1	4	2

GSM2236067	47	1	4	2
GSM2236073	60	1	4	2
GSM2236077	68	1	4	2
GSM2235986	53	0	3	3
GSM2235698	34	1	3	3
GSM2235724	74	1	3	3
GSM2235975	59	1	3	3
GSM2236072	49	1	3	3
GSM2235557	47	0	4	3
GSM2235717	43	0	4	3
GSM2235818	74	0	4	3
GSM2235854	66	0	4	3
GSM2235878	51	0	4	3
GSM2235985	69	0	4	3
GSM2235699	68	1	4	3
GSM2235727	71	1	4	3
GSM2235749	65	1	4	3
GSM2235778	61	1	4	3
GSM2235780	75	1	4	3
GSM2235796	62	1	4	3
GSM2235799	62	1	4	3

GSM2235807	70	1	4	3
GSM2235812	68	1	4	3
GSM2235814	66	1	4	3
GSM2235816	48	1	4	3
GSM2235831	81	1	4	3
GSM2235838	65	1	4	3
GSM2235842	72	1	4	3
GSM2235852	69	1	4	3
GSM2235966	40	1	4	3
GSM2235972	67	1	4	3
GSM2235984	69	1	4	3
GSM2236012	33	1	4	3
GSM2236021	32	1	4	3
GSM2236022	53	1	4	3
GSM2236060	47	1	4	3

Supplementary Table 3| The detailed information of each immune-related genes obtained from the Immport website.

Symbol	ID	Name	Synonyms	Chromosome	Category
AZGP1	563	alpha-2-glycoprotein 1, zinc-binding	ZA2G|ZAG	7	Antigen_Processing_and_Presentation
B2M	567	beta-2-microglobulin	-	15	Antigen_Processing_and_Presentation
CALR	811	calreticulin	CRT|FLJ26680|RO|SSA|cC1qR	19	Antigen_Processing_and_Presentation
CANX	821	calnexin	CNX|FLJ26570|IP90|P90	5	Antigen_Processing_and_Presentation
CD1A	909	CD1a molecule	CD1|FCB6|HTA1|R4|T6	1	Antigen_Processing_and_Presentation
CD1B	910	CD1b molecule	CD1|CD1A|MGC125990|MGC125991|R1	1	Antigen_Processing_and_Presentation
CD1C	911	CD1c molecule	BDCA1|CD1|CD1A|R7	1	Antigen_Processing_and_Presentation
CD1D	912	CD1d molecule	CD1A|MGC34622|R3	1	Antigen_Processing_and_Presentation
CD1E	913	CD1e molecule	CD1A|R2	1	Antigen_Processing_and_Presentation
CD4	920	CD4 molecule	CD4mut	12	Antigen_Processing_and_Presentation
CD8A	925	CD8a molecule	CD8|Leu2|MAL|p32	2	Antigen_Processing_and_Presentation
CD8B	926	CD8b molecule	CD8B1|LYT3|Leu2|Ly3|MGC119115	2	Antigen_Processing_and_Presentation
CD74	972	CD74 molecule, major histocompatibility complex, class II invariant chain	DHLAG|HLADG|Ia-GAMMA	5	Antigen_Processing_and_Presentation
CREB1	1385	cAMP responsive element binding protein 1	CREB|MGC9284	2	Antigen_Processing_and_Presentation
CTSB	1508	cathepsin B	APPS|CPSB	8	Antigen_Processing_and_Presentation
CTSE	1510	cathepsin E	CATE	1	Antigen_Processing_and_Presentation
CTSL1	1514	cathepsin L1	CATL|CTSL|FLJ31037|MEP	9	Antigen_Processing_and_Presentation
CTSS	1520	cathepsin S	MGC3886	1	Antigen_Processing_and_Presentation
FCER1G	2207	Fc fragment of IgE, high affinity I,	FCRG	1	Antigen_Processing_and_Presentation

		receptor for; gamma polypeptide			
FCGRT	2217	Fc fragment of IgG, receptor, transporter, alpha	FCRN|alpha-chain	19	Antigen_Processing_and_Presentation
PDIA3	2923	protein disulfide isomerase family A, member 3	ER60|ERp57|ERp60|ERp61|GRP57|GRP58|HsT17083|P58|PI-PLC	15	Antigen_Processing_and_Presentation
HFE	3077	hemochromatosis	HFE1|HH|HLA-H|MGC103790|MVCD7|dJ221C16.10.1	6	Antigen_Processing_and_Presentation
HLA-A	3105	major histocompatibility complex, class I, A	FLJ26655|HLAA	6	Antigen_Processing_and_Presentation
HLA-B	3106	major histocompatibility complex, class I, B	AS|HLA-B-7301|HLA-B73|HLAB|HLAC|MGC111087|SPDA1	6	Antigen_Processing_and_Presentation
HLA-C	3107	major histocompatibility complex, class I, C	D6S204|FLJ27082|HLA-Cw|HLA-Cw12|HLA-JY3|HLC-C|PSORS1	6	Antigen_Processing_and_Presentation
HLA-DMA	3108	major histocompatibility complex, class II, DM alpha	D6S222E|DMA|HLADM|RING6	6	Antigen_Processing_and_Presentation
HLA-DMB	3109	major histocompatibility complex, class II, DM beta	D6S221E|RING7	6	Antigen_Processing_and_Presentation
HLA-DOA	3111	major histocompatibility complex, class II, DO alpha	HLA-DNA|HLA-DZA|HLADZ	6	Antigen_Processing_and_Presentation
HLA-DOB	3112	major histocompatibility complex, class II, DO beta	DOB	6	Antigen_Processing_and_Presentation
HLA-DPA1	3113	major histocompatibility complex, class II, DP alpha 1	HLA-DP1A|HLADP|HLASB	6	Antigen_Processing_and_Presentation
HLA-DPB1	3115	major histocompatibility complex, class II, DP beta 1	DPB1|HLA-DP1B	6	Antigen_Processing_and_Presentation
HLA-DQA1	3117	major histocompatibility complex,	CD|CELIAC1|DQ-	6	Antigen_Processing_and_Presentation

		class II, DQ alpha 1	A1|FLJ27088|FLJ27328|GSE|HLA-DQA|MGC149527		
HLA-DQA2	3118	major histocompatibility complex, class II, DQ alpha 2	HLA-DXA	6	Antigen_Processing_and_Presentation
HLA-DQB1	3119	major histocompatibility complex, class II, DQ beta 1	CELIAC1|HLA-DQB|IDDM1	6	Antigen_Processing_and_Presentation
HLA-DRA	3122	major histocompatibility complex, class II, DR alpha	HLA-DRA1	6	Antigen_Processing_and_Presentation
HLA-DRB1	3123	major histocompatibility complex, class II, DR beta 1	DRB1|FLJ75017|FLJ76359|HLA-DR1B|HLA-DRB|HLA-DRB1*|SS1	6	Antigen_Processing_and_Presentation
HLA-DRB3	3125	major histocompatibility complex, class II, DR beta 3	HLA-DR3B|HLA-DR52|MGC117330	6	Antigen_Processing_and_Presentation
HLA-DRB4	3126	major histocompatibility complex, class II, DR beta 4	DRB4|HLA-DR4B	6	Antigen_Processing_and_Presentation
HLA-DRB5	3127	major histocompatibility complex, class II, DR beta 5	FLJ76359|HLA-DRB	6	Antigen_Processing_and_Presentation
HLA-E	3133	major histocompatibility complex, class I, E	DKFZp686P19218|EA1.2|EA2.1|HLA-6.2|MHC|QA1	6	Antigen_Processing_and_Presentation
HLA-F	3134	major histocompatibility complex, class I, F	CDA12|HLA-5.4|HLA-CDA12|HLAF	6	Antigen_Processing_and_Presentation
HLA-G	3135	major histocompatibility complex, class I, G	MHC-G	6	Antigen_Processing_and_Presentation
HLA-H	3136	major histocompatibility complex, class I, H (pseudogene)	HLAHP	6	Antigen_Processing_and_Presentation
MR1	3140	major histocompatibility complex, class I-related	HLALS	1	Antigen_Processing_and_Presentation

HSPA1A	3303	heat shock 70kDa protein 1A	FLJ54303|FLJ54370|FLJ54392|FLJ54408|FLJ75127|HSP70-1|HSP70-1A|HSP70I|HSP72|HSPA1|HSPA1B	6	Antigen_Processing_and_Presentation
HSPA1B	3304	heat shock 70kDa protein 1B	FLJ54328|HSP70-1B|HSP70-2|HSPA1A	6	Antigen_Processing_and_Presentation
HSPA1L	3305	heat shock 70kDa protein 1-like	HSP70-1L|HSP70-HOM|HSP70T|hum70t	6	Antigen_Processing_and_Presentation
HSPA2	3306	heat shock 70kDa protein 2	HSP70-2|HSP70-3	14	Antigen_Processing_and_Presentation
HSPA4	3308	heat shock 70kDa protein 4	APG-2|HS24/P52|MGC131852|RY|hsp70|hsp70RY	5	Antigen_Processing_and_Presentation
HSPA5	3309	heat shock 70kDa protein 5 (glucose-regulated protein, 78kDa)	BIP|FLJ26106|GRP78|MIF2	9	Antigen_Processing_and_Presentation
HSPA6	3310	heat shock 70kDa protein 6 (HSP70B')	-	1	Antigen_Processing_and_Presentation
HSPA8	3312	heat shock 70kDa protein 8	HSC54|HSC70|HSC71|HSP71|HSP73|HSPA10|LAP1|MGC131511|MGC29929|NIP71	11	Antigen_Processing_and_Presentation
HSP90AA1	3320	heat shock protein 90kDa alpha (cytosolic), class A member 1	FLJ31884|HSP86|HSP89A|HSP90A|HSP90N|HSPC1|HSPCA|HSPCAL1|HSPCAL4|HSPN|Hsp89|Hsp90|LAP2	14	Antigen_Processing_and_Presentation
HSP90AB1	3326	heat shock protein 90kDa alpha (cytosolic), class B member 1	D6S182|FLJ26984|HSP90-BETA|HSP90B|HSPC2|HSPCB	6	Antigen_Processing_and_Presentation
ICAM1	3383	intercellular adhesion molecule 1	BB2|CD54|P3.58	19	Antigen_Processing_and_Presentation
IFNA1	3439	interferon, alpha 1	IFL|IFN|IFN-ALPHA|IFNA13|IFNA@|MGC138207|MGC138505|MGC138507	9	Antigen_Processing_and_Presentation
IFNA2	3440	interferon, alpha 2	IFNA|INFA2|MGC125764|MGC125765	9	Antigen_Processing_and_Presentation

IFNA4	3441	interferon, alpha 4	INFA4|MGC142200	9	Antigen_Processing_and_Presentation
IFNA5	3442	interferon, alpha 5	INFA5	9	Antigen_Processing_and_Presentation
IFNA6	3443	interferon, alpha 6	-	9	Antigen_Processing_and_Presentation
IFNA7	3444	interferon, alpha 7	IFNA-J	9	Antigen_Processing_and_Presentation
IFNA8	3445	interferon, alpha 8	-	9	Antigen_Processing_and_Presentation
IFNA10	3446	interferon, alpha 10	MGC119878|MGC119879	9	Antigen_Processing_and_Presentation
IFNA13	3447	interferon, alpha 13	-	9	Antigen_Processing_and_Presentation
IFNA14	3448	interferon, alpha 14	LEIF2H|MGC125756|MGC125757	9	Antigen_Processing_and_Presentation
IFNA16	3449	interferon, alpha 16	-	9	Antigen_Processing_and_Presentation
IFNA17	3451	interferon, alpha 17	IFNA|INFA|LEIF2C1	9	Antigen_Processing_and_Presentation
IFNA21	3452	interferon, alpha 21	MGC126687|MGC126689	9	Antigen_Processing_and_Presentation
IFNG	3458	interferon, gamma	IFG|IFI	12	Antigen_Processing_and_Presentation
KIR2DL1	3802	killer cell immunoglobulin-like receptor, two domains, long cytoplasmic tail, 1	CD158A|KIR-K64|KIR221|NKAT|NKAT1|p58.1	19	Antigen_Processing_and_Presentation
KIR2DL2	3803	killer cell immunoglobulin-like receptor, two domains, long cytoplasmic tail, 2	CD158B1|CD158b|NKAT6|p58.2	19	Antigen_Processing_and_Presentation
KIR2DL3	3804	killer cell immunoglobulin-like receptor, two domains, long cytoplasmic tail, 3	CD158B2|CD158b|GL183|KIR-023GB|KIR-K7b|KIR-K7c|KIRCL23|MGC129943|NKAT|NKAT2|NKAT2A|NKAT2B|p58	19	Antigen_Processing_and_Presentation
KIR2DL4	3805	killer cell immunoglobulin-like receptor, two domains, long cytoplasmic tail, 4	CD158D|G9P|KIR103|KIR103AS	19	Antigen_Processing_and_Presentation
KIR2DS1	3806	killer cell immunoglobulin-like	CD158H|CD158a|p50.1	19	Antigen_Processing_and_Presentation

		receptor, two domains, short cytoplasmic tail, 1			
KIR2DS3	3808	killer cell immunoglobulin-like receptor, two domains, short cytoplasmic tail, 3	NKAT7	19	Antigen_Processing_and_Presentation
KIR2DS4	3809	killer cell immunoglobulin-like receptor, two domains, short cytoplasmic tail, 4	CD158I|KIR1D|KIR412|KKA3|MGC120019|MGC125315|MGC125317|NKAT8	19	Antigen_Processing_and_Presentation
KIR2DS5	3810	killer cell immunoglobulin-like receptor, two domains, short cytoplasmic tail, 5	CD158G|NKAT9	19	Antigen_Processing_and_Presentation
KIR3DL1	3811	killer cell immunoglobulin-like receptor, three domains, long cytoplasmic tail, 1	CD158E1|KIR|MGC119726|MGC119728|MGC126589|MGC126591|NKAT3|NKB1|NKB1B	19	Antigen_Processing_and_Presentation
KIR3DL2	3812	killer cell immunoglobulin-like receptor, three domains, long cytoplasmic tail, 2	CD158K|MGC125321|NKAT4|NKAT4B|p140	19	Antigen_Processing_and_Presentation
KLRC1	3821	killer cell lectin-like receptor subfamily C, member 1	CD159A|MGC13374|MGC59791|NKG2|NKG2A	12	Antigen_Processing_and_Presentation
KLRC2	3822	killer cell lectin-like receptor subfamily C, member 2	CD159c|MGC138244|NKG2-C|NKG2C	12	Antigen_Processing_and_Presentation
KLRC3	3823	killer cell lectin-like receptor subfamily C, member 3	NKG2-E|NKG2E	12	Antigen_Processing_and_Presentation
KLRD1	3824	killer cell lectin-like receptor subfamily D, member 1	CD94	12	Antigen_Processing_and_Presentation
LTA	4049	lymphotoxin alpha (TNF	LT|TNFB|TNFSF1	6	Antigen_Processing_and_Presentation

		superfamily, member 1)			
CIITA	4261	class II, major histocompatibility complex, transactivator	C2TA|CIITAIV|MHC2TA|NLRA	16	Antigen_Processing_and_Presentation
MICA	4276	MHC class I polypeptide-related sequence A	FLJ60820|MGC111087|PERB11.1	6	Antigen_Processing_and_Presentation
MICB	4277	MHC class I polypeptide-related sequence B	PERB11.2	6	Antigen_Processing_and_Presentation
NFYA	4800	nuclear transcription factor Y, alpha	CBF-A|CBF-B|FLJ11236|HAP2|NF-YA	6	Antigen_Processing_and_Presentation
NFYB	4801	nuclear transcription factor Y, beta	CBF-A|CBF-B|HAP3|NF-YB	12	Antigen_Processing_and_Presentation
NFYC	4802	nuclear transcription factor Y, gamma	CBF-C|CBFC|DKFZp667G242|FLJ45775|H1TF2A|HAP5|HSM|NF-YC	1	Antigen_Processing_and_Presentation
LGMN	5641	legumain	AEP|LGMN1|PRSC1	14	Antigen_Processing_and_Presentation
PSMB8	5696	proteasome (prosome, macropain) subunit, beta type, 8 (large multifunctional peptidase 7)	D6S216|D6S216E|LMP7|MGC1491|PSMB5i|RING10|beta5i	6	Antigen_Processing_and_Presentation
PSMC1	5700	proteasome (prosome, macropain) 26S subunit, ATPase, 1	MGC24583|MGC8541|P26S4|S4|p56	14	Antigen_Processing_and_Presentation
PSMC2	5701	proteasome (prosome, macropain) 26S subunit, ATPase, 2	MGC3004|MSS1|Nbla10058|S7	7	Antigen_Processing_and_Presentation
PSMC3	5702	proteasome (prosome,	MGC8487|TBP1	11	Antigen_Processing_and_Presentation

		macropain) 26S subunit, ATPase, 3			
PSMC4	5704	proteasome (prosome, macropain) 26S subunit, ATPase, 4	MGC13687|MGC23214|MGC8570|MIP224|S6|TBP7	19	Antigen_Processing_and_Presentation
PSMC5	5705	proteasome (prosome, macropain) 26S subunit, ATPase, 5	S8|SUG-1|SUG1|TBP10|TRIP1|p45|p45/SUG	17	Antigen_Processing_and_Presentation
PSMC6	5706	proteasome (prosome, macropain) 26S subunit, ATPase, 6	CADP44|MGC12520|P44|SUG2|p42	14	Antigen_Processing_and_Presentation
PSMD1	5707	proteasome (prosome, macropain) 26S subunit, non-ATPase, 1	MGC133040|MGC133041|P112|Rpn2|S1	2	Antigen_Processing_and_Presentation
PSMD2	5708	proteasome (prosome, macropain) 26S subunit, non-ATPase, 2	MGC14274|P97|Rpn1|S2|TRAP2	3	Antigen_Processing_and_Presentation
PSMD3	5709	proteasome (prosome, macropain) 26S subunit, non-ATPase, 3	P58|RPN3|S3	17	Antigen_Processing_and_Presentation
PSMD4	5710	proteasome (prosome, macropain) 26S subunit, non-ATPase, 4	AF|AF-1|ASF|MCB1|Rpn10|S5A|pUB-R5	1	Antigen_Processing_and_Presentation
PSMD5	5711	proteasome (prosome, macropain) 26S subunit, non-ATPase, 5	KIAA0072|MGC23145|S5B	9	Antigen_Processing_and_Presentation

PSMD7	5713	proteasome (prosome, macropain) 26S subunit, non-ATPase, 7	MOV34|P40|Rpn8|S12	16	Antigen_Processing_and_Presentation
PSMD8	5714	proteasome (prosome, macropain) 26S subunit, non-ATPase, 8	HIP6|HYPF|MGC1660|Nin1p|Rpn12|S14|p31	19	Antigen_Processing_and_Presentation
PSMD10	5716	proteasome (prosome, macropain) 26S subunit, non-ATPase, 10	dJ889N15.2|p28	X	Antigen_Processing_and_Presentation
PSMD11	5717	proteasome (prosome, macropain) 26S subunit, non-ATPase, 11	MGC3844|Rpn6|S9|p44.5	17	Antigen_Processing_and_Presentation
PSMD13	5719	proteasome (prosome, macropain) 26S subunit, non-ATPase, 13	HSPC027|Rpn9|S11|p40.5	11	Antigen_Processing_and_Presentation
PSME1	5720	proteasome (prosome, macropain) activator subunit 1 (PA28 alpha)	IFI5111|MGC8628|PA28A|PA28alpha|REGalpha	14	Antigen_Processing_and_Presentation
PSME1	5720	proteasome (prosome, macropain) activator subunit 1 (PA28 alpha)	IFI5111|MGC8628|PA28A|PA28alpha|REGalpha	14	Antigen_Processing_and_Presentation
PSME2	5721	proteasome (prosome, macropain) activator subunit 2 (PA28 beta)	PA28B|PA28beta|REGbeta	14	Antigen_Processing_and_Presentation
PSME2	5721	proteasome (prosome, macropain) activator subunit 2	PA28B|PA28beta|REGbeta	14	Antigen_Processing_and_Presentation

		(PA28 beta)			
RELB	5971	v-rel reticuloendotheliosis viral oncogene homolog B	I-REL|IREL	19	Antigen_Processing_and_Presentation
RFX5	5993	regulatory factor X, 5 (influences HLA class II expression)	-	1	Antigen_Processing_and_Presentation
RFXAP	5994	regulatory factor X-associated protein	-	13	Antigen_Processing_and_Presentation
SLC10A2	6555	solute carrier family 10 (sodium/bile acid cotransporter family), member 2	ASBT|ISBT|NTCP2	13	Antigen_Processing_and_Presentation
TAP1	6890	transporter 1, ATP-binding cassette, sub-family B (MDR/TAP)	ABC17|ABCB2|APT1|D6S114E|FLJ26666|FLJ41500|PSF1|RING4|TAP1*0102N|TAP1N	6	Antigen_Processing_and_Presentation
TAP2	6891	transporter 2, ATP-binding cassette, sub-family B (MDR/TAP)	ABC18|ABCB3|APT2|D6S217E|PSF2|RING11	6	Antigen_Processing_and_Presentation
TAPBP	6892	TAP binding protein (tapasin)	NGS17|TAPA|TPN|TPSN|tapasin	6	Antigen_Processing_and_Presentation
THBS1	7057	thrombospondin 1	THBS|THBS-1|TSP|TSP-1|TSP1	15	Antigen_Processing_and_Presentation
SHFM1	7979	split hand/foot malformation (ectrodactyly) type 1	DSS1|ECD|SEM1|SHFD1|SHSF1|Shfdg1	7	Antigen_Processing_and_Presentation
KLRC4	8302	killer cell lectin-like receptor subfamily C, member 4	FLJ17759|FLJ78582|NKG2-F|NKG2F	12	Antigen_Processing_and_Presentation
AP3B1	8546	adaptor-related protein complex 3, beta 1 subunit	ADTB3|ADTB3A|HPS|HPS2|PE	5	Antigen_Processing_and_Presentation
RFXANK	8625	regulatory factor X-associated ankyrin-containing protein	ANKRA1|BLS|F14150_1|MGC138628|RFX-B	19	Antigen_Processing_and_Presentation

PSMD6	9861	proteasome (prosome, macropain) 26S subunit, non-ATPase, 6	KIAA0107|Rpn7|S10|SGA-113M|p44S10	3	Antigen_Processing_and_Presentation
PSME3	10197	proteasome (prosome, macropain) activator subunit 3 (PA28 gamma; Ki)	Ki|PA28-gamma|PA28G|REG-GAMMA	17	Antigen_Processing_and_Presentation
PSMD14	10213	proteasome (prosome, macropain) 26S subunit, non-ATPase, 14	PAD1|POH1|rpn11	2	Antigen_Processing_and_Presentation
CLEC4M	10332	C-type lectin domain family 4, member M	CD209L|CD299|DC-SIGN2|DC-SIGNR|DCSIGNR|HP10347|L-SIGN|LSIGN|MGC129964|MGC47866	19	Antigen_Processing_and_Presentation
IFI30	10437	interferon, gamma-inducible protein 30	GILT|IFI-30|IP30|MGC32056	19	Antigen_Processing_and_Presentation
PROCR	10544	protein C receptor, endothelial (EPCR)	CCCA|CCD41|CD201|EPCR|MGC23024|bA42O4.2	20	Antigen_Processing_and_Presentation
ADRM1	11047	adhesion regulating molecule 1	ARM1|GP110|MGC29536|Rpn13	20	Antigen_Processing_and_Presentation
KIAA0368	23392	KIAA0368	ECM29|FLJ22036|KIAA1962|RP11-386D8.2	9	Antigen_Processing_and_Presentation
TRPC4AP	26133	transient receptor potential cation channel, subfamily C, member 4 associated protein	C20orf188|TRRP4AP|TRUSS	20	Antigen_Processing_and_Presentation
CD209	30835	CD209 molecule	CDSIGN|CLEC4L|DC-SIGN|DC-SIGN1|MGC129965	19	Antigen_Processing_and_Presentation
UBXN1	51035	UBX domain protein 1	2B28|UBXD10	11	Antigen_Processing_and_Presentation
ERAP1	51752	endoplasmic reticulum aminopeptidase 1	A-LAP|ALAP|APPILS|ARTS-1|ARTS1|ERAAP|ERAAP1|KIAA0525|PILS-	5	Antigen_Processing_and_Presentation

			AP|PILSAP		
TAPBPL	55080	TAP binding protein-like	FLJ10143|TAPBP-R|TAPBPR	12	Antigen_Processing_and_Presentation
KIR2DL5A	57292	killer cell immunoglobulin-like receptor, two domains, long cytoplasmic tail, 5A	CD158F|KIR2DL5|KIR2DL5.1|KIR2DL5.3	19	Antigen_Processing_and_Presentation
ERAP2	64167	endoplasmic reticulum aminopeptidase 2	FLJ23633|FLJ23701|FLJ23807|L-RAP|LRAP	5	Antigen_Processing_and_Presentation
ULBP3	79465	UL16 binding protein 3	RAET1N	6	Antigen_Processing_and_Presentation
ULBP2	80328	UL16 binding protein 2	N2DL2|RAET1H	6	Antigen_Processing_and_Presentation
ULBP1	80329	UL16 binding protein 1	RAET1I	6	Antigen_Processing_and_Presentation
KIR3DL3	115653	killer cell immunoglobulin-like receptor, three domains, long cytoplasmic tail, 3	CD158Z|KIR3DL7|KIR44|KIRC1	19	Antigen_Processing_and_Presentation
RAET1E	135250	retinoic acid early transcript 1E	LETAL|MGC125308|MGC125309|RAET1E2|ULBP4|bA350J20.7	6	Antigen_Processing_and_Presentation
RAET1L	154064	retinoic acid early transcript 1L	-	6	Antigen_Processing_and_Presentation
UBR1	197131	ubiquitin protein ligase E3 component n-recognin 1	JBS|MGC142065|MGC142067	15	Antigen_Processing_and_Presentation
RAET1G	353091	retinoic acid early transcript 1G	ULBP5	6	Antigen_Processing_and_Presentation
PDIA2	64714	protein disulfide isomerase family A, member 2	PDA2|PDI|PDIP|PDIR	16	Antigen_Processing_and_Presentation
HAMP	57817	hepcidin antimicrobial peptide	HEPC|HEPCIDIN|HFE2B|LEAP-1|LEAP1|PLTR	19	Antimicrobials
PI3	5266	peptidase inhibitor 3, skin-derived	ESI|MGC13613|SKALP|WAP3|WFDC14|cementoin	20	Antimicrobials
CAMP	820	cathelicidin antimicrobial peptide	CAP18|CRAMP|FALL-39|FALL39|HSD26|LL37	3	Antimicrobials
DEFB4	1673	defensin, beta 4	DEFB-2|DEFB102|DEFB2|HBD-2|SAP1	8	Antimicrobials

PPBP	5473	pro-platelet basic protein (chemokine (C-X-C motif) ligand 7)	B-TG1|Beta-TG|CTAP-III|CTAP3|CTAPIII|CXCL7|LA-PF4|LDGF|MDGF|NAP-2|PBP|SCYB7|TC1|TC2|TGB|TGB1|THBGB|THBGB1	4	Antimicrobials
REG3G	130120	regenerating islet-derived 3 gamma	MGC118998|MGC118999|MGC119001|PAP1B|PAPIB|REG-III|UNQ429	2	Antimicrobials
CXCL14	9547	chemokine (C-X-C motif) ligand 14	BMAC|BRAK|KS1|Kec|MGC10687|MIP-2g|NJAC|SCYB14|bolekine	5	Antimicrobials
CXCL16	58191	chemokine (C-X-C motif) ligand 16	CXCLG16|SR-PSOX|SRPSOX	17	Antimicrobials
SLPI	6590	secretory leukocyte peptidase inhibitor	ALK1|ALP|BLPI|HUSI|HUSI-I|MPI|WAP4|WFDC4	20	Antimicrobials
IL8	3576	interleukin 8	CXCL8|GCP-1|GCP1|LECT|LUCT|LYNAP|MDNCF|MONAP|NAF|NAP-1|NAP1	4	Antimicrobials
CXCL10	3627	chemokine (C-X-C motif) ligand 10	C7|IFI10|INP10|IP-10|SCYB10|crg-2|gIP-10|mob-1	4	Antimicrobials
CXCL9	4283	chemokine (C-X-C motif) ligand 9	CMK|Humig|MIG|SCYB9|crg-10	4	Antimicrobials
CXCL5	6374	chemokine (C-X-C motif) ligand 5	ENA-78|SCYB5	4	Antimicrobials
CXCL11	6373	chemokine (C-X-C motif) ligand 11	H174|I-TAC|IP-9|IP9|MGC102770|SCYB11|SCYB9B|b-R1	4	Antimicrobials
CXCL6	6372	chemokine (C-X-C motif) ligand 6 (granulocyte chemotactic protein 2)	CKA-3|GCP-2|GCP2|SCYB6	4	Antimicrobials
CXCL1	2919	chemokine (C-X-C motif) ligand 1	FSP|GRO1|GROa|MGSA|MGSA-a|NAP-	4	Antimicrobials

		(melanoma growth stimulating activity, alpha)	3|SCYB1		
CXCL12	6387	chemokine (C-X-C motif) ligand 12 (stromal cell-derived factor 1)	PBSF|SCYB12|SDF-1a|SDF-1b|SDF1|SDF1A|SDF1B|TLSF-a|TLSF-b|TPAR1	10	Antimicrobials
CXCL13	10563	chemokine (C-X-C motif) ligand 13	ANGIE|ANGIE2|BCA-1|BCA1|BLC|BLR1L|SCYB13	4	Antimicrobials
CXCL2	2920	chemokine (C-X-C motif) ligand 2	CINC-2a|GRO2|GROb|MGSA-b|MIP-2a|MIP2|MIP2A|SCYB2	4	Antimicrobials
PF4	5196	platelet factor 4	CXCL4|MGC138298|SCYB4	4	Antimicrobials
XCL1	6375	chemokine (C motif) ligand 1	ATAC|LPTN|LTN|SCM-1|SCM-1a|SCM1|SCYC1	1	Antimicrobials
CXCL3	2921	chemokine (C-X-C motif) ligand 3	CINC-2b|GRO3|GROg|MIP-2b|MIP2B|SCYB3	4	Antimicrobials
DEFB103A	55894	defensin, beta 103A	DEFB103|DEFB3|HBD-3|HBD3|HBP-3|HBP3	8	Antimicrobials
CCL13	6357	chemokine (C-C motif) ligand 13	CKb10|MCP-4|MGC17134|NCC-1|NCC1|SCYA13|SCYL1	17	Antimicrobials
CCL1	6346	chemokine (C-C motif) ligand 1	I-309|P500|SCYA1|SISe|TCA3	17	Antimicrobials
DEFB1	1672	defensin, beta 1	BD1|DEFB-1|DEFB101|HBD1|MGC51822	8	Antimicrobials
CCL8	6355	chemokine (C-C motif) ligand 8	HC14|MCP-2|MCP2|SCYA10|SCYA8	17	Antimicrobials
ELANE	1991	elastase, neutrophil expressed	ELA2|GE|HLE|HNE|NE|PMN-E	19	Antimicrobials
DEFB103B	414325	defensin, beta 103B	-	8	Antimicrobials
DEFA3	1668	defensin, alpha 3, neutrophil-specific	DEF3|HNP-3|HNP3|HP-3	8	Antimicrobials
DEFA1	1667	defensin, alpha 1	DEF1|DEFA2|HNP-1|HP-1|MGC138393|MRS	8	Antimicrobials
TMSB10	9168	thymosin beta 10	MIG12|TB10	2	Antimicrobials
DEFA6	1671	defensin, alpha 6, Paneth cell-specific	DEF6|HD-6	8	Antimicrobials
DEFA5	1670	defensin, alpha 5, Paneth cell-	DEF5|HD-5|MGC129728	8	Antimicrobials

		specific			
DEFA4	1669	defensin, alpha 4, corticostatin	DEF4|HNP-4|HP-4|HP4|MGC120099|MGC138296	8	Antimicrobials
LCN2	3934	lipocalin 2	24p3|NGAL	9	Antimicrobials
LCN1	3933	lipocalin 1 (tear prealbumin)	MGC71975|PMFA|TP|VEGP	9	Antimicrobials
COLEC10	10584	collectin sub-family member 10 (C-type lectin)	CLL1|MGC118794|MGC118795	8	Antimicrobials
BPI	671	bactericidal/permeability-increasing protein	-	20	Antimicrobials
S100A9	6280	S100 calcium binding protein A9	60B8AG|CAGB|CFAG|CGLB|L1AG|LIAG|MAC387|MIF|MRP14|NIF|P14	1	Antimicrobials
S100A8	6279	S100 calcium binding protein A8	60B8AG|CAGA|CFAG|CGLA|CP-10|L1Ag|MA387|MIF|MRP8|NIF|P8	1	Antimicrobials
DCD	117159	dermcidin	AIDD|DCD-1|DSEP|HCAP|MGC71930|PIF	12	Antimicrobials
LCN6	158062	lipocalin 6	LCN5|UNQ643|hLcn5	9	Antimicrobials
S100A12	6283	S100 calcium binding protein A12	CAAF1|CAGC|CGRP|ENRAGE|MRP6|p6	1	Antimicrobials
HTN3	3347	histatin 3	HIS2|HTN2|HTN5	4	Antimicrobials
LCN8	138307	lipocalin 8	EP17|LCN5	9	Antimicrobials
LOC728358	728358	defensin, alpha 1	-	8	Antimicrobials
CCR10	2826	chemokine (C-C motif) receptor 10	GPR2	17	Antimicrobials
CELA1	1990	chymotrypsin-like elastase family, member 1	ELA1	12	Antimicrobials
DEFB106A	245909	defensin, beta 106A	BD-6|DEFB-	8	Antimicrobials

			6|DEFB106|MGC118938|MGC118939|MGC118940|MGC118941|MGC133011|MGC133012		
PENK	5179	proenkephalin	-	8	Antimicrobials
BPIL2	254240	bactericidal/permeability-increasing protein-like 2	-	22	Antimicrobials
MMP12	4321	matrix metallopeptidase 12 (macrophage elastase)	HME|MGC138506|MME	11	Antimicrobials
BPIL3	128859	bactericidal/permeability-increasing protein-like 3	LPLUNC6	20	Antimicrobials
LEAP2	116842	liver expressed antimicrobial peptide 2	LEAP-2	5	Antimicrobials
SFTPD	6441	surfactant protein D	COLEC7|PSP-D|SFTP4|SP-D	10	Antimicrobials
LCN9	392399	lipocalin 9	9230102I19Rik	9	Antimicrobials
BPIL1	80341	bactericidal/permeability-increasing protein-like 1	C20orf184|LPLUNC2|RYSR|dJ726C3.2	20	Antimicrobials
PTGDS	5730	prostaglandin D2 synthase 21kDa (brain)	LPGDS|PDS|PGD2|PGDS|PGDS2	9	Antimicrobials
TMSB4X	7114	thymosin beta 4, X-linked	FX|PTMB4|TB4X|TMSB4	X	Antimicrobials
PGLYRP1	8993	peptidoglycan recognition protein 1	MGC126894|MGC126896|PGLYRP|PGRP|PGRP-S|PGRPS|TAG7|TNFSF3L	19	Antimicrobials
ZC3HAV1	56829	zinc finger CCCH-type, antiviral 1	DKFZp686F2052|DKFZp686H1869|DKFZp686O19171|FLB6421|FLJ13288|MGC48898|ZAP|ZC3H2|ZC3HDC2	7	Antimicrobials
TMSB15A	11013	thymosin beta 15a	TMSB15|TMSL8|TMSNB|Tb15|TbNB	X	Antimicrobials
S100B	6285	S100 calcium binding protein B	NEF|S100|S100beta	21	Antimicrobials
S100A13	6284	S100 calcium binding protein	-	1	Antimicrobials

		A13			
S100A6	6277	S100 calcium binding protein A6	2A9|5B10|CABP|CACY|PRA	1	Antimicrobials
DEFB119	245932	defensin, beta 119	DEFB-19|DEFB-20|DEFB120|ESC42-RELA|ESC42-RELB|MGC71893	20	Antimicrobials
DEFB107A	245910	defensin, beta 107A	BD-7|DEFB-7|DEFB107	8	Antimicrobials
DEFB105A	245908	defensin, beta 105A	BD-5|DEFB-5|DEFB105	8	Antimicrobials
SERPIND1	3053	serpin peptidase inhibitor, clade D (heparin cofactor), member 1	D22S673|HC2|HCF2|HCII|HLS2|LS2	22	Antimicrobials
DEFB129	140881	defensin, beta 129	C20orf87|DEFB-29|DEFB29|bA530N10.3|hBD-29	20	Antimicrobials
DEFB127	140850	defensin, beta 127	C20orf73|DEF-27|DEFB-27|DEFB27|bA530N10.2|hBD-27	20	Antimicrobials
S100P	6286	S100 calcium binding protein P	MIG9	4	Antimicrobials
S100A7	6278	S100 calcium binding protein A7	PSOR1|S100A7c	1	Antimicrobials
DEFB104A	140596	defensin, beta 104A	BD-4|DEFB-4|DEFB104|DEFB4|MGC118942|MGC118944|MGC118945|hBD-4	8	Antimicrobials
DEFB126	81623	defensin, beta 126	C20orf8|DEFB-26|DEFB26|ESP13.2|bA530N10.1|hBD-26	20	Antimicrobials
DEFB106B	503841	defensin, beta 106B	-	8	Antimicrobials
DEFB104B	503618	defensin, beta 104B	-	8	Antimicrobials
DEFB107B	503614	defensin, beta 107B	HsT21816	8	Antimicrobials
PGLYRP3	114771	peptidoglycan recognition protein 3	MGC149197|PGRP-Ialpha|PGRPIA	1	Antimicrobials
PGLYRP2	114770	peptidoglycan recognition protein 2	HMFT0141|PGLYRPL|PGRP-L|PGRPL|TAGL-like|tagL|tagL-alpha|tagl-beta	19	Antimicrobials

S100A10	6281	S100 calcium binding protein A10	42C|ANX2L|ANX2LG|CAL1L|CLP11|Ca[1]|GP11 |MGC111133|P11|p10	1	Antimicrobials
S100A2	6273	S100 calcium binding protein A2	CAN19|MGC111539|S100L	1	Antimicrobials
DEFB125	245938	defensin, beta 125	DEFB-25|MGC57449	20	Antimicrobials
DEFB123	245936	defensin, beta 123	DEFB-23|ESC42-RELD	20	Antimicrobials
DEFB105B	504180	defensin, beta 105B	-	8	Antimicrobials
DEFB132	400830	defensin, beta 132	DEFB32|UNQ827	20	Antimicrobials
C20orf185	359710	chromosome 20 open reading frame 185	LPLUNC3|RYA3|dJ726C3.4	20	Antimicrobials
LCN12	286256	lipocalin 12	MGC34753|MGC48935	9	Antimicrobials
PGLYRP4	57115	peptidoglycan recognition protein 4	PGLYRPIbeta|PGRP-Ibeta|PGRPIB|SBBI67	1	Antimicrobials
S100A11	6282	S100 calcium binding protein A11	MLN70|S100C	1	Antimicrobials
S100A5	6276	S100 calcium binding protein A5	S100D	1	Antimicrobials
S100A3	6274	S100 calcium binding protein A3	S100E	1	Antimicrobials
S100A1	6271	S100 calcium binding protein A1	S100|S100-alpha|S100A	1	Antimicrobials
DEFB128	245939	defensin, beta 128	DEFB-28|DEFB28|hBD-28	20	Antimicrobials
DEFB108B	245911	defensin, beta 108B	-	11	Antimicrobials
HTN1	3346	histatin 1	HIS1	4	Antimicrobials
LMBR1L	55716	limb region 1 homolog (mouse)-like	FLJ10494|FLJ36251|KIAA1174|LIMR	12	Antimicrobials
S100A7A	338324	S100 calcium binding protein A7A	NICE-2|S100A15|S100A7L1|S100A7f	1	Antimicrobials
DEFB118	117285	defensin, beta 118	C20orf63|DEFB-18|ESC42|dJ1018D12.3	20	Antimicrobials
COLEC12	81035	collectin sub-family member 12	CLP1|NSR2|SCARA4|SRCL	18	Antimicrobials

TMSB4Y	9087	thymosin beta 4, Y-linked	MGC26307|TB4Y	Y	Antimicrobials
DEFB131	644414	defensin, beta 131	DEFB-31	4	Antimicrobials
DEFB134	613211	defensin, beta 134	MGC163333|MGC163335	8	Antimicrobials
DEFB130	245940	defensin, beta 130	DEFB-30	8	Antimicrobials
DEFB124	245937	defensin, beta 124	DEFB-24	20	Antimicrobials
DEFB121	245934	defensin, beta 121	DEFB-21|ESC42-RELC	20	Antimicrobials
DEFB116	245930	defensin, beta 116	DEFB-16	20	Antimicrobials
DEFB115	245929	defensin, beta 115	DEFB-15	20	Antimicrobials
DEFB114	245928	defensin, beta 114	DEFB-14	6	Antimicrobials
DEFB113	245927	defensin, beta 113	DEFB-13	6	Antimicrobials
DEFB112	245915	defensin, beta 112	DEFB-12	6	Antimicrobials
DEFB110	245913	defensin, beta 110	DEFB-10	6	Antimicrobials
TMSB15B	286527	thymosin beta 15B	MGC39900|Tbeta15b	X	Antimicrobials
DEFB133	403339	defensin, beta 133	-	12	Antimicrobials
S100Z	170591	S100 calcium binding protein Z	Gm625|S100-zeta	5	Antimicrobials
MAVS	57506	mitochondrial antiviral signaling protein	CARDIF|DKFZp547C224|DKFZp666M015|FLJ27482|FLJ35386|FLJ38051|FLJ41962|IPS-1|IPS1|KIAA1271|MGC3260|VISA	20	Antimicrobials
TMSL3	7117	thymosin-like 3	-	4	Antimicrobials
S100A14	57402	S100 calcium binding protein A14	BCMP84|S100A15	1	Antimicrobials
LCN10	414332	lipocalin 10	-	9	Antimicrobials
S100A16	140576	S100 calcium binding protein A16	AAG13|DT1P1A7|MGC17528|S100F	1	Antimicrobials
DEFB137	613210	beta-defensin 137	DEFB136	8	Antimicrobials
DEFB136	613209	beta-defensin 136	DEFB135	8	Antimicrobials

DEFB117	245931	defensin, beta 117	DEFB-17	20	Antimicrobials
DEFB111	245914	defensin, beta 111	DEFB-11	6	Antimicrobials
ZC3HAV1L	92092	zinc finger CCCH-type, antiviral 1-like	C7orf39|MGC14289	7	Antimicrobials
S100A7L2	645922	S100 calcium binding protein A7-like 2	S100a7b	1	Antimicrobials
LOC731414	731414	similar to S100 calcium-binding protein A10	-	17	Antimicrobials
LOC730963	730963	similar to Neutrophil defensin 4 precursor (HNP-4) (HP-4) (Defensin, alpha 4)	-	8	Antimicrobials
COLEC2	50639	collectin sub-family member 2	MBL	-	Antimicrobials
DEFB4P	728454	defensin, beta 4, pseudogene	-	8	Antimicrobials
C20orf186	149954	chromosome 20 open reading frame 186	LPLUNC4|RY2G5|dJ726C3.5	20	Antimicrobials
IFNAR1	3454	interferon (alpha, beta and omega) receptor 1	AVP|IFN-alpha-REC|IFNAR|IFNBR|IFRC	21	Antimicrobials
AZU1	566	azurocidin 1	AZAMP|AZU|CAP37|HBP|HUMAZUR|NAZC	19	Antimicrobials
LOC729523	729523	similar to beta-defensin 130	-	11	Antimicrobials
LOC100130154	1E+08	similar to thymosin, beta 10	-	2	Antimicrobials
LOC100134379	1E+08	similar to bactericidal/permeability-increasing protein	-	Un	Antimicrobials
LOC10013	1E+08	similar to bactericidal	-	20	Antimicrobials

4289		permeability increasing protein (BPI)			
LOC100129216	1E+08	similar to beta-defensin 131	-	11	Antimicrobials
DEFA1A3	613253	defensin, alpha 1 and alpha 3, variable copy number locus	DEFA1|DEFA3|DEFT1P	8	Antimicrobials
LOC100131433	1E+08	similar to thymosin, beta 10	-	9	Antimicrobials
LCN1L1	286310	lipocalin 1-like 1	bA430N14.2	9	Antimicrobials
S100G	795	S100 calcium binding protein G	CABP1|CABP9K|CALB3|MGC138379	X	Antimicrobials
LOC648637	648637	similar to Neutrophil defensin 4 precursor (HNP-4) (HP-4) (Defensin, alpha 4)	-	8	Antimicrobials
LOC100130969	1E+08	similar to lipocalin-like protein	-	9	Antimicrobials
LOC100133267	1E+08	similar to beta-defensin 130	-	8	Antimicrobials
LOC100133128	1E+08	Beta-defensin 108B-like	-	4	Antimicrobials
LOC100128174	1E+08	similar to beta-defensin 131	-	8	Antimicrobials
TCHHL1	126637	trichohyalin-like 1	S100A17|THHL1|basalin	1	Antimicrobials
TINAGL1	64129	tubulointerstitial nephritis antigen-like 1	ARG1|LCN7|LIECG3|TINAGRP	1	Antimicrobials
IFNGR1	3459	interferon gamma receptor 1	CD119|FLJ45734|IFNGR	6	Antimicrobials
SLC22A17	51310	solute carrier family 22, member	BOCT|BOIT|NGALR|hBOIT	14	Antimicrobials

		17			
WFIKKN1	117166	WAP, follistatin/kazal, immunoglobulin, kunitz and netrin domain containing 1	C16orf12|MGC126651|MGC126655|RJD2|WFIKKN	16	Antimicrobials
WFDC2	10406	WAP four-disulfide core domain 2	HE4|MGC57529|WAP5|dJ461P17.6	20	Antimicrobials
IL6	3569	interleukin 6 (interferon, beta 2)	BSF2|HGF|HSF|IFNB2|IL-6	7	Antimicrobials
UMODL1	89766	uromodulin-like 1	-	21	Antimicrobials
TGFB1	7040	transforming growth factor, beta 1	CED|DPD1|TGFB|TGFbeta	19	Antimicrobials
PF4V1	5197	platelet factor 4 variant 1	CXCL4L1|CXCL4V1|PF4-ALT|PF4A|SCYB4V1	4	Antimicrobials
MMP9	4318	matrix metallopeptidase 9 (gelatinase B, 92kDa gelatinase, 92kDa type IV collagenase)	CLG4B|GELB|MMP-9	20	Antimicrobials
KAL1	3730	Kallmann syndrome 1 sequence	ADMLX|HHA|KAL|KALIG-1|KMS	X	Antimicrobials
TLR4	7099	toll-like receptor 4	ARMD10|CD284|TOLL|hToll	9	Antimicrobials
IFNG	3458	interferon, gamma	IFG|IFI	12	Antimicrobials
SPAG11B	10407	sperm associated antigen 11B	EP2|EP2C|EP2D|HE2|HE2C|MGC61846|SPAG11	8	Antimicrobials
A2M	2	alpha-2-macroglobulin	CPAMD5|DKFZp779B086|FWP007|S863-7	12	Antimicrobials
CTSL1	1514	cathepsin L1	CATL|CTSL|FLJ31037|MEP	9	Antimicrobials
NFKB1	4790	nuclear factor of kappa light polypeptide gene enhancer in B-cells 1	DKFZp686C01211|EBP-1|KBF1|MGC54151|NF-kappa-B|NFKB-p105|NFKB-p50|p105|p50	4	Antimicrobials
APOBEC3G	60489	apolipoprotein B mRNA editing enzyme, catalytic polypeptide-	ARP9|CEM15|FLJ12740|MDS019|bK150C2.7|dJ494G10.1	22	Antimicrobials

		like 3G			
FABP6	2172	fatty acid binding protein 6, ileal	I-15P|I-BABP|I-BALB|I-BAP|ILBP|ILBP3|ILLBP	5	Antimicrobials
NOD2	64127	nucleotide-binding oligomerization domain containing 2	ACUG|BLAU|CARD15|CD|CLR16.3|IBD1|NLRC2|NOD2B|PSORAS1	16	Antimicrobials
MBL2	4153	mannose-binding lectin (protein C) 2, soluble (opsonic defect)	COLEC1|HSMBPC|MBL|MBP|MBP1|MGC116832|MGC116833	10	Antimicrobials
SFTPA1B	6435	surfactant protein A1B	AC068139.6|MGC133365|PSAP|PSPA|SFTP1|SFTPA1	10	Antimicrobials
RBP1	5947	retinol binding protein 1, cellular	CRABP-I|CRBP|CRBP1|CRBPI|RBPC	3	Antimicrobials
TLR2	7097	toll-like receptor 2	CD282|TIL4	4	Antimicrobials
SLC40A1	30061	solute carrier family 40 (iron-regulated transporter), member 1	FPN1|HFE4|IREG1|MST079|MSTP079|MTP1|SLC11A3	2	Antimicrobials
PLAU	5328	plasminogen activator, urokinase	ATF|UPA|URK|u-PA	10	Antimicrobials
IL1B	3553	interleukin 1, beta	IL-1|IL1-BETA|IL1F2	2	Antimicrobials
PAEP	5047	progestagen-associated endometrial protein	GD|GdA|GdF|GdS|MGC138509|MGC142288|PAEG|PEP|PP14	9	Antimicrobials
HFE2	148738	hemochromatosis type 2 (juvenile)	HFE2A|HJV|JH|MGC23953|RGMC	1	Antimicrobials
MUC5AC	4586	mucin 5AC, oligomeric mucus/gel-forming	MUC5	11	Antimicrobials
CTSS	1520	cathepsin S	MGC3886	1	Antimicrobials
OBP2A	29991	odorant binding protein 2A	OBP|OBP2C|OBPIIa|hOBPIIa	9	Antimicrobials
PLTP	5360	phospholipid transfer protein	HDLCQ9	20	Antimicrobials
MX1	4599	myxovirus (influenza virus) resistance 1, interferon-inducible	IFI-78K|IFI78|MX|MxA	21	Antimicrobials

		protein p78 (mouse)			
DDX58	23586	DEAD (Asp-Glu-Ala-Asp) box polypeptide 58	DKFZp434J1111|DKFZp686N19181|FLJ13599|RIG-I	9	Antimicrobials
IL29	282618	interleukin 29 (interferon, lambda 1)	IFNL1|IL-29	19	Antimicrobials
IRF3	3661	interferon regulatory factor 3	-	19	Antimicrobials
SFTPA2	729238	surfactant protein A2	COLEC5|MGC189761|SP-A2|SPA2|SPAII	10	Antimicrobials
SFTPA2B	6436	surfactant protein A2B	AC068139.3|SFTPA2|SP-2A|SP-A1|SP-A2|SPAII	10	Antimicrobials
LPA	4018	lipoprotein, Lp(a)	AK38|APOA|LP	6	Antimicrobials
LBP	3929	lipopolysaccharide binding protein	MGC22233	20	Antimicrobials
RBP4	5950	retinol binding protein 4, plasma	-	10	Antimicrobials
SFTPA1	653509	surfactant protein A1	COLEC4|FLJ51913|SFTP1|SP-A|SP-A1	10	Antimicrobials
NOX4	50507	NADPH oxidase 4	KOX|KOX-1|RENOX	11	Antimicrobials
LTF	4057	lactotransferrin	GIG12|HLF2|LF	3	Antimicrobials
IFNB1	3456	interferon, beta 1, fibroblast	IFB|IFF|IFNB|MGC96956	9	Antimicrobials
RBP5	83758	retinol binding protein 5, cellular	CRBP-III|CRBP3|CRBPIII	12	Antimicrobials
FABP7	2173	fatty acid binding protein 7, brain	B-FABP|BLBP|DKFZp547J2313|FABPB|MRG	6	Antimicrobials
FABP5	2171	fatty acid binding protein 5 (psoriasis-associated)	E-FABP|EFABP|PA-FABP|PAFABP	8	Antimicrobials
FABP3	2170	fatty acid binding protein 3, muscle and heart (mammary-derived growth inhibitor)	FABP11|H-FABP|MDGI|O-FABP	1	Antimicrobials
FABP2	2169	fatty acid binding protein 2, intestinal	FABPI|I-FABP|MGC133132	4	Antimicrobials

FABP4	2167	fatty acid binding protein 4, adipocyte	A-FABP|aP2	8	Antimicrobials
R3HDML	140902	R3H domain containing-like	MGC129564|dJ881L22.3	20	Antimicrobials
C20orf71	128861	chromosome 20 open reading frame 71	MGC44525|SPLUNC3	20	Antimicrobials
C20orf114	92747	chromosome 20 open reading frame 114	LPLUNC1|MGC14597	20	Antimicrobials
OASL	8638	2'-5'-oligoadenylate synthetase-like	TRIP14|p59OASL	12	Antimicrobials
CRABP2	1382	cellular retinoic acid binding protein 2	CRABP-II|RBP6	1	Antimicrobials
CRABP1	1381	cellular retinoic acid binding protein 1	CRABP|CRABP-I|CRABPI|RBP5	15	Antimicrobials
RBP7	116362	retinol binding protein 7, cellular	CRBP4|CRBPIV|MGC70641	1	Antimicrobials
DUOX1	53905	dual oxidase 1	LNOX1|MGC138840|MGC138841|NOXEF1|THOX1	15	Antimicrobials
OBP2B	29989	odorant binding protein 2B	MGC119022|hOBPIIb	9	Antimicrobials
RBP2	5948	retinol binding protein 2, cellular	CRABP-II|CRBP2|CRBPII|RBPC2	3	Antimicrobials
LCN15	389812	lipocalin 15	PRO6093|UNQ2541	9	Antimicrobials
CETP	1071	cholesteryl ester transfer protein, plasma	HDLCQ10	16	Antimicrobials
FABP12	646486	fatty acid binding protein 12	-	8	Antimicrobials
FABP9	646480	fatty acid binding protein 9, testis	PERF|PERF15|T-FABP	8	Antimicrobials
PLUNC	51297	palate, lung and nasal epithelium associated	LPLUNC3|LUNX|NASG|SPLUNC1|SPURT|bA49G10.5	20	Antimicrobials
LCNL1	401562	lipocalin-like 1	FLJ45224	9	Antimicrobials

C8G	733	complement component 8, gamma polypeptide	C8C|MGC142186	9	Antimicrobials
SPAG11A	653423	sperm associated antigen 11A	HE2	8	Antimicrobials
PI15	51050	peptidase inhibitor 15	CRISP8|DKFZp686F0366|P24TI|P25TI	8	Antimicrobials
NOX1	27035	NADPH oxidase 1	GP91-2|MOX1|NOH-1|NOH1	X	Antimicrobials
PMP2	5375	peripheral myelin protein 2	FABP8|M-FABP|MP2|P2	8	Antimicrobials
APOD	347	apolipoprotein D	-	3	Antimicrobials
ORM2	5005	orosomucoid 2	AGP-B|AGP-B'|AGP2	9	Antimicrobials
ORM1	5004	orosomucoid 1	AGP-A|AGP1|ORM	9	Antimicrobials
TNF	7124	tumor necrosis factor (TNF superfamily, member 2)	DIF|TNF-alpha|TNFA|TNFSF2	6	Antimicrobials
CTSG	1511	cathepsin G	CG|MGC23078	14	Antimicrobials
PRTN3	5657	proteinase 3	ACPA|AGP7|C-ANCA|MBT|P29|PR-3	19	Antimicrobials
MAPK1	5594	mitogen-activated protein kinase 1	ERK|ERK2|ERT1|MAPK2|P42MAPK|PRKM1|PRKM2|p38|p40|p41|p41mapk	22	Antimicrobials
PML	5371	promyelocytic leukemia	MYL|PP8675|RNF71|TRIM19	15	Antimicrobials
AEN	64782	apoptosis enhancing nuclease	FLJ12484|FLJ12562|ISG20L1|pp12744	15	Antimicrobials
CYBB	1536	cytochrome b-245, beta polypeptide	CGD|GP91-1|GP91-PHOX|GP91PHOX|NOX2|p91-PHOX	X	Antimicrobials
C20orf70	140683	chromosome 20 open reading frame 70	PSP|SPLUNC2|bA49G10.1	20	Antimicrobials
ISG20	3669	interferon stimulated exonuclease gene 20kDa	CD25|HEM45	15	Antimicrobials
BCL3	602	B-cell CLL/lymphoma 3	BCL4|D19S37	19	Antimicrobials
ISG20L2	81875	interferon stimulated exonuclease gene 20kDa-like 2	FLJ12671	1	Antimicrobials

NOX5	79400	NADPH oxidase, EF-hand calcium binding domain 5	MGC149776|MGC149777|NOX5A|NOX5B	15	Antimicrobials
NOX3	50508	NADPH oxidase 3	GP91-3	6	Antimicrobials
DUOX2	50506	dual oxidase 2	LNOX2|NOXEF2|P138-TOX|THOX2	15	Antimicrobials
TLR3	7098	toll-like receptor 3	CD283	4	Antimicrobials
TFRC	7037	transferrin receptor (p90, CD71)	CD71|TFR|TFR1|TRFR	3	Antimicrobials
IFIH1	64135	interferon induced with helicase C domain 1	Hlcd|IDDM19|MDA-5|MDA5|MGC133047	2	Antimicrobials
LRP1	4035	low density lipoprotein-related protein 1 (alpha-2-macroglobulin receptor)	A2MR|APOER|APR|CD91|FLJ16451|IGFBP3R|LRP|MGC88725|TGFBR5	12	Antimicrobials
TRIM5	85363	tripartite motif-containing 5	RNF88|TRIM5alpha	11	Antimicrobials
IDO1	3620	indoleamine 2,3-dioxygenase 1	CD107B|IDO|INDO	8	Antimicrobials
GDF15	9518	growth differentiation factor 15	GDF-15|MIC-1|MIC1|NAG-1|PDF|PLAB|PTGFB	19	Antimicrobials
NEDD4	4734	neural precursor cell expressed, developmentally down-regulated 4	KIAA0093|MGC176705|NEDD4-1|RPF1	15	Antimicrobials
ADIPOQ	9370	adiponectin, C1Q and collagen domain containing	ACDC|ACRP30|ADIPQTL1|ADPN|APM-1|APM1|GBP28|adiponectin	3	Antimicrobials
STAT3	6774	signal transducer and activator of transcription 3 (acute-phase response factor)	APRF|FLJ20882|HIES|MGC16063	17	Antimicrobials
STAT1	6772	signal transducer and activator of transcription 1, 91kDa	DKFZp686B04100|ISGF-3|STAT91	2	Antimicrobials
IL28A	282616	interleukin 28A (interferon, lambda 2)	IFNL2|IL-28A	19	Antimicrobials

SOCS3	9021	suppressor of cytokine signaling 3	ATOD4|CIS3|Cish3|MGC71791|SOCS-3|SSI-3|SSI3	17	Antimicrobials
SEMG1	6406	semenogelin I	MGC14719|SEMG|SGI	20	Antimicrobials
TNFSF10	8743	tumor necrosis factor (ligand) superfamily, member 10	APO2L|Apo-2L|CD253|TL2|TRAIL	3	Antimicrobials
CCL20	6364	chemokine (C-C motif) ligand 20	CKb4|LARC|MIP-3a|MIP3A|SCYA20|ST38	2	Antimicrobials
SOCS1	8651	suppressor of cytokine signaling 1	CIS1|CISH1|JAB|SOCS-1|SSI-1|SSI1|TIP3	16	Antimicrobials
RNASEL	6041	ribonuclease L (2',5'-oligoisoadenylate synthetase-dependent)	DKFZp781D08126|MGC104972|MGC133329|PRCA1|RNS4	1	Antimicrobials
IRF1	3659	interferon regulatory factor 1	IRF-1|MAR	5	Antimicrobials
IL15	3600	interleukin 15	IL-15|MGC9721	4	Antimicrobials
APOBEC3F	200316	apolipoprotein B mRNA editing enzyme, catalytic polypeptide-like 3F	ARP8|BK150C2.4.MRNA|KA6|MGC74891	22	Antimicrobials
RARRES3	5920	retinoic acid receptor responder (tazarotene induced) 3	HRASLS4|MGC8906|PLA1/2-3|RIG1|TIG3	11	Antimicrobials
CHIT1	1118	chitinase 1 (chitotriosidase)	CHI3|CHIT|FLJ00314|MGC125322	1	Antimicrobials
IFNA1	3439	interferon, alpha 1	IFL|IFN|IFN-ALPHA|IFNA13|IFNA@|MGC138207|MGC138505|MGC138507	9	Antimicrobials
CD40	958	CD40 molecule, TNF receptor superfamily member 5	Bp50|CDW40|MGC9013|TNFRSF5|p50	20	Antimicrobials
TLR7	51284	toll-like receptor 7	-	X	Antimicrobials
PPIA	5478	peptidylprolyl isomerase A	CYPA|CYPH|MGC117158|MGC12404|MGC233	7	Antimicrobials

		(cyclophilin A)	97		
HFE	3077	hemochromatosis	HFE1|HH|HLA-H|MGC103790|MVCD7|dJ221C16.10.1	6	Antimicrobials
ZYX	7791	zyxin	ESP-2|HED-2	7	Antimicrobials
NLRX1	79671	NLR family member X1	CLR11.3|DLNB26|FLJ21478|MGC131937|MGC21025|NOD26|NOD5|NOD9	11	Antimicrobials
PGC	5225	progastricsin (pepsinogen C)	-	6	Antimicrobials
VEGFA	7422	vascular endothelial growth factor A	MGC70609|MVCD1|VEGF|VEGF-A|VPF	6	Antimicrobials
IKBKE	9641	inhibitor of kappa light polypeptide gene enhancer in B-cells, kinase epsilon	IKK-i|IKKE|IKKI|KIAA0151|MGC125294|MGC125295|MGC125297	1	Antimicrobials
ISG15	9636	ISG15 ubiquitin-like modifier	G1P2|IFI15|UCRP	1	Antimicrobials
DHX58	79132	DEXH (Asp-Glu-X-His) box polypeptide 58	D11LGP2|D11lgp2e|LGP2	17	Antimicrobials
TNFAIP3	7128	tumor necrosis factor, alpha-induced protein 3	A20|MGC104522|MGC138687|MGC138688|OTUD7C|TNFA1P2	6	Antimicrobials
TFR2	7036	transferrin receptor 2	HFE3|MGC126368|TFRC2	7	Antimicrobials
FCN2	2220	ficolin (collagen/fibrinogen domain containing lectin) 2 (hucolin)	EBP-37|FCNL|P35|ficolin-2	9	Antimicrobials
MUC4	4585	mucin 4, cell surface associated	HSA276359	3	Antimicrobials
F2R	2149	coagulation factor II (thrombin) receptor	CF2R|HTR|PAR1|TR	5	Antimicrobials
ELN	2006	elastin	FLJ38671|FLJ43523|SVAS|WBS|WS	7	Antimicrobials
IL27	246778	interleukin 27	IL-27|IL-27A|IL27p28|IL30|MGC71873|p28	16	Antimicrobials

MAPT	4137	microtubule-associated protein tau	DDPAC|FLJ31424|FTDP-17|MAPTL|MGC138549|MSTD|MTBT1|MTBT2|PPND|TAU	17	Antimicrobials
LYZ	4069	lysozyme (renal amyloidosis)	LZM|lysozyme	12	Antimicrobials
CCL5	6352	chemokine (C-C motif) ligand 5	D17S136E|MGC17164|RANTES|SCYA5|SISd|TCP228	17	Antimicrobials
LEP	3952	leptin	FLJ94114|OB|OBS	7	Antimicrobials
CYLD	1540	cylindromatosis (turban tumor syndrome)	CDMT|CYLD1|CYLDI|EAC|FLJ20180|FLJ31664|FLJ78684|HSPC057|KIAA0849|MFT|MFT1|SBS|TEM|USPL2	16	Antimicrobials
KLKB1	3818	kallikrein B, plasma (Fletcher factor) 1	KLK3|PPK	4	Antimicrobials
CST4	1472	cystatin S	MGC71923	20	Antimicrobials
CSRP1	1465	cysteine and glycine-rich protein 1	CRP|CRP1|CSRP|CYRP|D1S181E|DKFZp686M148	1	Antimicrobials
MAPK14	1432	mitogen-activated protein kinase 14	CSBP1|CSBP2|CSPB1|EXIP|Mxi2|PRKM14|PRKM15|RK|SAPK2A|p38|p38ALPHA	6	Antimicrobials
JUN	3725	jun oncogene	AP-1|AP1|c-Jun	1	Antimicrobials
ITGAV	3685	integrin, alpha V (vitronectin receptor, alpha polypeptide, antigen CD51)	CD51|DKFZp686A08142|MSK8|VNRA	2	Antimicrobials
IRF5	3663	interferon regulatory factor 5	SLEB10	7	Antimicrobials
CCR6	1235	chemokine (C-C motif) receptor 6	BN-1|CD196|CKR-L3|CKR6|CKRL3|CMKBR6|DCR2|DRY-6|GPR-CY4|GPR29|GPRCY4|STRL22	6	Antimicrobials
IL12B	3593	interleukin 12B (natural killer cell	CLMF|CLMF2|IL-12B|NKSF|NKSF2	5	Antimicrobials

		stimulatory factor 2, cytotoxic lymphocyte maturation factor 2, p40)			
TLR8	51311	toll-like receptor 8	CD288|MGC119599|MGC119600	X	Antimicrobials
GNLY	10578	granulysin	519|D2S69E|LAG-2|LAG2|NKG5|TLA519	2	Antimicrobials
CD81	975	CD81 molecule	S5.7|TAPA1|TSPAN28	11	Antimicrobials
EIF2AK2	5610	eukaryotic translation initiation factor 2-alpha kinase 2	EIF2AK1|MGC126524|PKR|PRKR	2	Antimicrobials
APOM	55937	apolipoprotein M	G3a|HSPC336|MGC22400|NG20	6	Antimicrobials
CACYBP	27101	calcyclin binding protein	GIG5|MGC87971|PNAS-107|RP1-102G20.6|S100A6BP|SIP	1	Antimicrobials
NOD1	10392	nucleotide-binding oligomerization domain containing 1	CARD4|CLR7.1|NLRC1	7	Antimicrobials
MAPK8	5599	mitogen-activated protein kinase 8	JNK|JNK1|JNK1A2|JNK21B1/2|PRKM8|SAPK1	10	Antimicrobials
MAPK3	5595	mitogen-activated protein kinase 3	ERK1|HS44KDAP|HUMKER1A|MGC20180|P44ERK1|P44MAPK|PRKM3	16	Antimicrobials
BST2	684	bone marrow stromal cell antigen 2	CD317	19	Antimicrobials
BPHL	670	biphenyl hydrolase-like (serine hydrolase)	BPH-RP|MCNAA|MGC125930|MGC41865|VACVASE	6	Antimicrobials
PLA2G2A	5320	phospholipase A2, group IIA (platelets, synovial fluid)	MOM1|PLA2|PLA2B|PLA2L|PLA2S|PLAS1|sPLA2	1	Antimicrobials
GRN	2896	granulin	GEP|GP88|PCDGF|PEPI|PGRN	17	Antimicrobials
NEWENTR	192343	Record to support submission of	-	-	Antimicrobials

Y		GeneRIFs for a gene not in Entrez Gene (human; man).			
PDGFRA	5156	platelet-derived growth factor receptor, alpha polypeptide	CD140A|MGC74795|PDGFR2|Rhe-PDGFRA	4	Antimicrobials
GNAI1	2770	guanine nucleotide binding protein (G protein), alpha inhibiting activity polypeptide 1	Gi	7	Antimicrobials
WNT5A	7474	wingless-type MMTV integration site family, member 5A	hWNT5A	3	Antimicrobials
FURIN	5045	furin (paired basic amino acid cleaving enzyme)	FUR|PACE|PCSK3|SPC1	15	Antimicrobials
ADAR	103	adenosine deaminase, RNA-specific	ADAR1|DRADA|DSH|DSRAD|G1P1|IFI-4|IFI4|K88dsRBP|p136	1	Antimicrobials
TYK2	7297	tyrosine kinase 2	JTK1	19	Antimicrobials
NOS2	4843	nitric oxide synthase 2, inducible	HEP-NOS|INOS|NOS|NOS2A	17	Antimicrobials
TRAF3	7187	TNF receptor-associated factor 3	CAP-1|CD40bp|CRAF1|LAP1	14	Antimicrobials
TPT1	7178	tumor protein, translationally-controlled 1	FLJ27337|HRF|TCTP|p02	13	Antimicrobials
TPM2	7169	tropomyosin 2 (beta)	AMCD1|DA1|DA2B|TMSB	9	Antimicrobials
NEO1	4756	neogenin homolog 1 (chicken)	DKFZp547A066|DKFZp547B146|HsT17534|IGDCC2|NGN	15	Antimicrobials
AHNAK	79026	AHNAK nucleoprotein	AHNAKRS|MGC5395	11	Antimicrobials
TLR1	7096	toll-like receptor 1	CD281|DKFZp547I0610|DKFZp564I0682|KIAA0012|MGC104956|MGC126311|MGC126312|TIL|rsc786	4	Antimicrobials
TK2	7084	thymidine kinase 2,	-	16	Antimicrobials

		mitochondrial			
PRDX2	7001	peroxiredoxin 2	MGC4104|NKEFB|PRP|PRX2|PRXII|TDPX1|TSA	19	Antimicrobials
MX2	4600	myxovirus (influenza virus) resistance 2 (mouse)	MXB	21	Antimicrobials
FGF2	2247	fibroblast growth factor 2 (basic)	BFGF|FGFB|HBGF-2	4	Antimicrobials
FGA	2243	fibrinogen alpha chain	Fib2|MGC119422|MGC119423|MGC119425	4	Antimicrobials
TCF7L2	6934	transcription factor 7-like 2 (T-cell specific, HMG-box)	TCF-4|TCF4	10	Antimicrobials
F2RL1	2150	coagulation factor II (thrombin) receptor-like 1	GPR11|PAR2	5	Antimicrobials
DAK	26007	dihydroxyacetone kinase 2 homolog (S. cerevisiae)	DKFZp586B1621|MGC5621	11	Antimicrobials
MSR1	4481	macrophage scavenger receptor 1	CD204|SCARA1|SR-A|phSR1|phSR2	8	Antimicrobials
NFKBIZ	64332	nuclear factor of kappa light polypeptide gene enhancer in B-cells inhibitor, zeta	FLJ30225|FLJ34463|IKBZ|INAP|MAIL	3	Antimicrobials
LMBR1	64327	limb region 1 homolog (mouse)	ACHP|C7orf2|DIF14|FLJ11665|PPD2|TPT	7	Antimicrobials
SPINLW1	57119	serine peptidase inhibitor-like, with Kunitz and WAP domains 1 (eppin)	EPPIN|EPPIN1|EPPIN2|EPPIN3|WAP7|WFDC7|dJ461P17.2	20	Antimicrobials
SRC	6714	v-Src sarcoma (Schmidt-Ruppin A-2) viral oncogene homolog (avian)	ASV|SRC1|c-SRC|p60-Src	20	Antimicrobials
MPO	4353	myeloperoxidase	-	17	Antimicrobials

ELAVL1	1994	ELAV (embryonic lethal, abnormal vision, Drosophila)-like 1 (Hu antigen R)	ELAV1|HUR|Hua|MelG	19	Antimicrobials
ROBO3	64221	roundabout, axon guidance receptor, homolog 3 (Drosophila)	FLJ21044|HGPPS|HGPS|RBIG1|RIG1	11	Antimicrobials
SP1	6667	Sp1 transcription factor	-	12	Antimicrobials
SOD1	6647	superoxide dismutase 1, soluble	ALS|ALS1|IPOA|SOD|homodimer	21	Antimicrobials
PDF	64146	peptide deformylase (mitochondrial)	-	16	Antimicrobials
DLL4	54567	delta-like 4 (Drosophila)	MGC126344|hdelta2	15	Antimicrobials
ECD	11319	ecdysoneless homolog (Drosophila)	GCR2|HSGT1	10	Antimicrobials
SLC11A1	6556	solute carrier family 11 (proton-coupled divalent metal ion transporters), member 1	LSH|NRAMP|NRAMP1	2	Antimicrobials
DMBT1	1755	deleted in malignant brain tumors 1	GP340|MGC164738|muclin	10	Antimicrobials
TMEM173	340061	transmembrane protein 173	FLJ38577|MITA|MPYS|STING	5	Antimicrobials
SKIV2L	6499	superkiller viralicidic activity 2-like (S. cerevisiae)	170A|DDX13|HLP|SKI2|SKI2W|SKIV2	6	Antimicrobials
SEMG2	6407	semenogelin II	SGII	20	Antimicrobials
LTA	4049	lymphotoxin alpha (TNF superfamily, member 1)	LT|TNFB|TNFSF1	6	Antimicrobials
DES	1674	desmin	CMD1I|CSM1|CSM2|FLJ12025|FLJ39719|FLJ41013|FLJ41793	2	Antimicrobials
DCK	1633	deoxycytidine kinase	MGC117410|MGC138632	4	Antimicrobials

DAXX	1616	death-domain associated protein	BING2|DAP6|EAP1|MGC126245|MGC126246	6	Antimicrobials
TNFRSF10A	8797	tumor necrosis factor receptor superfamily, member 10a	APO2|CD261|DR4|MGC9365|TRAILR-1|TRAILR1	8	Antimicrobials
TNFRSF10B	8795	tumor necrosis factor receptor superfamily, member 10b	CD262|DR5|KILLER|KILLER/DR5|TRAIL-R2|TRAILR2|TRICK2|TRICK2A|TRICK2B|TRICKB|ZTNFR9	8	Antimicrobials
EED	8726	embryonic ectoderm development	HEED|WAIT1	11	Antimicrobials
CCL4	6351	chemokine (C-C motif) ligand 4	ACT2|AT744.1|G-26|LAG1|MGC104418|MGC126025|MGC126026|MIP-1-beta|MIP1B|MIP1B1|SCYA2|SCYA4	17	Antimicrobials
LIMS1	3987	LIM and senescent cell antigen-like domains 1	PINCH|PINCH1	2	Antimicrobials
LALBA	3906	lactalbumin, alpha-	MGC138521|MGC138523	12	Antimicrobials
APOBEC3H	164668	apolipoprotein B mRNA editing enzyme, catalytic polypeptide-like 3H	ARP10|dJ742C19.2	22	Antimicrobials
TMPRSS6	164656	transmembrane protease, serine 6	IRIDA	22	Antimicrobials
SPINK5	11005	serine peptidase inhibitor, Kazal type 5	DKFZp686K19184|FLJ21544|FLJ97536|FLJ97596|FLJ99794|LEKTI|LETKI|NETS|NS|VAKTI	5	Antimicrobials
MARCO	8685	macrophage receptor with collagenous structure	SCARA2	2	Antimicrobials
BECN1	8678	beclin 1, autophagy related	ATG6|VPS30|beclin1	17	Antimicrobials
TNFSF11	8600	tumor necrosis factor (ligand) superfamily, member 11	CD254|ODF|OPGL|OPTB2|RANKL|TRANCE|hRANKL2|sOdf	13	Antimicrobials

KNG1	3827	kininogen 1	BDK|KNG	3	Antimicrobials
CSK	1445	c-src tyrosine kinase	MGC117393	15	Antimicrobials
KLRK1	22914	killer cell lectin-like receptor subfamily K, member 1	CD314|D12S2489E|FLJ17759|FLJ75772|KLR|NKG2-D|NKG2D	12	Antimicrobials
KCNH2	3757	potassium voltage-gated channel, subfamily H (eag-related), member 2	ERG1|HERG|HERG1|Kv11.1|LQT2|SQT1	7	Antimicrobials
JUND	3727	jun D proto-oncogene	AP-1	19	Antimicrobials
JAK1	3716	Janus kinase 1	JAK1A|JAK1B|JTK3	1	Antimicrobials
CREB1	1385	cAMP responsive element binding protein 1	CREB|MGC9284	2	Antimicrobials
CLDN4	1364	claudin 4	CPE-R|CPER|CPETR|CPETR1|WBSCR8|hCPE-R	7	Antimicrobials
CCL28	56477	chemokine (C-C motif) ligand 28	CCK1|MEC|MGC71902|SCYA28	5	Antimicrobials
RNASE3	6037	ribonuclease, RNase A family, 3 (eosinophil cationic protein)	ECP|RNS3	14	Antimicrobials
RN7SL1	6029	RNA, 7SL, cytoplasmic 1	7L1a|7SL|RN7SL|RNSRP1	14	Antimicrobials
IRF7	3665	interferon regulatory factor 7	IRF-7H|IRF7A	11	Antimicrobials
IREB2	3658	iron-responsive element binding protein 2	ACO3|FLJ23381|IRP2|IRP2AD	15	Antimicrobials
ILK	3611	integrin-linked kinase	DKFZp686F1765|P59	11	Antimicrobials
IL18	3606	interleukin 18 (interferon-gamma-inducing factor)	IGIF|IL-18|IL-1g|IL1F4|MGC12320	11	Antimicrobials
IL17A	3605	interleukin 17A	CTLA8|IL-17|IL-17A|IL17	6	Antimicrobials
LTB4R	1241	leukotriene B4 receptor	BLT1|BLTR|CMKRL1|GPR16|LTB4R1|LTBR1|P2RY7|P2Y7	14	Antimicrobials

APOBEC3A	200315	apolipoprotein B mRNA editing enzyme, catalytic polypeptide-like 3A	ARP3|PHRBN|bK150C2.1	22	Antimicrobials
MASP2	10747	mannan-binding lectin serine peptidase 2	MAP19|MASP-2|sMAP	1	Antimicrobials
TRIM27	5987	tripartite motif-containing 27	RFP|RNF76	6	Antimicrobials
RELA	5970	v-rel reticuloendotheliosis viral oncogene homolog A (avian)	MGC131774|NFKB3|p65	11	Antimicrobials
IL7R	3575	interleukin 7 receptor	CD127|CDW127|IL-7R-alpha|IL7RA|ILRA	5	Antimicrobials
IL1A	3552	interleukin 1, alpha	IL-1A|IL1|IL1-ALPHA|IL1F1	2	Antimicrobials
PTX3	5806	pentraxin-related gene, rapidly induced by IL-1 beta	TNFAIP5|TSG-14	3	Antimicrobials
IFNAR2	3455	interferon (alpha, beta and omega) receptor 2	IFN-R|IFN-alpha-REC|IFNABR|IFNARB	21	Antimicrobials
IFN1@	3438	interferon, type 1, cluster	IFNA	9	Antimicrobials
SYTL1	84958	synaptotagmin-like 1	FLJ14996|JFC1|SLP1	1	Antimicrobials
APOBEC3C	27350	apolipoprotein B mRNA editing enzyme, catalytic polypeptide-like 3C	APOBEC1L|ARDC2|ARDC4|ARP5|MGC19485|PBI|bK150C2.3	22	Antimicrobials
DDX17	10521	DEAD (Asp-Glu-Ala-Asp) box polypeptide 17	DKFZp761H2016|P72|RH70	22	Antimicrobials
PTGS2	5743	prostaglandin-endoperoxide synthase 2 (prostaglandin G/H synthase and cyclooxygenase)	COX-2|COX2|GRIPGHS|PGG/HS|PGHS-2|PHS-2|hCox-2	1	Antimicrobials
HTR1A	3350	5-hydroxytryptamine (serotonin) receptor 1A	5-HT1A|5HT1a|ADRB2RL1|ADRBRL1	5	Antimicrobials

SEPT-7	989	septin 7	CDC10|CDC3|Nbla02942|SEPT7A	7	Antimicrobials
CD40LG	959	CD40 ligand	CD154|CD40L|HIGM1|IGM|IMD3|T-BAM|TNFSF5|TRAP|gp39|hCD40L	X	Antimicrobials
CD14	929	CD14 molecule	-	5	Antimicrobials
CD8A	925	CD8a molecule	CD8|Leu2|MAL|p32	2	Antimicrobials
CD4	920	CD4 molecule	CD4mut	12	Antimicrobials
MASP1	5648	mannan-binding lectin serine peptidase 1 (C4/C2 activating component of Ra-reactive factor)	CRARF|CRARF1|DKFZp686I01199|FLJ26383|MASP|MGC126283|MGC126284|PRSS5|RaRF	3	Antimicrobials
PROC	5624	protein C (inactivator of coagulation factors Va and VIIIa)	PC|PROC1	2	Antimicrobials
MAP2K2	5605	mitogen-activated protein kinase kinase 2	FLJ26075|MAPKK2|MEK2|MKK2|PRKMK2	19	Antimicrobials
MAP2K1	5604	mitogen-activated protein kinase kinase 1	MAPKK1|MEK1|MKK1|PRKMK1	15	Antimicrobials
HRG	3273	histidine-rich glycoprotein	DKFZp779H1622|HPRG|HRGP	3	Antimicrobials
NDRG1	10397	N-myc downstream regulated 1	CAP43|CMT4D|DRG1|GC4|HMSNL|NDR1|NMSL|PROXY1|RIT42|RTP|TARG1|TDD5	8	Antimicrobials
IRF9	10379	interferon regulatory factor 9	IRF-9|ISGF3|ISGF3G|p48	14	Antimicrobials
TRIM22	10346	tripartite motif-containing 22	GPSTAF50|RNF94|STAF50	11	Antimicrobials
LANCL1	10314	LanC lantibiotic synthetase component C-like 1 (bacterial)	GPR69A|p40	2	Antimicrobials
PPP4C	5531	protein phosphatase 4 (formerly X), catalytic subunit	PP4|PPH3|PPX	16	Antimicrobials
HMOX1	3162	heme oxygenase (decycling) 1	HO-1|HSP32|bK286B10	22	Antimicrobials
HMGB1	3146	high-mobility group box 1	DKFZp686A04236|HMG1|HMG3|SBP-1	13	Antimicrobials

HLA-B	3106	major histocompatibility complex, class I, B	AS|HLA-B-7301|HLA-B73|HLAB|HLAC|MGC111087|SPDA1	6	Antimicrobials
RNASE7	84659	ribonuclease, RNase A family, 7	MGC133220	14	Antimicrobials
ABCC4	10257	ATP-binding cassette, sub-family C (CFTR/MRP), member 4	EST170205|MOAT-B|MOATB|MRP4	13	Antimicrobials
HGF	3082	hepatocyte growth factor (hepapoietin A; scatter factor)	F-TCF|HGFB|HPTA|SF	7	Antimicrobials
HDAC1	3065	histone deacetylase 1	DKFZp686H12203|GON-10|HD1|RPD3|RPD3L1	1	Antimicrobials
IL28RA	163702	interleukin 28 receptor, alpha (interferon, lambda receptor)	CRF2/12|IFNLR|IFNLR1|IL-28R1|LICR2	1	Antimicrobials
PLSCR1	5359	phospholipid scramblase 1	MMTRA1B	3	Antimicrobials
B2M	567	beta-2-microglobulin	-	15	Antimicrobials
BACH2	60468	BTB and CNC homology 1, basic leucine zipper transcription factor 2	-	6	Antimicrobials
TANK	10010	TRAF family member-associated NFKB activator	I-TRAF|TRAF2	2	Antimicrobials
PIK3CG	5294	phosphoinositide-3-kinase, catalytic, gamma polypeptide	PI3CG|PI3K|PI3Kgamma|PIK3	7	Antimicrobials
ARRB1	408	arrestin, beta 1	ARB1|ARR1	11	Antimicrobials
RSAD2	91543	radical S-adenosyl methionine domain containing 2	2510004L01Rik|cig33|cig5|vig1	2	Antimicrobials
STAB2	55576	stabilin 2	DKFZp434E0321|FEEL-2|FELE-2|FELL|FELL-2|FEX2|HARE|STAB-2	12	Antimicrobials
TBK1	29110	TANK-binding kinase 1	FLJ11330|NAK|T2K	12	Antimicrobials
PDYN	5173	prodynorphin	MGC26418|PENKB	20	Antimicrobials

PDGFRB	5159	platelet-derived growth factor receptor, beta polypeptide	CD140B|JTK12|PDGF-R-beta|PDGFR|PDGFR1	5	Antimicrobials
PDCD1	5133	programmed cell death 1	CD279|PD1|SLEB2|hPD-1|hPD-l	2	Antimicrobials
PCSK2	5126	proprotein convertase subtilisin/kexin type 2	NEC2|PC2|SPC2	20	Antimicrobials
PCSK1	5122	proprotein convertase subtilisin/kexin type 1	BMIQ12|NEC1|PC1|PC3|SPC3	5	Antimicrobials
ARG2	384	arginase, type II	-	14	Antimicrobials
AQP9	366	aquaporin 9	HsT17287|SSC1	15	Antimicrobials
FASLG	356	Fas ligand (TNF superfamily, member 6)	APT1LG1|CD178|CD95L|FASL|TNFSF6	1	Antimicrobials
APOH	350	apolipoprotein H (beta-2-glycoprotein I)	B2G1|BG	17	Antimicrobials
BIRC5	332	baculoviral IAP repeat-containing 5	API4|EPR-1	17	Antimicrobials
ANXA6	309	annexin A6	ANX6|CBP68	5	Antimicrobials
IL22	50616	interleukin 22	IL-21|IL-22|IL-D110|IL-TIF|IL21|ILTIF|MGC79382|MGC79384|TIFIL-23|TIFa|zcyto18	12	Antimicrobials
VTN	7448	vitronectin	V75|VN|VNT	17	Antimicrobials
VIM	7431	vimentin	FLJ36605	10	Antimicrobials
VCAM1	7412	vascular cell adhesion molecule 1	CD106|DKFZp779G2333|INCAM-100|MGC99561	1	Antimicrobials
PRDX1	5052	peroxiredoxin 1	MSP23|NKEFA|PAG|PAGA|PAGB|PRX1|PRXI|TDPX2	1	Antimicrobials
GFAP	2670	glial fibrillary acidic protein	FLJ45472	17	Antimicrobials

GBP2	2634	guanylate binding protein 2, interferon-inducible	-	1	Antimicrobials
ALB	213	albumin	DKFZp779N1935|PRO0883|PRO0903|PRO1341	4	Antimicrobials
SLC29A3	55315	solute carrier family 29 (nucleoside transporters), member 3	ENT3|FLJ11160	10	Antimicrobials
OAS1	4938	2',5'-oligoadenylate synthetase 1, 40/46kDa	IFI-4|OIAS|OIASI	12	Antimicrobials
AGER	177	advanced glycosylation end product-specific receptor	MGC22357|RAGE	6	Antimicrobials
UNC93B1	81622	unc-93 homolog B1 (C. elegans)	MGC126617|UNC93|UNC93B	11	Antimicrobials
TNFSF4	7292	tumor necrosis factor (ligand) superfamily, member 4	CD134L|CD252|GP34|OX-40L|OX4OL|TXGP1	1	Antimicrobials
NOS1	4842	nitric oxide synthase 1 (neuronal)	IHPS1|NOS|nNOS	12	Antimicrobials
ACTG1	71	actin, gamma 1	ACT|ACTG|DFNA20|DFNA26	17	Antimicrobials
ACTA1	58	actin, alpha 1, skeletal muscle	ACTA|ASMA|CFTD|CFTD1|CFTDM|MPFD|NEM1|NEM2|NEM3	1	Antimicrobials
ACO1	48	aconitase 1, soluble	ACONS|IREB1|IREBP|IREBP1|IRP1	9	Antimicrobials
SERPINA3	12	serpin peptidase inhibitor, clade A (alpha-1 antiproteinase, antitrypsin), member 3	AACT|ACT|GIG24|GIG25|MGC88254	14	Antimicrobials
IL8RA	3577	interleukin 8 receptor, alpha	C-C|C-C-CKR-1|CD128|CD181|CDw128a|CKR-1|CMKAR1|CXCR1|IL8R1|IL8RBA	2	Antimicrobials
CCL15	6359	chemokine (C-C motif) ligand 15	HCC-2|HMRP-2B|LKN1|Lkn-1|MIP-1d|MIP-5|NCC-3|NCC3|SCYA15|SCYL3|SY15	17	Antimicrobials

CCL14	6358	chemokine (C-C motif) ligand 14	CC-1|CC-3|CKb1|FLJ16015|HCC-1|HCC-3|MCIF|NCC-2|NCC2|SCYA14|SCYL2|SY14	17	Antimicrobials
CCL4	6351	chemokine (C-C motif) ligand 4	ACT2|AT744.1|G-26|LAG1|MGC104418|MGC126025|MGC126026|MIP-1-beta|MIP1B|MIP1B1|SCYA2|SCYA4	17	Antimicrobials
CCL16	6360	chemokine (C-C motif) ligand 16	CKb12|HCC-4|ILINCK|LCC-1|LEC|LMC|MGC117051|Mtn-1|NCC-4|NCC4|SCYA16|SCYL4	17	Antimicrobials
CCL19	6363	chemokine (C-C motif) ligand 19	CKb11|ELC|MGC34433|MIP-3b|MIP3B|SCYA19	9	Antimicrobials
CCL13	6357	chemokine (C-C motif) ligand 13	CKb10|MCP-4|MGC17134|NCC-1|NCC1|SCYA13|SCYL1	17	Antimicrobials
CCL18	6362	chemokine (C-C motif) ligand 18 (pulmonary and activation-regulated)	AMAC-1|AMAC1|CKb7|DC-CK1|DCCK1|MIP-4|PARC|SCYA18	17	Antimicrobials
CCL17	6361	chemokine (C-C motif) ligand 17	A-152E5.3|ABCD-2|MGC138271|MGC138273|SCYA17|TARC	16	Antimicrobials
CCL26	10344	chemokine (C-C motif) ligand 26	IMAC|MGC126714|MIP-4a|MIP-4alpha|SCYA26|TSC-1	7	Antimicrobials
CCL22	6367	chemokine (C-C motif) ligand 22	A-152E5.1|ABCD-1|DC/B-CK|MDC|MGC34554|SCYA22|STCP-1	16	Antimicrobials
CCR3	1232	chemokine (C-C motif) receptor 3	CC-CKR-3|CD193|CKR3|CMKBR3|MGC102841	3	Antimicrobials
CCL28	56477	chemokine (C-C motif) ligand 28	CCK1|MEC|MGC71902|SCYA28	5	Antimicrobials
CCL4L2	388372	chemokine (C-C motif) ligand 4-like 2	AT744.2|CCL4L|SCYA4L	17	Antimicrobials
CCBP2	1238	chemokine binding protein 2	CCR10|CCR9|CMKBR9|D6|MGC126678|MGC138250|hD6	3	Antimicrobials

CCR7	1236	chemokine (C-C motif) receptor 7	BLR2|CD197|CDw197|CMKBR7|EBI1	17	Antimicrobials
CCL27	10850	chemokine (C-C motif) ligand 27	ALP|CTACK|CTAK|ESKINE|ILC|PESKY|SCYA27	9	Antimicrobials
CCR8	1237	chemokine (C-C motif) receptor 8	CDw198|CKR-L1|CKRL1|CMKBR8|CMKBRL2|CY6|GPR-CY6|MGC129966|MGC129973|TER1	3	Antimicrobials
CCRL1	51554	chemokine (C-C motif) receptor-like 1	CC-CKR-11|CCBP2|CCR10|CCR11|CCX-CKR|CKR-11|PPR1|VSHK1	3	Antimicrobials
CCR10	2826	chemokine (C-C motif) receptor 10	GPR2	17	Antimicrobials
CCL2	6347	chemokine (C-C motif) ligand 2	GDCF-2|HC11|HSMCR30|MCAF|MCP-1|MCP1|MGC9434|SCYA2|SMC-CF	17	Antimicrobials
CCL21	6366	chemokine (C-C motif) ligand 21	6Ckine|CKb9|ECL|MGC34555|SCYA21|SLC|TCA4	9	Antimicrobials
CCL7	6354	chemokine (C-C motif) ligand 7	FIC|MARC|MCP-3|MCP3|MGC138463|MGC138465|NC28|SCYA6|SCYA7	17	Antimicrobials
CCL5	6352	chemokine (C-C motif) ligand 5	D17S136E|MGC17164|RANTES|SCYA5|SISd|TCP228	17	Antimicrobials
CCL3	6348	chemokine (C-C motif) ligand 3	G0S19-1|LD78ALPHA|MIP-1-alpha|MIP1A|SCYA3	17	Antimicrobials
CCL20	6364	chemokine (C-C motif) ligand 20	CKb4|LARC|MIP-3a|MIP3A|SCYA20|ST38	2	Antimicrobials
CCL11	6356	chemokine (C-C motif) ligand 11	MGC22554|SCYA11	17	Antimicrobials
CCR5	1234	chemokine (C-C motif) receptor 5	CC-CKR-5|CCCKR5|CD195|CKR-5|CKR5|CMKBR5|FLJ78003|IDDM22	3	Antimicrobials
CCL23	6368	chemokine (C-C motif) ligand 23	CK-BETA-8|CKb8|Ckb-8|Ckb-8-1|MIP-	17	Antimicrobials

			3|MIP3|MPIF-1|SCYA23		
CCL25	6370	chemokine (C-C motif) ligand 25	Ckb15|MGC150327|SCYA25|TECK	19	Antimicrobials
CCL1	6346	chemokine (C-C motif) ligand 1	I-309|P500|SCYA1|SISe|TCA3	17	Antimicrobials
CCL3L3	414062	chemokine (C-C motif) ligand 3-like 3	464.2|D17S1718|LD78|LD78BETA|MGC12815|SCYA3L|SCYA3L1	17	Antimicrobials
CCL4L1	9560	chemokine (C-C motif) ligand 4-like 1	AT744.2|CCL4L|LAG-1|LAG1|SCYA4L	17	Antimicrobials
CXCL12	6387	chemokine (C-X-C motif) ligand 12 (stromal cell-derived factor 1)	PBSF|SCYB12|SDF-1a|SDF-1b|SDF1|SDF1A|SDF1B|TLSF-a|TLSF-b|TPAR1	10	Antimicrobials
XCL1	6375	chemokine (C motif) ligand 1	ATAC|LPTN|LTN|SCM-1|SCM-1a|SCM1|SCYC1	1	Antimicrobials
CCL8	6355	chemokine (C-C motif) ligand 8	HC14|MCP-2|MCP2|SCYA10|SCYA8	17	Antimicrobials
CCL3L1	6349	chemokine (C-C motif) ligand 3-like 1	464.2|D17S1718|G0S19-2|LD78|LD78BETA|MGC104178|MGC12815|MGC182017|MIP1AP|SCYA3L|SCYA3L1	17	Antimicrobials
CCR1	1230	chemokine (C-C motif) receptor 1	CD191|CKR-1|CKR1|CMKBR1|HM145|MIP1aR|SCYAR1	3	Antimicrobials
CCL24	6369	chemokine (C-C motif) ligand 24	Ckb-6|MPIF-2|MPIF2|SCYA24	7	Antimicrobials
XCL2	6846	chemokine (C motif) ligand 2	SCM-1b|SCM1B|SCYC2	1	Antimicrobials
CXCL1	2919	chemokine (C-X-C motif) ligand 1 (melanoma growth stimulating activity, alpha)	FSP|GRO1|GROa|MGSA|MGSA-a|NAP-3|SCYB1	4	Antimicrobials
CXCL10	3627	chemokine (C-X-C motif) ligand 10	C7|IFI10|INP10|IP-10|SCYB10|crg-2|gIP-10|mob-1	4	Antimicrobials
CXCR4	7852	chemokine (C-X-C motif) receptor 4	CD184|D2S201E|FB22|HM89|HSY3RR|LAP3|LCR1|LESTR|NPY3R|NPYR|NPYRL|NPYY3R|WHIM	2	Antimicrobials

CXCL2	2920	chemokine (C-X-C motif) ligand 2	CINC-2a|GRO2|GROb|MGSA-b|MIP-2a|MIP2|MIP2A|SCYB2	4	Antimicrobials
CXCR6	10663	chemokine (C-X-C motif) receptor 6	BONZO|CD186|STRL33|TYMSTR	3	Antimicrobials
CCR4	1233	chemokine (C-C motif) receptor 4	CC-CKR-4|CD194|CKR4|CMKBR4|ChemR13|HGCN:14099|K5-5|MGC88293	3	Antimicrobials
CXCL11	6373	chemokine (C-X-C motif) ligand 11	H174|I-TAC|IP-9|IP9|MGC102770|SCYB11|SCYB9B|b-R1	4	Antimicrobials
FAM19A5	25817	family with sequence similarity 19 (chemokine (C-C motif)-like), member A5	QLLK5208|TAFA-5|TAFA5|UNQ5208	22	Antimicrobials
FAM19A3	284467	family with sequence similarity 19 (chemokine (C-C motif)-like), member A3	MGC138473|TAFA-3|TAFA3	1	Antimicrobials
FAM19A4	151647	family with sequence similarity 19 (chemokine (C-C motif)-like), member A4	FLJ25161|TAFA-4|TAFA4	3	Antimicrobials
FAM19A1	407738	family with sequence similarity 19 (chemokine (C-C motif)-like), member A1	TAFA-1|TAFA1	3	Antimicrobials
FAM19A2	338811	family with sequence similarity 19 (chemokine (C-C motif)-like), member A2	DKFZp761E1217|DKFZp781P0552|MGC42403|TAFA-2|TAFA2	12	Antimicrobials
CCL14-CCL15	348249	chemokine ligand 14, chemokine ligand 15 transcription unit	-	17	Antimicrobials

IL6	3569	interleukin 6 (interferon, beta 2)	BSF2|HGF|HSF|IFNB2|IL-6	7	Antimicrobials
TNF	7124	tumor necrosis factor (TNF superfamily, member 2)	DIF|TNF-alpha|TNFA|TNFSF2	6	Antimicrobials
IL1B	3553	interleukin 1, beta	IL-1|IL1-BETA|IL1F2	2	Antimicrobials
IL18	3606	interleukin 18 (interferon-gamma-inducing factor)	IGIF|IL-18|IL-1g|IL1F4|MGC12320	11	Antimicrobials
PTK2B	2185	PTK2B protein tyrosine kinase 2 beta	CADTK|CAKB|FADK2|FAK2|FRNK|PKB|PTK|PYK2|RAFTK	8	Antimicrobials
VEGFA	7422	vascular endothelial growth factor A	MGC70609|MVCD1|VEGF|VEGF-A|VPF	6	Antimicrobials
IL4	3565	interleukin 4	BCGF-1|BCGF1|BSF1|IL-4|MGC79402	5	Antimicrobials
CDH1	999	cadherin 1, type 1, E-cadherin (epithelial)	Arc-1|CD324|CDHE|ECAD|LCAM|UVO	16	Antimicrobials
CD40	958	CD40 molecule, TNF receptor superfamily member 5	Bp50|CDW40|MGC9013|TNFRSF5|p50	20	Antimicrobials
DEFB103A	55894	defensin, beta 103A	DEFB103|DEFB3|HBD-3|HBD3|HBP-3|HBP3	8	Antimicrobials
F2RL1	2150	coagulation factor II (thrombin) receptor-like 1	GPR11|PAR2	5	Antimicrobials
MMP9	4318	matrix metallopeptidase 9 (gelatinase B, 92kDa gelatinase, 92kDa type IV collagenase)	CLG4B|GELB|MMP-9	20	Antimicrobials
LTBP1	4052	latent transforming growth factor beta binding protein 1	MGC163161	2	Antimicrobials
DEFB4	1673	defensin, beta 4	DEFB-2|DEFB102|DEFB2|HBD-2|SAP1	8	Antimicrobials
TNFSF10	8743	tumor necrosis factor (ligand) superfamily, member 10	APO2L|Apo-2L|CD253|TL2|TRAIL	3	Antimicrobials

IL13	3596	interleukin 13	ALRH|BHR1|IL-13|MGC116786|MGC116788|MGC116789|P600	5	Antimicrobials
IL10	3586	interleukin 10	CSIF|IL-10|IL10A|MGC126450|MGC126451|TGIF	1	Antimicrobials
IL2	3558	interleukin 2	IL-2|TCGF|lymphokine	4	Antimicrobials
PPARG	5468	peroxisome proliferator-activated receptor gamma	CIMT1|NR1C3|PPARG1|PPARG2|PPARgamma	3	Antimicrobials
FGR	2268	Gardner-Rasheed feline sarcoma viral (v-fgr) oncogene homolog	FLJ43153|MGC75096|SRC2|c-fgr|c-src2|p55c-fgr|p58c-fgr	1	Antimicrobials
MIF	4282	macrophage migration inhibitory factor (glycosylation-inhibiting factor)	GIF|GLIF|MMIF	22	Antimicrobials
CRP	1401	C-reactive protein, pentraxin-related	MGC149895|MGC88244|PTX1	1	Antimicrobials
JAK2	3717	Janus kinase 2	JTK10	9	Antimicrobials
IL1A	3552	interleukin 1, alpha	IL-1A|IL1|IL1-ALPHA|IL1F1	2	Antimicrobials
PTK2	5747	PTK2 protein tyrosine kinase 2	FADK|FAK|FAK1|pp125FAK	8	Antimicrobials
PTGDR	5729	prostaglandin D2 receptor (DP)	AS1|ASRT1|DP|DP1|MGC49004	14	Antimicrobials
CD86	942	CD86 molecule	B7-2|B70|CD28LG2|LAB72|MGC34413	3	Antimicrobials
HCK	3055	hemopoietic cell kinase	JTK9	20	Antimicrobials
ARRB1	408	arrestin, beta 1	ARB1|ARR1	11	Antimicrobials
GNAI1	2770	guanine nucleotide binding protein (G protein), alpha inhibiting activity polypeptide 1	Gi	7	Antimicrobials
VDR	7421	vitamin D (1,25-	NR1I1	12	Antimicrobials

		dihydroxyvitamin D3) receptor			
OLR1	4973	oxidized low density lipoprotein (lectin-like) receptor 1	CLEC8A|LOX1|SCARE1	12	Antimicrobials
ADRBK1	156	adrenergic, beta, receptor kinase 1	BARK1|BETA-ARK1|FLJ16718|GRK2	11	Antimicrobials
TXK	7294	TXK tyrosine kinase	BTKL|MGC22473|PSCTK5|PTK4|RLK|TKL	4	Antimicrobials
RNASE2	6036	ribonuclease, RNase A family, 2 (liver, eosinophil-derived neurotoxin)	EDN|RNS2	14	Antimicrobials
CD79A	973	CD79a molecule, immunoglobulin-associated alpha	IGA|MB-1	19	BCR Signaling Pathway
CD79B	974	CD79b molecule, immunoglobulin-associated beta	B29|IGB	17	BCR Signaling Pathway
LYN	4067	v-yes-1 Yamaguchi sarcoma viral related oncogene homolog	FLJ26625|JTK8	8	BCR Signaling Pathway
SYK	6850	spleen tyrosine kinase	DKFZp313N1010|FLJ25043|FLJ37489	9	BCR Signaling Pathway
BTK	695	Bruton agammaglobulinemia tyrosine kinase	AGMX1|AT|ATK|BPK|IMD1|MGC126261|MGC126262|PSCTK1|XLA	X	BCR Signaling Pathway
BLNK	29760	B-cell linker	BASH|BLNK-S|LY57|MGC111051|SLP-65|SLP65	10	BCR Signaling Pathway
VAV3	10451	vav 3 guanine nucleotide exchange factor	FLJ40431	1	BCR Signaling Pathway
VAV1	7409	vav 1 guanine nucleotide exchange factor	VAV	19	BCR Signaling Pathway
VAV2	7410	vav 2 guanine nucleotide	-	9	BCR Signaling Pathway

		exchange factor			
RAC1	5879	ras-related C3 botulinum toxin substrate 1 (rho family, small GTP binding protein Rac1)	MGC111543|MIG5|TC-25|p21-Rac1	7	BCR Signaling Pathway
RAC2	5880	ras-related C3 botulinum toxin substrate 2 (rho family, small GTP binding protein Rac2)	EN-7|Gx|HSPC022	22	BCR Signaling Pathway
RAC3	5881	ras-related C3 botulinum toxin substrate 3 (rho family, small GTP binding protein Rac3)	-	17	BCR Signaling Pathway
PPP3CA	5530	protein phosphatase 3 (formerly 2B), catalytic subunit, alpha isoform	CALN|CALNA|CALNA1|CCN1|CNA1|PPP2B	4	BCR Signaling Pathway
PPP3CB	5532	protein phosphatase 3 (formerly 2B), catalytic subunit, beta isoform	CALNA2|CALNB	10	BCR Signaling Pathway
PPP3CC	5533	protein phosphatase 3 (formerly 2B), catalytic subunit, gamma isoform	CALNA3	8	BCR Signaling Pathway
CHP	11261	calcium binding protein P22	SLC9A1BP	15	BCR Signaling Pathway
PPP3R1	5534	protein phosphatase 3 (formerly 2B), regulatory subunit B, alpha isoform	CALNB1|CNB|CNB1	2	BCR Signaling Pathway
PPP3R2	5535	protein phosphatase 3 (formerly 2B), regulatory subunit B, beta isoform	PPP3RL	9	BCR Signaling Pathway

CHP2	63928	calcineurin B homologous protein 2	-	16	BCR Signaling Pathway
NFAT5	10725	nuclear factor of activated T-cells 5, tonicity-responsive	KIAA0827|NF-AT5|NFATL1|NFATZ|OREBP|TONEBP	16	BCR Signaling Pathway
NFATC1	4772	nuclear factor of activated T-cells, cytoplasmic, calcineurin-dependent 1	MGC138448|NF-ATC|NFAT2|NFATc	18	BCR Signaling Pathway
NFATC2	4773	nuclear factor of activated T-cells, cytoplasmic, calcineurin-dependent 2	NFAT1|NFATP	20	BCR Signaling Pathway
NFATC3	4775	nuclear factor of activated T-cells, cytoplasmic, calcineurin-dependent 3	NFAT4|NFATX	16	BCR Signaling Pathway
NFATC4	4776	nuclear factor of activated T-cells, cytoplasmic, calcineurin-dependent 4	NF-ATc4|NFAT3	14	BCR Signaling Pathway
HRAS	3265	v-Ha-ras Harvey rat sarcoma viral oncogene homolog	C-BAS/HAS|C-H-RAS|C-HA-RAS1|CTLO|H-RASIDX|HAMSV|HRAS1|K-RAS|N-RAS|RASH1	11	BCR Signaling Pathway
KRAS	3845	v-Ki-ras2 Kirsten rat sarcoma viral oncogene homolog	C-K-RAS|K-RAS2A|K-RAS2B|K-RAS4A|K-RAS4B|KI-RAS|KRAS1|KRAS2|NS3|RASK2	12	BCR Signaling Pathway
NRAS	4893	neuroblastoma RAS viral (v-ras) oncogene homolog	ALPS4|N-ras|NRAS1	1	BCR Signaling Pathway
FOS	2353	v-fos FBJ murine osteosarcoma viral oncogene homolog	AP-1|C-FOS	14	BCR Signaling Pathway
JUN	3725	jun oncogene	AP-1|AP1|c-Jun	1	BCR Signaling Pathway
CARD11	84433	caspase recruitment domain	BIMP3|CARMA1|MGC133069	7	BCR Signaling Pathway

		family, member 11			
BCL10	8915	B-cell CLL/lymphoma 10	CARMEN|CIPER|CLAP|c-E10|mE10	1	BCR Signaling Pathway
MALT1	10892	mucosa associated lymphoid tissue lymphoma translocation gene 1	DKFZp434L132|MLT|MLT1	18	BCR Signaling Pathway
CHUK	1147	conserved helix-loop-helix ubiquitous kinase	IKBKA|IKK-alpha|IKK1|IKKA|NFKBIKA|TCF16	10	BCR Signaling Pathway
IKBKB	3551	inhibitor of kappa light polypeptide gene enhancer in B-cells, kinase beta	FLJ40509|IKK-beta|IKK2|IKKB|MGC131801|NFKBIKB	8	BCR Signaling Pathway
IKBKG	8517	inhibitor of kappa light polypeptide gene enhancer in B-cells, kinase gamma	AMCBX1|FIP-3|FIP3|Fip3p|IKK-gamma|IP|IP1|IP2|IPD2|NEMO	X	BCR Signaling Pathway
NFKB1	4790	nuclear factor of kappa light polypeptide gene enhancer in B-cells 1	DKFZp686C01211|EBP-1|KBF1|MGC54151|NF-kappa-B|NFKB-p105|NFKB-p50|p105|p50	4	BCR Signaling Pathway
RELA	5970	v-rel reticuloendotheliosis viral oncogene homolog A (avian)	MGC131774|NFKB3|p65	11	BCR Signaling Pathway
NFKBIA	4792	nuclear factor of kappa light polypeptide gene enhancer in B-cells inhibitor, alpha	IKBA|MAD-3|NFKBI	14	BCR Signaling Pathway
NFKBIB	4793	nuclear factor of kappa light polypeptide gene enhancer in B-cells inhibitor, beta	IKBB|TRIP9	19	BCR Signaling Pathway
NFKBIE	4794	nuclear factor of kappa light polypeptide gene enhancer in B-	IKBE	6	BCR Signaling Pathway

		cells inhibitor, epsilon			
CD81	975	CD81 molecule	S5.7|TAPA1|TSPAN28	11	BCR Signaling Pathway
CD19	930	CD19 molecule	B4|MGC12802	16	BCR Signaling Pathway
CR2	1380	complement component (3d/Epstein Barr virus) receptor 2	C3DR|CD21|SLEB9	1	BCR Signaling Pathway
PIK3R5	23533	phosphoinositide-3-kinase, regulatory subunit 5	F730038I15Rik|FOAP-2|P101-PI3K|p101	17	BCR Signaling Pathway
PIK3R1	5295	phosphoinositide-3-kinase, regulatory subunit 1 (alpha)	GRB1|p85|p85-ALPHA	5	BCR Signaling Pathway
PIK3R2	5296	phosphoinositide-3-kinase, regulatory subunit 2 (beta)	P85B|p85|p85-BETA	19	BCR Signaling Pathway
PIK3R3	8503	phosphoinositide-3-kinase, regulatory subunit 3 (gamma)	DKFZp686P05226|FLJ41892|p55|p55-GAMMA	1	BCR Signaling Pathway
PIK3CA	5290	phosphoinositide-3-kinase, catalytic, alpha polypeptide	MGC142161|MGC142163|PI3K|p110-alpha	3	BCR Signaling Pathway
PIK3CB	5291	phosphoinositide-3-kinase, catalytic, beta polypeptide	DKFZp779K1237|MGC133043|PI3K|PI3KCB|PI3Kbeta|PIK3C1|p110-BETA	3	BCR Signaling Pathway
PIK3CD	5293	phosphoinositide-3-kinase, catalytic, delta polypeptide	p110D	1	BCR Signaling Pathway
PIK3CG	5294	phosphoinositide-3-kinase, catalytic, gamma polypeptide	PI3CG|PI3K|PI3Kgamma|PIK3	7	BCR Signaling Pathway
AKT3	10000	v-AKT murine thymoma viral oncogene homolog 3 (protein kinase B, gamma)	DKFZp434N0250|PKB-GAMMA|PKBG|PRKBG|RAC-PK-gamma|RAC-gamma|STK-2	1	BCR Signaling Pathway
AKT1	207	v-AKT murine thymoma viral oncogene homolog 1	AKT|MGC99656|PKB|PKB-ALPHA|PRKBA|RAC|RAC-ALPHA	14	BCR Signaling Pathway

AKT2	208	v-AKT murine thymoma viral oncogene homolog 2	PKBB|PKBBETA|PRKBB|RAC-BETA	19	BCR Signaling Pathway
GSK3B	2932	glycogen synthase kinase 3 beta	-	3	BCR Signaling Pathway
INPP5D	3635	inositol polyphosphate-5-phosphatase, 145kDa	MGC104855|MGC142140|MGC142142|SHIP|SHIP1|SIP-145|hp51CN	2	BCR Signaling Pathway
CD22	933	CD22 molecule	FLJ22814|MGC130020|SIGLEC-2|SIGLEC2	19	BCR Signaling Pathway
CD72	971	CD72 molecule	CD72b|LYB2	9	BCR Signaling Pathway
PTPN6	5777	protein tyrosine phosphatase, non-receptor type 6	HCP|HCPH|HPTP1C|PTP-1C|SH-PTP1|SHP-1|SHP-1L|SHP1	12	BCR Signaling Pathway
LILRB3	11025	leukocyte immunoglobulin-like receptor, subfamily B (with TM and ITIM domains), member 3	CD85A|HL9|ILT5|LIR-3|LIR3|MGC138403|PIRB	19	BCR Signaling Pathway
FCGR2B	2213	Fc fragment of IgG, low affinity IIb, receptor (CD32)	CD32|CD32B|FCG2|FCGR2|IGFR2	1	BCR Signaling Pathway
RASGRP3	25780	RAS guanyl releasing protein 3 (calcium and DAG-regulated)	GRP3|KIAA0846	2	BCR Signaling Pathway
PLCG2	5336	phospholipase C, gamma 2 (phosphatidylinositol-specific)	-	16	BCR Signaling Pathway
PRKCB	5579	protein kinase C, beta	MGC41878|PKC-beta|PKCB|PRKCB1|PRKCB2	16	BCR Signaling Pathway
IFITM1	8519	interferon induced transmembrane protein 1 (9-27)	9-27|CD225|IFI17|LEU13	11	BCR Signaling Pathway
IGH@	3492	immunoglobulin heavy locus	DKFZp686C15213|IGH|IGH.1@|IGHDY1|MGC72071|MGC88774	14	BCR Signaling Pathway
IGHA1	3493	immunoglobulin heavy constant alpha 1	FLJ14473|FLJ35065|FLJ35500|FLJ36402|FLJ39698|FLJ40001|FLJ41548|FLJ41552|FLJ41789|FLJ43248|FLJ43594|FLJ44293|FLJ46028|FLJ466	14	BCR Signaling Pathway

			21|FLJ46724|FLJ46811|FLJ46824|FLJ90170|IgA1|MGC102857		
IGHA2	3494	immunoglobulin heavy constant alpha 2 (A2m marker)	-	14	BCR Signaling Pathway
IGHD	3495	immunoglobulin heavy constant delta	FLJ00382|FLJ46727|MGC29633	14	BCR Signaling Pathway
IGHD@	50648	immunoglobulin heavy diversity group	IGD1|IGHDY1	14	BCR Signaling Pathway
IGHD1-1	28510	immunoglobulin heavy diversity 1-1	IGHD11	14	BCR Signaling Pathway
IGHD1-14	28508	immunoglobulin heavy diversity 1-14 (non-functional)	DM2|IGHD114	14	BCR Signaling Pathway
IGHD1-20	28507	immunoglobulin heavy diversity 1-20	IGHD120	14	BCR Signaling Pathway
IGHD1-26	28506	immunoglobulin heavy diversity 1-26	IGHD126	14	BCR Signaling Pathway
IGHD1-7	28509	immunoglobulin heavy diversity 1-7	DM1|IGHD17	14	BCR Signaling Pathway
IGHD2-15	28503	immunoglobulin heavy diversity 2-15	D2|IGHD215	14	BCR Signaling Pathway
IGHD2-2	28505	immunoglobulin heavy diversity 2-2	IGHD22	14	BCR Signaling Pathway
IGHD2-21	28502	immunoglobulin heavy diversity 2-21	IGHD221	14	BCR Signaling Pathway
IGHD2-8	28504	immunoglobulin heavy diversity 2-8	DLR1|IGHD28	14	BCR Signaling Pathway

IGHD3-10	28499	immunoglobulin heavy diversity 3-10	DXP'1|IGHD310	14	BCR Signaling Pathway
IGHD3-16	28498	immunoglobulin heavy diversity 3-16	IGHD316	14	BCR Signaling Pathway
IGHD3-22	28497	immunoglobulin heavy diversity 3-22	IGHD322	14	BCR Signaling Pathway
IGHD3-3	28501	immunoglobulin heavy diversity 3-3	DXP4|IGHD33	14	BCR Signaling Pathway
IGHD3-9	28500	immunoglobulin heavy diversity 3-9	DXP1|IGHD39	14	BCR Signaling Pathway
IGHD4-11	28495	immunoglobulin heavy diversity 4-11 (non-functional)	DA1|IGHD411	14	BCR Signaling Pathway
IGHD4-17	28494	immunoglobulin heavy diversity 4-17	IGHD417	14	BCR Signaling Pathway
IGHD4-23	28493	immunoglobulin heavy diversity 4-23 (non-functional)	IGHD423	14	BCR Signaling Pathway
IGHD4-4	28496	immunoglobulin heavy diversity 4-4	DA4|IGHD44	14	BCR Signaling Pathway
IGHD5-12	28491	immunoglobulin heavy diversity 5-12	DK1|IGHD512	14	BCR Signaling Pathway
IGHD5-18	28490	immunoglobulin heavy diversity 5-18	IGHD518	14	BCR Signaling Pathway
IGHD5-24	28489	immunoglobulin heavy diversity 5-24 (non-functional)	IGHD524	14	BCR Signaling Pathway
IGHD5-5	28492	immunoglobulin heavy diversity 5-5	DK4|IGHD55	14	BCR Signaling Pathway

IGHD6-13	28487	immunoglobulin heavy diversity 6-13	DN1|IGHD613	14	BCR Signaling Pathway
IGHD6-19	28486	immunoglobulin heavy diversity 6-19	IGHD619	14	BCR Signaling Pathway
IGHD6-25	28485	immunoglobulin heavy diversity 6-25	IGHD625	14	BCR Signaling Pathway
IGHD6-6	28488	immunoglobulin heavy diversity 6-6	D(N4)|IGHD66	14	BCR Signaling Pathway
IGHD7-27	28484	immunoglobulin heavy diversity 7-27	DHQ52|IGHD727	14	BCR Signaling Pathway
IGHE	3497	immunoglobulin heavy constant epsilon	IgE	14	BCR Signaling Pathway
IGHG1	3500	immunoglobulin heavy constant gamma 1 (G1m marker)	-	14	BCR Signaling Pathway
IGHG2	3501	immunoglobulin heavy constant gamma 2 (G2m marker)	DKFZp686I04196	14	BCR Signaling Pathway
IGHG3	3502	immunoglobulin heavy constant gamma 3 (G3m marker)	DKFZp686H11213|FLJ39988|FLJ40036|FLJ40253|FLJ40587|FLJ40789|FLJ40834|IgG3|MGC45809	14	BCR Signaling Pathway
IGHG4	3503	immunoglobulin heavy constant gamma 4 (G4m marker)	MGC117419	14	BCR Signaling Pathway
IGHJ@	3506	immunoglobulin heavy joining group	IGHJ	14	BCR Signaling Pathway
IGHJ1	28483	immunoglobulin heavy joining 1	JH1	14	BCR Signaling Pathway
IGHJ2	28481	immunoglobulin heavy joining 2	JH2	14	BCR Signaling Pathway
IGHJ3	28479	immunoglobulin heavy joining 3	JH3b	14	BCR Signaling Pathway

IGHJ4	28477	immunoglobulin heavy joining 4	JH4b	14	BCR Signaling Pathway
IGHJ5	28476	immunoglobulin heavy joining 5	JH5b	14	BCR Signaling Pathway
IGHJ6	28475	immunoglobulin heavy joining 6	JH6b	14	BCR Signaling Pathway
IGHM	3507	immunoglobulin heavy constant mu	DKFZp686I15196|DKFZp686I15212|FLJ00385|MGC104996|MGC52291|MU|VH	14	BCR Signaling Pathway
IGHV@	3509	immunoglobulin heavy variable group	IGHV	14	BCR Signaling Pathway
IGHV1-18	28468	immunoglobulin heavy variable 1-18	IGHV118	14	BCR Signaling Pathway
IGHV1-2	28474	immunoglobulin heavy variable 1-2	IGHV12	14	BCR Signaling Pathway
IGHV1-24	28467	immunoglobulin heavy variable 1-24	IGHV124|VH	14	BCR Signaling Pathway
IGHV1-3	28473	immunoglobulin heavy variable 1-3	IGHV13	14	BCR Signaling Pathway
IGHV1-45	28466	immunoglobulin heavy variable 1-45	IGHV145|VH	14	BCR Signaling Pathway
IGHV1-46	28465	immunoglobulin heavy variable 1-46	IGHV146	14	BCR Signaling Pathway
IGHV1-58	28464	immunoglobulin heavy variable 1-58	IGHV158|VH	14	BCR Signaling Pathway
IGHV1-69	28461	immunoglobulin heavy variable 1-69	IGHV1-E|IGHV169|IGHV1E	14	BCR Signaling Pathway
IGHV1-8	28472	immunoglobulin heavy variable 1-8	IGHV18	14	BCR Signaling Pathway
IGHV1-C	28460	immunoglobulin heavy variable	IGHV1C	14	BCR Signaling Pathway

		1-C (provisional, non-functional)			
IGHV1-F	28458	immunoglobulin heavy variable 1-F (provisional)	IGHV1F	14	BCR Signaling Pathway
IGHV2-26	28455	immunoglobulin heavy variable 2-26	IGHV226|VH	14	BCR Signaling Pathway
IGHV2-5	28457	immunoglobulin heavy variable 2-5	IGHV25|VH	14	BCR Signaling Pathway
IGHV2-70	28454	immunoglobulin heavy variable 2-70	IGHV270|VH	14	BCR Signaling Pathway
IGHV3-11	28450	immunoglobulin heavy variable 3-11 (gene/pseudogene)	IGHV311|VH	14	BCR Signaling Pathway
IGHV3-13	28449	immunoglobulin heavy variable 3-13	IGHV313	14	BCR Signaling Pathway
IGHV3-15	28448	immunoglobulin heavy variable 3-15	IGHV315|VH	14	BCR Signaling Pathway
IGHV3-16	28447	immunoglobulin heavy variable 3-16 (non-functional)	IGHV316|VH	14	BCR Signaling Pathway
IGHV3-20	28445	immunoglobulin heavy variable 3-20	IGHV320|VH	14	BCR Signaling Pathway
IGHV3-21	28444	immunoglobulin heavy variable 3-21	IGHV321|VH	14	BCR Signaling Pathway
IGHV3-23	28442	immunoglobulin heavy variable 3-23	DP47|IGHV323|V3-23|VH26	14	BCR Signaling Pathway
IGHV3-30	28439	immunoglobulin heavy variable 3-30	IGHV330|VH	14	BCR Signaling Pathway
IGHV3-30-	57290	immunoglobulin heavy variable	IGHV3-3|IGHV3303	14	BCR Signaling Pathway

3		3-30-3			
IGHV3-30-5	89770	immunoglobulin heavy variable 3-30-5	IGHV3-3|IGHV3305	14	BCR Signaling Pathway
IGHV3-33	28434	immunoglobulin heavy variable 3-33	IGHV333|VH	14	BCR Signaling Pathway
IGHV3-35	28432	immunoglobulin heavy variable 3-35 (non-functional)	IGHV335|VH	14	BCR Signaling Pathway
IGHV3-38	28429	immunoglobulin heavy variable 3-38 (non-functional)	IGHV338|VH	14	BCR Signaling Pathway
IGHV3-43	28426	immunoglobulin heavy variable 3-43	IGHV343|VH	14	BCR Signaling Pathway
IGHV3-48	28424	immunoglobulin heavy variable 3-48	IGHV348|VH	14	BCR Signaling Pathway
IGHV3-49	28423	immunoglobulin heavy variable 3-49	IGHV349|VH	14	BCR Signaling Pathway
IGHV3-53	28420	immunoglobulin heavy variable 3-53	IGHV353|VH	14	BCR Signaling Pathway
IGHV3-64	28414	immunoglobulin heavy variable 3-64	IGHV364|VH	14	BCR Signaling Pathway
IGHV3-66	28412	immunoglobulin heavy variable 3-66	IGHV366|VH	14	BCR Signaling Pathway
IGHV3-7	28452	immunoglobulin heavy variable 3-7	IGHV37|VH	14	BCR Signaling Pathway
IGHV3-72	28410	immunoglobulin heavy variable 3-72	IGHV372|VH	14	BCR Signaling Pathway
IGHV3-73	28409	immunoglobulin heavy variable	IGHV373|VH	14	BCR Signaling Pathway

		3-73			
IGHV3-74	28408	immunoglobulin heavy variable 3-74	IGHV374|VH	14	BCR Signaling Pathway
IGHV3-9	28451	immunoglobulin heavy variable 3-9	IGHV39|VH	14	BCR Signaling Pathway
IGHV3-D	28404	immunoglobulin heavy variable 3-D (provisional)	IGHV3D	14	BCR Signaling Pathway
IGHV3-H	28402	immunoglobulin heavy variable 3-H pseudogene (provisional)	IGHV3H	14	BCR Signaling Pathway
IGHV4-28	28400	immunoglobulin heavy variable 4-28	IGHV428|VH	14	BCR Signaling Pathway
IGHV4-30-1	28399	immunoglobulin heavy variable 4-30-1	IGHV4-3	14	BCR Signaling Pathway
IGHV4-30-2	28398	immunoglobulin heavy variable 4-30-2	IGHV4-3|IGHV4302	14	BCR Signaling Pathway
IGHV4-30-4	28397	immunoglobulin heavy variable 4-30-4	IGHV4-3|IGHV4304	14	BCR Signaling Pathway
IGHV4-31	28396	immunoglobulin heavy variable 4-31	FLJ45507|IGHV431	14	BCR Signaling Pathway
IGHV4-34	28395	immunoglobulin heavy variable 4-34	IGHV434|VH	14	BCR Signaling Pathway
IGHV4-39	28394	immunoglobulin heavy variable 4-39	IGHV439|VH	14	BCR Signaling Pathway
IGHV4-4	28401	immunoglobulin heavy variable 4-4	IGHV44|VH	14	BCR Signaling Pathway
IGHV4-59	28392	immunoglobulin heavy variable	IGHV459|VH	14	BCR Signaling Pathway

		4-59			
IGHV4-61	28391	immunoglobulin heavy variable 4-61	IGHV461|VH	14	BCR Signaling Pathway
IGHV4-B	28389	immunoglobulin heavy variable 4-B (provisional)	IGHV4B	14	BCR Signaling Pathway
IGHV5-51	28388	immunoglobulin heavy variable 5-51	IGHV551|VH	14	BCR Signaling Pathway
IGHV5-A	28386	immunoglobulin heavy variable 5-A (provisional, gene/pseudogene)	IGHV5A	14	BCR Signaling Pathway
IGHV6-1	28385	immunoglobulin heavy variable 6-1	IGHV61|VH	14	BCR Signaling Pathway
IGHV7-4-1	57289	immunoglobulin heavy variable 7-4-1	IGHV7-41|IGHV741	14	BCR Signaling Pathway
IGHV7-81	28378	immunoglobulin heavy variable 7-81 (non-functional)	IGHV781	14	BCR Signaling Pathway
IGK@	50802	immunoglobulin kappa locus	FLJ26296|IGK|IGKC|MGC22645|MGC27376|MGC40426|MGC71990	2	BCR Signaling Pathway
IGKC	3514	immunoglobulin kappa constant	HCAK1|Km|MGC111575|MGC62011|MGC72072|MGC88770|MGC88771|MGC88809	2	BCR Signaling Pathway
IGKDEL	3515	immunoglobulin kappa deleting element or like	IGKDE	2	BCR Signaling Pathway
IGKJ@	7842	immunoglobulin kappa joining group	IGKJ	2	BCR Signaling Pathway
IGKJ1	28950	immunoglobulin kappa joining 1	J1	2	BCR Signaling Pathway
IGKJ2	28949	immunoglobulin kappa joining 2	J2	2	BCR Signaling Pathway

IGKJ3	28948	immunoglobulin kappa joining 3	J3	2	BCR Signaling Pathway
IGKJ4	28947	immunoglobulin kappa joining 4	J4	2	BCR Signaling Pathway
IGKJ5	28946	immunoglobulin kappa joining 5	J5	2	BCR Signaling Pathway
IGKV@	3519	immunoglobulin kappa variable group	IGKV|IGKV1|IGKV1@|IGKV2|IGKV2@|IGKV3|IGKV3@	2	BCR Signaling Pathway
IGKV1-12	28940	immunoglobulin kappa variable 1-12	IGKV112|L19	2	BCR Signaling Pathway
IGKV1-13	28939	immunoglobulin kappa variable 1-13 (gene/pseudogene)	IGKV113|L18	2	BCR Signaling Pathway
IGKV1-16	28938	immunoglobulin kappa variable 1-16	IGKV116|L1	2	BCR Signaling Pathway
IGKV1-17	28937	immunoglobulin kappa variable 1-17	A30|IGKV117	2	BCR Signaling Pathway
IGKV1-27	28935	immunoglobulin kappa variable 1-27	A20|IGKV127	2	BCR Signaling Pathway
IGKV1-33	28933	immunoglobulin kappa variable 1-33	IGKV133|O18	2	BCR Signaling Pathway
IGKV1-37	28931	immunoglobulin kappa variable 1-37 (non-functional)	IGKV137|O14	2	BCR Signaling Pathway
IGKV1-39	28930	immunoglobulin kappa variable 1-39 (gene/pseudogene)	IGKV139|O12|O12a	2	BCR Signaling Pathway
IGKV1-5	28299	immunoglobulin kappa variable 1-5	IGKV|IGKV15|L12|L12a|MGC22745|MGC32715|MGC88810|V1	2	BCR Signaling Pathway
IGKV1-6	28943	immunoglobulin kappa variable 1-6	IGKV16|L11	2	BCR Signaling Pathway
IGKV1-8	28942	immunoglobulin kappa variable	IGKV18|L9	2	BCR Signaling Pathway

		1-8			
IGKV1-9	28941	immunoglobulin kappa variable 1-9	IGKV19|L8	2	BCR Signaling Pathway
IGKV1D-12	28903	immunoglobulin kappa variable 1D-12	IGKV1D12|L19	2	BCR Signaling Pathway
IGKV1D-13	28902	immunoglobulin kappa variable 1D-13	IGKV1D13|L18	2	BCR Signaling Pathway
IGKV1D-16	28901	immunoglobulin kappa variable 1D-16	IGKV1D16|L15|L15a	2	BCR Signaling Pathway
IGKV1D-17	28900	immunoglobulin kappa variable 1D-17	IGKV1D17|L14	2	BCR Signaling Pathway
IGKV1D-33	28896	immunoglobulin kappa variable 1D-33	IGKV1D33|O8	2	BCR Signaling Pathway
IGKV1D-37	28894	immunoglobulin kappa variable 1D-37 (non-functional)	IGKV1D37|O4	2	BCR Signaling Pathway
IGKV1D-39	28893	immunoglobulin kappa variable 1D-39	IGKV1D39|O2	2	BCR Signaling Pathway
IGKV1D-42	28892	immunoglobulin kappa variable 1D-42 (non-functional)	IGKV1D42|L22	2	BCR Signaling Pathway
IGKV1D-43	28891	immunoglobulin kappa variable 1D-43	IGKV1D43|L23|L23a	2	BCR Signaling Pathway
IGKV1D-8	28904	immunoglobulin kappa variable 1D-8	IGKV1D8|L24|L24a	2	BCR Signaling Pathway
IGKV2-24	28923	immunoglobulin kappa variable 2-24	A23|IGKV224	2	BCR Signaling Pathway
IGKV2-28	28921	immunoglobulin kappa variable	A19|IGKV228	2	BCR Signaling Pathway

		2-28			
IGKV2-30	28919	immunoglobulin kappa variable 2-30	A17|IGKV230	2	BCR Signaling Pathway
IGKV2-40	28916	immunoglobulin kappa variable 2-40	IGKV240|O11|O11a	2	BCR Signaling Pathway
IGKV2D-24	28885	immunoglobulin kappa variable 2D-24 (non-functional)	A7|IGKV2D24	2	BCR Signaling Pathway
IGKV2D-28	28883	immunoglobulin kappa variable 2D-28	A3|IGKV2D28	2	BCR Signaling Pathway
IGKV2D-29	28882	immunoglobulin kappa variable 2D-29	A2a|A2c|IGKV2D29	2	BCR Signaling Pathway
IGKV2D-30	28881	immunoglobulin kappa variable 2D-30	A1|IGKV2D30	2	BCR Signaling Pathway
IGKV2D-40	28878	immunoglobulin kappa variable 2D-40	IGKV2D40|O1	2	BCR Signaling Pathway
IGKV3-11	28914	immunoglobulin kappa variable 3-11	IGKV311|L6	2	BCR Signaling Pathway
IGKV3-15	28913	immunoglobulin kappa variable 3-15	IGKV315|L2	2	BCR Signaling Pathway
IGKV3-20	28912	immunoglobulin kappa variable 3-20	13K18|A27|IGKV320	2	BCR Signaling Pathway
IGKV3-7	28915	immunoglobulin kappa variable 3-7 (non-functional)	IGKV37|L10|L10a|Vh	2	BCR Signaling Pathway
IGKV3D-11	28876	immunoglobulin kappa variable 3D-11	IGKV3D11|L20	2	BCR Signaling Pathway
IGKV3D-	28875	immunoglobulin kappa variable	IGKV3D15|L16|L16a|L16b|L16c	2	BCR Signaling Pathway

15		3D-15 (gene/pseudogene)			
IGKV3D-20	28874	immunoglobulin kappa variable 3D-20	A11|A11a|IGKV3D20	2	BCR Signaling Pathway
IGKV3D-7	28877	immunoglobulin kappa variable 3D-7	IGKV3D7|L25	2	BCR Signaling Pathway
IGKV4-1	28908	immunoglobulin kappa variable 4-1	B3|IGKV41	2	BCR Signaling Pathway
IGKV5-2	28907	immunoglobulin kappa variable 5-2	B2|IGKV52	2	BCR Signaling Pathway
IGKV6-21	28906	immunoglobulin kappa variable 6-21 (non-functional)	A26|IGKV621	2	BCR Signaling Pathway
IGKV6D-21	28870	immunoglobulin kappa variable 6D-21 (non-functional)	A10|IGKV6D21	2	BCR Signaling Pathway
IGKV6D-41	28869	immunoglobulin kappa variable 6D-41 (non-functional)	A14	2	BCR Signaling Pathway
IGL@	3535	immunoglobulin lambda locus	IGL|MGC88804	22	BCR Signaling Pathway
IGLC@	3536	immunoglobulin lambda constant group	IGLC	22	BCR Signaling Pathway
IGLC1	3537	immunoglobulin lambda constant 1 (Mcg marker)	IGLC	22	BCR Signaling Pathway
IGLC2	3538	immunoglobulin lambda constant 2	IGLC|MGC20392|MGC45681	22	BCR Signaling Pathway

		(Kern-Oz- marker)			
IGLC3	3539	immunoglobulin lambda constant 3 (Kern-Oz+ marker)	IGLC	22	BCR Signaling Pathway
IGLC6	3542	immunoglobulin lambda constant 6 (Kern+Oz- marker, gene/pseudogene)	IGLC	22	BCR Signaling Pathway
IGLC7	28834	immunoglobulin lambda constant 7	C7	22	BCR Signaling Pathway
IGLJ@	8217	immunoglobulin lambda joining group	IGLJ	22	BCR Signaling Pathway
IGLJ1	28833	immunoglobulin lambda joining 1	J1	22	BCR Signaling Pathway
IGLJ2	28832	immunoglobulin lambda joining 2	J2	22	BCR Signaling Pathway
IGLJ3	28831	immunoglobulin lambda joining 3	J3	22	BCR Signaling Pathway
IGLJ4	28830	immunoglobulin lambda joining 4 (non-functional)	-	22	BCR Signaling Pathway
IGLJ5	28829	immunoglobulin lambda joining 5 (non-functional)	-	22	BCR Signaling Pathway
IGLJ6	28828	immunoglobulin lambda joining 6	-	22	BCR Signaling Pathway
IGLJ7	28827	immunoglobulin lambda joining 7	J7	22	BCR Signaling Pathway

IGLV@	3546	immunoglobulin lambda variable group	IGLV	22	BCR Signaling Pathway
IGLV1-36	28826	immunoglobulin lambda variable 1-36	IGLV136|V1-11	22	BCR Signaling Pathway
IGLV1-40	28825	immunoglobulin lambda variable 1-40	IGLV140|V1-13	22	BCR Signaling Pathway
IGLV1-44	28823	immunoglobulin lambda variable 1-44	IGLV144|V1-16	22	BCR Signaling Pathway
IGLV1-47	28822	immunoglobulin lambda variable 1-47	IGLV147|V1-17	22	BCR Signaling Pathway
IGLV1-50	28821	immunoglobulin lambda variable 1-50 (non-functional)	IGLV150|V1-18	22	BCR Signaling Pathway
IGLV1-51	28820	immunoglobulin lambda variable 1-51	IGLV151|V1-19	22	BCR Signaling Pathway
IGLV10-54	28772	immunoglobulin lambda variable 10-54	IGLV1054|V1-20	22	BCR Signaling Pathway
IGLV11-55	28770	immunoglobulin lambda variable 11-55 (non-functional)	IGLV1155|V4-6	22	BCR Signaling Pathway
IGLV2-11	28816	immunoglobulin lambda variable 2-11	IGLV211|V1-3	22	BCR Signaling Pathway
IGLV2-14	28815	immunoglobulin lambda variable 2-14	IGLV214|V1-4	22	BCR Signaling Pathway
IGLV2-18	28814	immunoglobulin lambda variable 2-18	IGLV218|V1-5	22	BCR Signaling Pathway

IGLV2-23	28813	immunoglobulin lambda variable 2-23	IGLV223|V1-7	22	BCR Signaling Pathway
IGLV2-33	28811	immunoglobulin lambda variable 2-33 (non-functional)	IGLV233|V1-9	22	BCR Signaling Pathway
IGLV2-8	28817	immunoglobulin lambda variable 2-8	IGLV28|V1-2	22	BCR Signaling Pathway
IGLV3-1	28809	immunoglobulin lambda variable 3-1	IGLV31|V2-1	22	BCR Signaling Pathway
IGLV3-10	28803	immunoglobulin lambda variable 3-10	IGLV310|V2-7	22	BCR Signaling Pathway
IGLV3-12	28802	immunoglobulin lambda variable 3-12	IGLV312|V2-8	22	BCR Signaling Pathway
IGLV3-16	28799	immunoglobulin lambda variable 3-16	IGLV316|V2-11	22	BCR Signaling Pathway
IGLV3-19	28797	immunoglobulin lambda variable 3-19	IGLV319|V2-13|VL3L	22	BCR Signaling Pathway
IGLV3-21	28796	immunoglobulin lambda variable 3-21	IGLV321|V2-14	22	BCR Signaling Pathway
IGLV3-22	28795	immunoglobulin lambda variable 3-22 (gene/pseudogene)	IGLV322|V2-15	22	BCR Signaling Pathway
IGLV3-25	28793	immunoglobulin lambda variable 3-25	IGLV325|MGC105005|V2-17	22	BCR Signaling Pathway
IGLV3-27	28791	immunoglobulin lambda variable 3-27	IGLV327|V2-19	22	BCR Signaling Pathway
IGLV3-32	28787	immunoglobulin lambda variable 3-32 (non-functional)	IGLV332|V2-23P	22	BCR Signaling Pathway

IGLV3-9	28804	immunoglobulin lambda variable 3-9 (gene/pseudogene)	IGLV39|V2-6	22	BCR Signaling Pathway
IGLV4-3	28786	immunoglobulin lambda variable 4-3	IGLV43|V5-1	22	BCR Signaling Pathway
IGLV4-60	28785	immunoglobulin lambda variable 4-60	IGLV460|V5-4	22	BCR Signaling Pathway
IGLV4-69	28784	immunoglobulin lambda variable 4-69	IGLV469|V5-6	22	BCR Signaling Pathway
IGLV5-37	28783	immunoglobulin lambda variable 5-37	IGLV537|V4-1	22	BCR Signaling Pathway
IGLV5-39	28782	immunoglobulin lambda variable 5-39	IGLV539	22	BCR Signaling Pathway
IGLV5-45	28781	immunoglobulin lambda variable 5-45	IGLV545|V4-2	22	BCR Signaling Pathway
IGLV5-48	28780	immunoglobulin lambda variable 5-48 (non-functional)	IGLV548|V4-3	22	BCR Signaling Pathway
IGLV5-52	28779	immunoglobulin lambda variable 5-52	IGLV552|V4-4	22	BCR Signaling Pathway
IGLV6-57	28778	immunoglobulin lambda variable 6-57	IGLV657|MGC34845|V1-22	22	BCR Signaling Pathway
IGLV7-43	28776	immunoglobulin lambda variable 7-43	IGLV743|V3-2	22	BCR Signaling Pathway
IGLV7-46	28775	immunoglobulin lambda variable 7-46 (gene/pseudogene)	IGLV746|V3-3	22	BCR Signaling Pathway
IGLV8-61	28774	immunoglobulin lambda variable 8-61	IGLV861|V3-4	22	BCR Signaling Pathway

IGLV9-49	28773	immunoglobulin lambda variable 9-49	IGLV949|V5-2	22	BCR Signaling Pathway
C3	718	complement component 3	ARMD9|ASP|CPAMD1	19	Chemokines
C5	727	complement component 5	CPAMD4|FLJ17816|FLJ17822|MGC142298	9	Chemokines
CAMP	820	cathelicidin antimicrobial peptide	CAP18|CRAMP|FALL-39|FALL39|HSD26|LL37	3	Chemokines
CCL1	6346	chemokine (C-C motif) ligand 1	I-309|P500|SCYA1|SISe|TCA3	17	Chemokines
CCL11	6356	chemokine (C-C motif) ligand 11	MGC22554|SCYA11	17	Chemokines
CCL13	6357	chemokine (C-C motif) ligand 13	CKb10|MCP-4|MGC17134|NCC-1|NCC1|SCYA13|SCYL1	17	Chemokines
CCL14	6358	chemokine (C-C motif) ligand 14	CC-1|CC-3|CKb1|FLJ16015|HCC-1|HCC-3|MCIF|NCC-2|NCC2|SCYA14|SCYL2|SY14	17	Chemokines
CCL14-CCL15	348249	chemokine ligand 14, chemokine ligand 15 transcription unit	-	17	Chemokines
CCL15	6359	chemokine (C-C motif) ligand 15	HCC-2|HMRP-2B|LKN1|Lkn-1|MIP-1d|MIP-5|NCC-3|NCC3|SCYA15|SCYL3|SY15	17	Chemokines
CCL16	6360	chemokine (C-C motif) ligand 16	CKb12|HCC-4|ILINCK|LCC-1|LEC|LMC|MGC117051|Mtn-1|NCC-4|NCC4|SCYA16|SCYL4	17	Chemokines
CCL17	6361	chemokine (C-C motif) ligand 17	A-152E5.3|ABCD-2|MGC138271|MGC138273|SCYA17|TARC	16	Chemokines
CCL18	6362	chemokine (C-C motif) ligand 18 (pulmonary and activation-regulated)	AMAC-1|AMAC1|CKb7|DC-CK1|DCCK1|MIP-4|PARC|SCYA18	17	Chemokines
CCL19	6363	chemokine (C-C motif) ligand 19	CKb11|ELC|MGC34433|MIP-3b|MIP3B|SCYA19	9	Chemokines
CCL2	6347	chemokine (C-C motif) ligand 2	GDCF-2|HC11|HSMCR30|MCAF|MCP-	17	Chemokines

			1|MCP1|MGC9434|SCYA2|SMC-CF		
CCL20	6364	chemokine (C-C motif) ligand 20	CKb4|LARC|MIP-3a|MIP3A|SCYA20|ST38	2	Chemokines
CCL21	6366	chemokine (C-C motif) ligand 21	6Ckine|CKb9|ECL|MGC34555|SCYA21|SLC|TCA4	9	Chemokines
CCL22	6367	chemokine (C-C motif) ligand 22	A-152E5.1|ABCD-1|DC/B-CK|MDC|MGC34554|SCYA22|STCP-1	16	Chemokines
CCL23	6368	chemokine (C-C motif) ligand 23	CK-BETA-8|CKb8|Ckb-8|Ckb-8-1|MIP-3|MIP3|MPIF-1|SCYA23	17	Chemokines
CCL24	6369	chemokine (C-C motif) ligand 24	Ckb-6|MPIF-2|MPIF2|SCYA24	7	Chemokines
CCL25	6370	chemokine (C-C motif) ligand 25	Ckb15|MGC150327|SCYA25|TECK	19	Chemokines
CCL26	10344	chemokine (C-C motif) ligand 26	IMAC|MGC126714|MIP-4a|MIP-4alpha|SCYA26|TSC-1	7	Chemokines
CCL27	10850	chemokine (C-C motif) ligand 27	ALP|CTACK|CTAK|ESKINE|ILC|PESKY|SCYA27	9	Chemokines
CCL28	56477	chemokine (C-C motif) ligand 28	CCK1|MEC|MGC71902|SCYA28	5	Chemokines
CCL3	6348	chemokine (C-C motif) ligand 3	G0S19-1|LD78ALPHA|MIP-1-alpha|MIP1A|SCYA3	17	Chemokines
CCL3L1	6349	chemokine (C-C motif) ligand 3-like 1	464.2|D17S1718|G0S19-2|LD78|LD78BETA|MGC104178|MGC12815|MGC182017|MIP1AP|SCYA3L|SCYA3L1	17	Chemokines
CCL3L2	390788	chemokine (C-C motif) ligand 3-like 2	G0S19-3|LD78gamma|SCYA3L2	17	Chemokines
CCL3L3	414062	chemokine (C-C motif) ligand 3-like 3	464.2|D17S1718|LD78|LD78BETA|MGC12815|SCYA3L|SCYA3L1	17	Chemokines
CCL4	6351	chemokine (C-C motif) ligand 4	ACT2|AT744.1|G-26|LAG1|MGC104418|MGC126025|MGC12602	17	Chemokines

			6|MIP-1-beta|MIP1B|MIP1B1|SCYA2|SCYA4		
CCL4L1	9560	chemokine (C-C motif) ligand 4-like 1	AT744.2|CCL4L|LAG-1|LAG1|SCYA4L	17	Chemokines
CCL4L2	388372	chemokine (C-C motif) ligand 4-like 2	AT744.2|CCL4L|SCYA4L	17	Chemokines
CCL5	6352	chemokine (C-C motif) ligand 5	D17S136E|MGC17164|RANTES|SCYA5|SISd|TCP228	17	Chemokines
CCL7	6354	chemokine (C-C motif) ligand 7	FIC|MARC|MCP-3|MCP3|MGC138463|MGC138465|NC28|SCYA6|SCYA7	17	Chemokines
CCL8	6355	chemokine (C-C motif) ligand 8	HC14|MCP-2|MCP2|SCYA10|SCYA8	17	Chemokines
CKLF	51192	chemokine-like factor	C32|CKLF1|CKLF2|CKLF3|CKLF4|HSPC224|UCK-1	16	Chemokines
CMA1	1215	chymase 1, mast cell	CYH|MCT1|MGC119890|MGC119891|chymase	14	Chemokines
CTSG	1511	cathepsin G	CG|MGC23078	14	Chemokines
CX3CL1	6376	chemokine (C-X3-C motif) ligand 1	ABCD-3|C3Xkine|CXC3|CXC3C|NTN|NTT|SCYD1|fractalkine|neurotactin	16	Chemokines
CXCL1	2919	chemokine (C-X-C motif) ligand 1 (melanoma growth stimulating activity, alpha)	FSP|GRO1|GROa|MGSA|MGSA-a|NAP-3|SCYB1	4	Chemokines
CXCL10	3627	chemokine (C-X-C motif) ligand 10	C7|IFI10|INP10|IP-10|SCYB10|crg-2|gIP-10|mob-1	4	Chemokines
CXCL11	6373	chemokine (C-X-C motif) ligand 11	H174|I-TAC|IP-9|IP9|MGC102770|SCYB11|SCYB9B|b-R1	4	Chemokines

CXCL12	6387	chemokine (C-X-C motif) ligand 12 (stromal cell-derived factor 1)	PBSF|SCYB12|SDF-1a|SDF-1b|SDF1|SDF1A|SDF1B|TLSF-a|TLSF-b|TPAR1	10	Chemokines
CXCL13	10563	chemokine (C-X-C motif) ligand 13	ANGIE|ANGIE2|BCA-1|BCA1|BLC|BLR1L|SCYB13	4	Chemokines
CXCL14	9547	chemokine (C-X-C motif) ligand 14	BMAC|BRAK|KS1|Kec|MGC10687|MIP-2g|NJAC|SCYB14|bolekine	5	Chemokines
CXCL16	58191	chemokine (C-X-C motif) ligand 16	CXCLG16|SR-PSOX|SRPSOX	17	Chemokines
CXCL17	284340	chemokine (C-X-C motif) ligand 17	DMC|Dcip1|MGC138300|UNQ473|VCC-1|VCC1	19	Chemokines
CXCL2	2920	chemokine (C-X-C motif) ligand 2	CINC-2a|GRO2|GROb|MGSA-b|MIP-2a|MIP2|MIP2A|SCYB2	4	Chemokines
CXCL3	2921	chemokine (C-X-C motif) ligand 3	CINC-2b|GRO3|GROg|MIP-2b|MIP2B|SCYB3	4	Chemokines
CXCL5	6374	chemokine (C-X-C motif) ligand 5	ENA-78|SCYB5	4	Chemokines
CXCL6	6372	chemokine (C-X-C motif) ligand 6 (granulocyte chemotactic protein 2)	CKA-3|GCP-2|GCP2|SCYB6	4	Chemokines
CXCL9	4283	chemokine (C-X-C motif) ligand 9	CMK|Humig|MIG|SCYB9|crg-10	4	Chemokines
CYR61	3491	cysteine-rich, angiogenic inducer, 61	CCN1|GIG1|IGFBP10	1	Chemokines
DEFA1	1667	defensin, alpha 1	DEF1|DEFA2|HNP-1|HP-1|MGC138393|MRS	8	Chemokines
DEFA3	1668	defensin, alpha 3, neutrophil-specific	DEF3|HNP-3|HNP3|HP-3	8	Chemokines

DEFA5	1670	defensin, alpha 5, Paneth cell-specific	DEF5|HD-5|MGC129728	8	Chemokines
DEFB1	1672	defensin, beta 1	BD1|DEFB-1|DEFB101|HBD1|MGC51822	8	Chemokines
DEFB103A	55894	defensin, beta 103A	DEFB103|DEFB3|HBD-3|HBD3|HBP-3|HBP3	8	Chemokines
DEFB104A	140596	defensin, beta 104A	BD-4|DEFB-4|DEFB104|DEFB4|MGC118942|MGC118944|MGC118945|hBD-4	8	Chemokines
DEFB4	1673	defensin, beta 4	DEFB-2|DEFB102|DEFB2|HBD-2|SAP1	8	Chemokines
EDN1	1906	endothelin 1	ET1|HDLCQ7	6	Chemokines
EDN2	1907	endothelin 2	ET2|PPET2	1	Chemokines
EDN3	1908	endothelin 3	ET3|MGC15067|MGC61498	20	Chemokines
FGF10	2255	fibroblast growth factor 10	-	5	Chemokines
FGF2	2247	fibroblast growth factor 2 (basic)	BFGF|FGFB|HBGF-2	4	Chemokines
HTN3	3347	histatin 3	HIS2|HTN2|HTN5	4	Chemokines
IL8	3576	interleukin 8	CXCL8|GCP-1|GCP1|LECT|LUCT|LYNAP|MDNCF|MONAP|NAF|NAP-1|NAP1	4	Chemokines
LECT2	3950	leukocyte cell-derived chemotaxin 2	MGC126628|chm-II|chm2	5	Chemokines
PF4	5196	platelet factor 4	CXCL4|MGC138298|SCYB4	4	Chemokines
PF4V1	5197	platelet factor 4 variant 1	CXCL4L1|CXCL4V1|PF4-ALT|PF4A|SCYB4V1	4	Chemokines
PLAU	5328	plasminogen activator, urokinase	ATF|UPA|URK|u-PA	10	Chemokines
PPBP	5473	pro-platelet basic protein (chemokine (C-X-C motif) ligand 7)	B-TG1|Beta-TG|CTAP-III|CTAP3|CTAPIII|CXCL7|LA-PF4|LDGF|MDGF|NAP-	4	Chemokines

			2|PBP|SCYB7|TC1|TC2|TGB|TGB1|THBGB|THBGB1		
PPBPL1	728045	pro-platelet basic protein-like 1	TGB2	4	Chemokines
PROK2	60675	prokineticin 2	BV8|KAL4|MIT1|PK2	3	Chemokines
RNASE2	6036	ribonuclease, RNase A family, 2 (liver, eosinophil-derived neurotoxin)	EDN|RNS2	14	Chemokines
SAA1	6288	serum amyloid A1	MGC111216|PIG4|SAA|TP53I4	11	Chemokines
SAA2	6289	serum amyloid A2	-	11	Chemokines
SBDS	51119	Shwachman-Bodian-Diamond syndrome	CGI-97|FLJ10917|SDS|SWDS	7	Chemokines
SEMA3A	10371	sema domain, immunoglobulin domain (Ig), short basic domain, secreted, (semaphorin) 3A	Hsema-I|Hsema-III|MGC133243|SEMA1|SEMAD|SEMAIII|SEMAL|SemD|coll-1	7	Chemokines
SEMA3B	7869	sema domain, immunoglobulin domain (Ig), short basic domain, secreted, (semaphorin) 3B	FLJ34863|LUCA-1|SEMA5|SEMAA|SemA|semaV	3	Chemokines
SEMA3C	10512	sema domain, immunoglobulin domain (Ig), short basic domain, secreted, (semaphorin) 3C	SEMAE|SemE	7	Chemokines
SEMA3D	223117	sema domain, immunoglobulin domain (Ig), short basic domain, secreted, (semaphorin) 3D	MGC39708|Sema-Z2|coll-2	7	Chemokines
SEMA3E	9723	sema domain, immunoglobulin domain (Ig), short basic domain, secreted, (semaphorin) 3E	KIAA0331|M-SEMAH|M-SemaK|SEMAH|coll-5	7	Chemokines

SEMA3F	6405	sema domain, immunoglobulin domain (Ig), short basic domain, secreted, (semaphorin) 3F	SEMA-IV|SEMA4|SEMAK	3	Chemokines
SEMA3G	56920	sema domain, immunoglobulin domain (Ig), short basic domain, secreted, (semaphorin) 3G	FLJ00014|MGC119473|sem2	3	Chemokines
SEMA4A	64218	sema domain, immunoglobulin domain (Ig), transmembrane domain (TM) and short cytoplasmic domain, (semaphorin) 4A	CORD10|FLJ12287|RP35|SEMAB|SEMB	1	Chemokines
SEMA4B	10509	sema domain, immunoglobulin domain (Ig), transmembrane domain (TM) and short cytoplasmic domain, (semaphorin) 4B	KIAA1745|MGC131831|SEMAC|SemC	15	Chemokines
SEMA4C	54910	sema domain, immunoglobulin domain (Ig), transmembrane domain (TM) and short cytoplasmic domain, (semaphorin) 4C	FLJ20369|KIAA1739|M-SEMA-F|MGC126382|MGC126383|SEMACL1|SEMAF|SEMAI	2	Chemokines
SEMA4D	10507	sema domain, immunoglobulin domain (Ig), transmembrane domain (TM) and short cytoplasmic domain, (semaphorin) 4D	C9orf164|CD100|FLJ33485|FLJ34282|FLJ39737|FLJ46484|M-sema-G|MGC169138|MGC169141|SEMAJ|coll-4	9	Chemokines

SEMA4F	10505	sema domain, immunoglobulin domain (Ig), transmembrane domain (TM) and short cytoplasmic domain, (semaphorin) 4F	M-SEMA|PRO2353|SEMAM|SEMAW|m-Sema-M	2	Chemokines
SEMA4G	57715	sema domain, immunoglobulin domain (Ig), transmembrane domain (TM) and short cytoplasmic domain, (semaphorin) 4G	FLJ20590|KIAA1619|MGC102867	10	Chemokines
SEMA5A	9037	sema domain, seven thrombospondin repeats (type 1 and type 1-like), transmembrane domain (TM) and short cytoplasmic domain, (semaphorin) 5A	FLJ12815|SEMAF|semF	5	Chemokines
SEMA5B	54437	sema domain, seven thrombospondin repeats (type 1 and type 1-like), transmembrane domain (TM) and short cytoplasmic domain, (semaphorin) 5B	FLJ10372|KIAA1445|SEMAG|SemG	3	Chemokines
SEMA6A	57556	sema domain, transmembrane domain (TM), and cytoplasmic domain, (semaphorin) 6A	HT018|KIAA1368|SEMA|SEMA6A1|SEMAQ|VIA	5	Chemokines

SEMA6B	10501	sema domain, transmembrane domain (TM), and cytoplasmic domain, (semaphorin) 6B	SEM-SEMA-Y|SEMA-VIB|SEMAN|semaZ	19	Chemokines
SEMA6C	10500	sema domain, transmembrane domain (TM), and cytoplasmic domain, (semaphorin) 6C	SEMAY|m-SemaY|m-SemaY2	1	Chemokines
SEMA6D	80031	sema domain, transmembrane domain (TM), and cytoplasmic domain, (semaphorin) 6D	FLJ11598|KIAA1479	15	Chemokines
SEMA7A	8482	semaphorin 7A, GPI membrane anchor (John Milton Hagen blood group)	CD108|CDw108|H-SEMA-K1|H-Sema-L|JMH|MGC126692|MGC126696|SEMAK1|SEMAL	15	Chemokines
SLIT1	6585	slit homolog 1 (Drosophila)	MEGF4|MGC164811|SLIL1|SLIT3|Slit-1	10	Chemokines
SLIT2	9353	slit homolog 2 (Drosophila)	FLJ14420|SLIL3|Slit-2	4	Chemokines
TNC	3371	tenascin C	HXB|MGC167029|TN	9	Chemokines
TYMP	1890	thymidine phosphorylase	ECGF1|MNGIE|PDECGF|TP|hPD-ECGF	22	Chemokines
XCL1	6375	chemokine (C motif) ligand 1	ATAC|LPTN|LTN|SCM-1|SCM-1a|SCM1|SCYC1	1	Chemokines
XCL2	6846	chemokine (C motif) ligand 2	SCM-1b|SCM1B|SCYC2	1	Chemokines
C5AR1	728	complement component 5a receptor 1	C5A|C5AR|C5R1|CD88	19	Chemokine_Receptors
CCBP2	1238	chemokine binding protein 2	CCR10|CCR9|CMKBR9|D6|MGC126678|MGC138250|hD6	3	Chemokine_Receptors
CCR1	1230	chemokine (C-C motif) receptor 1	CD191|CKR-1|CKR1|CMKBR1|HM145|MIP1aR|SCYAR1	3	Chemokine_Receptors
CCR10	2826	chemokine (C-C motif) receptor 10	GPR2	17	Chemokine_Receptors

CCR3	1232	chemokine (C-C motif) receptor 3	CC-CKR-3|CD193|CKR3|CMKBR3|MGC102841	3	Chemokine_Receptors
CCR4	1233	chemokine (C-C motif) receptor 4	CC-CKR-4|CD194|CKR4|CMKBR4|ChemR13|HGCN:14099|K5-5|MGC88293	3	Chemokine_Receptors
CCR5	1234	chemokine (C-C motif) receptor 5	CC-CKR-5|CCCKR5|CD195|CKR-5|CKR5|CMKBR5|FLJ78003|IDDM22	3	Chemokine_Receptors
CCR6	1235	chemokine (C-C motif) receptor 6	BN-1|CD196|CKR-L3|CKR6|CKRL3|CMKBR6|DCR2|DRY-6|GPR-CY4|GPR29|GPRCY4|STRL22	6	Chemokine_Receptors
CCR7	1236	chemokine (C-C motif) receptor 7	BLR2|CD197|CDw197|CMKBR7|EBI1	17	Chemokine_Receptors
CCR8	1237	chemokine (C-C motif) receptor 8	CDw198|CKR-L1|CKRL1|CMKBR8|CMKBRL2|CY6|GPR-CY6|MGC129966|MGC129973|TER1	3	Chemokine_Receptors
CCR9	10803	chemokine (C-C motif) receptor 9	CDw199|GPR-9-6|GPR28	3	Chemokine_Receptors
CCRL1	51554	chemokine (C-C motif) receptor-like 1	CC-CKR-11|CCBP2|CCR10|CCR11|CCX-CKR|CKR-11|PPR1|VSHK1	3	Chemokine_Receptors
CCRL2	9034	chemokine (C-C motif) receptor-like 2	CKRX|CRAM-A|CRAM-B|FLJ55815|HCR|MGC116710|MGC34104	3	Chemokine_Receptors
CMKLR1	1240	chemokine-like receptor 1	CHEMERINR|ChemR23|DEZ|MGC126105|MGC126106	12	Chemokine_Receptors
CX3CR1	1524	chemokine (C-X3-C motif) receptor 1	CCRL1|CMKBRL1|CMKDR1|GPR13|GPRV28|V28	3	Chemokine_Receptors

CXCR3	2833	chemokine (C-X-C motif) receptor 3	CD182|CD183|CKR-L2|CMKAR3|GPR9|IP10-R|Mig-R|MigR	X	Chemokine_Receptors
CXCR4	7852	chemokine (C-X-C motif) receptor 4	CD184|D2S201E|FB22|HM89|HSY3RR|LAP3|LCR1|LESTR|NPY3R|NPYR|NPYRL|NPYY3R|WHIM	2	Chemokine_Receptors
CXCR5	643	chemokine (C-X-C motif) receptor 5	BLR1|CD185|MDR15|MGC117347	11	Chemokine_Receptors
CXCR6	10663	chemokine (C-X-C motif) receptor 6	BONZO|CD186|STRL33|TYMSTR	3	Chemokine_Receptors
CXCR7	57007	chemokine (C-X-C motif) receptor 7	CMKOR1|GPR159|RDC1	2	Chemokine_Receptors
CYSLTR1	10800	cysteinyl leukotriene receptor 1	CYSLT1|CYSLT1R|CYSLTR|HG55|HMTMF81|MGC46139	X	Chemokine_Receptors
CYSLTR2	57105	cysteinyl leukotriene receptor 2	CYSLT2|CYSLT2R|GPCR|HG57|HPN321|KPG_011|PSEC0146|hGPCR21	13	Chemokine_Receptors
DARC	2532	Duffy blood group, chemokine receptor	CCBP1|CD234|Dfy|FY|GPD|GpFy|WBCQ1	1	Chemokine_Receptors
EDNRA	1909	endothelin receptor type A	ETA|ETRA	4	Chemokine_Receptors
EDNRB	1910	endothelin receptor type B	ABCDS|ETB|ETBR|ETRB|HSCR|HSCR2	13	Chemokine_Receptors
FPR1	2357	formyl peptide receptor 1	FMLP|FPR	19	Chemokine_Receptors
FPR2	2358	formyl peptide receptor 2	ALXR|FMLP-R-II|FMLPX|FPR2A|FPRH1|FPRH2|FPRL1|HM63|LXA4R	19	Chemokine_Receptors
FPR2	2358	formyl peptide receptor 2	ALXR|FMLP-R-II|FMLPX|FPR2A|FPRH1|FPRH2|FPRL1|HM63|LXA4R	19	Chemokine_Receptors

GPR17	2840	G protein-coupled receptor 17	DKFZp686M18273	2	Chemokine_Receptors
GPR32	2854	G protein-coupled receptor 32	-	19	Chemokine_Receptors
GPR33	2856	G protein-coupled receptor 33 (gene/pseudogene)	-	14	Chemokine_Receptors
GPR44	11251	G protein-coupled receptor 44	CD294|CRTH2|DP2	11	Chemokine_Receptors
GPR77	27202	G protein-coupled receptor 77	C5L2|GPF77	19	Chemokine_Receptors
IL8RA	3577	interleukin 8 receptor, alpha	C-C|C-C-CKR-1|CD128|CD181|CDw128a|CKR-1|CMKAR1|CXCR1|IL8R1|IL8RBA	2	Chemokine_Receptors
IL8RB	3579	interleukin 8 receptor, beta	CD182|CDw128b|CMKAR2|CXCR2|IL8R2|IL8RA	2	Chemokine_Receptors
LTB4R	1241	leukotriene B4 receptor	BLT1|BLTR|CMKRL1|GPR16|LTB4R1|LTBR1|P2RY7|P2Y7	14	Chemokine_Receptors
LTB4R2	56413	leukotriene B4 receptor 2	BLT2|BLTR2|JULF2|KPG_004|NOP9	14	Chemokine_Receptors
PLAUR	5329	plasminogen activator, urokinase receptor	CD87|UPAR|URKR	19	Chemokine_Receptors
PLXNA1	5361	plexin A1	NOV|NOVP|PLEXIN-A1|PLXN1	3	Chemokine_Receptors
PLXNA2	5362	plexin A2	FLJ11751|FLJ30634|KIAA0463|OCT|PLXN2	1	Chemokine_Receptors
PLXNA3	55558	plexin A3	6.3|HSSEXGENE|PLEXIN-A3|PLXN3|PLXN4|SEX|XAP-6	X	Chemokine_Receptors
PLXNA4	91584	plexin A4	DKFZp434G0625|DKFZp566O0546|FAYV2820|FLJ35026|FLJ38287|KIAA1550|PLEXA4|PLXNA4A|PLXNA4B|PRO34003	7	Chemokine_Receptors
PLXNB1	5364	plexin B1	KIAA0407|MGC149167|PLEXIN-B1|PLXN5|SEP	3	Chemokine_Receptors
PLXNB2	23654	plexin B2	KIAA0315|MM1|Nbla00445|PLEXB2|dJ402G11.3	22	Chemokine_Receptors
PLXNB3	5365	plexin B3	FLJ76953|PLEXB3|PLEXR|PLXN6	X	Chemokine_Receptors

PLXNC1	10154	plexin C1	CD232|PLXN-C1|VESPR	12	Chemokine_Receptors
PLXND1	23129	plexin D1	KIAA0620|MGC75353|PLEXD1	3	Chemokine_Receptors
PTAFR	5724	platelet-activating factor receptor	PAFR	1	Chemokine_Receptors
ROBO1	6091	roundabout, axon guidance receptor, homolog 1 (Drosophila)	DUTT1|FLJ21882|MGC131599|MGC133277|SAX3	3	Chemokine_Receptors
ROBO2	6092	roundabout, axon guidance receptor, homolog 2 (Drosophila)	KIAA1568|SAX3	3	Chemokine_Receptors
ROBO3	64221	roundabout, axon guidance receptor, homolog 3 (Drosophila)	FLJ21044|HGPPS|HGPS|RBIG1|RIG1	11	Chemokine_Receptors
RXFP3	51289	relaxin/insulin-like family peptide receptor 3	GPCR135|MGC141998|MGC142000|RLN3R1|RXFPR3|SALPR	5	Chemokine_Receptors
XCR1	2829	chemokine (C motif) receptor 1	CCXCR1|GPR5	3	Chemokine_Receptors
ADIPOQ	9370	adiponectin, C1Q and collagen domain containing	ACDC|ACRP30|ADIPQTL1|ADPN|APM-1|APM1|GBP28|adiponectin	3	Cytokines
ADM	133	adrenomedullin	AM	11	Cytokines
ADM2	79924	adrenomedullin 2	AM2|FLJ21135|dJ579N16.4	22	Cytokines
AGRP	181	agouti related protein homolog (mouse)	AGRT|ART|ASIP2|MGC118963	16	Cytokines
AGT	183	angiotensinogen (serpin peptidase inhibitor, clade A, member 8)	ANHU|FLJ92595|FLJ97926|SERPINA8	1	Cytokines
AMBN	258	ameloblastin (enamel matrix protein)	-	4	Cytokines

AMELX	265	amelogenin (amelogenesis imperfecta 1, X-linked)	AIH1|ALGN|AMG|AMGL|AMGX	X	Cytokines
AMH	268	anti-Mullerian hormone	MIF|MIS	19	Cytokines
ANGPTL5	253935	angiopoietin-like 5	-	11	Cytokines
ANGPTL7	10218	angiopoietin-like 7	AngX|CDT6|RP4-647M16.2|dJ647M16.1	1	Cytokines
APLN	8862	apelin	XNPEP2	X	Cytokines
AREG	374	amphiregulin	AR|CRDGF|MGC13647|SDGF	4	Cytokines
ARMET	7873	arginine-rich, mutated in early stage tumors	ARP|MANF|MGC142148|MGC142150	3	Cytokines
ARMETL1	441549	arginine-rich, mutated in early stage tumors-like 1	cdnf	10	Cytokines
ARTN	9048	artemin	ENOVIN|EVN|NBN	1	Cytokines
AVP	551	arginine vasopressin	ADH|ARVP|AVP-NPII|AVRP|VP	20	Cytokines
AZU1	566	azurocidin 1	AZAMP|AZU|CAP37|HBP|HUMAZUR|NAZC	19	Cytokines
BDNF	627	brain-derived neurotrophic factor	MGC34632	11	Cytokines
BMP1	649	bone morphogenetic protein 1	FLJ44432|PCOLC|PCP|TLD|pCP-2	8	Cytokines
BMP10	27302	bone morphogenetic protein 10	MGC126783	2	Cytokines
BMP15	9210	bone morphogenetic protein 15	GDF9B|ODG2|POF4	X	Cytokines
BMP2	650	bone morphogenetic protein 2	BMP2A	20	Cytokines
BMP3	651	bone morphogenetic protein 3	BMP-3A	4	Cytokines
BMP4	652	bone morphogenetic protein 4	BMP2B|BMP2B1|MCOPS6|OFC11|ZYME	14	Cytokines
BMP5	653	bone morphogenetic protein 5	MGC34244	6	Cytokines
BMP6	654	bone morphogenetic protein 6	VGR|VGR1	6	Cytokines
BMP7	655	bone morphogenetic protein 7	OP-1	20	Cytokines
BMP8A	353500	bone morphogenetic protein 8a	FLJ14351|FLJ45264	1	Cytokines
BMP8B	656	bone morphogenetic protein 8b	BMP8|MGC131757|OP2	1	Cytokines

BTC	685	betacellulin	-	4	Cytokines
C19orf10	56005	chromosome 19 open reading frame 10	EUROIMAGE1875335|IL25|IL27|IL27w|R33729_1|SF20	19	Cytokines
C3	718	complement component 3	ARMD9|ASP|CPAMD1	19	Cytokines
C5	727	complement component 5	CPAMD4|FLJ17816|FLJ17822|MGC142298	9	Cytokines
CALCA	796	calcitonin-related polypeptide alpha	CALC1|CGRP|CGRP-I|CGRP1|CT|KC|MGC126648	11	Cytokines
CALCB	797	calcitonin-related polypeptide beta	CALC2|CGRP-II|CGRP2|FLJ30166	11	Cytokines
CAMP	820	cathelicidin antimicrobial peptide	CAP18|CRAMP|FALL-39|FALL39|HSD26|LL37	3	Cytokines
CAT	847	catalase	MGC138422|MGC138424	11	Cytokines
CCK	885	cholecystokinin	MGC117187	3	Cytokines
CCL1	6346	chemokine (C-C motif) ligand 1	I-309|P500|SCYA1|SISe|TCA3	17	Cytokines
CCL11	6356	chemokine (C-C motif) ligand 11	MGC22554|SCYA11	17	Cytokines
CCL13	6357	chemokine (C-C motif) ligand 13	CKb10|MCP-4|MGC17134|NCC-1|NCC1|SCYA13|SCYL1	17	Cytokines
CCL14	6358	chemokine (C-C motif) ligand 14	CC-1|CC-3|CKb1|FLJ16015|HCC-1|HCC-3|MCIF|NCC-2|NCC2|SCYA14|SCYL2|SY14	17	Cytokines
CCL14-CCL15	348249	chemokine ligand 14, chemokine ligand 15 transcription unit	-	17	Cytokines
CCL15	6359	chemokine (C-C motif) ligand 15	HCC-2|HMRP-2B|LKN1|Lkn-1|MIP-1d|MIP-5|NCC-3|NCC3|SCYA15|SCYL3|SY15	17	Cytokines
CCL16	6360	chemokine (C-C motif) ligand 16	CKb12|HCC-4|ILINCK|LCC-1|LEC|LMC|MGC117051|Mtn-1|NCC-4|NCC4|SCYA16|SCYL4	17	Cytokines

CCL17	6361	chemokine (C-C motif) ligand 17	A-152E5.3|ABCD-2|MGC138271|MGC138273|SCYA17|TARC	16	Cytokines
CCL18	6362	chemokine (C-C motif) ligand 18 (pulmonary and activation-regulated)	AMAC-1|AMAC1|CKb7|DC-CK1|DCCK1|MIP-4|PARC|SCYA18	17	Cytokines
CCL19	6363	chemokine (C-C motif) ligand 19	CKb11|ELC|MGC34433|MIP-3b|MIP3B|SCYA19	9	Cytokines
CCL2	6347	chemokine (C-C motif) ligand 2	GDCF-2|HC11|HSMCR30|MCAF|MCP-1|MCP1|MGC9434|SCYA2|SMC-CF	17	Cytokines
CCL20	6364	chemokine (C-C motif) ligand 20	CKb4|LARC|MIP-3a|MIP3A|SCYA20|ST38	2	Cytokines
CCL21	6366	chemokine (C-C motif) ligand 21	6Ckine|CKb9|ECL|MGC34555|SCYA21|SLC|TCA4	9	Cytokines
CCL22	6367	chemokine (C-C motif) ligand 22	A-152E5.1|ABCD-1|DC/B-CK|MDC|MGC34554|SCYA22|STCP-1	16	Cytokines
CCL23	6368	chemokine (C-C motif) ligand 23	CK-BETA-8|CKb8|Ckb-8|Ckb-8-1|MIP-3|MIP3|MPIF-1|SCYA23	17	Cytokines
CCL24	6369	chemokine (C-C motif) ligand 24	Ckb-6|MPIF-2|MPIF2|SCYA24	7	Cytokines
CCL25	6370	chemokine (C-C motif) ligand 25	Ckb15|MGC150327|SCYA25|TECK	19	Cytokines
CCL26	10344	chemokine (C-C motif) ligand 26	IMAC|MGC126714|MIP-4a|MIP-4alpha|SCYA26|TSC-1	7	Cytokines
CCL27	10850	chemokine (C-C motif) ligand 27	ALP|CTACK|CTAK|ESKINE|ILC|PESKY|SCYA27	9	Cytokines
CCL28	56477	chemokine (C-C motif) ligand 28	CCK1|MEC|MGC71902|SCYA28	5	Cytokines
CCL3	6348	chemokine (C-C motif) ligand 3	G0S19-1|LD78ALPHA|MIP-1-alpha|MIP1A|SCYA3	17	Cytokines
CCL3L1	6349	chemokine (C-C motif) ligand 3-like 1	464.2|D17S1718|G0S19-2|LD78|LD78BETA|MGC104178|MGC12815|M	17	Cytokines

			GC182017|MIP1AP|SCYA3L|SCYA3L1		
CCL3L2	390788	chemokine (C-C motif) ligand 3-like 2	G0S19-3|LD78gamma|SCYA3L2	17	Cytokines
CCL3L3	414062	chemokine (C-C motif) ligand 3-like 3	464.2|D17S1718|LD78|LD78BETA|MGC12815|SCYA3L|SCYA3L1	17	Cytokines
CCL4	6351	chemokine (C-C motif) ligand 4	ACT2|AT744.1|G-26|LAG1|MGC104418|MGC126025|MGC126026|MIP-1-beta|MIP1B|MIP1B1|SCYA2|SCYA4	17	Cytokines
CCL4L1	9560	chemokine (C-C motif) ligand 4-like 1	AT744.2|CCL4L|LAG-1|LAG1|SCYA4L	17	Cytokines
CCL4L2	388372	chemokine (C-C motif) ligand 4-like 2	AT744.2|CCL4L|SCYA4L	17	Cytokines
CCL5	6352	chemokine (C-C motif) ligand 5	D17S136E|MGC17164|RANTES|SCYA5|SISd|TCP228	17	Cytokines
CCL7	6354	chemokine (C-C motif) ligand 7	FIC|MARC|MCP-3|MCP3|MGC138463|MGC138465|NC28|SCYA6|SCYA7	17	Cytokines
CCL8	6355	chemokine (C-C motif) ligand 8	HC14|MCP-2|MCP2|SCYA10|SCYA8	17	Cytokines
CD320	51293	CD320 molecule	8D6|8D6A	19	Cytokines
CD40LG	959	CD40 ligand	CD154|CD40L|HIGM1|IGM|IMD3|T-BAM|TNFSF5|TRAP|gp39|hCD40L	X	Cytokines
CD70	970	CD70 molecule	CD27L|CD27LG|TNFSF7	19	Cytokines
CECR1	51816	cat eye syndrome chromosome region, candidate 1	ADGF|IDGFL	22	Cytokines

CER1	9350	cerberus 1, cysteine knot superfamily, homolog (Xenopus laevis)	DAND4|MGC119894|MGC119895|MGC96951	9	Cytokines
CGA	1081	glycoprotein hormones, alpha polypeptide	CG-ALPHA|FSHA|GPHA1|GPHa|HCG|LHA|TSHA	6	Cytokines
CGB	1082	chorionic gonadotropin, beta polypeptide	CGB3|hCGB	19	Cytokines
CGB1	114335	chorionic gonadotropin, beta polypeptide 1	-	19	Cytokines
CGB2	114336	chorionic gonadotropin, beta polypeptide 2	-	19	Cytokines
CGB5	93659	chorionic gonadotropin, beta polypeptide 5	HCG|MGC119822	19	Cytokines
CGB7	94027	chorionic gonadotropin, beta polypeptide 7	CG-beta-a|FLJ35403|FLJ43118	19	Cytokines
CGB8	94115	chorionic gonadotropin, beta polypeptide 8	-	19	Cytokines
CHGA	1113	chromogranin A (parathyroid secretory protein 1)	CGA	14	Cytokines
CHGB	1114	chromogranin B (secretogranin 1)	SCG1	20	Cytokines
CKLF	51192	chemokine-like factor	C32|CKLF1|CKLF2|CKLF3|CKLF4|HSPC224|UCK-1	16	Cytokines
CLCF1	23529	cardiotrophin-like cytokine factor 1	BSF3|CISS2|CLC|NNT1|NR6	11	Cytokines

CLEC11A	6320	C-type lectin domain family 11, member A	CLECSF3|LSLCL|P47|SCGF	19	Cytokines
CMA1	1215	chymase 1, mast cell	CYH|MCT1|MGC119890|MGC119891|chymase	14	Cytokines
CMTM1	113540	CKLF-like MARVEL transmembrane domain containing 1	CKLFH|CKLFH1|CKLFSF1|MGC71870	16	Cytokines
CMTM2	146225	CKLF-like MARVEL transmembrane domain containing 2	CKLFSF2|MGC39436	16	Cytokines
CMTM3	123920	CKLF-like MARVEL transmembrane domain containing 3	BNAS2|CKLFSF3|FLJ31762|MGC51956	16	Cytokines
CMTM4	146223	CKLF-like MARVEL transmembrane domain containing 4	CKLFSF4	16	Cytokines
CMTM5	116173	CKLF-like MARVEL transmembrane domain containing 5	CKLFSF5|FLJ37521	14	Cytokines
CMTM6	54918	CKLF-like MARVEL transmembrane domain containing 6	CKLFSF6|FLJ20396|PRO2219	3	Cytokines
CMTM7	112616	CKLF-like MARVEL transmembrane domain containing 7	CKLFSF7|FLJ30992	3	Cytokines

CMTM8	152189	CKLF-like MARVEL transmembrane domain containing 8	CKLFSF8|CKLFSF8-V2	3	Cytokines
CNTF	1270	ciliary neurotrophic factor	HCNTF	11	Cytokines
CORT	1325	cortistatin	CST-14|CST-17|CST-29	1	Cytokines
CRH	1392	corticotropin releasing hormone	CRF	8	Cytokines
CSF1	1435	colony stimulating factor 1 (macrophage)	MCSF|MGC31930	1	Cytokines
CSF2	1437	colony stimulating factor 2 (granulocyte-macrophage)	GMCSF|MGC131935|MGC138897	5	Cytokines
CSF3	1440	colony stimulating factor 3 (granulocyte)	G-CSF|GCSF|MGC45931	17	Cytokines
CSH1	1442	chorionic somatomammotropin hormone 1 (placental lactogen)	CSA|CSMT|FLJ75407|PL	17	Cytokines
CSH2	1443	chorionic somatomammotropin hormone 2	CS-2|CSB|hCS-B	17	Cytokines
CSHL1	1444	chorionic somatomammotropin hormone-like 1	CS-5|CSHP1|CSL|MGC149868|hCS-L	17	Cytokines
CSPG5	10675	chondroitin sulfate proteoglycan 5 (neuroglycan C)	MGC44034|NGC	3	Cytokines
CTF1	1489	cardiotrophin 1	CT-1|CT1	16	Cytokines
CTGF	1490	connective tissue growth factor	CCN2|HCS24|IGFBP8|MGC102839|NOV2	6	Cytokines
CTSG	1511	cathepsin G	CG|MGC23078	14	Cytokines
CX3CL1	6376	chemokine (C-X3-C motif) ligand 1	ABCD-3|C3Xkine|CXC3|CXC3C|NTN|NTT|SCYD1|fractalkine|neurotactin	16	Cytokines

CXCL1	2919	chemokine (C-X-C motif) ligand 1 (melanoma growth stimulating activity, alpha)	FSP|GRO1|GROa|MGSA|MGSA-a|NAP-3|SCYB1	4	Cytokines
CXCL10	3627	chemokine (C-X-C motif) ligand 10	C7|IFI10|INP10|IP-10|SCYB10|crg-2|gIP-10|mob-1	4	Cytokines
CXCL11	6373	chemokine (C-X-C motif) ligand 11	H174|I-TAC|IP-9|IP9|MGC102770|SCYB11|SCYB9B|b-R1	4	Cytokines
CXCL12	6387	chemokine (C-X-C motif) ligand 12 (stromal cell-derived factor 1)	PBSF|SCYB12|SDF-1a|SDF-1b|SDF1|SDF1A|SDF1B|TLSF-a|TLSF-b|TPAR1	10	Cytokines
CXCL13	10563	chemokine (C-X-C motif) ligand 13	ANGIE|ANGIE2|BCA-1|BCA1|BLC|BLR1L|SCYB13	4	Cytokines
CXCL14	9547	chemokine (C-X-C motif) ligand 14	BMAC|BRAK|KS1|Kec|MGC10687|MIP-2g|NJAC|SCYB14|bolekine	5	Cytokines
CXCL16	58191	chemokine (C-X-C motif) ligand 16	CXCLG16|SR-PSOX|SRPSOX	17	Cytokines
CXCL17	284340	chemokine (C-X-C motif) ligand 17	DMC|Dcip1|MGC138300|UNQ473|VCC-1|VCC1	19	Cytokines
CXCL2	2920	chemokine (C-X-C motif) ligand 2	CINC-2a|GRO2|GROb|MGSA-b|MIP-2a|MIP2|MIP2A|SCYB2	4	Cytokines
CXCL3	2921	chemokine (C-X-C motif) ligand 3	CINC-2b|GRO3|GROg|MIP-2b|MIP2B|SCYB3	4	Cytokines
CXCL5	6374	chemokine (C-X-C motif) ligand 5	ENA-78|SCYB5	4	Cytokines
CXCL6	6372	chemokine (C-X-C motif) ligand 6 (granulocyte chemotactic protein 2)	CKA-3|GCP-2|GCP2|SCYB6	4	Cytokines

CXCL9	4283	chemokine (C-X-C motif) ligand 9	CMK|Humig|MIG|SCYB9|crg-10	4	Cytokines
CYR61	3491	cysteine-rich, angiogenic inducer, 61	CCN1|GIG1|IGFBP10	1	Cytokines
DEFA1	1667	defensin, alpha 1	DEF1|DEFA2|HNP-1|HP-1|MGC138393|MRS	8	Cytokines
DEFA3	1668	defensin, alpha 3, neutrophil-specific	DEF3|HNP-3|HNP3|HP-3	8	Cytokines
DEFA5	1670	defensin, alpha 5, Paneth cell-specific	DEF5|HD-5|MGC129728	8	Cytokines
DEFB1	1672	defensin, beta 1	BD1|DEFB-1|DEFB101|HBD1|MGC51822	8	Cytokines
DEFB103A	55894	defensin, beta 103A	DEFB103|DEFB3|HBD-3|HBD3|HBP-3|HBP3	8	Cytokines
DEFB104A	140596	defensin, beta 104A	BD-4|DEFB-4|DEFB104|DEFB4|MGC118942|MGC118944|MGC118945|hBD-4	8	Cytokines
DEFB4	1673	defensin, beta 4	DEFB-2|DEFB102|DEFB2|HBD-2|SAP1	8	Cytokines
DKK1	22943	dickkopf homolog 1 (Xenopus laevis)	DKK-1|SK	10	Cytokines
EBI3	10148	Epstein-Barr virus induced 3	IL27B	19	Cytokines
EDN1	1906	endothelin 1	ET1|HDLCQ7	6	Cytokines
EDN2	1907	endothelin 2	ET2|PPET2	1	Cytokines
EDN3	1908	endothelin 3	ET3|MGC15067|MGC61498	20	Cytokines
EGF	1950	epidermal growth factor (beta-urogastrone)	HOMG4|URG	4	Cytokines
EPGN	255324	epithelial mitogen homolog (mouse)	ALGV3072|EPG|FLJ75542|PRO9904|epigen	4	Cytokines
EPO	2056	erythropoietin	EP|MGC138142|MVCD2	7	Cytokines

EREG	2069	epiregulin	ER	4	Cytokines
ESM1	11082	endothelial cell-specific molecule 1	endocan	5	Cytokines
FAM3B	54097	family with sequence similarity 3, member B	2-21|C21orf11|C21orf76|ORF9|PANDER|PRED44	21	Cytokines
FAM3C	10447	family with sequence similarity 3, member C	GS3786|ILEI	7	Cytokines
FAM3D	131177	family with sequence similarity 3, member D	EF7|OIT1	3	Cytokines
FASLG	356	Fas ligand (TNF superfamily, member 6)	APT1LG1|CD178|CD95L|FASL|TNFSF6	1	Cytokines
FGF1	2246	fibroblast growth factor 1 (acidic)	AFGF|ECGF|ECGF-beta|ECGFA|ECGFB|FGF-alpha|FGFA|GLIO703|HBGF1	5	Cytokines
FGF10	2255	fibroblast growth factor 10	-	5	Cytokines
FGF11	2256	fibroblast growth factor 11	FHF3|FLJ16061|MGC102953|MGC45269	17	Cytokines
FGF12	2257	fibroblast growth factor 12	FGF12B|FHF1	3	Cytokines
FGF13	2258	fibroblast growth factor 13	FGF2|FHF-2|FHF2	X	Cytokines
FGF14	2259	fibroblast growth factor 14	FHF4|MGC119129|SCA27	13	Cytokines
FGF16	8823	fibroblast growth factor 16	-	X	Cytokines
FGF17	8822	fibroblast growth factor 17	FGF-13	8	Cytokines
FGF18	8817	fibroblast growth factor 18	FGF-18|ZFGF5	5	Cytokines
FGF19	9965	fibroblast growth factor 19	-	11	Cytokines
FGF2	2247	fibroblast growth factor 2 (basic)	BFGF|FGFB|HBGF-2	4	Cytokines
FGF20	26281	fibroblast growth factor 20	-	8	Cytokines
FGF21	26291	fibroblast growth factor 21	-	19	Cytokines
FGF22	27006	fibroblast growth factor 22	-	19	Cytokines

FGF23	8074	fibroblast growth factor 23	ADHR|HPDR2|HYPF|PHPTC	12	Cytokines
FGF3	2248	fibroblast growth factor 3 (murine mammary tumor virus integration site (v-int-2) oncogene homolog)	HBGF-3|INT2	11	Cytokines
FGF4	2249	fibroblast growth factor 4	HBGF-4|HST|HST-1|HSTF1|K-FGF|KFGF	11	Cytokines
FGF5	2250	fibroblast growth factor 5	HBGF-5|Smag-82	4	Cytokines
FGF6	2251	fibroblast growth factor 6	HBGF-6|HST2	12	Cytokines
FGF7	2252	fibroblast growth factor 7 (keratinocyte growth factor)	HBGF-7|KGF	15	Cytokines
FGF8	2253	fibroblast growth factor 8 (androgen-induced)	AIGF|HBGF-8|KAL6|MGC149376	10	Cytokines
FGF9	2254	fibroblast growth factor 9 (glia-activating factor)	GAF|HBFG-9|MGC119914|MGC119915	13	Cytokines
FIGF	2277	c-fos induced growth factor (vascular endothelial growth factor D)	VEGF-D|VEGFD	X	Cytokines
FIGNL2	401720	fidgetin-like 2	-	12	Cytokines
FLT3LG	2323	fms-related tyrosine kinase 3 ligand	FL	19	Cytokines
FSHB	2488	follicle stimulating hormone, beta polypeptide	-	11	Cytokines
GAL	51083	galanin prepropeptide	GALN|GLNN|GMAP|MGC40167	11	Cytokines
GALP	85569	galanin-like peptide	-	19	Cytokines
GAST	2520	gastrin	GAS	17	Cytokines
GCG	2641	glucagon	GLP1|GLP2|GRPP	2	Cytokines

GDF1	2657	growth differentiation factor 1	-	19	Cytokines
GDF10	2662	growth differentiation factor 10	BMP-3b|BMP3B	10	Cytokines
GDF11	10220	growth differentiation factor 11	BMP-11|BMP11	12	Cytokines
GDF15	9518	growth differentiation factor 15	GDF-15|MIC-1|MIC1|NAG-1|PDF|PLAB|PTGFB	19	Cytokines
GDF2	2658	growth differentiation factor 2	BMP-9|BMP9	10	Cytokines
GDF3	9573	growth differentiation factor 3	-	12	Cytokines
GDF5	8200	growth differentiation factor 5	BMP14|CDMP1|LAP4|OS5|SYNS2	20	Cytokines
GDF6	392255	growth differentiation factor 6	BMP13|CDMP2|KFS|KFSL|MGC158100|MGC158101|SGM1	8	Cytokines
GDF7	151449	growth differentiation factor 7	BMP12	2	Cytokines
GDF9	2661	growth differentiation factor 9	-	5	Cytokines
GDNF	2668	glial cell derived neurotrophic factor	ATF1|ATF2|HFB1-GDNF	5	Cytokines
GH1	2688	growth hormone 1	GH|GH-N|GHN|hGH-N	17	Cytokines
GH2	2689	growth hormone 2	GH-V|GHL|GHV|hGH-V	17	Cytokines
GHRH	2691	growth hormone releasing hormone	GHRF|GRF|MGC119781	20	Cytokines
GHRL	51738	ghrelin/obestatin prepropeptide	MTLRP|obestatin	3	Cytokines
GIP	2695	gastric inhibitory polypeptide	-	17	Cytokines
GKN1	56287	gastrokine 1	AMP18|BRICD1|CA11|FOV|MGC70354|foveolin	2	Cytokines
GMFB	2764	glia maturation factor, beta	GMF	14	Cytokines
GMFG	9535	glia maturation factor, gamma	GMF-GAMMA|MGC126867	19	Cytokines
GNRH1	2796	gonadotropin-releasing hormone 1 (luteinizing-releasing hormone)	GNRH|GRH|LHRH|LNRH	8	Cytokines

GNRH2	2797	gonadotropin-releasing hormone 2	GnRH-II|LH-RHII	20	Cytokines
GPHA2	170589	glycoprotein hormone alpha 2	A2|GPA2|MGC126572|ZSIG51	11	Cytokines
GPHB5	122876	glycoprotein hormone beta 5	B5|GPB5|ZLUT1	14	Cytokines
GPI	2821	glucose phosphate isomerase	AMF|GNPI|NLK|PGI|PHI|SA-36	19	Cytokines
GREM1	26585	gremlin 1, cysteine knot superfamily, homolog (Xenopus laevis)	CKTSF1B1|DAND2|DRM|GREMLIN|IHG-2|MGC126660|PIG2	15	Cytokines
GREM2	64388	gremlin 2, cysteine knot superfamily, homolog (Xenopus laevis)	CKTSF1B2|DAND3|PRDC	1	Cytokines
GRN	2896	granulin	GEP|GP88|PCDGF|PEPI|PGRN	17	Cytokines
GRP	2922	gastrin-releasing peptide	BN|GRP-10|preproGRP|proGRP	18	Cytokines
GUCA2A	2980	guanylate cyclase activator 2A (guanylin)	GUANYLIN|GUCA2|STARA	1	Cytokines
HAMP	57817	hepcidin antimicrobial peptide	HEPC|HEPCIDIN|HFE2B|LEAP-1|LEAP1|PLTR	19	Cytokines
HBEGF	1839	heparin-binding EGF-like growth factor	DTR|DTS|DTSF|HEGFL	5	Cytokines
HDGF	3068	hepatoma-derived growth factor (high-mobility group protein 1-like)	DKFZp686J1764|FLJ96580|HMG1L2	1	Cytokines
HDGFRP3	50810	hepatoma-derived growth factor, related protein 3	CGI-142|HDGF2	15	Cytokines
HGF	3082	hepatocyte growth factor (hepapoietin A; scatter factor)	F-TCF|HGFB|HPTA|SF	7	Cytokines
HTN3	3347	histatin 3	HIS2|HTN2|HTN5	4	Cytokines

IAPP	3375	islet amyloid polypeptide	AMYLIN|DAP|IAP	12	Cytokines
IFNA1	3439	interferon, alpha 1	IFL|IFN|IFN-ALPHA|IFNA13|IFNA@|MGC138207|MGC138505|MGC138507	9	Cytokines
IFNA10	3446	interferon, alpha 10	MGC119878|MGC119879	9	Cytokines
IFNA13	3447	interferon, alpha 13	-	9	Cytokines
IFNA14	3448	interferon, alpha 14	LEIF2H|MGC125756|MGC125757	9	Cytokines
IFNA16	3449	interferon, alpha 16	-	9	Cytokines
IFNA17	3451	interferon, alpha 17	IFNA|INFA|LEIF2C1	9	Cytokines
IFNA2	3440	interferon, alpha 2	IFNA|INFA2|MGC125764|MGC125765	9	Cytokines
IFNA21	3452	interferon, alpha 21	MGC126687|MGC126689	9	Cytokines
IFNA4	3441	interferon, alpha 4	INFA4|MGC142200	9	Cytokines
IFNA5	3442	interferon, alpha 5	INFA5	9	Cytokines
IFNA6	3443	interferon, alpha 6	-	9	Cytokines
IFNA7	3444	interferon, alpha 7	IFNA-J	9	Cytokines
IFNA8	3445	interferon, alpha 8	-	9	Cytokines
IFNB1	3456	interferon, beta 1, fibroblast	IFB|IFF|IFNB|MGC96956	9	Cytokines
IFNE	338376	interferon, epsilon	IFN-E|IFNE1|IFNT1|MGC119018|MGC119020|PRO655	9	Cytokines
IFNG	3458	interferon, gamma	IFG|IFI	12	Cytokines
IFNK	56832	interferon, kappa	RP11-27J8.1	9	Cytokines
IFNW1	3467	interferon, omega 1	-	9	Cytokines
IGF1	3479	insulin-like growth factor 1 (somatomedin C)	IGF1A|IGFI	12	Cytokines

IGF2	3481	insulin-like growth factor 2 (somatomedin A)	C11orf43|FLJ22066|FLJ44734|INSIGF|pp9974	11	Cytokines
IL10	3586	interleukin 10	CSIF|IL-10|IL10A|MGC126450|MGC126451|TGIF	1	Cytokines
IL11	3589	interleukin 11	AGIF|IL-11	19	Cytokines
IL12A	3592	interleukin 12A (natural killer cell stimulatory factor 1, cytotoxic lymphocyte maturation factor 1, p35)	CLMF|IL-12A|NFSK|NKSF1|P35	3	Cytokines
IL12B	3593	interleukin 12B (natural killer cell stimulatory factor 2, cytotoxic lymphocyte maturation factor 2, p40)	CLMF|CLMF2|IL-12B|NKSF|NKSF2	5	Cytokines
IL13	3596	interleukin 13	ALRH|BHR1|IL-13|MGC116786|MGC116788|MGC116789|P600	5	Cytokines
IL15	3600	interleukin 15	IL-15|MGC9721	4	Cytokines
IL16	3603	interleukin 16 (lymphocyte chemoattractant factor)	FLJ16806|FLJ42735|FLJ44234|HsT19289|IL-16|LCF|prIL-16	15	Cytokines
IL17A	3605	interleukin 17A	CTLA8|IL-17|IL-17A|IL17	6	Cytokines
IL17B	27190	interleukin 17B	IL-17B|IL-20|MGC138900|MGC138901|ZCYTO7	5	Cytokines
IL17C	27189	interleukin 17C	CX2|IL-17C|IL-21|MGC126884|MGC138401	16	Cytokines
IL17D	53342	interleukin 17D	FLJ30846|IL-17D|IL-22|IL-27|IL27	13	Cytokines
IL17F	112744	interleukin 17F	IL-17F|ML-1|ML1	6	Cytokines
IL18	3606	interleukin 18 (interferon-gamma-inducing factor)	IGIF|IL-18|IL-1g|IL1F4|MGC12320	11	Cytokines
IL19	29949	interleukin 19	IL-10C|MDA1|NG.1|ZMDA1	1	Cytokines

IL1A	3552	interleukin 1, alpha	IL-1A|IL1|IL1-ALPHA|IL1F1	2	Cytokines
IL1B	3553	interleukin 1, beta	IL-1|IL1-BETA|IL1F2	2	Cytokines
IL1F10	84639	interleukin 1 family, member 10 (theta)	FIL1-theta|FKSG75|IL-1HY2|IL1-theta|MGC119831|MGC119832|MGC119833	2	Cytokines
IL1F5	26525	interleukin 1 family, member 5 (delta)	FIL1|FIL1(DELTA)|FIL1D|IL1HY1|IL1L1|IL1RP3|MGC29840	2	Cytokines
IL1F6	27179	interleukin 1 family, member 6 (epsilon)	FIL1|FIL1(EPSILON)|FIL1E|IL-1F6|IL1(EPSILON)|MGC129552|MGC129553	2	Cytokines
IL1F7	27178	interleukin 1 family, member 7 (zeta)	FIL1|FIL1(ZETA)|FIL1Z|IL-1F7|IL-1H4|IL-1RP1|IL1H4|IL1RP1	2	Cytokines
IL1F8	27177	interleukin 1 family, member 8 (eta)	FIL1|FIL1-(ETA)|FIL1H|IL-1F8|IL-1H2|IL1-ETA|IL1H2|MGC126880|MGC126882	2	Cytokines
IL1F9	56300	interleukin 1 family, member 9	IL-1F9|IL-1H1|IL-1RP2|IL1E|IL1H1|IL1RP2	2	Cytokines
IL1RN	3557	interleukin 1 receptor antagonist	ICIL-1RA|IL-1ra3|IL1F3|IL1RA|IRAP|MGC10430	2	Cytokines
IL2	3558	interleukin 2	IL-2|TCGF|lymphokine	4	Cytokines
IL20	50604	interleukin 20	IL-20|IL10D|MGC96907|ZCYTO10	1	Cytokines
IL21	59067	interleukin 21	IL-21|Za11	4	Cytokines
IL22	50616	interleukin 22	IL-21|IL-22|IL-D110|IL-TIF|IL21|ILTIF|MGC79382|MGC79384|TIFIL-23|TIFa|zcyto18	12	Cytokines
IL23A	51561	interleukin 23, alpha subunit p19	IL-23|IL-23A|IL23P19|MGC79388|P19|SGRF	12	Cytokines
IL24	11009	interleukin 24	C49A|FISP|IL-24|IL10B|MDA7|Mob-5|ST16|mda-7	1	Cytokines
IL25	64806	interleukin 25	IL-17E|IL-25|IL17E	14	Cytokines
IL26	55801	interleukin 26	AK155|IL-26	12	Cytokines
IL27	246778	interleukin 27	IL-27|IL-27A|IL27p28|IL30|MGC71873|p28	16	Cytokines

IL28A	282616	interleukin 28A (interferon, lambda 2)	IFNL2|IL-28A	19	Cytokines
IL28B	282617	interleukin 28B (interferon, lambda 3)	IFNL3|IL-28B|IL28C	19	Cytokines
IL29	282618	interleukin 29 (interferon, lambda 1)	IFNL1|IL-29	19	Cytokines
IL3	3562	interleukin 3 (colony-stimulating factor, multiple)	IL-3|MCGF|MGC79398|MGC79399|MULTI-CSF	5	Cytokines
IL31	386653	interleukin 31	IL-31	12	Cytokines
IL32	9235	interleukin 32	IL-32alpha|IL-32beta|IL-32delta|IL-32gamma|NK4|TAIF|TAIFa|TAIFb|TAIFc|TAIFd	16	Cytokines
IL33	90865	interleukin 33	C9orf26|DKFZp586H0523|DVS27|NF-HEV|NFEHEV|RP11-575C20.2	9	Cytokines
IL34	146433	interleukin 34	C16orf77|IL-34|MGC34647	16	Cytokines
IL4	3565	interleukin 4	BCGF-1|BCGF1|BSF1|IL-4|MGC79402	5	Cytokines
IL5	3567	interleukin 5 (colony-stimulating factor, eosinophil)	EDF|IL-5|TRF	5	Cytokines
IL6	3569	interleukin 6 (interferon, beta 2)	BSF2|HGF|HSF|IFNB2|IL-6	7	Cytokines
IL6ST	3572	interleukin 6 signal transducer (gp130, oncostatin M receptor)	CD130|CDw130|GP130|GP130-RAPS|IL6R-beta	5	Cytokines
IL7	3574	interleukin 7	IL-7	8	Cytokines
IL8	3576	interleukin 8	CXCL8|GCP-1|GCP1|LECT|LUCT|LYNAP|MDNCF|MONAP|NAF|NAP-1|NAP1	4	Cytokines
IL9	3578	interleukin 9	HP40|IL-9|P40	5	Cytokines
INHA	3623	inhibin, alpha	-	2	Cytokines

INHBA	3624	inhibin, beta A	EDF|FRP	7	Cytokines
INHBB	3625	inhibin, beta B	MGC157939	2	Cytokines
INHBC	3626	inhibin, beta C	IHBC	12	Cytokines
INHBE	83729	inhibin, beta E	MGC4638	12	Cytokines
INS	3630	insulin	ILPR|IRDN	11	Cytokines
INS-IGF2	723961	INS-IGF2 readthrough transcript	-	11	Cytokines
INSL3	3640	insulin-like 3 (Leydig cell)	MGC119818|MGC119819|RLF|RLNL	19	Cytokines
INSL4	3641	insulin-like 4 (placenta)	EPIL|PLACENTIN	9	Cytokines
INSL5	10022	insulin-like 5	MGC126695|MGC126697|PRO182|UNQ156	1	Cytokines
INSL6	11172	insulin-like 6	RIF1	9	Cytokines
JAG1	182	jagged 1 (Alagille syndrome)	AGS|AHD|AWS|CD339|HJ1|JAGL1|MGC104644	20	Cytokines
JAG2	3714	jagged 2	HJ2|SER2	14	Cytokines
KGFLP1	387628	keratinocyte growth factor-like protein 1	MGC125746|MGC125747|MGC126891	9	Cytokines
KGFLP2	654466	keratinocyte growth factor-like protein 2	-	9	Cytokines
KITLG	4254	KIT ligand	DKFZp686F2250|KL-1|Kitl|MGF|SCF|SF|SHEP7	12	Cytokines
KL	9365	klotho	-	13	Cytokines
LACRT	90070	lacritin	MGC71934	12	Cytokines
LECT2	3950	leukocyte cell-derived chemotaxin 2	MGC126628|chm-II|chm2	5	Cytokines
LEFTY1	10637	left-right determination factor 1	LEFTB|LEFTYB	1	Cytokines
LEFTY2	7044	left-right determination factor 2	EBAF|LEFTA|LEFTYA|MGC46222|TGFB4	1	Cytokines
LEP	3952	leptin	FLJ94114|OB|OBS	7	Cytokines

LHB	3972	luteinizing hormone beta polypeptide	CGB4|LSH-B|hLHB	19	Cytokines
LIF	3976	leukemia inhibitory factor (cholinergic differentiation factor)	CDF|DIA|HILDA	22	Cytokines
LRSAM1	90678	leucine rich repeat and sterile alpha motif containing 1	FLJ31641|RIFLE|TAL	9	Cytokines
LTA	4049	lymphotoxin alpha (TNF superfamily, member 1)	LT|TNFB|TNFSF1	6	Cytokines
LTB	4050	lymphotoxin beta (TNF superfamily, member 3)	TNFC|TNFSF3|p33	6	Cytokines
LTBP1	4052	latent transforming growth factor beta binding protein 1	MGC163161	2	Cytokines
LTBP2	4053	latent transforming growth factor beta binding protein 2	C14orf141|LTBP3|MSTP031	14	Cytokines
LTBP3	4054	latent transforming growth factor beta binding protein 3	DKFZp586M2123|FLJ33431|FLJ39893|FLJ42533|FLJ44138|FLJ45576|LTBP-3|LTBP2|pp6425	11	Cytokines
LTBP4	8425	latent transforming growth factor beta binding protein 4	FLJ46318|FLJ90018|LTBP-4|LTBP-4L	19	Cytokines
MDK	4192	midkine (neurite growth-promoting factor 2)	FLJ27379|MK|NEGF2	11	Cytokines
MIA	8190	melanoma inhibitory activity	CD-RAP	19	Cytokines
MIF	4282	macrophage migration inhibitory factor (glycosylation-inhibiting factor)	GIF|GLIF|MMIF	22	Cytokines
MLN	4295	motilin	MGC138519	6	Cytokines
MSTN	2660	myostatin	GDF8	2	Cytokines

NAMPT	10135	nicotinamide phosphoribosyltransferase	1110035O14Rik|DKFZp666B131|MGC117256|PBEF|PBEF1|VF|VISFATIN	7	Cytokines
NDP	4693	Norrie disease (pseudoglioma)	EVR2|FEVR|ND	X	Cytokines
NENF	29937	neuron derived neurotrophic factor	CIR2|NEUDESIN|SCIRP10|SPUF	1	Cytokines
NGF	4803	nerve growth factor (beta polypeptide)	Beta-NGF|HSAN5|MGC161426|MGC161428|NGFB	1	Cytokines
NMB	4828	neuromedin B	MGC17211|MGC2277|MGC3936	15	Cytokines
NODAL	4838	nodal homolog (mouse)	MGC138230	10	Cytokines
NOV	4856	nephroblastoma overexpressed gene	CCN3|IGFBP9	8	Cytokines
NPFF	8620	neuropeptide FF-amide peptide precursor	FMRFAL	12	Cytokines
NPPA	4878	natriuretic peptide precursor A	ANF|ANP|ATFB6|CDD-ANF|PND	1	Cytokines
NPPB	4879	natriuretic peptide precursor B	BNP	1	Cytokines
NPPC	4880	natriuretic peptide precursor C	CNP	2	Cytokines
NPY	4852	neuropeptide Y	PYY4	7	Cytokines
NRG1	3084	neuregulin 1	ARIA|GGF|GGF2|HGL|HRG|HRG1|HRGA|NDF|SMDF	8	Cytokines
NRG2	9542	neuregulin 2	Don-1|HRG2|NTAK	5	Cytokines
NRG3	10718	neuregulin 3	HRG3|pro-NRG3	10	Cytokines
NRG4	145957	neuregulin 4	DKFZp779N0541|DKFZp779N1944|HRG4	15	Cytokines
NRTN	4902	neurturin	NTN	19	Cytokines
NTF3	4908	neurotrophin 3	HDNF|MGC129711|NGF-2|NGF2|NT3	12	Cytokines
NTF4	4909	neurotrophin 4	NT-4/5|NT4|NT5|NTF5	19	Cytokines
NTS	4922	neurotensin	NMN-125|NN|NT|NT/N|NTS1	12	Cytokines

NUDT6	11162	nudix (nucleoside diphosphate linked moiety X)-type motif 6	ASFGF2|FGF-2|FGF-AS|FGF2AS|bFGF|gfg|gfg-1	4	Cytokines
OGN	4969	osteoglycin	DKFZp586P2421|OG|OIF|SLRR3A	9	Cytokines
OSGIN1	29948	oxidative stress induced growth inhibitor 1	BDGI|OKL38	16	Cytokines
OSM	5008	oncostatin M	MGC20461	22	Cytokines
OSTN	344901	osteocrin	MUSCLIN	3	Cytokines
OXT	5020	oxytocin, prepropeptide	MGC126890|MGC126892|OT|OT-NPI	20	Cytokines
P11	8909	26 serine protease	MGC133268|PP11|PRSS26	12	Cytokines
PDGFA	5154	platelet-derived growth factor alpha polypeptide	PDGF-A|PDGF1	7	Cytokines
PDGFB	5155	platelet-derived growth factor beta polypeptide (simian sarcoma viral (v-sis) oncogene homolog)	FLJ12858|PDGF2|SIS|SSV|c-sis	22	Cytokines
PDGFC	56034	platelet derived growth factor C	FALLOTEIN|SCDGF	4	Cytokines
PDGFD	80310	platelet derived growth factor D	IEGF|MGC26867|MSTP036|SCDGF-B|SCDGFB	11	Cytokines
PDGFRA	5156	platelet-derived growth factor receptor, alpha polypeptide	CD140A|MGC74795|PDGFR2|Rhe-PDGFRA	4	Cytokines
PDGFRB	5159	platelet-derived growth factor receptor, beta polypeptide	CD140B|JTK12|PDGF-R-beta|PDGFR|PDGFR1	5	Cytokines
PDGFRL	5157	platelet-derived growth factor receptor-like	PDGRL|PRLTS	8	Cytokines
PDYN	5173	prodynorphin	MGC26418|PENKB	20	Cytokines
PENK	5179	proenkephalin	-	8	Cytokines
PF4	5196	platelet factor 4	CXCL4|MGC138298|SCYB4	4	Cytokines

PF4V1	5197	platelet factor 4 variant 1	CXCL4L1|CXCL4V1|PF4-ALT|PF4A|SCYB4V1	4	Cytokines
PGF	5228	placental growth factor	D12S1900|PGFL|PLGF|PlGF-2|SHGC-10760	14	Cytokines
PLAU	5328	plasminogen activator, urokinase	ATF|UPA|URK|u-PA	10	Cytokines
PMCH	5367	pro-melanin-concentrating hormone	MCH	12	Cytokines
PNOC	5368	prepronociceptin	PPNOC	8	Cytokines
POMC	5443	proopiomelanocortin	ACTH|CLIP|LPH|MSH|NPP|POC	2	Cytokines
PPBP	5473	pro-platelet basic protein (chemokine (C-X-C motif) ligand 7)	B-TG1|Beta-TG|CTAP-III|CTAP3|CTAPIII|CXCL7|LA-PF4|LDGF|MDGF|NAP-2|PBP|SCYB7|TC1|TC2|TGB|TGB1|THBGB|THBGB1	4	Cytokines
PPBPL1	728045	pro-platelet basic protein-like 1	TGB2	4	Cytokines
PPBPL2	10895	pro-platelet basic protein-like 2	SPBPBP	4	Cytokines
PPY	5539	pancreatic polypeptide	PNP	17	Cytokines
PRL	5617	prolactin	-	6	Cytokines
PRLH	51052	prolactin releasing hormone	PRH|PRRP	2	Cytokines
PROK1	84432	prokineticin 1	EGVEGF|PK1|PRK1	1	Cytokines
PROK2	60675	prokineticin 2	BV8|KAL4|MIT1|PK2	3	Cytokines
PSPN	5623	persephin	PSP	19	Cytokines
PTH	5741	parathyroid hormone	PTH1	11	Cytokines
PTH2	113091	parathyroid hormone 2	TIP39	19	Cytokines
PTHLH	5744	parathyroid hormone-like hormone	HHM|MGC14611|PLP|PTHR|PTHRP	12	Cytokines
PTN	5764	pleiotrophin	HARP|HBGF8|HBNF|NEGF1	7	Cytokines

PYY	5697	peptide YY	PYY1	17	Cytokines
QRFP	347148	pyroglutamylated RFamide peptide	26RFa|MGC119794|P518	9	Cytokines
RABEP1	9135	rabaptin, RAB GTPase binding effector protein 1	RAB5EP|RABPT5	17	Cytokines
RABEP2	79874	rabaptin, RAB GTPase binding effector protein 2	FLJ23282|FRA	16	Cytokines
REG1A	5967	regenerating islet-derived 1 alpha	ICRF|MGC12447|P19|PSP|PSPS|PSPS1|PTP|REG	2	Cytokines
RETN	56729	resistin	ADSF|FIZZ3|MGC126603|MGC126609|RETN1|RSTN|XCP1	19	Cytokines
RETNLB	84666	resistin like beta	FIZZ1|FIZZ2|HXCP2|RELM-beta|RELMb|RELMbeta|XCP2	3	Cytokines
RLN1	6013	relaxin 1	H1|RLXH1|bA12D24.3.1|bA12D24.3.2	9	Cytokines
RLN2	6019	relaxin 2	H2|RLXH2|bA12D24.1.1|bA12D24.1.2	9	Cytokines
RLN3	117579	relaxin 3	H3|RXN3|ZINS4|insl7	19	Cytokines
RNASE2	6036	ribonuclease, RNase A family, 2 (liver, eosinophil-derived neurotoxin)	EDN|RNS2	14	Cytokines
S100A6	6277	S100 calcium binding protein A6	2A9|5B10|CABP|CACY|PRA	1	Cytokines
SAA1	6288	serum amyloid A1	MGC111216|PIG4|SAA|TP53I4	11	Cytokines
SAA2	6289	serum amyloid A2	-	11	Cytokines
SBDS	51119	Shwachman-Bodian-Diamond syndrome	CGI-97|FLJ10917|SDS|SWDS	7	Cytokines
SCG2	7857	secretogranin II (chromogranin C)	CHGC|SN|SgII	2	Cytokines

SCGB3A1	92304	secretoglobin, family 3A, member 1	HIN-1|HIN1|LU105|MGC87867|PnSP-2|UGRP2	5	Cytokines
SCT	6343	secretin	-	11	Cytokines
SCYE1	9255	small inducible cytokine subfamily E, member 1 (endothelial monocyte-activating)	AIMP1|EMAP2|EMAPII|p43	4	Cytokines
SECTM1	6398	secreted and transmembrane 1	K12	17	Cytokines
SEMA3A	10371	sema domain, immunoglobulin domain (Ig), short basic domain, secreted, (semaphorin) 3A	Hsema-I|Hsema-III|MGC133243|SEMA1|SEMAD|SEMAIII|SEMAL|SemD|coll-1	7	Cytokines
SEMA3B	7869	sema domain, immunoglobulin domain (Ig), short basic domain, secreted, (semaphorin) 3B	FLJ34863|LUCA-1|SEMA5|SEMAA|SemA|semaV	3	Cytokines
SEMA3C	10512	sema domain, immunoglobulin domain (Ig), short basic domain, secreted, (semaphorin) 3C	SEMAE|SemE	7	Cytokines
SEMA3D	223117	sema domain, immunoglobulin domain (Ig), short basic domain, secreted, (semaphorin) 3D	MGC39708|Sema-Z2|coll-2	7	Cytokines
SEMA3E	9723	sema domain, immunoglobulin domain (Ig), short basic domain, secreted, (semaphorin) 3E	KIAA0331|M-SEMAH|M-SemaK|SEMAH|coll-5	7	Cytokines
SEMA3F	6405	sema domain, immunoglobulin domain (Ig), short basic domain, secreted, (semaphorin) 3F	SEMA-IV|SEMA4|SEMAK	3	Cytokines

SEMA3G	56920	sema domain, immunoglobulin domain (Ig), short basic domain, secreted, (semaphorin) 3G	FLJ00014|MGC119473|sem2	3	Cytokines
SEMA4A	64218	sema domain, immunoglobulin domain (Ig), transmembrane domain (TM) and short cytoplasmic domain, (semaphorin) 4A	CORD10|FLJ12287|RP35|SEMAB|SEMB	1	Cytokines
SEMA4B	10509	sema domain, immunoglobulin domain (Ig), transmembrane domain (TM) and short cytoplasmic domain, (semaphorin) 4B	KIAA1745|MGC131831|SEMAC|SemC	15	Cytokines
SEMA4C	54910	sema domain, immunoglobulin domain (Ig), transmembrane domain (TM) and short cytoplasmic domain, (semaphorin) 4C	FLJ20369|KIAA1739|M-SEMA-F|MGC126382|MGC126383|SEMACL1|SEMAF|SEMAI	2	Cytokines
SEMA4D	10507	sema domain, immunoglobulin domain (Ig), transmembrane domain (TM) and short cytoplasmic domain, (semaphorin) 4D	C9orf164|CD100|FLJ33485|FLJ34282|FLJ39737|FLJ46484|M-sema-G|MGC169138|MGC169141|SEMAJ|coll-4	9	Cytokines
SEMA4F	10505	sema domain, immunoglobulin domain (Ig), transmembrane domain (TM) and short	M-SEMA|PRO2353|SEMAM|SEMAW|m-Sema-M	2	Cytokines

		cytoplasmic domain, (semaphorin) 4F			
SEMA4G	57715	sema domain, immunoglobulin domain (Ig), transmembrane domain (TM) and short cytoplasmic domain, (semaphorin) 4G	FLJ20590|KIAA1619|MGC102867	10	Cytokines
SEMA5A	9037	sema domain, seven thrombospondin repeats (type 1 and type 1-like), transmembrane domain (TM) and short cytoplasmic domain, (semaphorin) 5A	FLJ12815|SEMAF|semF	5	Cytokines
SEMA5B	54437	sema domain, seven thrombospondin repeats (type 1 and type 1-like), transmembrane domain (TM) and short cytoplasmic domain, (semaphorin) 5B	FLJ10372|KIAA1445|SEMAG|SemG	3	Cytokines
SEMA6A	57556	sema domain, transmembrane domain (TM), and cytoplasmic domain, (semaphorin) 6A	HT018|KIAA1368|SEMA|SEMA6A1|SEMAQ|VIA	5	Cytokines
SEMA6B	10501	sema domain, transmembrane domain (TM), and cytoplasmic domain, (semaphorin) 6B	SEM-SEMA-Y|SEMA-VIB|SEMAN|semaZ	19	Cytokines

SEMA6C	10500	sema domain, transmembrane domain (TM), and cytoplasmic domain, (semaphorin) 6C	SEMAY|m-SemaY|m-SemaY2	1	Cytokines
SEMA6D	80031	sema domain, transmembrane domain (TM), and cytoplasmic domain, (semaphorin) 6D	FLJ11598|KIAA1479	15	Cytokines
SEMA7A	8482	semaphorin 7A, GPI membrane anchor (John Milton Hagen blood group)	CD108|CDw108|H-SEMA-K1|H-Sema-L|JMH|MGC126692|MGC126696|SEMAK1|SEMAL	15	Cytokines
SLIT1	6585	slit homolog 1 (Drosophila)	MEGF4|MGC164811|SLIL1|SLIT3|Slit-1	10	Cytokines
SLIT2	9353	slit homolog 2 (Drosophila)	FLJ14420|SLIL3|Slit-2	4	Cytokines
SLURP1	57152	secreted LY6/PLAUR domain containing 1	ANUP|ARS|ArsB|LY6LS|MDM	8	Cytokines
SPP1	6696	secreted phosphoprotein 1	BNSP|BSPI|ETA-1|MGC110940|OPN	4	Cytokines
SST	6750	somatostatin	SMST	3	Cytokines
STC1	6781	stanniocalcin 1	STC	8	Cytokines
STC2	8614	stanniocalcin 2	STC-2|STCRP	5	Cytokines
TAC1	6863	tachykinin, precursor 1	Hs.2563|NK2|NKNA|NPK|TAC2	7	Cytokines
TDGF1	6997	teratocarcinoma-derived growth factor 1	CR|CRGF|CRIPTO|Cripto-1	3	Cytokines
TDGF3	6998	teratocarcinoma-derived growth factor 3, pseudogene	CR-3|CRIPTO|CRIPTO-3|TDGF1|TDGF2	X	Cytokines
TG	7038	thyroglobulin	AITD3|TGN	8	Cytokines
TGFA	7039	transforming growth factor, alpha	TFGA	2	Cytokines

TGFB1	7040	transforming growth factor, beta 1	CED|DPD1|TGFB|TGFbeta	19	Cytokines
TGFB2	7042	transforming growth factor, beta 2	MGC116892|TGF-beta2	1	Cytokines
TGFB3	7043	transforming growth factor, beta 3	ARVD|FLJ16571|TGF-beta3	14	Cytokines
THPO	7066	thrombopoietin	MGC163194|MGDF|MKCSF|ML|MPLLG|TPO	3	Cytokines
TNC	3371	tenascin C	HXB|MGC167029|TN	9	Cytokines
TNF	7124	tumor necrosis factor (TNF superfamily, member 2)	DIF|TNF-alpha|TNFA|TNFSF2	6	Cytokines
TNFRSF11B	4982	tumor necrosis factor receptor superfamily, member 11b	MGC29565|OCIF|OPG|TR1	8	Cytokines
TNFSF10	8743	tumor necrosis factor (ligand) superfamily, member 10	APO2L|Apo-2L|CD253|TL2|TRAIL	3	Cytokines
TNFSF11	8600	tumor necrosis factor (ligand) superfamily, member 11	CD254|ODF|OPGL|OPTB2|RANKL|TRANCE|hRANKL2|sOdf	13	Cytokines
TNFSF12	8742	tumor necrosis factor (ligand) superfamily, member 12	APO3L|DR3LG|MGC129581|MGC20669|TWEAK	17	Cytokines
TNFSF13	8741	tumor necrosis factor (ligand) superfamily, member 13	APRIL|CD256|TALL2|TRDL-1|UNQ383/PRO715|ligand	17	Cytokines
TNFSF13B	10673	tumor necrosis factor (ligand) superfamily, member 13b	BAFF|BLYS|CD257|DTL|TALL-1|TALL1|THANK|TNFSF20|ZTNF4	13	Cytokines
TNFSF14	8740	tumor necrosis factor (ligand) superfamily, member 14	CD258|HVEML|LIGHT|LTg|TR2	19	Cytokines
TNFSF15	9966	tumor necrosis factor (ligand) superfamily, member 15	MGC129934|MGC129935|TL1|TL1A|VEGI|VEGI192A	9	Cytokines

TNFSF18	8995	tumor necrosis factor (ligand) superfamily, member 18	AITRL|GITRL|MGC138237|TL6|hGITRL	1	Cytokines
TNFSF4	7292	tumor necrosis factor (ligand) superfamily, member 4	CD134L|CD252|GP34|OX-40L|OX4OL|TXGP1	1	Cytokines
TNFSF8	944	tumor necrosis factor (ligand) superfamily, member 8	CD153|CD30L|CD30LG|MGC138144	9	Cytokines
TNFSF9	8744	tumor necrosis factor (ligand) superfamily, member 9	4-1BB-L|CD137L	19	Cytokines
TOR2A	27433	torsin family 2, member A	FLJ14771|MGC99558|TORP1	9	Cytokines
TRH	7200	thyrotropin-releasing hormone	MGC125964|MGC125965	3	Cytokines
TSHB	7252	thyroid stimulating hormone, beta	CHNG4|TSH-BETA	1	Cytokines
TSLP	85480	thymic stromal lymphopoietin	-	5	Cytokines
TXLNA	200081	taxilin alpha	DKFZp451J0118|IL14|MGC118870|MGC118871|RP4-622L5.4|TXLN	1	Cytokines
TYMP	1890	thymidine phosphorylase	ECGF1|MNGIE|PDECGF|TP|hPD-ECGF	22	Cytokines
UCN	7349	urocortin	MGC129974|MGC129975|UI|UROC	2	Cytokines
UCN2	90226	urocortin 2	SRP|UCN-II|UCNI|UR|URP	3	Cytokines
UCN3	114131	urocortin 3 (stresscopin)	MGC119002|SCP|SPC|UCNIII	10	Cytokines
UTS2	10911	urotensin 2	PRO1068|U-II|UCN2|UII	1	Cytokines
UTS2D	257313	urotensin 2 domain containing	MGC138371|U2B|URP	3	Cytokines
VEGFA	7422	vascular endothelial growth factor A	MGC70609|MVCD1|VEGF|VEGF-A|VPF	6	Cytokines
VEGFB	7423	vascular endothelial growth factor B	VEGFL|VRF	11	Cytokines

VEGFC	7424	vascular endothelial growth factor C	Flt4-L|VRP	4	Cytokines
VGF	7425	VGF nerve growth factor inducible	-	7	Cytokines
VIP	7432	vasoactive intestinal peptide	MGC13587|PHM27	6	Cytokines
XCL1	6375	chemokine (C motif) ligand 1	ATAC|LPTN|LTN|SCM-1|SCM-1a|SCM1|SCYC1	1	Cytokines
XCL2	6846	chemokine (C motif) ligand 2	SCM-1b|SCM1B|SCYC2	1	Cytokines
ACVR1B	91	activin A receptor, type IB	ACTRIB|ACVRLK4|ALK4|SKR2	12	Cytokine_Receptors
ACVR1C	130399	activin A receptor, type IC	ACVRLK7|ALK7	2	Cytokine_Receptors
ACVR2A	92	activin A receptor, type IIA	ACTRII|ACVR2	2	Cytokine_Receptors
ACVR2B	93	activin A receptor, type IIB	ACTRIIB|ActR-IIB|MGC116908	3	Cytokine_Receptors
ACVRL1	94	activin A receptor type II-like 1	ACVRLK1|ALK-1|ALK1|HHT|HHT2|ORW2|SKR3|TSR-I	12	Cytokine_Receptors
ADCYAP1R1	117	adenylate cyclase activating polypeptide 1 (pituitary) receptor type I	PAC1|PACAPR|PACAPRI	7	Cytokine_Receptors
ADIPOR1	51094	adiponectin receptor 1	ACDCR1|CGI-45|CGI45|FLJ25385|FLJ42464|PAQR1|TESBP1A	1	Cytokine_Receptors
ADIPOR2	79602	adiponectin receptor 2	ACDCR2|FLJ21432|MGC4640|PAQR2	12	Cytokine_Receptors
ADRB1	153	adrenergic, beta-1-, receptor	ADRB1R|B1AR|BETA1AR|RHR	10	Cytokine_Receptors
ADRB2	154	adrenergic, beta-2-, receptor, surface	ADRB2R|ADRBR|B2AR|BAR|BETA2AR	5	Cytokine_Receptors
AGTR1	185	angiotensin II receptor, type 1	AG2S|AGTR1A|AGTR1B|AT1|AT1B|AT1R|AT2R1|AT2R1A|AT2R1B|HAT1R	3	Cytokine_Receptors
AGTR2	186	angiotensin II receptor, type 2	AT2|ATGR2|MRX88	X	Cytokine_Receptors

AMHR2	269	anti-Mullerian hormone receptor, type II	AMHR|MISR2|MISRII	12	Cytokine_Receptors
ANGPT1	284	angiopoietin 1	AGP1|AGPT|ANG1	8	Cytokine_Receptors
ANGPT4	51378	angiopoietin 4	AGP4|ANG-3|ANG4|MGC138181|MGC138183	20	Cytokine_Receptors
ANGPTL1	9068	angiopoietin-like 1	ANG3|ANGPT3|ARP1|AngY|KIAA0351|UNQ162|dJ595C2.2	1	Cytokine_Receptors
ANGPTL2	23452	angiopoietin-like 2	ARP2|HARP|MGC8889	9	Cytokine_Receptors
ANGPTL3	27329	angiopoietin-like 3	ANGPT5	1	Cytokine_Receptors
ANGPTL4	51129	angiopoietin-like 4	ANGPTL2|ARP4|FIAF|HFARP|NL2|PGAR|pp1158	19	Cytokine_Receptors
ANGPTL6	83854	angiopoietin-like 6	AGF|ARP5	19	Cytokine_Receptors
APLNR	187	apelin receptor	AGTRL1|APJ|APJR|FLJ90771|MGC45246	11	Cytokine_Receptors
AR	367	androgen receptor	AIS|DHTR|HUMARA|HYSP1|KD|NR3C4|SBMA|SMAX1|TFM	X	Cytokine_Receptors
AVPR1A	552	arginine vasopressin receptor 1A	AVPR1	12	Cytokine_Receptors
AVPR1B	553	arginine vasopressin receptor 1B	AVPR3	1	Cytokine_Receptors
AVPR2	554	arginine vasopressin receptor 2	ADHR|DI1|DIR|DIR3|MGC126533|MGC138386|NDI|V2R	X	Cytokine_Receptors
BMPR1A	657	bone morphogenetic protein receptor, type IA	10q23del|ACVRLK3|ALK3|CD292|SKR5	10	Cytokine_Receptors
BMPR1B	658	bone morphogenetic protein receptor, type IB	ALK-6|ALK6|CDw293	4	Cytokine_Receptors

BMPR2	659	bone morphogenetic protein receptor, type II (serine/threonine kinase)	BMPR-II|BMPR3|BMR2|BRK-3|FLJ41585|FLJ76945|PPH1|T-ALK	2	Cytokine_Receptors
BRD8	10902	bromodomain containing 8	SMAP|SMAP2|p120	5	Cytokine_Receptors
C3AR1	719	complement component 3a receptor 1	AZ3B|C3AR|HNFAG09	12	Cytokine_Receptors
C5AR1	728	complement component 5a receptor 1	C5A|C5AR|C5R1|CD88	19	Cytokine_Receptors
CALCR	799	calcitonin receptor	CRT|CTR|CTR1	7	Cytokine_Receptors
CALCRL	10203	calcitonin receptor-like	CGRPR|CRLR	2	Cytokine_Receptors
CCBP2	1238	chemokine binding protein 2	CCR10|CCR9|CMKBR9|D6|MGC126678|MGC138250|hD6	3	Cytokine_Receptors
CCR1	1230	chemokine (C-C motif) receptor 1	CD191|CKR-1|CKR1|CMKBR1|HM145|MIP1aR|SCYAR1	3	Cytokine_Receptors
CCR10	2826	chemokine (C-C motif) receptor 10	GPR2	17	Cytokine_Receptors
CCR3	1232	chemokine (C-C motif) receptor 3	CC-CKR-3|CD193|CKR3|CMKBR3|MGC102841	3	Cytokine_Receptors
CCR4	1233	chemokine (C-C motif) receptor 4	CC-CKR-4|CD194|CKR4|CMKBR4|ChemR13|HGCN:14099|K5-5|MGC88293	3	Cytokine_Receptors
CCR5	1234	chemokine (C-C motif) receptor 5	CC-CKR-5|CCCKR5|CD195|CKR-5|CKR5|CMKBR5|FLJ78003|IDDM22	3	Cytokine_Receptors
CCR6	1235	chemokine (C-C motif) receptor 6	BN-1|CD196|CKR-L3|CKR6|CKRL3|CMKBR6|DCR2|DRY-6|GPR-CY4|GPR29|GPRCY4|STRL22	6	Cytokine_Receptors

CCR7	1236	chemokine (C-C motif) receptor 7	BLR2|CD197|CDw197|CMKBR7|EBI1	17	Cytokine_Receptors
CCR8	1237	chemokine (C-C motif) receptor 8	CDw198|CKR-L1|CKRL1|CMKBR8|CMKBRL2|CY6|GPR-CY6|MGC129966|MGC129973|TER1	3	Cytokine_Receptors
CCR9	10803	chemokine (C-C motif) receptor 9	CDw199|GPR-9-6|GPR28	3	Cytokine_Receptors
CCRL1	51554	chemokine (C-C motif) receptor-like 1	CC-CKR-11|CCBP2|CCR10|CCR11|CCX-CKR|CKR-11|PPR1|VSHK1	3	Cytokine_Receptors
CCRL2	9034	chemokine (C-C motif) receptor-like 2	CKRX|CRAM-A|CRAM-B|FLJ55815|HCR|MGC116710|MGC34104	3	Cytokine_Receptors
CD40	958	CD40 molecule, TNF receptor superfamily member 5	Bp50|CDW40|MGC9013|TNFRSF5|p50	20	Cytokine_Receptors
CMKLR1	1240	chemokine-like receptor 1	CHEMERINR|ChemR23|DEZ|MGC126105|MGC126106	12	Cytokine_Receptors
CNTFR	1271	ciliary neurotrophic factor receptor	MGC1774	9	Cytokine_Receptors
CRHR1	1394	corticotropin releasing hormone receptor 1	CRF-R|CRF1|CRFR1|CRH-R1h|CRHR|CRHR1f	17	Cytokine_Receptors
CRHR2	1395	corticotropin releasing hormone receptor 2	CRFR2	7	Cytokine_Receptors
CRIM1	51232	cysteine rich transmembrane BMP regulator 1 (chordin-like)	MGC138194|S52	2	Cytokine_Receptors
CRLF1	9244	cytokine receptor-like factor 1	CISS|CISS1|CLF|CLF-1|NR6	19	Cytokine_Receptors
CRLF2	64109	cytokine receptor-like factor 2	CRL2|CRLF2Y|TSLPR	X|Y	Cytokine_Receptors
CRLF3	51379	cytokine receptor-like factor 3	CREME9|CYTOR4|FRWS|MGC20661	17	Cytokine_Receptors

CSF1R	1436	colony stimulating factor 1 receptor	C-FMS|CD115|CSFR|FIM2|FMS	5	Cytokine_Receptors
CSF2RA	1438	colony stimulating factor 2 receptor, alpha, low-affinity (granulocyte-macrophage)	CD116|CDw116|CSF2R|CSF2RAX|CSF2RAY|CSF2RX|CSF2RY|GM-CSF-R-alpha|GMCSFR|GMR|MGC3848|MGC4838	X|Y	Cytokine_Receptors
CSF2RB	1439	colony stimulating factor 2 receptor, beta, low-affinity (granulocyte-macrophage)	CD131|CDw131|IL3RB|IL5RB	22	Cytokine_Receptors
CSF3R	1441	colony stimulating factor 3 receptor (granulocyte)	CD114|GCSFR	1	Cytokine_Receptors
CX3CR1	1524	chemokine (C-X3-C motif) receptor 1	CCRL1|CMKBRL1|CMKDR1|GPR13|GPRV28|V28	3	Cytokine_Receptors
CXCR3	2833	chemokine (C-X-C motif) receptor 3	CD182|CD183|CKR-L2|CMKAR3|GPR9|IP10-R|Mig-R|MigR	X	Cytokine_Receptors
CXCR4	7852	chemokine (C-X-C motif) receptor 4	CD184|D2S201E|FB22|HM89|HSY3RR|LAP3|LCR1|LESTR|NPY3R|NPYR|NPYRL|NPYY3R|WHIM	2	Cytokine_Receptors
CXCR5	643	chemokine (C-X-C motif) receptor 5	BLR1|CD185|MDR15|MGC117347	11	Cytokine_Receptors
CXCR6	10663	chemokine (C-X-C motif) receptor 6	BONZO|CD186|STRL33|TYMSTR	3	Cytokine_Receptors
CXCR7	57007	chemokine (C-X-C motif) receptor 7	CMKOR1|GPR159|RDC1	2	Cytokine_Receptors
CYSLTR1	10800	cysteinyl leukotriene receptor 1	CYSLT1|CYSLT1R|CYSLTR|HG55|HMTMF81|MGC46139	X	Cytokine_Receptors
CYSLTR2	57105	cysteinyl leukotriene receptor 2	CYSLT2|CYSLT2R|GPCR|HG57|HPN321|KPG_	13	Cytokine_Receptors

			011|PSEC0146|hGPCR21		
DARC	2532	Duffy blood group, chemokine receptor	CCBP1|CD234|Dfy|FY|GPD|GpFy|WBCQ1	1	Cytokine_Receptors
EDNRA	1909	endothelin receptor type A	ETA|ETRA	4	Cytokine_Receptors
EDNRB	1910	endothelin receptor type B	ABCDS|ETB|ETBR|ETRB|HSCR|HSCR2	13	Cytokine_Receptors
EGFR	1956	epidermal growth factor receptor (erythroblastic leukemia viral (v-erb-b) oncogene homolog, avian)	ERBB|ERBB1|HER1|PIG61|mENA	7	Cytokine_Receptors
ENG	2022	endoglin	CD105|END|FLJ41744|HHT1|ORW|ORW1	9	Cytokine_Receptors
EPOR	2057	erythropoietin receptor	MGC138358	19	Cytokine_Receptors
ESR1	2099	estrogen receptor 1	DKFZp686N23123|ER|ESR|ESRA|Era|NR3A1	6	Cytokine_Receptors
ESR2	2100	estrogen receptor 2 (ER beta)	ER-BETA|ESR-BETA|ESRB|ESTRB|Erb|NR3A2	14	Cytokine_Receptors
ESRRA	2101	estrogen-related receptor alpha	ERR1|ERRa|ERRalpha|ESRL1|NR3B1	11	Cytokine_Receptors
ESRRB	2103	estrogen-related receptor beta	DFNB35|ERR2|ERRb|ERRbeta|ERRbeta-2|ESRL2|NR3B2	14	Cytokine_Receptors
ESRRG	2104	estrogen-related receptor gamma	DKFZp781L1617|ERR3|FLJ16023|KIAA0832|NR3B3	1	Cytokine_Receptors
FGFR1	2260	fibroblast growth factor receptor 1	BFGFR|CD331|CEK|FGFBR|FLG|FLJ99988|FLT2|HBGFR|KAL2|N-SAM|OGD	8	Cytokine_Receptors
FGFR2	2263	fibroblast growth factor receptor 2	BEK|BFR-1|CD332|CEK3|CFD1|ECT1|FLJ98662|JWS|K-SAM|KGFR|TK14|TK25	10	Cytokine_Receptors
FGFR3	2261	fibroblast growth factor receptor 3	ACH|CD333|CEK2|HSFGFR3EX|JTK4	4	Cytokine_Receptors

FGFR4	2264	fibroblast growth factor receptor 4	CD334|JTK2|MGC20292|TKF	5	Cytokine_Receptors
FGFRL1	53834	fibroblast growth factor receptor-like 1	FGFR5|FHFR	4	Cytokine_Receptors
FLT1	2321	fms-related tyrosine kinase 1 (vascular endothelial growth factor/vascular permeability factor receptor)	FLT|VEGFR1	13	Cytokine_Receptors
FLT3	2322	fms-related tyrosine kinase 3	CD135|FLK2|STK1	13	Cytokine_Receptors
FLT4	2324	fms-related tyrosine kinase 4	FLT41|LMPH1A|PCL|VEGFR3	5	Cytokine_Receptors
FPR1	2357	formyl peptide receptor 1	FMLP|FPR	19	Cytokine_Receptors
FPR2	2358	formyl peptide receptor 2	ALXR|FMLP-R-II|FMLPX|FPR2A|FPRH1|FPRH2|FPRL1|HM63|LXA4R	19	Cytokine_Receptors
FPR2	2358	formyl peptide receptor 2	ALXR|FMLP-R-II|FMLPX|FPR2A|FPRH1|FPRH2|FPRL1|HM63|LXA4R	19	Cytokine_Receptors
FSHR	2492	follicle stimulating hormone receptor	FSHRO|LGR1|MGC141667|MGC141668|ODG1	2	Cytokine_Receptors
GALR2	8811	galanin receptor 2	GALNR2|MGC125983|MGC125984	17	Cytokine_Receptors
GALR3	8484	galanin receptor 3	-	22	Cytokine_Receptors
GCGR	2642	glucagon receptor	GGR|MGC138246	17	Cytokine_Receptors
GHR	2690	growth hormone receptor	GHBP	5	Cytokine_Receptors
GHRHR	2692	growth hormone releasing hormone receptor	GHRFR|GHRHRpsv|GRFR	7	Cytokine_Receptors

GHSR	2693	growth hormone secretagogue receptor	-	3	Cytokine_Receptors
GIPR	2696	gastric inhibitory polypeptide receptor	MGC126722	19	Cytokine_Receptors
GLP1R	2740	glucagon-like peptide 1 receptor	MGC138331	6	Cytokine_Receptors
GLP2R	9340	glucagon-like peptide 2 receptor	-	17	Cytokine_Receptors
GNRHR	2798	gonadotropin-releasing hormone receptor	GNRHR1|GRHR|LHRHR|LRHR	4	Cytokine_Receptors
GPER	2852	G protein-coupled estrogen receptor 1	CEPR|CMKRL2|DRY12|FEG-1|GPCR-Br|GPR30|LERGU|LERGU2|LyGPR|MGC99678	7	Cytokine_Receptors
GPR17	2840	G protein-coupled receptor 17	DKFZp686M18273	2	Cytokine_Receptors
GPR32	2854	G protein-coupled receptor 32	-	19	Cytokine_Receptors
GPR33	2856	G protein-coupled receptor 33 (gene/pseudogene)	-	14	Cytokine_Receptors
GPR44	11251	G protein-coupled receptor 44	CD294|CRTH2|DP2	11	Cytokine_Receptors
GPR77	27202	G protein-coupled receptor 77	C5L2|GPF77	19	Cytokine_Receptors
HNF4A	3172	hepatocyte nuclear factor 4, alpha	FLJ39654|HNF4|HNF4a7|HNF4a8|HNF4a9|HNF4alpha|MODY|MODY1|NR2A1|NR2A21|TCF|TCF14	20	Cytokine_Receptors
HNF4G	3174	hepatocyte nuclear factor 4, gamma	NR2A2|NR2A3	8	Cytokine_Receptors
HTR3A	3359	5-hydroxytryptamine (serotonin) receptor 3A	5-HT-3|5-HT3A|5-HT3R|5HT3R|HTR3	11	Cytokine_Receptors
HTR3B	9177	5-hydroxytryptamine (serotonin) receptor 3B	5-HT3B	11	Cytokine_Receptors

HTR3C	170572	5-hydroxytryptamine (serotonin) receptor 3, family member C	-	3	Cytokine_Receptors
HTR3D	200909	5-hydroxytryptamine (serotonin) receptor 3 family member D	MGC119636|MGC119637	3	Cytokine_Receptors
HTR3E	285242	5-hydroxytryptamine (serotonin) receptor 3, family member E	5-HT3c1|MGC120035|MGC120036|MGC120037	3	Cytokine_Receptors
IFNAR1	3454	interferon (alpha, beta and omega) receptor 1	AVP|IFN-alpha-REC|IFNAR|IFNBR|IFRC	21	Cytokine_Receptors
IFNAR2	3455	interferon (alpha, beta and omega) receptor 2	IFN-R|IFN-alpha-REC|IFNABR|IFNARB	21	Cytokine_Receptors
IFNGR1	3459	interferon gamma receptor 1	CD119|FLJ45734|IFNGR	6	Cytokine_Receptors
IFNGR2	3460	interferon gamma receptor 2 (interferon gamma transducer 1)	AF-1|IFGR2|IFNGT1	21	Cytokine_Receptors
IGF1R	3480	insulin-like growth factor 1 receptor	CD221|IGFIR|IGFR|JTK13|MGC142170|MGC142172|MGC18216	15	Cytokine_Receptors
IGF2R	3482	insulin-like growth factor 2 receptor	CD222|CIMPR|M6P-R|MPR1|MPRI	6	Cytokine_Receptors
IL10RA	3587	interleukin 10 receptor, alpha	CDW210A|HIL-10R|IL-10R1|IL10R	11	Cytokine_Receptors
IL10RB	3588	interleukin 10 receptor, beta	CDW210B|CRF2-4|CRFB4|D21S58|D21S66|IL-10R2	21	Cytokine_Receptors
IL11RA	3590	interleukin 11 receptor, alpha	MGC2146	9	Cytokine_Receptors
IL11RB	3591	interleukin 11 receptor, beta	-	-	Cytokine_Receptors
IL12RB1	3594	interleukin 12 receptor, beta 1	CD212|IL-12R-BETA1|IL12RB|MGC34454	19	Cytokine_Receptors
IL12RB2	3595	interleukin 12 receptor, beta 2	-	1	Cytokine_Receptors
IL13RA1	3597	interleukin 13 receptor, alpha 1	CD213A1|IL-13Ra|NR4	X	Cytokine_Receptors
IL13RA2	3598	interleukin 13 receptor, alpha 2	CD213A2|CT19|IL-13R|IL13BP	X	Cytokine_Receptors

IL15RA	3601	interleukin 15 receptor, alpha	MGC104179	10	Cytokine_Receptors
IL15RB	3602	interleukin 15 receptor, beta	-	-	Cytokine_Receptors
IL17RA	23765	interleukin 17 receptor A	CD217|CDw217|IL-17RA|IL17R|MGC10262|hIL-17R	22	Cytokine_Receptors
IL17RB	55540	interleukin 17 receptor B	CRL4|EVI27|IL17BR|IL17RH1|MGC5245	3	Cytokine_Receptors
IL17RC	84818	interleukin 17 receptor C	FLJ95963|FLJ96005|IL17-RL|IL17RL|MGC10763	3	Cytokine_Receptors
IL17RD	54756	interleukin 17 receptor D	DKFZp434N1928|FLJ35755|IL-17RD|IL17RLM|MGC133309|SEF	3	Cytokine_Receptors
IL17RE	132014	interleukin 17 receptor E	FLJ23658|MGC71884	3	Cytokine_Receptors
IL18R1	8809	interleukin 18 receptor 1	CD218a|CDw218a|IL-1Rrp|IL18RA|IL1RRP	2	Cytokine_Receptors
IL18RAP	8807	interleukin 18 receptor accessory protein	ACPL|CD218b|CDw218b|IL18RB|MGC120589|MGC120590	2	Cytokine_Receptors
IL1R1	3554	interleukin 1 receptor, type I	CD121A|D2S1473|IL-1R-alpha|IL1R|IL1RA|P80	2	Cytokine_Receptors
IL1R2	7850	interleukin 1 receptor, type II	CD121b|IL1RB|MGC47725	2	Cytokine_Receptors
IL1RAP	3556	interleukin 1 receptor accessory protein	C3orf13|FLJ37788|IL-1RAcP|IL1R3	3	Cytokine_Receptors
IL1RL1	9173	interleukin 1 receptor-like 1	DER4|FIT-1|MGC32623|ST2|ST2L|ST2V|T1	2	Cytokine_Receptors
IL1RL2	8808	interleukin 1 receptor-like 2	IL1R-rp2|IL1RRP2	2	Cytokine_Receptors
IL20RA	53832	interleukin 20 receptor, alpha	FLJ40993|IL-20R1|ZCYTOR7	6	Cytokine_Receptors
IL20RB	53833	interleukin 20 receptor beta	DIRS1|FNDC6|IL-20R2|MGC34923	3	Cytokine_Receptors
IL21R	50615	interleukin 21 receptor	MGC10967|NILR	16	Cytokine_Receptors
IL22RA1	58985	interleukin 22 receptor, alpha 1	CRF2-9|IL22R|IL22R1	1	Cytokine_Receptors
IL22RA2	116379	interleukin 22 receptor, alpha 2	CRF2-10|CRF2-S1|CRF2X|IL-22BP|MGC150509|MGC150510	6	Cytokine_Receptors
IL23R	149233	interleukin 23 receptor	-	1	Cytokine_Receptors

IL27RA	9466	interleukin 27 receptor, alpha	CRL1|IL27R|TCCR|WSX1|zcytor1	19	Cytokine_Receptors
IL28RA	163702	interleukin 28 receptor, alpha (interferon, lambda receptor)	CRF2/12|IFNLR|IFNLR1|IL-28R1|LICR2	1	Cytokine_Receptors
IL2RA	3559	interleukin 2 receptor, alpha	CD25|IDDM10|IL2R|TCGFR	10	Cytokine_Receptors
IL2RB	3560	interleukin 2 receptor, beta	CD122|P70-75	22	Cytokine_Receptors
IL2RG	3561	interleukin 2 receptor, gamma (severe combined immunodeficiency)	CD132|IMD4|SCIDX|SCIDX1	X	Cytokine_Receptors
IL31RA	133396	interleukin 31 receptor A	CRL|CRL3|GLM-R|GLMR|GPL|IL-31RA|MGC125346|PRO21384	5	Cytokine_Receptors
IL3RA	3563	interleukin 3 receptor, alpha (low affinity)	CD123|IL3R|IL3RAY|IL3RX|IL3RY|MGC34174|hIL-3Ra	X|Y	Cytokine_Receptors
IL4R	3566	interleukin 4 receptor	CD124|IL4RA	16	Cytokine_Receptors
IL5RA	3568	interleukin 5 receptor, alpha	CD125|CDw125|HSIL5R3|IL5R|MGC26560	3	Cytokine_Receptors
IL6R	3570	interleukin 6 receptor	CD126|IL-6R-1|IL-6R-alpha|IL6RA|MGC104991	1	Cytokine_Receptors
IL7R	3575	interleukin 7 receptor	CD127|CDW127|IL-7R-alpha|IL7RA|ILRA	5	Cytokine_Receptors
IL8RA	3577	interleukin 8 receptor, alpha	C-C|C-C-CKR-1|CD128|CD181|CDw128a|CKR-1|CMKAR1|CXCR1|IL8R1|IL8RBA	2	Cytokine_Receptors
IL8RB	3579	interleukin 8 receptor, beta	CD182|CDw128b|CMKAR2|CXCR2|IL8R2|IL8RA	2	Cytokine_Receptors
IL9R	3581	interleukin 9 receptor	CD129	X|Y	Cytokine_Receptors
INSR	3643	insulin receptor	CD220|HHF5	19	Cytokine_Receptors
KDR	3791	kinase insert domain receptor (a type III receptor tyrosine kinase)	CD309|FLK1|VEGFR|VEGFR2	4	Cytokine_Receptors
LEPR	3953	leptin receptor	CD295|OBR	1	Cytokine_Receptors

LGR4	55366	leucine-rich repeat-containing G protein-coupled receptor 4	GPR48	11	Cytokine_Receptors
LGR5	8549	leucine-rich repeat-containing G protein-coupled receptor 5	FEX|GPR49|GPR67|GRP49|HG38|MGC117008	12	Cytokine_Receptors
LGR6	59352	leucine-rich repeat-containing G protein-coupled receptor 6	FLJ14471|GPCR|VTS20631	1	Cytokine_Receptors
LHCGR	3973	luteinizing hormone/choriogonadotropin receptor	FLJ41504|LCGR|LGR2|LH/CG-R|LH/CGR|LHR|LHRHR|LSH-R	2	Cytokine_Receptors
LIFR	3977	leukemia inhibitory factor receptor alpha	CD118|FLJ98106|FLJ99923|LIF-R|SJS2|STWS|SWS	5	Cytokine_Receptors
LTB4R	1241	leukotriene B4 receptor	BLT1|BLTR|CMKRL1|GPR16|LTB4R1|LTBR1|P2RY7|P2Y7	14	Cytokine_Receptors
LTB4R2	56413	leukotriene B4 receptor 2	BLT2|BLTR2|JULF2|KPG_004|NOP9	14	Cytokine_Receptors
LTBR	4055	lymphotoxin beta receptor (TNFR superfamily, member 3)	CD18|D12S370|LT-BETA-R|TNF-R-III|TNFCR|TNFR-RP|TNFR2-RP|TNFRSF3	12	Cytokine_Receptors
MC1R	4157	melanocortin 1 receptor (alpha melanocyte stimulating hormone receptor)	MGC14337|MSH-R|SHEP2	16	Cytokine_Receptors
MC2R	4158	melanocortin 2 receptor (adrenocorticotropic hormone)	ACTHR|MGC125798	18	Cytokine_Receptors
MC3R	4159	melanocortin 3 receptor	BMIQ9|MC3|MC3-R|OB20|OQTL	20	Cytokine_Receptors
MC4R	4160	melanocortin 4 receptor	MGC126851|MGC138197	18	Cytokine_Receptors
MCHR1	2847	melanin-concentrating hormone receptor 1	GPR24|MCH1R|MGC32129|SLC1	22	Cytokine_Receptors

MCHR2	84539	melanin-concentrating hormone receptor 2	GPR145|MCH2|MCH2R|SLT	6	Cytokine_Receptors
MET	4233	met proto-oncogene (hepatocyte growth factor receptor)	AUTS9|HGFR|RCCP2|c-Met	7	Cytokine_Receptors
MLNR	2862	motilin receptor	GPR38|MTLR1	13	Cytokine_Receptors
MPL	4352	myeloproliferative leukemia virus oncogene	C-MPL|CD110|MPLV|TPOR	1	Cytokine_Receptors
MTNR1A	4543	melatonin receptor 1A	MEL-1A-R|MT1	4	Cytokine_Receptors
MTNR1B	4544	melatonin receptor 1B	MEL-1B-R|MT2	11	Cytokine_Receptors
NGFR	4804	nerve growth factor receptor (TNFR superfamily, member 16)	CD271|Gp80-LNGFR|TNFRSF16|p75(NTR)|p75NTR	17	Cytokine_Receptors
NMBR	4829	neuromedin B receptor	-	6	Cytokine_Receptors
NPR1	4881	natriuretic peptide receptor A/guanylate cyclase A (atrionatriuretic peptide receptor A)	ANPRA|ANPa|GUC2A|GUCY2A|NPRA	1	Cytokine_Receptors
NPR3	4883	natriuretic peptide receptor C/guanylate cyclase C (atrionatriuretic peptide receptor C)	ANPRC|GUCY2B|NPRC	5	Cytokine_Receptors
NR0B1	190	nuclear receptor subfamily 0, group B, member 1	AHC|AHCH|AHX|DAX-1|DAX1|DSS|GTD|HHG|NROB1	X	Cytokine_Receptors
NR0B2	8431	nuclear receptor subfamily 0, group B, member 2	FLJ17090|SHP|SHP1	1	Cytokine_Receptors
NR1D1	9572	nuclear receptor subfamily 1, group D, member 1	EAR1|THRA1|THRAL|ear-1|hRev	17	Cytokine_Receptors

NR1D2	9975	nuclear receptor subfamily 1, group D, member 2	BD73|EAR-1R|RVR	3	Cytokine_Receptors
NR1H2	7376	nuclear receptor subfamily 1, group H, member 2	LXR-b|LXRB|NER|NER-I|RIP15|UNR	19	Cytokine_Receptors
NR1H3	10062	nuclear receptor subfamily 1, group H, member 3	LXR-a|LXRA|RLD-1	11	Cytokine_Receptors
NR1H4	9971	nuclear receptor subfamily 1, group H, member 4	BAR|FXR|HRR-1|HRR1|MGC163445|RIP14	12	Cytokine_Receptors
NR1I2	8856	nuclear receptor subfamily 1, group I, member 2	BXR|ONR1|PAR|PAR1|PAR2|PARq|PRR|PXR|SAR|SXR	3	Cytokine_Receptors
NR1I3	9970	nuclear receptor subfamily 1, group I, member 3	CAR|CAR1|MB67|MGC150433|MGC97144|MGC97209	1	Cytokine_Receptors
NR2C1	7181	nuclear receptor subfamily 2, group C, member 1	TR2	12	Cytokine_Receptors
NR2C2	7182	nuclear receptor subfamily 2, group C, member 2	TAK1|TR2R1|TR4|hTAK1	3	Cytokine_Receptors
NR2E1	7101	nuclear receptor subfamily 2, group E, member 1	TLL|TLX|XTLL	6	Cytokine_Receptors
NR2E3	10002	nuclear receptor subfamily 2, group E, member 3	ESCS|MGC49976|PNR|RNR|RP37|rd7	15	Cytokine_Receptors
NR2F1	7025	nuclear receptor subfamily 2, group F, member 1	COUP-TFI|EAR-3|EAR3|ERBAL3|NR2F2|SVP44|TCFCOUP1|TFCOUP1	5	Cytokine_Receptors
NR2F2	7026	nuclear receptor subfamily 2, group F, member 2	ARP1|COUP-TFII|COUPTFB|MGC117452|SVP40|TFCOUP2	15	Cytokine_Receptors

NR2F6	2063	nuclear receptor subfamily 2, group F, member 6	EAR-2|EAR2|ERBAL2	19	Cytokine_Receptors
NR3C1	2908	nuclear receptor subfamily 3, group C, member 1 (glucocorticoid receptor)	GCCR|GCR|GR|GRL	5	Cytokine_Receptors
NR3C2	4306	nuclear receptor subfamily 3, group C, member 2	MCR|MGC133092|MLR|MR	4	Cytokine_Receptors
NR4A1	3164	nuclear receptor subfamily 4, group A, member 1	GFRP1|HMR|MGC9485|N10|NAK-1|NGFIB|NP10|NUR77|TR3	12	Cytokine_Receptors
NR4A2	4929	nuclear receptor subfamily 4, group A, member 2	HZF-3|NOT|NURR1|RNR1|TINUR	2	Cytokine_Receptors
NR4A3	8013	nuclear receptor subfamily 4, group A, member 3	CHN|CSMF|MINOR|NOR1|TEC	9	Cytokine_Receptors
NR5A1	2516	nuclear receptor subfamily 5, group A, member 1	AD4BP|ELP|FTZ1|FTZF1|SF-1|SF1	9	Cytokine_Receptors
NR5A2	2494	nuclear receptor subfamily 5, group A, member 2	B1F|B1F2|CPF|FTF|FTZ-F1|FTZ-F1beta|LRH-1|hB1F|hB1F-2	1	Cytokine_Receptors
NR6A1	2649	nuclear receptor subfamily 6, group A, member 1	GCNF|GCNF1|NR61|RTR	9	Cytokine_Receptors
NRP1	8829	neuropilin 1	BDCA4|CD304|DKFZp686A03134|DKFZp781F1414|NP1|NRP|VEGF165R	10	Cytokine_Receptors
NRP2	8828	neuropilin 2	MGC126574|NP2|NPN2|PRO2714|VEGF165R2	2	Cytokine_Receptors
OGFR	11054	opioid growth factor receptor	-	20	Cytokine_Receptors
OPRD1	4985	opioid receptor, delta 1	OPRD	1	Cytokine_Receptors
OPRK1	4986	opioid receptor, kappa 1	KOR|OPRK	8	Cytokine_Receptors
OPRL1	4987	opiate receptor-like 1	KOR-3|MGC34578|NOCIR|OOR|ORL1	20	Cytokine_Receptors

OPRM1	4988	opioid receptor, mu 1	KIAA0403|LMOR|MOR|MOR1|OPRM	6	Cytokine_Receptors
OSMR	9180	oncostatin M receptor	MGC150626|MGC150627|MGC75127|OSMRB	5	Cytokine_Receptors
OXTR	5021	oxytocin receptor	OT-R	3	Cytokine_Receptors
PGR	5241	progesterone receptor	NR3C3|PR	11	Cytokine_Receptors
PGRMC2	10424	progesterone receptor membrane component 2	DG6|PMBP	4	Cytokine_Receptors
PLAUR	5329	plasminogen activator, urokinase receptor	CD87|UPAR|URKR	19	Cytokine_Receptors
PLXNA1	5361	plexin A1	NOV|NOVP|PLEXIN-A1|PLXN1	3	Cytokine_Receptors
PLXNA2	5362	plexin A2	FLJ11751|FLJ30634|KIAA0463|OCT|PLXN2	1	Cytokine_Receptors
PLXNA3	55558	plexin A3	6.3|HSSEXGENE|PLEXIN-A3|PLXN3|PLXN4|SEX|XAP-6	X	Cytokine_Receptors
PLXNA4	91584	plexin A4	DKFZp434G0625|DKFZp566O0546|FAYV2820|FLJ35026|FLJ38287|KIAA1550|PLEXA4|PLXNA4A|PLXNA4B|PRO34003	7	Cytokine_Receptors
PLXNB1	5364	plexin B1	KIAA0407|MGC149167|PLEXIN-B1|PLXN5|SEP	3	Cytokine_Receptors
PLXNB2	23654	plexin B2	KIAA0315|MM1|Nbla00445|PLEXB2|dJ402G11.3	22	Cytokine_Receptors
PLXNB3	5365	plexin B3	FLJ76953|PLEXB3|PLEXR|PLXN6	X	Cytokine_Receptors
PLXNC1	10154	plexin C1	CD232|PLXN-C1|VESPR	12	Cytokine_Receptors
PLXND1	23129	plexin D1	KIAA0620|MGC75353|PLEXD1	3	Cytokine_Receptors
PPARA	5465	peroxisome proliferator-activated receptor alpha	MGC2237|MGC2452|NR1C1|PPAR|hPPAR	22	Cytokine_Receptors
PPARD	5467	peroxisome proliferator-activated receptor delta	FAAR|MGC3931|NR1C2|NUC1|NUCI|NUCII|PPAR-beta|PPARB	6	Cytokine_Receptors

PPARG	5468	peroxisome proliferator-activated receptor gamma	CIMT1|NR1C3|PPARG1|PPARG2|PPARgamma	3	Cytokine_Receptors
PRLHR	2834	prolactin releasing hormone receptor	GPR10|GR3|MGC126539|MGC126541|PrRPR	10	Cytokine_Receptors
PRLR	5618	prolactin receptor	hPRLrI	5	Cytokine_Receptors
PTAFR	5724	platelet-activating factor receptor	PAFR	1	Cytokine_Receptors
PTGDR	5729	prostaglandin D2 receptor (DP)	AS1|ASRT1|DP|DP1|MGC49004	14	Cytokine_Receptors
PTGDS	5730	prostaglandin D2 synthase 21kDa (brain)	LPGDS|PDS|PGD2|PGDS|PGDS2	9	Cytokine_Receptors
PTGER1	5731	prostaglandin E receptor 1 (subtype EP1), 42kDa	EP1	19	Cytokine_Receptors
PTGER2	5732	prostaglandin E receptor 2 (subtype EP2), 53kDa	EP2	14	Cytokine_Receptors
PTGER3	5733	prostaglandin E receptor 3 (subtype EP3)	EP3|EP3-I|EP3-II|EP3-III|EP3-IV|EP3e|MGC141828|MGC141829|MGC27302	1	Cytokine_Receptors
PTGER4	5734	prostaglandin E receptor 4 (subtype EP4)	EP4|EP4R|MGC126583	5	Cytokine_Receptors
PTGFR	5737	prostaglandin F receptor (FP)	FP|MGC120498|MGC46203	1	Cytokine_Receptors
PTH1R	5745	parathyroid hormone 1 receptor	MGC138426|MGC138452|PFE|PTHR|PTHR1	3	Cytokine_Receptors
PTH2R	5746	parathyroid hormone 2 receptor	PTHR2	2	Cytokine_Receptors
RARA	5914	retinoic acid receptor, alpha	NR1B1|RAR	17	Cytokine_Receptors
RARB	5915	retinoic acid receptor, beta	HAP|NR1B2|RRB2	3	Cytokine_Receptors
RARG	5916	retinoic acid receptor, gamma	NR1B3|RARC	12	Cytokine_Receptors
ROBO1	6091	roundabout, axon guidance receptor, homolog 1 (Drosophila)	DUTT1|FLJ21882|MGC131599|MGC133277|SAX3	3	Cytokine_Receptors

ROBO2	6092	roundabout, axon guidance receptor, homolog 2 (Drosophila)	KIAA1568|SAX3	3	Cytokine_Receptors
ROBO3	64221	roundabout, axon guidance receptor, homolog 3 (Drosophila)	FLJ21044|HGPPS|HGPS|RBIG1|RIG1	11	Cytokine_Receptors
RORA	6095	RAR-related orphan receptor A	DKFZp686M2414|MGC119326|MGC119329|NR1F1|ROR1|ROR2|ROR3|RZR-ALPHA|RZRA	15	Cytokine_Receptors
RORB	6096	RAR-related orphan receptor B	NR1F2|ROR-BETA|RZR-BETA|RZRB|bA133M9.1	9	Cytokine_Receptors
RORC	6097	RAR-related orphan receptor C	MGC129539|NR1F3|RORG|RZR-GAMMA|RZRG|TOR	1	Cytokine_Receptors
RXFP1	59350	relaxin/insulin-like family peptide receptor 1	LGR7|LGR7.1|LGR7.10|LGR7.2|MGC138347|MGC142177|RXFPR1	4	Cytokine_Receptors
RXFP2	122042	relaxin/insulin-like family peptide receptor 2	GPR106|GREAT|INSL3R|LGR8|LGR8.1|RXFPR2	13	Cytokine_Receptors
RXFP3	51289	relaxin/insulin-like family peptide receptor 3	GPCR135|MGC141998|MGC142000|RLN3R1|RXFPR3|SALPR	5	Cytokine_Receptors
RXRA	6256	retinoid X receptor, alpha	FLJ00280|FLJ00318|FLJ16020|FLJ16733|MGC102720|NR2B1	9	Cytokine_Receptors
RXRB	6257	retinoid X receptor, beta	DAUDI6|H-2RIIBP|MGC1831|NR2B2|RCoR-1	6	Cytokine_Receptors
RXRG	6258	retinoid X receptor, gamma	NR2B3|RXRC	1	Cytokine_Receptors
S1PR1	1901	sphingosine-1-phosphate receptor 1	CHEDG1|D1S3362|ECGF1|EDG-1|EDG1|FLJ58121|S1P1	1	Cytokine_Receptors
S1PR2	9294	sphingosine-1-phosphate receptor 2	AGR16|EDG-5|EDG5|Gpcr13|H218|LPB2|S1P2	19	Cytokine_Receptors

SCTR	6344	secretin receptor	SR	2	Cytokine_Receptors
SDC1	6382	syndecan 1	CD138|SDC|SYND1|syndecan	2	Cytokine_Receptors
SDC2	6383	syndecan 2	HSPG|HSPG1|SYND2	8	Cytokine_Receptors
SDC3	9672	syndecan 3	N-syndecan|SDCN|SYND3	1	Cytokine_Receptors
SDC4	6385	syndecan 4	MGC22217|SYND4	20	Cytokine_Receptors
SORT1	6272	sortilin 1	Gp95|NT3	1	Cytokine_Receptors
SSTR1	6751	somatostatin receptor 1	SRIF-2	14	Cytokine_Receptors
SSTR2	6752	somatostatin receptor 2	-	17	Cytokine_Receptors
SSTR5	6755	somatostatin receptor 5	-	16	Cytokine_Receptors
ST2	6761	suppression of tumorigenicity 2	-	11	Cytokine_Receptors
TACR1	6869	tachykinin receptor 1	NK1R|NKIR|SPR|TAC1R	2	Cytokine_Receptors
TEK	7010	TEK tyrosine kinase, endothelial	CD202B|TIE-2|TIE2|VMCM|VMCM1	9	Cytokine_Receptors
TGFBR1	7046	transforming growth factor, beta receptor 1	AAT5|ACVRLK4|ALK-5|ALK5|LDS1A|LDS2A|SKR4|TGFR-1	9	Cytokine_Receptors
TGFBR2	7048	transforming growth factor, beta receptor II (70/80kDa)	AAT3|FAA3|LDS1B|LDS2B|MFS2|RIIC|TAAD2|TGFR-2|TGFbeta-RII	3	Cytokine_Receptors
TGFBR3	7049	transforming growth factor, beta receptor III	BGCAN|betaglycan	1	Cytokine_Receptors
THRA	7067	thyroid hormone receptor, alpha (erythroblastic leukemia viral (v-erb-a) oncogene homolog, avian)	AR7|EAR7|ERB-T-1|ERBA|ERBA1|MGC000261|MGC43240|NR1A1|THRA1|THRA2|c-ERBA-1	17	Cytokine_Receptors
THRB	7068	thyroid hormone receptor, beta (erythroblastic leukemia viral (v-erb-a) oncogene homolog 2, avian)	ERBA-BETA|ERBA2|GRTH|MGC126109|MGC126110|NR1A2|PRTH|THR1|THRB1|THRB2	3	Cytokine_Receptors

TIE1	7075	tyrosine kinase with immunoglobulin-like and EGF-like domains 1	JTK14|TIE	1	Cytokine_Receptors
TNFRSF10A	8797	tumor necrosis factor receptor superfamily, member 10a	APO2|CD261|DR4|MGC9365|TRAILR-1|TRAILR1	8	Cytokine_Receptors
TNFRSF10B	8795	tumor necrosis factor receptor superfamily, member 10b	CD262|DR5|KILLER|KILLER/DR5|TRAIL-R2|TRAILR2|TRICK2|TRICK2A|TRICK2B|TRICKB|ZTNFR9	8	Cytokine_Receptors
TNFRSF10C	8794	tumor necrosis factor receptor superfamily, member 10c, decoy without an intracellular domain	CD263|DCR1|LIT|MGC149501|MGC149502|TRAILR3|TRID	8	Cytokine_Receptors
TNFRSF10D	8793	tumor necrosis factor receptor superfamily, member 10d, decoy with truncated death domain	CD264|DCR2|TRAILR4|TRUNDD	8	Cytokine_Receptors
TNFRSF11A	8792	tumor necrosis factor receptor superfamily, member 11a, NFKB activator	CD265|FEO|LOH18CR1|ODFR|OFE|OPTB7|OSTS|PDB2|RANK|TRANCER	18	Cytokine_Receptors
TNFRSF12A	51330	tumor necrosis factor receptor superfamily, member 12A	CD266|FN14|TWEAKR	16	Cytokine_Receptors
TNFRSF13B	23495	tumor necrosis factor receptor superfamily, member 13B	CD267|CVID|FLJ39942|MGC133214|MGC39952|TACI|TNFRSF14B	17	Cytokine_Receptors
TNFRSF13C	115650	tumor necrosis factor receptor superfamily, member 13C	BAFF-R|BAFFR|CD268|MGC138235	22	Cytokine_Receptors
TNFRSF14	8764	tumor necrosis factor receptor superfamily, member 14 (herpesvirus entry mediator)	ATAR|HVEA|HVEM|LIGHTR|TR2	1	Cytokine_Receptors

TNFRSF17	608	tumor necrosis factor receptor superfamily, member 17	BCM|BCMA|CD269	16	Cytokine_Receptors
TNFRSF18	8784	tumor necrosis factor receptor superfamily, member 18	AITR|GITR|GITR-D	1	Cytokine_Receptors
TNFRSF19	55504	tumor necrosis factor receptor superfamily, member 19	TAJ|TAJ-alpha|TRADE|TROY	13	Cytokine_Receptors
TNFRSF1A	7132	tumor necrosis factor receptor superfamily, member 1A	CD120a|FPF|MGC19588|TBP1|TNF-R|TNF-R-I|TNF-R55|TNFAR|TNFR1|TNFR55|TNFR60|p55|p55-R|p60	12	Cytokine_Receptors
TNFRSF1B	7133	tumor necrosis factor receptor superfamily, member 1B	CD120b|TBPII|TNF-R-II|TNF-R75|TNFBR|TNFR1B|TNFR2|TNFR80|p75|p75TNFR	1	Cytokine_Receptors
TNFRSF21	27242	tumor necrosis factor receptor superfamily, member 21	BM-018|DR6|MGC31965	6	Cytokine_Receptors
TNFRSF25	8718	tumor necrosis factor receptor superfamily, member 25	APO-3|DDR3|DR3|LARD|TNFRSF12|TR3|TRAMP|WSL-1|WSL-LR	1	Cytokine_Receptors
TNFRSF4	7293	tumor necrosis factor receptor superfamily, member 4	ACT35|CD134|OX40|TXGP1L	1	Cytokine_Receptors
TNFRSF6B	8771	tumor necrosis factor receptor superfamily, member 6b, decoy	DCR3|DJ583P15.1.1|M68|TR6	20	Cytokine_Receptors
TNFRSF8	943	tumor necrosis factor receptor superfamily, member 8	CD30|D1S166E|Ki-1	1	Cytokine_Receptors
TNFRSF9	3604	tumor necrosis factor receptor superfamily, member 9	4-1BB|CD137|CDw137|ILA|MGC2172	1	Cytokine_Receptors

TRHR	7201	thyrotropin-releasing hormone receptor	MGC141920	8	Cytokine_Receptors
TSHR	7253	thyroid stimulating hormone receptor	CHNG1|LGR3|MGC75129|hTSHR-I	14	Cytokine_Receptors
TUBB3	10381	tubulin, beta 3	MC1R|TUBB4|beta-4	16	Cytokine_Receptors
VDR	7421	vitamin D (1,25-dihydroxyvitamin D3) receptor	NR1I1	12	Cytokine_Receptors
VIPR1	7433	vasoactive intestinal peptide receptor 1	FLJ41949|HVR1|II|PACAP-R-2|RDC1|VAPC1|VIPR|VIRG|VPAC1|VPCAP1R	3	Cytokine_Receptors
VIPR2	7434	vasoactive intestinal peptide receptor 2	FLJ16511|VPAC2|VPCAP2R	7	Cytokine_Receptors
XCR1	2829	chemokine (C motif) receptor 1	CCXCR1|GPR5	3	Cytokine_Receptors
IFNA10	3446	interferon, alpha 10	MGC119878|MGC119879	9	Interferons
IFNA13	3447	interferon, alpha 13	-	9	Interferons
IFNA14	3448	interferon, alpha 14	LEIF2H|MGC125756|MGC125757	9	Interferons
IFNA16	3449	interferon, alpha 16	-	9	Interferons
IFNA17	3451	interferon, alpha 17	IFNA|INFA|LEIF2C1	9	Interferons
IFNA2	3440	interferon, alpha 2	IFNA|INFA2|MGC125764|MGC125765	9	Interferons
IFNA21	3452	interferon, alpha 21	MGC126687|MGC126689	9	Interferons
IFNA4	3441	interferon, alpha 4	INFA4|MGC142200	9	Interferons
IFNA5	3442	interferon, alpha 5	INFA5	9	Interferons
IFNA6	3443	interferon, alpha 6	-	9	Interferons
IFNA7	3444	interferon, alpha 7	IFNA-J	9	Interferons
IFNA8	3445	interferon, alpha 8	-	9	Interferons
IFNB1	3456	interferon, beta 1, fibroblast	IFB|IFF|IFNB|MGC96956	9	Interferons
IFNE	338376	interferon, epsilon	IFN-	9	Interferons

			E|IFNE1|IFNT1|MGC119018|MGC119020|PRO 655		
IFNG	3458	interferon, gamma	IFG|IFI	12	Interferons
IFNK	56832	interferon, kappa	RP11-27J8.1	9	Interferons
IFNW1	3467	interferon, omega 1	-	9	Interferons
IFNAR2	3455	interferon (alpha, beta and omega) receptor 2	IFN-R|IFN-alpha-REC|IFNABR|IFNARB	21	Interferon_Receptor
IFNGR1	3459	interferon gamma receptor 1	CD119|FLJ45734|IFNGR	6	Interferon_Receptor
IFNGR2	3460	interferon gamma receptor 2 (interferon gamma transducer 1)	AF-1|IFGR2|IFNGT1	21	Interferon_Receptor
IL11	3589	interleukin 11	AGIF|IL-11	19	Interleukins
IL12A	3592	interleukin 12A (natural killer cell stimulatory factor 1, cytotoxic lymphocyte maturation factor 1, p35)	CLMF|IL-12A|NFSK|NKSF1|P35	3	Interleukins
IL12B	3593	interleukin 12B (natural killer cell stimulatory factor 2, cytotoxic lymphocyte maturation factor 2, p40)	CLMF|CLMF2|IL-12B|NKSF|NKSF2	5	Interleukins
IL13	3596	interleukin 13	ALRH|BHR1|IL-13|MGC116786|MGC116788|MGC116789|P600	5	Interleukins
IL15	3600	interleukin 15	IL-15|MGC9721	4	Interleukins
IL16	3603	interleukin 16 (lymphocyte chemoattractant factor)	FLJ16806|FLJ42735|FLJ44234|HsT19289|IL-16|LCF|prIL-16	15	Interleukins
IL17A	3605	interleukin 17A	CTLA8|IL-17|IL-17A|IL17	6	Interleukins
IL17B	27190	interleukin 17B	IL-17B|IL-20|MGC138900|MGC138901|ZCYTO7	5	Interleukins

IL17C	27189	interleukin 17C	CX2|IL-17C|IL-21|MGC126884|MGC138401	16	Interleukins
IL17D	53342	interleukin 17D	FLJ30846|IL-17D|IL-22|IL-27|IL27	13	Interleukins
IL17F	112744	interleukin 17F	IL-17F|ML-1|ML1	6	Interleukins
IL18	3606	interleukin 18 (interferon-gamma-inducing factor)	IGIF|IL-18|IL-1g|IL1F4|MGC12320	11	Interleukins
IL19	29949	interleukin 19	IL-10C|MDA1|NG.1|ZMDA1	1	Interleukins
IL1A	3552	interleukin 1, alpha	IL-1A|IL1|IL1-ALPHA|IL1F1	2	Interleukins
IL1B	3553	interleukin 1, beta	IL-1|IL1-BETA|IL1F2	2	Interleukins
IL1F10	84639	interleukin 1 family, member 10 (theta)	FIL1-theta|FKSG75|IL-1HY2|IL1-theta|MGC119831|MGC119832|MGC119833	2	Interleukins
IL1F5	26525	interleukin 1 family, member 5 (delta)	FIL1|FIL1(DELTA)|FIL1D|IL1HY1|IL1L1|IL1RP3|MGC29840	2	Interleukins
IL1F6	27179	interleukin 1 family, member 6 (epsilon)	FIL1|FIL1(EPSILON)|FIL1E|IL-1F6|IL1(EPSILON)|MGC129552|MGC129553	2	Interleukins
IL1F7	27178	interleukin 1 family, member 7 (zeta)	FIL1|FIL1(ZETA)|FIL1Z|IL-1F7|IL-1H4|IL-1RP1|IL1H4|IL1RP1	2	Interleukins
IL1F8	27177	interleukin 1 family, member 8 (eta)	FIL1|FIL1-(ETA)|FIL1H|IL-1F8|IL-1H2|IL1-ETA|IL1H2|MGC126880|MGC126882	2	Interleukins
IL1F9	56300	interleukin 1 family, member 9	IL-1F9|IL-1H1|IL-1RP2|IL1E|IL1H1|IL1RP2	2	Interleukins
IL1RN	3557	interleukin 1 receptor antagonist	ICIL-1RA|IL-1ra3|IL1F3|IL1RA|IRAP|MGC10430	2	Interleukins
IL2	3558	interleukin 2	IL-2|TCGF|lymphokine	4	Interleukins
IL20	50604	interleukin 20	IL-20|IL10D|MGC96907|ZCYTO10	1	Interleukins
IL21	59067	interleukin 21	IL-21|Za11	4	Interleukins
IL22	50616	interleukin 22	IL-21|IL-22|IL-D110|IL-TIF|IL21|ILTIF|MGC79382|MGC79384|TIFIL-23|TIFa|zcyto18	12	Interleukins

IL23A	51561	interleukin 23, alpha subunit p19	IL-23|IL-23A|IL23P19|MGC79388|P19|SGRF	12	Interleukins
IL24	11009	interleukin 24	C49A|FISP|IL-24|IL10B|MDA7|Mob-5|ST16|mda-7	1	Interleukins
IL25	64806	interleukin 25	IL-17E|IL-25|IL17E	14	Interleukins
IL26	55801	interleukin 26	AK155|IL-26	12	Interleukins
IL27	246778	interleukin 27	IL-27|IL-27A|IL27p28|IL30|MGC71873|p28	16	Interleukins
IL28A	282616	interleukin 28A (interferon, lambda 2)	IFNL2|IL-28A	19	Interleukins
IL28B	282617	interleukin 28B (interferon, lambda 3)	IFNL3|IL-28B|IL28C	19	Interleukins
IL29	282618	interleukin 29 (interferon, lambda 1)	IFNL1|IL-29	19	Interleukins
IL3	3562	interleukin 3 (colony-stimulating factor, multiple)	IL-3|MCGF|MGC79398|MGC79399|MULTI-CSF	5	Interleukins
IL31	386653	interleukin 31	IL-31	12	Interleukins
IL32	9235	interleukin 32	IL-32alpha|IL-32beta|IL-32delta|IL-32gamma|NK4|TAIF|TAIFa|TAIFb|TAIFc|TAIFd	16	Interleukins
IL33	90865	interleukin 33	C9orf26|DKFZp586H0523|DVS27|NF-HEV|NFEHEV|RP11-575C20.2	9	Interleukins
IL34	146433	interleukin 34	C16orf77|IL-34|MGC34647	16	Interleukins
IL4	3565	interleukin 4	BCGF-1|BCGF1|BSF1|IL-4|MGC79402	5	Interleukins
IL5	3567	interleukin 5 (colony-stimulating factor, eosinophil)	EDF|IL-5|TRF	5	Interleukins
IL6	3569	interleukin 6 (interferon, beta 2)	BSF2|HGF|HSF|IFNB2|IL-6	7	Interleukins
IL6ST	3572	interleukin 6 signal transducer (gp130, oncostatin M receptor)	CD130|CDw130|GP130|GP130-RAPS|IL6R-beta	5	Interleukins

IL7	3574	interleukin 7	IL-7	8	Interleukins
IL8	3576	interleukin 8	CXCL8|GCP-1|GCP1|LECT|LUCT|LYNAP|MDNCF|MONAP|NAF|NAP-1|NAP1	4	Interleukins
IL9	3578	interleukin 9	HP40|IL-9|P40	5	Interleukins
TXLNA	200081	taxilin alpha	DKFZp451J0118|IL14|MGC118870|MGC118871|RP4-622L5.4|TXLN	1	Interleukins
IL10RA	3587	interleukin 10 receptor, alpha	CDW210A|HIL-10R|IL-10R1|IL10R	11	Interleukins_Receptor
IL10RB	3588	interleukin 10 receptor, beta	CDW210B|CRF2-4|CRFB4|D21S58|D21S66|IL-10R2	21	Interleukins_Receptor
IL11RA	3590	interleukin 11 receptor, alpha	MGC2146	9	Interleukins_Receptor
IL11RB	3591	interleukin 11 receptor, beta	-	-	Interleukins_Receptor
IL12RB1	3594	interleukin 12 receptor, beta 1	CD212|IL-12R-BETA1|IL12RB|MGC34454	19	Interleukins_Receptor
IL12RB2	3595	interleukin 12 receptor, beta 2	-	1	Interleukins_Receptor
IL13RA1	3597	interleukin 13 receptor, alpha 1	CD213A1|IL-13Ra|NR4	X	Interleukins_Receptor
IL13RA2	3598	interleukin 13 receptor, alpha 2	CD213A2|CT19|IL-13R|IL13BP	X	Interleukins_Receptor
IL15RA	3601	interleukin 15 receptor, alpha	MGC104179	10	Interleukins_Receptor
IL15RB	3602	interleukin 15 receptor, beta	-	-	Interleukins_Receptor
IL17RA	23765	interleukin 17 receptor A	CD217|CDw217|IL-17RA|IL17R|MGC10262|hIL-17R	22	Interleukins_Receptor
IL17RB	55540	interleukin 17 receptor B	CRL4|EVI27|IL17BR|IL17RH1|MGC5245	3	Interleukins_Receptor
IL17RC	84818	interleukin 17 receptor C	FLJ95963|FLJ96005|IL17-RL|IL17RL|MGC10763	3	Interleukins_Receptor
IL17RD	54756	interleukin 17 receptor D	DKFZp434N1928|FLJ35755|IL-17RD|IL17RLM|MGC133309|SEF	3	Interleukins_Receptor
IL17RE	132014	interleukin 17 receptor E	FLJ23658|MGC71884	3	Interleukins_Receptor

IL18R1	8809	interleukin 18 receptor 1	CD218a|CDw218a|IL-1Rrp|IL18RA|IL1RRP	2	Interleukins_Receptor
IL18RAP	8807	interleukin 18 receptor accessory protein	ACPL|CD218b|CDw218b|IL18RB|MGC120589|MGC120590	2	Interleukins_Receptor
IL1R1	3554	interleukin 1 receptor, type I	CD121A|D2S1473|IL-1R-alpha|IL1R|IL1RA|P80	2	Interleukins_Receptor
IL1R2	7850	interleukin 1 receptor, type II	CD121b|IL1RB|MGC47725	2	Interleukins_Receptor
IL1RAP	3556	interleukin 1 receptor accessory protein	C3orf13|FLJ37788|IL-1RAcP|IL1R3	3	Interleukins_Receptor
IL1RL1	9173	interleukin 1 receptor-like 1	DER4|FIT-1|MGC32623|ST2|ST2L|ST2V|T1	2	Interleukins_Receptor
IL1RL2	8808	interleukin 1 receptor-like 2	IL1R-rp2|IL1RRP2	2	Interleukins_Receptor
IL20RA	53832	interleukin 20 receptor, alpha	FLJ40993|IL-20R1|ZCYTOR7	6	Interleukins_Receptor
IL20RB	53833	interleukin 20 receptor beta	DIRS1|FNDC6|IL-20R2|MGC34923	3	Interleukins_Receptor
IL21R	50615	interleukin 21 receptor	MGC10967|NILR	16	Interleukins_Receptor
IL22RA1	58985	interleukin 22 receptor, alpha 1	CRF2-9|IL22R|IL22R1	1	Interleukins_Receptor
IL22RA2	116379	interleukin 22 receptor, alpha 2	CRF2-10|CRF2-S1|CRF2X|IL-22BP|MGC150509|MGC150510	6	Interleukins_Receptor
IL23R	149233	interleukin 23 receptor	-	1	Interleukins_Receptor
IL27RA	9466	interleukin 27 receptor, alpha	CRL1|IL27R|TCCR|WSX1|zcytor1	19	Interleukins_Receptor
IL28RA	163702	interleukin 28 receptor, alpha (interferon, lambda receptor)	CRF2/12|IFNLR|IFNLR1|IL-28R1|LICR2	1	Interleukins_Receptor
IL2RA	3559	interleukin 2 receptor, alpha	CD25|IDDM10|IL2R|TCGFR	10	Interleukins_Receptor
IL2RB	3560	interleukin 2 receptor, beta	CD122|P70-75	22	Interleukins_Receptor
IL2RG	3561	interleukin 2 receptor, gamma (severe combined immunodeficiency)	CD132|IMD4|SCIDX|SCIDX1	X	Interleukins_Receptor
IL31RA	133396	interleukin 31 receptor A	CRL|CRL3|GLM-R|GLMR|GPL|IL-31RA|MGC125346|PRO21384	5	Interleukins_Receptor

IL3RA	3563	interleukin 3 receptor, alpha (low affinity)	CD123|IL3R|IL3RAY|IL3RX|IL3RY|MGC34174|hIL-3Ra	X|Y	Interleukins_Receptor
IL4R	3566	interleukin 4 receptor	CD124|IL4RA	16	Interleukins_Receptor
IL5RA	3568	interleukin 5 receptor, alpha	CD125|CDw125|HSIL5R3|IL5R|MGC26560	3	Interleukins_Receptor
IL6R	3570	interleukin 6 receptor	CD126|IL-6R-1|IL-6R-alpha|IL6RA|MGC104991	1	Interleukins_Receptor
IL7R	3575	interleukin 7 receptor	CD127|CDW127|IL-7R-alpha|IL7RA|ILRA	5	Interleukins_Receptor
IL8RA	3577	interleukin 8 receptor, alpha	C-C|C-C-CKR-1|CD128|CD181|CDw128a|CKR-1|CMKAR1|CXCR1|IL8R1|IL8RBA	2	Interleukins_Receptor
IL8RB	3579	interleukin 8 receptor, beta	CD182|CDw128b|CMKAR2|CXCR2|IL8R2|IL8RA	2	Interleukins_Receptor
IL9R	3581	interleukin 9 receptor	CD129	X|Y	Interleukins_Receptor
ST2	6761	suppression of tumorigenicity 2	-	11	Interleukins_Receptor
HLA-A	3105	major histocompatibility complex, class I, A	FLJ26655|HLAA	6	NaturalKiller_Cell_Cytotoxicity
HLA-B	3106	major histocompatibility complex, class I, B	AS|HLA-B-7301|HLA-B73|HLAB|HLAC|MGC111087|SPDA1	6	NaturalKiller_Cell_Cytotoxicity
HLA-C	3107	major histocompatibility complex, class I, C	D6S204|FLJ27082|HLA-Cw|HLA-Cw12|HLA-JY3|HLC-C|PSORS1	6	NaturalKiller_Cell_Cytotoxicity
HLA-E	3133	major histocompatibility complex, class I, E	DKFZp686P19218|EA1.2|EA2.1|HLA-6.2|MHC|QA1	6	NaturalKiller_Cell_Cytotoxicity
HLA-G	3135	major histocompatibility complex, class I, G	MHC-G	6	NaturalKiller_Cell_Cytotoxicity
KIR3DL1	3811	killer cell immunoglobulin-like receptor, three domains, long cytoplasmic tail, 1	CD158E1|KIR|MGC119726|MGC119728|MGC126589|MGC126591|NKAT3|NKB1|NKB1B	19	NaturalKiller_Cell_Cytotoxicity

KIR3DL2	3812	killer cell immunoglobulin-like receptor, three domains, long cytoplasmic tail, 2	CD158K|MGC125321|NKAT4|NKAT4B|p140	19	NaturalKiller_Cell_Cytotoxicity
KIR2DL1	3802	killer cell immunoglobulin-like receptor, two domains, long cytoplasmic tail, 1	CD158A|KIR-K64|KIR221|NKAT|NKAT1|p58.1	19	NaturalKiller_Cell_Cytotoxicity
KIR2DL2	3803	killer cell immunoglobulin-like receptor, two domains, long cytoplasmic tail, 2	CD158B1|CD158b|NKAT6|p58.2	19	NaturalKiller_Cell_Cytotoxicity
KIR2DL3	3804	killer cell immunoglobulin-like receptor, two domains, long cytoplasmic tail, 3	CD158B2|CD158b|GL183|KIR-023GB|KIR-K7b|KIR-K7c|KIRCL23|MGC129943|NKAT|NKAT2|NKAT2A|NKAT2B|p58	19	NaturalKiller_Cell_Cytotoxicity
KIR2DL4	3805	killer cell immunoglobulin-like receptor, two domains, long cytoplasmic tail, 4	CD158D|G9P|KIR103|KIR103AS	19	NaturalKiller_Cell_Cytotoxicity
KIR2DL5A	57292	killer cell immunoglobulin-like receptor, two domains, long cytoplasmic tail, 5A	CD158F|KIR2DL5|KIR2DL5.1|KIR2DL5.3	19	NaturalKiller_Cell_Cytotoxicity
KLRC1	3821	killer cell lectin-like receptor subfamily C, member 1	CD159A|MGC13374|MGC59791|NKG2|NKG2A	12	NaturalKiller_Cell_Cytotoxicity
KLRC2	3822	killer cell lectin-like receptor subfamily C, member 2	CD159c|MGC138244|NKG2-C|NKG2C	12	NaturalKiller_Cell_Cytotoxicity
KLRC3	3823	killer cell lectin-like receptor subfamily C, member 3	NKG2-E|NKG2E	12	NaturalKiller_Cell_Cytotoxicity

KLRD1	3824	killer cell lectin-like receptor subfamily D, member 1	CD94	12	NaturalKiller_Cell_Cytotoxicity
PTPN6	5777	protein tyrosine phosphatase, non-receptor type 6	HCP|HCPH|HPTP1C|PTP-1C|SH-PTP1|SHP-1|SHP-1L|SHP1	12	NaturalKiller_Cell_Cytotoxicity
PTPN11	5781	protein tyrosine phosphatase, non-receptor type 11	BPTP3|CFC|MGC14433|NS1|PTP-1D|PTP2C|SH-PTP2|SH-PTP3|SHP2	12	NaturalKiller_Cell_Cytotoxicity
ICAM1	3383	intercellular adhesion molecule 1	BB2|CD54|P3.58	19	NaturalKiller_Cell_Cytotoxicity
ICAM2	3384	intercellular adhesion molecule 2	CD102	17	NaturalKiller_Cell_Cytotoxicity
ITGAL	3683	integrin, alpha L (antigen CD11A (p180), lymphocyte function-associated antigen 1; alpha polypeptide)	CD11A|LFA-1|LFA1A	16	NaturalKiller_Cell_Cytotoxicity
ITGB2	3689	integrin, beta 2 (complement component 3 receptor 3 and 4 subunit)	CD18|LAD|LCAMB|LFA-1|MAC-1|MF17|MFI7	21	NaturalKiller_Cell_Cytotoxicity
PTK2B	2185	PTK2B protein tyrosine kinase 2 beta	CADTK|CAKB|FADK2|FAK2|FRNK|PKB|PTK|PYK2|RAFTK	8	NaturalKiller_Cell_Cytotoxicity
VAV3	10451	vav 3 guanine nucleotide exchange factor	FLJ40431	1	NaturalKiller_Cell_Cytotoxicity
VAV1	7409	vav 1 guanine nucleotide exchange factor	VAV	19	NaturalKiller_Cell_Cytotoxicity
VAV2	7410	vav 2 guanine nucleotide exchange factor	-	9	NaturalKiller_Cell_Cytotoxicity

RAC1	5879	ras-related C3 botulinum toxin substrate 1 (rho family, small GTP binding protein Rac1)	MGC111543|MIG5|TC-25|p21-Rac1	7	NaturalKiller_Cell_Cytotoxicity
RAC2	5880	ras-related C3 botulinum toxin substrate 2 (rho family, small GTP binding protein Rac2)	EN-7|Gx|HSPC022	22	NaturalKiller_Cell_Cytotoxicity
RAC3	5881	ras-related C3 botulinum toxin substrate 3 (rho family, small GTP binding protein Rac3)	-	17	NaturalKiller_Cell_Cytotoxicity
PAK1	5058	p21 protein (Cdc42/Rac)-activated kinase 1	MGC130000|MGC130001|PAKalpha	11	NaturalKiller_Cell_Cytotoxicity
MAP2K1	5604	mitogen-activated protein kinase kinase 1	MAPKK1|MEK1|MKK1|PRKMK1	15	NaturalKiller_Cell_Cytotoxicity
MAP2K2	5605	mitogen-activated protein kinase kinase 2	FLJ26075|MAPKK2|MEK2|MKK2|PRKMK2	19	NaturalKiller_Cell_Cytotoxicity
MAPK1	5594	mitogen-activated protein kinase 1	ERK|ERK2|ERT1|MAPK2|P42MAPK|PRKM1|PRKM2|p38|p40|p41|p41mapk	22	NaturalKiller_Cell_Cytotoxicity
MAPK3	5595	mitogen-activated protein kinase 3	ERK1|HS44KDAP|HUMKER1A|MGC20180|P44ERK1|P44MAPK|PRKM3	16	NaturalKiller_Cell_Cytotoxicity
TNF	7124	tumor necrosis factor (TNF superfamily, member 2)	DIF|TNF-alpha|TNFA|TNFSF2	6	NaturalKiller_Cell_Cytotoxicity
CSF2	1437	colony stimulating factor 2 (granulocyte-macrophage)	GMCSF|MGC131935|MGC138897	5	NaturalKiller_Cell_Cytotoxicity
IFNG	3458	interferon, gamma	IFG|IFI	12	NaturalKiller_Cell_Cytotoxicity

KIR2DS1	3806	killer cell immunoglobulin-like receptor, two domains, short cytoplasmic tail, 1	CD158H|CD158a|p50.1	19	NaturalKiller_Cell_Cytotoxicity
KIR2DS3	3808	killer cell immunoglobulin-like receptor, two domains, short cytoplasmic tail, 3	NKAT7	19	NaturalKiller_Cell_Cytotoxicity
KIR2DS4	3809	killer cell immunoglobulin-like receptor, two domains, short cytoplasmic tail, 4	CD158I|KIR1D|KIR412|KKA3|MGC120019|MGC125315|MGC125317|NKAT8	19	NaturalKiller_Cell_Cytotoxicity
KIR2DS5	3810	killer cell immunoglobulin-like receptor, two domains, short cytoplasmic tail, 5	CD158G|NKAT9	19	NaturalKiller_Cell_Cytotoxicity
NCR2	9436	natural cytotoxicity triggering receptor 2	CD336|LY95|NK-p44|NKP44|dJ149M18.1	6	NaturalKiller_Cell_Cytotoxicity
TYROBP	7305	TYRO protein tyrosine kinase binding protein	DAP12|KARAP|PLOSL	19	NaturalKiller_Cell_Cytotoxicity
LCK	3932	lymphocyte-specific protein tyrosine kinase	YT16|p56lck|pp58lck	1	NaturalKiller_Cell_Cytotoxicity
FCGR3A	2214	Fc fragment of IgG, low affinity IIIa, receptor (CD16a)	CD16|CD16A|FCG3|FCGR3|FCGRIII|FCR-10|FCRIII|FCRIIIA|IGFR3	1	NaturalKiller_Cell_Cytotoxicity
FCGR3B	2215	Fc fragment of IgG, low affinity IIIb, receptor (CD16b)	CD16|CD16b|FCG3|FCGR3	1	NaturalKiller_Cell_Cytotoxicity
LOC652578	652578	similar to Fc fragment of IgG, low affinity IIIa, receptor for (CD16)	-	Un	NaturalKiller_Cell_Cytotoxicity

NCR1	9437	natural cytotoxicity triggering receptor 1	CD335|FLJ99094|LY94|NK-p46|NKP46	19	NaturalKiller_Cell_Cytotoxicity
NCR3	259197	natural cytotoxicity triggering receptor 3	1C7|CD337|LY117|MALS|NKp30	6	NaturalKiller_Cell_Cytotoxicity
FCER1G	2207	Fc fragment of IgE, high affinity I, receptor for; gamma polypeptide	FCRG	1	NaturalKiller_Cell_Cytotoxicity
CD247	919	CD247 molecule	CD3-ZETA|CD3H|CD3Q|CD3Z|T3Z|TCRZ	1	NaturalKiller_Cell_Cytotoxicity
ZAP70	7535	zeta-chain (TCR) associated protein kinase 70kDa	FLJ17670|FLJ17679|SRK|STD|TZK|ZAP-70	2	NaturalKiller_Cell_Cytotoxicity
SYK	6850	spleen tyrosine kinase	DKFZp313N1010|FLJ25043|FLJ37489	9	NaturalKiller_Cell_Cytotoxicity
LCP2	3937	lymphocyte cytosolic protein 2 (SH2 domain containing leukocyte protein of 76kDa)	SLP-76|SLP76	5	NaturalKiller_Cell_Cytotoxicity
LAT	27040	linker for activation of T cells	LAT1|pp36	16	NaturalKiller_Cell_Cytotoxicity
PLCG1	5335	phospholipase C, gamma 1	PLC-II|PLC1|PLC148|PLCgamma1	20	NaturalKiller_Cell_Cytotoxicity
PLCG2	5336	phospholipase C, gamma 2 (phosphatidylinositol-specific)	-	16	NaturalKiller_Cell_Cytotoxicity
SH3BP2	6452	SH3-domain binding protein 2	3BP2|CRBM|CRPM|FLJ42079|FLJ54978|RES4-23	4	NaturalKiller_Cell_Cytotoxicity
PIK3CA	5290	phosphoinositide-3-kinase, catalytic, alpha polypeptide	MGC142161|MGC142163|PI3K|p110-alpha	3	NaturalKiller_Cell_Cytotoxicity
PIK3CB	5291	phosphoinositide-3-kinase, catalytic, beta polypeptide	DKFZp779K1237|MGC133043|PI3K|PI3KCB|PI3Kbeta|PIK3C1|p110-BETA	3	NaturalKiller_Cell_Cytotoxicity
PIK3CD	5293	phosphoinositide-3-kinase, catalytic, delta polypeptide	p110D	1	NaturalKiller_Cell_Cytotoxicity

PIK3CG	5294	phosphoinositide-3-kinase, catalytic, gamma polypeptide	PI3CG|PI3K|PI3Kgamma|PIK3	7	NaturalKiller_Cell_Cytotoxicity
PIK3R5	23533	phosphoinositide-3-kinase, regulatory subunit 5	F730038I15Rik|FOAP-2|P101-PI3K|p101	17	NaturalKiller_Cell_Cytotoxicity
PIK3R1	5295	phosphoinositide-3-kinase, regulatory subunit 1 (alpha)	GRB1|p85|p85-ALPHA	5	NaturalKiller_Cell_Cytotoxicity
PIK3R2	5296	phosphoinositide-3-kinase, regulatory subunit 2 (beta)	P85B|p85|p85-BETA	19	NaturalKiller_Cell_Cytotoxicity
PIK3R3	8503	phosphoinositide-3-kinase, regulatory subunit 3 (gamma)	DKFZp686P05226|FLJ41892|p55|p55-GAMMA	1	NaturalKiller_Cell_Cytotoxicity
FYN	2534	FYN oncogene related to SRC, FGR, YES	MGC45350|SLK|SYN	6	NaturalKiller_Cell_Cytotoxicity
SHC2	25759	SHC (Src homology 2 domain containing) transforming protein 2	SCK|SHCB|SLI	19	NaturalKiller_Cell_Cytotoxicity
SHC4	399694	SHC (Src homology 2 domain containing) family, member 4	MGC34023|RaLP|SHCD	15	NaturalKiller_Cell_Cytotoxicity
SHC3	53358	SHC (Src homology 2 domain containing) transforming protein 3	N-Shc|NSHC|RAI|SHCC	9	NaturalKiller_Cell_Cytotoxicity
SHC1	6464	SHC (Src homology 2 domain containing) transforming protein 1	FLJ26504|SHC|SHCA	1	NaturalKiller_Cell_Cytotoxicity
GRB2	2885	growth factor receptor-bound protein 2	ASH|EGFRBP-GRB2|Grb3-3|MST084|MSTP084	17	NaturalKiller_Cell_Cytotoxicity

SOS1	6654	son of sevenless homolog 1 (Drosophila)	GF1|GGF1|GINGF|HGF|NS4	2	NaturalKiller_Cell_Cytotoxicity
SOS2	6655	son of sevenless homolog 2 (Drosophila)	FLJ25596	14	NaturalKiller_Cell_Cytotoxicity
HRAS	3265	v-Ha-ras Harvey rat sarcoma viral oncogene homolog	C-BAS/HAS|C-H-RAS|C-HA-RAS1|CTLO|H-RASIDX|HAMSV|HRAS1|K-RAS|N-RAS|RASH1	11	NaturalKiller_Cell_Cytotoxicity
KRAS	3845	v-Ki-ras2 Kirsten rat sarcoma viral oncogene homolog	C-K-RAS|K-RAS2A|K-RAS2B|K-RAS4A|K-RAS4B|KI-RAS|KRAS1|KRAS2|NS3|RASK2	12	NaturalKiller_Cell_Cytotoxicity
NRAS	4893	neuroblastoma RAS viral (v-ras) oncogene homolog	ALPS4|N-ras|NRAS1	1	NaturalKiller_Cell_Cytotoxicity
ARAF	369	v-raf murine sarcoma 3611 viral oncogene homolog	A-RAF|ARAF1|PKS2|RAFA1	X	NaturalKiller_Cell_Cytotoxicity
BRAF	673	v-raf murine sarcoma viral oncogene homolog B1	B-RAF1|BRAF1|FLJ95109|MGC126806|MGC138284|RAFB1	7	NaturalKiller_Cell_Cytotoxicity
RAF1	5894	v-raf-1 murine leukemia viral oncogene homolog 1	CRAF|NS5|Raf-1|c-Raf	3	NaturalKiller_Cell_Cytotoxicity
MICA	4276	MHC class I polypeptide-related sequence A	FLJ60820|MGC111087|PERB11.1	6	NaturalKiller_Cell_Cytotoxicity
MICB	4277	MHC class I polypeptide-related sequence B	PERB11.2	6	NaturalKiller_Cell_Cytotoxicity
ULBP3	79465	UL16 binding protein 3	RAET1N	6	NaturalKiller_Cell_Cytotoxicity
ULBP2	80328	UL16 binding protein 2	N2DL2|RAET1H	6	NaturalKiller_Cell_Cytotoxicity
ULBP1	80329	UL16 binding protein 1	RAET1I	6	NaturalKiller_Cell_Cytotoxicity
KLRK1	22914	killer cell lectin-like receptor subfamily K, member 1	CD314|D12S2489E|FLJ17759|FLJ75772|KLR|NKG2-D|NKG2D	12	NaturalKiller_Cell_Cytotoxicity

HCST	10870	hematopoietic cell signal transducer	DAP10|DKFZP586C1522|KAP10|PIK3AP	19	NaturalKiller_Cell_Cytotoxicity
CD48	962	CD48 molecule	BCM1|BLAST|BLAST1|MEM-102|SLAMF2|hCD48|mCD48	1	NaturalKiller_Cell_Cytotoxicity
CD244	51744	CD244 molecule, natural killer cell receptor 2B4	2B4|NAIL|NKR2B4|Nmrk|SLAMF4	1	NaturalKiller_Cell_Cytotoxicity
PPP3CA	5530	protein phosphatase 3 (formerly 2B), catalytic subunit, alpha isoform	CALN|CALNA|CALNA1|CCN1|CNA1|PPP2B	4	NaturalKiller_Cell_Cytotoxicity
PPP3CB	5532	protein phosphatase 3 (formerly 2B), catalytic subunit, beta isoform	CALNA2|CALNB	10	NaturalKiller_Cell_Cytotoxicity
PPP3CC	5533	protein phosphatase 3 (formerly 2B), catalytic subunit, gamma isoform	CALNA3	8	NaturalKiller_Cell_Cytotoxicity
CHP	11261	calcium binding protein P22	SLC9A1BP	15	NaturalKiller_Cell_Cytotoxicity
PPP3R1	5534	protein phosphatase 3 (formerly 2B), regulatory subunit B, alpha isoform	CALNB1|CNB|CNB1	2	NaturalKiller_Cell_Cytotoxicity
PPP3R2	5535	protein phosphatase 3 (formerly 2B), regulatory subunit B, beta isoform	PPP3RL	9	NaturalKiller_Cell_Cytotoxicity
CHP2	63928	calcineurin B homologous protein 2	-	16	NaturalKiller_Cell_Cytotoxicity
NFAT5	10725	nuclear factor of activated T-cells 5, tonicity-responsive	KIAA0827|NF-AT5|NFATL1|NFATZ|OREBP|TONEBP	16	NaturalKiller_Cell_Cytotoxicity

NFATC1	4772	nuclear factor of activated T-cells, cytoplasmic, calcineurin-dependent 1	MGC138448|NF-ATC|NFAT2|NFATc	18	NaturalKiller_Cell_Cytotoxicity
NFATC2	4773	nuclear factor of activated T-cells, cytoplasmic, calcineurin-dependent 2	NFAT1|NFATP	20	NaturalKiller_Cell_Cytotoxicity
NFATC3	4775	nuclear factor of activated T-cells, cytoplasmic, calcineurin-dependent 3	NFAT4|NFATX	16	NaturalKiller_Cell_Cytotoxicity
NFATC4	4776	nuclear factor of activated T-cells, cytoplasmic, calcineurin-dependent 4	NF-ATc4|NFAT3	14	NaturalKiller_Cell_Cytotoxicity
PRKCA	5578	protein kinase C, alpha	AAG6|MGC129900|MGC129901|PKC-alpha|PKCA|PRKACA	17	NaturalKiller_Cell_Cytotoxicity
PRKCB	5579	protein kinase C, beta	MGC41878|PKC-beta|PKCB|PRKCB1|PRKCB2	16	NaturalKiller_Cell_Cytotoxicity
PRKCG	5582	protein kinase C, gamma	MGC57564|PKC-gamma|PKCC|PKCG|SCA14	19	NaturalKiller_Cell_Cytotoxicity
SH2D1B	117157	SH2 domain containing 1B	EAT2	1	NaturalKiller_Cell_Cytotoxicity
SH2D1A	4068	SH2 domain protein 1A	DSHP|EBVS|FLJ18687|FLJ92177|IMD5|LYP|MTCP1|SAP|XLP|XLPD	X	NaturalKiller_Cell_Cytotoxicity
IFNGR1	3459	interferon gamma receptor 1	CD119|FLJ45734|IFNGR	6	NaturalKiller_Cell_Cytotoxicity
IFNGR2	3460	interferon gamma receptor 2 (interferon gamma transducer 1)	AF-1|IFGR2|IFNGT1	21	NaturalKiller_Cell_Cytotoxicity
IFNA1	3439	interferon, alpha 1	IFL|IFN|IFN-ALPHA|IFNA13|IFNA@|MGC138207|MGC138505|MGC138507	9	NaturalKiller_Cell_Cytotoxicity
IFNA2	3440	interferon, alpha 2	IFNA|INFA2|MGC125764|MGC125765	9	NaturalKiller_Cell_Cytotoxicity

IFNA4	3441	interferon, alpha 4	INFA4|MGC142200	9	NaturalKiller_Cell_Cytotoxicity
IFNA5	3442	interferon, alpha 5	INFA5	9	NaturalKiller_Cell_Cytotoxicity
IFNA6	3443	interferon, alpha 6	-	9	NaturalKiller_Cell_Cytotoxicity
IFNA7	3444	interferon, alpha 7	IFNA-J	9	NaturalKiller_Cell_Cytotoxicity
IFNA8	3445	interferon, alpha 8	-	9	NaturalKiller_Cell_Cytotoxicity
IFNA10	3446	interferon, alpha 10	MGC119878|MGC119879	9	NaturalKiller_Cell_Cytotoxicity
IFNA13	3447	interferon, alpha 13	-	9	NaturalKiller_Cell_Cytotoxicity
IFNA14	3448	interferon, alpha 14	LEIF2H|MGC125756|MGC125757	9	NaturalKiller_Cell_Cytotoxicity
IFNA16	3449	interferon, alpha 16	-	9	NaturalKiller_Cell_Cytotoxicity
IFNA17	3451	interferon, alpha 17	IFNA|INFA|LEIF2C1	9	NaturalKiller_Cell_Cytotoxicity
IFNA21	3452	interferon, alpha 21	MGC126687|MGC126689	9	NaturalKiller_Cell_Cytotoxicity
IFNB1	3456	interferon, beta 1, fibroblast	IFB|IFF|IFNB|MGC96956	9	NaturalKiller_Cell_Cytotoxicity
IFNAR1	3454	interferon (alpha, beta and omega) receptor 1	AVP|IFN-alpha-REC|IFNAR|IFNBR|IFRC	21	NaturalKiller_Cell_Cytotoxicity
IFNAR2	3455	interferon (alpha, beta and omega) receptor 2	IFN-R|IFN-alpha-REC|IFNABR|IFNARB	21	NaturalKiller_Cell_Cytotoxicity
TNFSF10	8743	tumor necrosis factor (ligand) superfamily, member 10	APO2L|Apo-2L|CD253|TL2|TRAIL	3	NaturalKiller_Cell_Cytotoxicity
TNFRSF10D	8793	tumor necrosis factor receptor superfamily, member 10d, decoy with truncated death domain	CD264|DCR2|TRAILR4|TRUNDD	8	NaturalKiller_Cell_Cytotoxicity
TNFRSF10C	8794	tumor necrosis factor receptor superfamily, member 10c, decoy without an intracellular domain	CD263|DCR1|LIT|MGC149501|MGC149502|TRAILR3|TRID	8	NaturalKiller_Cell_Cytotoxicity
TNFRSF10B	8795	tumor necrosis factor receptor superfamily, member 10b	CD262|DR5|KILLER|KILLER/DR5|TRAIL-R2|TRAILR2|TRICK2|TRICK2A|TRICK2B|TRIC	8	NaturalKiller_Cell_Cytotoxicity

			KB|ZTNFR9		
TNFRSF10A	8797	tumor necrosis factor receptor superfamily, member 10a	APO2|CD261|DR4|MGC9365|TRAILR-1|TRAILR1	8	NaturalKiller_Cell_Cytotoxicity
FASLG	356	Fas ligand (TNF superfamily, member 6)	APT1LG1|CD178|CD95L|FASL|TNFSF6	1	NaturalKiller_Cell_Cytotoxicity
FAS	355	Fas (TNF receptor superfamily, member 6)	ALPS1A|APO-1|APT1|CD95|FAS1|FASTM|TNFRSF6	10	NaturalKiller_Cell_Cytotoxicity
GZMB	3002	granzyme B (granzyme 2, cytotoxic T-lymphocyte-associated serine esterase 1)	CCPI|CGL-1|CGL1|CSP-B|CSPB|CTLA1|CTSGL1|HLP|SECT	14	NaturalKiller_Cell_Cytotoxicity
PRF1	5551	perforin 1 (pore forming protein)	FLH2|HPLH2|MGC65093|P1|PFN1|PFP	10	NaturalKiller_Cell_Cytotoxicity
CASP3	836	caspase 3, apoptosis-related cysteine peptidase	CPP32|CPP32B|SCA-1	4	NaturalKiller_Cell_Cytotoxicity
BID	637	BH3 interacting domain death agonist	FP497|MGC15319|MGC42355	22	NaturalKiller_Cell_Cytotoxicity
CD3D	915	CD3d molecule, delta (CD3-TCR complex)	CD3-DELTA|T3D	11	TCR signaling Pathway
CD3E	916	CD3e molecule, epsilon (CD3-TCR complex)	FLJ18683|T3E|TCRE	11	TCR signaling Pathway
CD3G	917	CD3g molecule, gamma (CD3-TCR complex)	CD3-GAMMA|FLJ17620|FLJ17664|FLJ79544|FLJ94613|MGC138597|T3G	11	TCR signaling Pathway
CD247	919	CD247 molecule	CD3-ZETA|CD3H|CD3Q|CD3Z|T3Z|TCRZ	1	TCR signaling Pathway
CD4	920	CD4 molecule	CD4mut	12	TCR signaling Pathway
CD8A	925	CD8a molecule	CD8|Leu2|MAL|p32	2	TCR signaling Pathway
CD8B	926	CD8b molecule	CD8B1|LYT3|Leu2|Ly3|MGC119115	2	TCR signaling Pathway

PTPRC	5788	protein tyrosine phosphatase, receptor type, C	B220|CD45|CD45R|GP180|LCA|LY5|T200	1	TCR signaling Pathway
LCK	3932	lymphocyte-specific protein tyrosine kinase	YT16|p56lck|pp58lck	1	TCR signaling Pathway
FYN	2534	FYN oncogene related to SRC, FGR, YES	MGC45350|SLK|SYN	6	TCR signaling Pathway
ZAP70	7535	zeta-chain (TCR) associated protein kinase 70kDa	FLJ17670|FLJ17679|SRK|STD|TZK|ZAP-70	2	TCR signaling Pathway
LCP2	3937	lymphocyte cytosolic protein 2 (SH2 domain containing leukocyte protein of 76kDa)	SLP-76|SLP76	5	TCR signaling Pathway
LAT	27040	linker for activation of T cells	LAT1|pp36	16	TCR signaling Pathway
ITK	3702	IL2-inducible T-cell kinase	EMT|LYK|MGC126257|MGC126258|PSCTK2	5	TCR signaling Pathway
TEC	7006	tec protein tyrosine kinase	MGC126760|MGC126762|PSCTK4	4	TCR signaling Pathway
NCK1	4690	NCK adaptor protein 1	MGC12668|NCK|NCKalpha	3	TCR signaling Pathway
NCK2	8440	NCK adaptor protein 2	GRB4|NCKbeta	2	TCR signaling Pathway
VAV3	10451	vav 3 guanine nucleotide exchange factor	FLJ40431	1	TCR signaling Pathway
VAV1	7409	vav 1 guanine nucleotide exchange factor	VAV	19	TCR signaling Pathway
VAV2	7410	vav 2 guanine nucleotide exchange factor	-	9	TCR signaling Pathway
GRAP2	9402	GRB2-related adaptor protein 2	GADS|GRAP-2|GRB2L|GRBLG|GRID|GRPL|GrbX|Grf40|Mona|P38	22	TCR signaling Pathway

GRB2	2885	growth factor receptor-bound protein 2	ASH|EGFRBP-GRB2|Grb3-3|MST084|MSTP084	17	TCR signaling Pathway
PAK1	5058	p21 protein (Cdc42/Rac)-activated kinase 1	MGC130000|MGC130001|PAKalpha	11	TCR signaling Pathway
PAK2	5062	p21 protein (Cdc42/Rac)-activated kinase 2	PAK65|PAKgamma	3	TCR signaling Pathway
PAK3	5063	p21 protein (Cdc42/Rac)-activated kinase 3	CDKN1A|MRX30|MRX47|OPHN3|PAK3beta|bPAK|hPAK3	X	TCR signaling Pathway
PAK4	10298	p21 protein (Cdc42/Rac)-activated kinase 4	-	19	TCR signaling Pathway
PAK6	56924	p21 protein (Cdc42/Rac)-activated kinase 6	PAK5	15	TCR signaling Pathway
PAK7	57144	p21 protein (Cdc42/Rac)-activated kinase 7	KIAA1264|MGC26232|PAK5	20	TCR signaling Pathway
RHOA	387	ras homolog gene family, member A	ARH12|ARHA|RHO12|RHOH12	3	TCR signaling Pathway
CDC42	998	cell division cycle 42 (GTP binding protein, 25kDa)	CDC42Hs|G25K	1	TCR signaling Pathway
PPP3CA	5530	protein phosphatase 3 (formerly 2B), catalytic subunit, alpha isoform	CALN|CALNA|CALNA1|CCN1|CNA1|PPP2B	4	TCR signaling Pathway
PPP3CB	5532	protein phosphatase 3 (formerly 2B), catalytic subunit, beta isoform	CALNA2|CALNB	10	TCR signaling Pathway

PPP3CC	5533	protein phosphatase 3 (formerly 2B), catalytic subunit, gamma isoform	CALNA3	8	TCR signaling Pathway
CHP	11261	calcium binding protein P22	SLC9A1BP	15	TCR signaling Pathway
PPP3R1	5534	protein phosphatase 3 (formerly 2B), regulatory subunit B, alpha isoform	CALNB1|CNB|CNB1	2	TCR signaling Pathway
PPP3R2	5535	protein phosphatase 3 (formerly 2B), regulatory subunit B, beta isoform	PPP3RL	9	TCR signaling Pathway
CHP2	63928	calcineurin B homologous protein 2	-	16	TCR signaling Pathway
NFAT5	10725	nuclear factor of activated T-cells 5, tonicity-responsive	KIAA0827|NF-AT5|NFATL1|NFATZ|OREBP|TONEBP	16	TCR signaling Pathway
NFATC1	4772	nuclear factor of activated T-cells, cytoplasmic, calcineurin-dependent 1	MGC138448|NF-ATC|NFAT2|NFATc	18	TCR signaling Pathway
NFATC2	4773	nuclear factor of activated T-cells, cytoplasmic, calcineurin-dependent 2	NFAT1|NFATP	20	TCR signaling Pathway
NFATC3	4775	nuclear factor of activated T-cells, cytoplasmic, calcineurin-dependent 3	NFAT4|NFATX	16	TCR signaling Pathway
NFATC4	4776	nuclear factor of activated T-cells, cytoplasmic, calcineurin-dependent 4	NF-ATc4|NFAT3	14	TCR signaling Pathway

SOS1	6654	son of sevenless homolog 1 (Drosophila)	GF1|GGF1|GINGF|HGF|NS4	2	TCR signaling Pathway
SOS2	6655	son of sevenless homolog 2 (Drosophila)	FLJ25596	14	TCR signaling Pathway
HRAS	3265	v-Ha-ras Harvey rat sarcoma viral oncogene homolog	C-BAS/HAS|C-H-RAS|C-HA-RAS1|CTLO|H-RASIDX|HAMSV|HRAS1|K-RAS|N-RAS|RASH1	11	TCR signaling Pathway
KRAS	3845	v-Ki-ras2 Kirsten rat sarcoma viral oncogene homolog	C-K-RAS|K-RAS2A|K-RAS2B|K-RAS4A|K-RAS4B|KI-RAS|KRAS1|KRAS2|NS3|RASK2	12	TCR signaling Pathway
NRAS	4893	neuroblastoma RAS viral (v-ras) oncogene homolog	ALPS4|N-ras|NRAS1	1	TCR signaling Pathway
FOS	2353	v-fos FBJ murine osteosarcoma viral oncogene homolog	AP-1|C-FOS	14	TCR signaling Pathway
JUN	3725	jun oncogene	AP-1|AP1|c-Jun	1	TCR signaling Pathway
CARD11	84433	caspase recruitment domain family, member 11	BIMP3|CARMA1|MGC133069	7	TCR signaling Pathway
BCL10	8915	B-cell CLL/lymphoma 10	CARMEN|CIPER|CLAP|c-E10|mE10	1	TCR signaling Pathway
MALT1	10892	mucosa associated lymphoid tissue lymphoma translocation gene 1	DKFZp434L132|MLT|MLT1	18	TCR signaling Pathway
CHUK	1147	conserved helix-loop-helix ubiquitous kinase	IKBKA|IKK-alpha|IKK1|IKKA|NFKBIKA|TCF16	10	TCR signaling Pathway
IKBKB	3551	inhibitor of kappa light polypeptide gene enhancer in B-cells, kinase beta	FLJ40509|IKK-beta|IKK2|IKKB|MGC131801|NFKBIKB	8	TCR signaling Pathway

IKBKG	8517	inhibitor of kappa light polypeptide gene enhancer in B-cells, kinase gamma	AMCBX1|FIP-3|FIP3|Fip3p|IKK-gamma|IP|IP1|IP2|IPD2|NEMO	X	TCR signaling Pathway
NFKB1	4790	nuclear factor of kappa light polypeptide gene enhancer in B-cells 1	DKFZp686C01211|EBP-1|KBF1|MGC54151|NF-kappa-B|NFKB-p105|NFKB-p50|p105|p50	4	TCR signaling Pathway
RELA	5970	v-rel reticuloendotheliosis viral oncogene homolog A (avian)	MGC131774|NFKB3|p65	11	TCR signaling Pathway
NFKBIA	4792	nuclear factor of kappa light polypeptide gene enhancer in B-cells inhibitor, alpha	IKBA|MAD-3|NFKBI	14	TCR signaling Pathway
NFKBIB	4793	nuclear factor of kappa light polypeptide gene enhancer in B-cells inhibitor, beta	IKBB|TRIP9	19	TCR signaling Pathway
NFKBIE	4794	nuclear factor of kappa light polypeptide gene enhancer in B-cells inhibitor, epsilon	IKBE	6	TCR signaling Pathway
CD28	940	CD28 molecule	MGC138290|Tp44	2	TCR signaling Pathway
ICOS	29851	inducible T-cell co-stimulator	AILIM|CD278|MGC39850	2	TCR signaling Pathway
CD40LG	959	CD40 ligand	CD154|CD40L|HIGM1|IGM|IMD3|T-BAM|TNFSF5|TRAP|gp39|hCD40L	X	TCR signaling Pathway
PIK3R5	23533	phosphoinositide-3-kinase, regulatory subunit 5	F730038I15Rik|FOAP-2|P101-PI3K|p101	17	TCR signaling Pathway
PIK3R1	5295	phosphoinositide-3-kinase, regulatory subunit 1 (alpha)	GRB1|p85|p85-ALPHA	5	TCR signaling Pathway

PIK3R2	5296	phosphoinositide-3-kinase, regulatory subunit 2 (beta)	P85B|p85|p85-BETA	19	TCR signaling Pathway
PIK3R3	8503	phosphoinositide-3-kinase, regulatory subunit 3 (gamma)	DKFZp686P05226|FLJ41892|p55|p55-GAMMA	1	TCR signaling Pathway
PIK3CA	5290	phosphoinositide-3-kinase, catalytic, alpha polypeptide	MGC142161|MGC142163|PI3K|p110-alpha	3	TCR signaling Pathway
PIK3CB	5291	phosphoinositide-3-kinase, catalytic, beta polypeptide	DKFZp779K1237|MGC133043|PI3K|PI3KCB|PI3Kbeta|PIK3C1|p110-BETA	3	TCR signaling Pathway
PIK3CD	5293	phosphoinositide-3-kinase, catalytic, delta polypeptide	p110D	1	TCR signaling Pathway
PIK3CG	5294	phosphoinositide-3-kinase, catalytic, gamma polypeptide	PI3CG|PI3K|PI3Kgamma|PIK3	7	TCR signaling Pathway
AKT3	10000	v-akt murine thymoma viral oncogene homolog 3 (protein kinase B, gamma)	DKFZp434N0250|PKB-GAMMA|PKBG|PRKBG|RAC-PK-gamma|RAC-gamma|STK-2	1	TCR signaling Pathway
AKT1	207	v-akt murine thymoma viral oncogene homolog 1	AKT|MGC99656|PKB|PKB-ALPHA|PRKBA|RAC|RAC-ALPHA	14	TCR signaling Pathway
AKT2	208	v-akt murine thymoma viral oncogene homolog 2	PKBB|PKBBETA|PRKBB|RAC-BETA	19	TCR signaling Pathway
MAP3K8	1326	mitogen-activated protein kinase kinase kinase 8	COT|EST|ESTF|FLJ10486|TPL2|Tpl-2|c-COT	10	TCR signaling Pathway
MAP3K14	9020	mitogen-activated protein kinase kinase kinase 14	FTDCR1B|HS|HSNIK|NIK	17	TCR signaling Pathway
PDCD1	5133	programmed cell death 1	CD279|PD1|SLEB2|hPD-1|hPD-l	2	TCR signaling Pathway
CTLA4	1493	cytotoxic T-lymphocyte-associated protein 4	CD|CD152|CELIAC3|CTLA-4|GSE|ICOS|IDDM12	2	TCR signaling Pathway

PTPN6	5777	protein tyrosine phosphatase, non-receptor type 6	HCP|HCPH|HPTP1C|PTP-1C|SH-PTP1|SHP-1|SHP-1L|SHP1	12	TCR signaling Pathway
CBLC	23624	Cas-Br-M (murine) ecotropic retroviral transforming sequence c	CBL-3|CBL-SL|RNF57	19	TCR signaling Pathway
CBL	867	Cas-Br-M (murine) ecotropic retroviral transforming sequence	C-CBL|CBL2|RNF55	11	TCR signaling Pathway
CBLB	868	Cas-Br-M (murine) ecotropic retroviral transforming sequence b	DKFZp686J10223|DKFZp779A0729|DKFZp779F1443|FLJ36865|FLJ41152|Nbla00127|RNF56	3	TCR signaling Pathway
IL2	3558	interleukin 2	IL-2|TCGF|lymphokine	4	TCR signaling Pathway
IL4	3565	interleukin 4	BCGF-1|BCGF1|BSF1|IL-4|MGC79402	5	TCR signaling Pathway
IL5	3567	interleukin 5 (colony-stimulating factor, eosinophil)	EDF|IL-5|TRF	5	TCR signaling Pathway
IL10	3586	interleukin 10	CSIF|IL-10|IL10A|MGC126450|MGC126451|TGIF	1	TCR signaling Pathway
IFNG	3458	interferon, gamma	IFG|IFI	12	TCR signaling Pathway
CSF2	1437	colony stimulating factor 2 (granulocyte-macrophage)	GMCSF|MGC131935|MGC138897	5	TCR signaling Pathway
TNF	7124	tumor necrosis factor (TNF superfamily, member 2)	DIF|TNF-alpha|TNFA|TNFSF2	6	TCR signaling Pathway
CDK4	1019	cyclin-dependent kinase 4	CMM3|MGC14458|PSK-J3	12	TCR signaling Pathway
RASGRP1	10125	RAS guanyl releasing protein 1 (calcium and DAG-regulated)	CALDAG-GEFI|CALDAG-GEFII|MGC129998|MGC129999|RASGRP|V|hRasGRP1	15	TCR signaling Pathway

PDK1	5163	pyruvate dehydrogenase kinase, isozyme 1	-	2	TCR signaling Pathway
PLCG1	5335	phospholipase C, gamma 1	PLC-II|PLC1|PLC148|PLCgamma1	20	TCR signaling Pathway
PRKCQ	5588	protein kinase C, theta	MGC126514|MGC141919|PRKCT|nPKC-theta	10	TCR signaling Pathway
TRAC	28755	T cell receptor alpha constant	-	14	TCR signaling Pathway
TRAJ1	28754	T cell receptor alpha joining 1 (non-functional)	-	14	TCR signaling Pathway
TRAJ2	28753	T cell receptor alpha joining 2 (non-functional)	-	14	TCR signaling Pathway
TRAJ3	28752	T cell receptor alpha joining 3	-	14	TCR signaling Pathway
TRAJ4	28751	T cell receptor alpha joining 4	-	14	TCR signaling Pathway
TRAJ5	28750	T cell receptor alpha joining 5	-	14	TCR signaling Pathway
TRAJ6	28749	T cell receptor alpha joining 6	-	14	TCR signaling Pathway
TRAJ7	28748	T cell receptor alpha joining 7	-	14	TCR signaling Pathway
TRAJ8	28747	T cell receptor alpha joining 8	-	14	TCR signaling Pathway
TRAJ9	28746	T cell receptor alpha joining 9	-	14	TCR signaling Pathway
TRAJ10	28745	T cell receptor alpha joining 10	-	14	TCR signaling Pathway
TRAJ11	28744	T cell receptor alpha joining 11	-	14	TCR signaling Pathway
TRAJ12	28743	T cell receptor alpha joining 12	-	14	TCR signaling Pathway
TRAJ13	28742	T cell receptor alpha joining 13	-	14	TCR signaling Pathway
TRAJ14	28741	T cell receptor alpha joining 14	-	14	TCR signaling Pathway
TRAJ15	28740	T cell receptor alpha joining 15	-	14	TCR signaling Pathway
TRAJ16	28739	T cell receptor alpha joining 16	-	14	TCR signaling Pathway
TRAJ17	28738	T cell receptor alpha joining 17	-	14	TCR signaling Pathway
TRAJ18	28737	T cell receptor alpha joining 18	-	14	TCR signaling Pathway

TRAJ19	28736	T cell receptor alpha joining 19 (non-functional)	-	14	TCR signaling Pathway
TRAJ20	28735	T cell receptor alpha joining 20	-	14	TCR signaling Pathway
TRAJ21	28734	T cell receptor alpha joining 21	-	14	TCR signaling Pathway
TRAJ22	28733	T cell receptor alpha joining 22	-	14	TCR signaling Pathway
TRAJ23	28732	T cell receptor alpha joining 23	-	14	TCR signaling Pathway
TRAJ24	28731	T cell receptor alpha joining 24	-	14	TCR signaling Pathway
TRAJ25	28730	T cell receptor alpha joining 25 (non-functional)	-	14	TCR signaling Pathway
TRAJ26	28729	T cell receptor alpha joining 26	-	14	TCR signaling Pathway
TRAJ27	28728	T cell receptor alpha joining 27	-	14	TCR signaling Pathway
TRAJ28	28727	T cell receptor alpha joining 28	-	14	TCR signaling Pathway
TRAJ29	28726	T cell receptor alpha joining 29	-	14	TCR signaling Pathway
TRAJ30	28725	T cell receptor alpha joining 30	-	14	TCR signaling Pathway
TRAJ31	28724	T cell receptor alpha joining 31	-	14	TCR signaling Pathway
TRAJ32	28723	T cell receptor alpha joining 32	-	14	TCR signaling Pathway
TRAJ33	28722	T cell receptor alpha joining 33	-	14	TCR signaling Pathway
TRAJ34	28721	T cell receptor alpha joining 34	-	14	TCR signaling Pathway
TRAJ35	28720	T cell receptor alpha joining 35 (non-functional)	-	14	TCR signaling Pathway
TRAJ36	28719	T cell receptor alpha joining 36	-	14	TCR signaling Pathway
TRAJ37	28718	T cell receptor alpha joining 37	-	14	TCR signaling Pathway
TRAJ38	28717	T cell receptor alpha joining 38	-	14	TCR signaling Pathway
TRAJ39	28716	T cell receptor alpha joining 39	-	14	TCR signaling Pathway
TRAJ40	28715	T cell receptor alpha joining 40	-	14	TCR signaling Pathway
TRAJ41	28714	T cell receptor alpha joining 41	-	14	TCR signaling Pathway

TRAJ42	28713	T cell receptor alpha joining 42	-	14	TCR signaling Pathway
TRAJ43	28712	T cell receptor alpha joining 43	-	14	TCR signaling Pathway
TRAJ44	28711	T cell receptor alpha joining 44	-	14	TCR signaling Pathway
TRAJ45	28710	T cell receptor alpha joining 45	-	14	TCR signaling Pathway
TRAJ46	28709	T cell receptor alpha joining 46	-	14	TCR signaling Pathway
TRAJ47	28708	T cell receptor alpha joining 47	-	14	TCR signaling Pathway
TRAJ48	28707	T cell receptor alpha joining 48	-	14	TCR signaling Pathway
TRAJ49	28706	T cell receptor alpha joining 49	-	14	TCR signaling Pathway
TRAJ50	28705	T cell receptor alpha joining 50	-	14	TCR signaling Pathway
TRAJ52	28703	T cell receptor alpha joining 52	-	14	TCR signaling Pathway
TRAJ53	28702	T cell receptor alpha joining 53	-	14	TCR signaling Pathway
TRAJ54	28701	T cell receptor alpha joining 54	-	14	TCR signaling Pathway
TRAJ56	28699	T cell receptor alpha joining 56	-	14	TCR signaling Pathway
TRAJ57	28698	T cell receptor alpha joining 57	-	14	TCR signaling Pathway
TRAJ58	28697	T cell receptor alpha joining 58 (non-functional)	-	14	TCR signaling Pathway
TRAJ59	28696	T cell receptor alpha joining 59 (non-functional)	-	14	TCR signaling Pathway
TRAJ61	28694	T cell receptor alpha joining 61 (non-functional)	-	14	TCR signaling Pathway
TRAV1-1	28693	T cell receptor alpha variable 1-1	TCRAV1S1|TCRAV7S1|TRAV11	14	TCR signaling Pathway
TRAV1-2	28692	T cell receptor alpha variable 1-2	TCRAV1S2|TCRAV7S2|TRAV12	14	TCR signaling Pathway
TRAV2	28691	T cell receptor alpha variable 2	TCRAV11S1|TCRAV2S1	14	TCR signaling Pathway

TRAV3	28690	T cell receptor alpha variable 3 (gene/pseudogene)	TCRAV16S1|TCRAV3S1	14	TCR signaling Pathway
TRAV4	28689	T cell receptor alpha variable 4	TCRAV20S1|TCRAV4S1	14	TCR signaling Pathway
TRAV5	28688	T cell receptor alpha variable 5	TCRAV15S1|TCRAV5S1	14	TCR signaling Pathway
TRAV7	28686	T cell receptor alpha variable 7	TCRAV7S1	14	TCR signaling Pathway
TRAV8-1	28685	T cell receptor alpha variable 8-1	TCRAV1S1|TCRAV8S1|TRAV81	14	TCR signaling Pathway
TRAV8-2	28684	T cell receptor alpha variable 8-2	TCRAV1S5|TCRAV8S2|TRAV82	14	TCR signaling Pathway
TRAV8-3	28683	T cell receptor alpha variable 8-3	TCRAV1S4|TCRAV8S3|TRAV83	14	TCR signaling Pathway
TRAV8-4	28682	T cell receptor alpha variable 8-4	TCRAV1S2|TCRAV8S4|TRAV84	14	TCR signaling Pathway
TRAV8-6	28680	T cell receptor alpha variable 8-6	TCRAV1S3|TCRAV8S6|TRAV86	14	TCR signaling Pathway
TRAV8-7	28679	T cell receptor alpha variable 8-7 (non-functional)	TCRAV8S7|TRAV87	14	TCR signaling Pathway
TRAV9-1	28678	T cell receptor alpha variable 9-1	TCRAV9S1|TRAV91	14	TCR signaling Pathway
TRAV9-2	28677	T cell receptor alpha variable 9-2	TCRAV22S1|TCRAV9S2|TRAV92	14	TCR signaling Pathway
TRAV10	28676	T cell receptor alpha variable 10	TCRAV10S1|TCRAV24S1	14	TCR signaling Pathway
TRAV12-1	28674	T cell receptor alpha variable 12-1	TCRAV12S1|TCRAV2S3|TRAV121	14	TCR signaling Pathway
TRAV12-2	28673	T cell receptor alpha variable 12-2	TCRAV12S2|TCRAV2S1|TRAV122	14	TCR signaling Pathway

TRAV12-3	28672	T cell receptor alpha variable 12-3	TCRAV12S3|TCRAV2S2|TRAV123	14	TCR signaling Pathway
TRAV13-1	28671	T cell receptor alpha variable 13-1	TCRAV13S1|TCRAV8S1|TRAV131	14	TCR signaling Pathway
TRAV13-2	28670	T cell receptor alpha variable 13-2	TCRAV13S2|TCRAV8S2|TRAV132	14	TCR signaling Pathway
TRAV14DV4	28669	T cell receptor alpha variable 14/delta variable 4	TCRAV6S1-hDV104S1|TRAV14/DV4|hADV14S1	14	TCR signaling Pathway
TRAV16	28667	T cell receptor alpha variable 16	TCRAV16S1|TCRAV9S1	14	TCR signaling Pathway
TRAV17	28666	T cell receptor alpha variable 17	TCRAV17S1|TCRAV3S1	14	TCR signaling Pathway
TRAV18	28665	T cell receptor alpha variable 18	TCRAV18S1	14	TCR signaling Pathway
TRAV19	28664	T cell receptor alpha variable 19	TCRAV12S1|TCRAV19S1	14	TCR signaling Pathway
TRAV20	28663	T cell receptor alpha variable 20	TCRAV20S1|TCRAV30S1	14	TCR signaling Pathway
TRAV21	28662	T cell receptor alpha variable 21	TCRAV21S1|TCRAV23S1	14	TCR signaling Pathway
TRAV22	28661	T cell receptor alpha variable 22	TCRAV13S1|TCRAV22S1	14	TCR signaling Pathway
TRAV23DV6	28660	T cell receptor alpha variable 23/delta variable 6	TCRAV17S1|TRAV23/DV6|hADV23S1	14	TCR signaling Pathway
TRAV24	28659	T cell receptor alpha variable 24	TCRAV18S1|TCRAV24S1	14	TCR signaling Pathway
TRAV25	28658	T cell receptor alpha variable 25	TCRAV25S1|TCRAV32S1	14	TCR signaling Pathway
TRAV26-1	28657	T cell receptor alpha variable 26-1	TCRAV26S1|TCRAV4S2|TRAV261	14	TCR signaling Pathway
TRAV26-2	28656	T cell receptor alpha variable 26-2	TCRAV26S2|TCRAV4S1|TRAV262	14	TCR signaling Pathway
TRAV27	28655	T cell receptor alpha variable 27	TCRAV10S1|TCRAV27S1	14	TCR signaling Pathway

TRAV29DV5	28653	T cell receptor alpha variable 29/delta variable 5 (gene/pseudogene)	TCRAV21S1|TRAV29/DV5|hADV29S1	14	TCR signaling Pathway
TRAV30	28652	T cell receptor alpha variable 30	TCRAV29S1|TCRAV30S1	14	TCR signaling Pathway
TRAV34	28648	T cell receptor alpha variable 34	TCRAV26S1|TCRAV34S1	14	TCR signaling Pathway
TRAV35	28647	T cell receptor alpha variable 35	TCRAV25S1|TCRAV35S1	14	TCR signaling Pathway
TRAV36DV7	28646	T cell receptor alpha variable 36/delta variable 7	TCRAV28S1|TRAV36/DV7|hADV36S1	14	TCR signaling Pathway
TRAV38-1	28644	T cell receptor alpha variable 38-1	TCRAV14S2|TCRAV38S1|TRAV381	14	TCR signaling Pathway
TRAV38-2DV8	28643	T cell receptor alpha variable 38-2/delta variable 8	TCRAV14S1|TRAV382DV8|hADV38S2	14	TCR signaling Pathway
TRAV39	28642	T cell receptor alpha variable 39	TCRAV27S1|TCRAV39S1	14	TCR signaling Pathway
TRAV40	28641	T cell receptor alpha variable 40	TCRAV31S1|TCRAV40S1	14	TCR signaling Pathway
TRAV41	28640	T cell receptor alpha variable 41	TCRAV19S1|TCRAV41S1	14	TCR signaling Pathway
TRBC1	28639	T cell receptor beta constant 1	BV05S1J2.2|MGC88817|TCRBC1	7	TCR signaling Pathway
TRBC2	28638	T cell receptor beta constant 2	TCRBC2	7	TCR signaling Pathway
TRBD1	28637	T cell receptor beta diversity 1	TCRBD1	7	TCR signaling Pathway
TRBD2	28636	T cell receptor beta diversity 2	TCRBD2	7	TCR signaling Pathway
TRBJ1-1	28635	T cell receptor beta joining 1-1	TCRBJ1S1|TRBJ11	7	TCR signaling Pathway
TRBJ1-2	28634	T cell receptor beta joining 1-2	TCRBJ1S2|TRBJ12	7	TCR signaling Pathway
TRBJ1-3	28633	T cell receptor beta joining 1-3	TCRBJ1S3|TRBJ13	7	TCR signaling Pathway
TRBJ1-4	28632	T cell receptor beta joining 1-4	TCRBJ1S4|TRBJ14	7	TCR signaling Pathway
TRBJ1-5	28631	T cell receptor beta joining 1-5	TCRBJ1S5|TRBJ15	7	TCR signaling Pathway
TRBJ1-6	28630	T cell receptor beta joining 1-6	TCRBJ1S6|TRBJ16	7	TCR signaling Pathway
TRBJ2-1	28629	T cell receptor beta joining 2-1	TCRBJ2S1|TRBJ21	7	TCR signaling Pathway

TRBJ2-2	28628	T cell receptor beta joining 2-2	TCRBJ2S2|TRBJ22	7	TCR signaling Pathway
TRBJ2-3	28626	T cell receptor beta joining 2-3	TCRBJ2S3|TRBJ23	7	TCR signaling Pathway
TRBJ2-4	28625	T cell receptor beta joining 2-4	TCRBJ2S4|TRBJ24	7	TCR signaling Pathway
TRBJ2-5	28624	T cell receptor beta joining 2-5	TCRBJ2S5|TRBJ25	7	TCR signaling Pathway
TRBJ2-6	28623	T cell receptor beta joining 2-6	TCRBJ2S6|TRBJ26	7	TCR signaling Pathway
TRBJ2-7	28622	T cell receptor beta joining 2-7	TCRBJ2S7|TRBJ27	7	TCR signaling Pathway
TRBV2	28620	T cell receptor beta variable 2	TCRBV22S1A2N1T|TCRBV2S1	7	TCR signaling Pathway
TRBV3-1	28619	T cell receptor beta variable 3-1	TCRBV3S1|TCRBV9S1A1T|TRBV31	7	TCR signaling Pathway
TRBV4-1	28617	T cell receptor beta variable 4-1	BV07S1J2.7|TCRBV4S1|TCRBV7S1A1N2T|TRBV41	7	TCR signaling Pathway
TRBV4-2	28616	T cell receptor beta variable 4-2	TCRBV4S2|TCRBV7S3A2T|TRBV42	7	TCR signaling Pathway
TRBV4-3	28615	T cell receptor beta variable 4-3	TCRBV4S3|TCRBV7S2A1N4T|TRBV43	7	TCR signaling Pathway
TRBV5-1	28614	T cell receptor beta variable 5-1	TCRBV5S1|TCRBV5S1A1T|TRBV51	7	TCR signaling Pathway
TRBV5-4	28611	T cell receptor beta variable 5-4	TCRBV5S4|TCRBV5S6A3N2T|TRBV54	7	TCR signaling Pathway
TRBV5-5	28610	T cell receptor beta variable 5-5	TCRBV5S3A2T|TCRBV5S5|TRBV55	7	TCR signaling Pathway
TRBV5-6	28609	T cell receptor beta variable 5-6	TCRBV5S2|TCRBV5S6|TRBV56	7	TCR signaling Pathway
TRBV5-7	28608	T cell receptor beta variable 5-7 (non-functional)	TCRBV5S7|TCRBV5S7P|TRBV57	7	TCR signaling Pathway
TRBV5-8	28607	T cell receptor beta variable 5-8	TCRBV5S4A2T|TCRBV5S8|TRBV58	7	TCR signaling Pathway
TRBV6-1	28606	T cell receptor beta variable 6-1	TCRBV13S3|TCRBV6S1|TRBV61	7	TCR signaling Pathway
TRBV6-2	28605	T cell receptor beta variable 6-2 (gene/pseudogene)	TCRBV13S2A1T|TCRBV6S2|TRBV62	7	TCR signaling Pathway
TRBV6-3	28604	T cell receptor beta variable 6-3	TCRBV13S9/13S2A1T|TCRBV6S3|TRBV63	7	TCR signaling Pathway
TRBV6-4	28603	T cell receptor beta variable 6-4	TCRBV13S5|TCRBV6S4|TRBV64	7	TCR signaling Pathway
TRBV6-5	28602	T cell receptor beta variable 6-5	TCRBV13S1|TCRBV6S5|TRBV65	7	TCR signaling Pathway
TRBV6-6	28601	T cell receptor beta variable 6-6	TCRBV13S6A2T|TCRBV6S6|TRBV66	7	TCR signaling Pathway

TRBV6-7	28600	T cell receptor beta variable 6-7 (non-functional)	TCRBV13S8P|TCRBV6S7|TRBV67	7	TCR signaling Pathway
TRBV6-8	28599	T cell receptor beta variable 6-8	TCRBV13S7P|TCRBV6S8|TRBV68	7	TCR signaling Pathway
TRBV6-9	28598	T cell receptor beta variable 6-9	TCRBV13S4|TCRBV6S9|TRBV69	7	TCR signaling Pathway
TRBV7-2	28596	T cell receptor beta variable 7-2	MGC117435|TCRBV6S5A1N1|TCRBV7S2|TRBV72	7	TCR signaling Pathway
TRBV7-3	28595	T cell receptor beta variable 7-3	TCRBV6S1A1N1|TCRBV7S3|TRBV73	7	TCR signaling Pathway
TRBV7-4	28594	T cell receptor beta variable 7-4 (gene/pseudogene)	TCRBV6S8A2T|TCRBV7S4|TRBV74	7	TCR signaling Pathway
TRBV7-6	28592	T cell receptor beta variable 7-6	TCRBV6S3A1N1T|TCRBV7S6|TRBV76	7	TCR signaling Pathway
TRBV7-7	28591	T cell receptor beta variable 7-7	TCRBV6S6A2T|TCRBV7S7|TRBV77	7	TCR signaling Pathway
TRBV7-8	28590	T cell receptor beta variable 7-8	TCRBV6S2A1N1T|TCRBV7S8|TRBV78	7	TCR signaling Pathway
TRBV7-9	28589	T cell receptor beta variable 7-9	TCRBV6S4A1|TCRBV7S9|TRBV79	7	TCR signaling Pathway
TRBV9	28586	T cell receptor beta variable 9	TCRBV1S1A1N1|TCRBV9S1	7	TCR signaling Pathway
TRBV10-1	28585	T cell receptor beta variable 10-1(gene/pseudogene)	TCRBV10S1|TCRBV12S2|TCRBV12S2A1T|TRBV101	7	TCR signaling Pathway
TRBV10-2	28584	T cell receptor beta variable 10-2	TCRBV10S2|TCRBV12S3|TRBV102	7	TCR signaling Pathway
TRBV10-3	28583	T cell receptor beta variable 10-3	TCRBV10S3|TCRBV12S1A1N2|TRBV103	7	TCR signaling Pathway
TRBV11-1	28582	T cell receptor beta variable 11-1	TCRBV11S1|TCRBV21S1|TRBV111	7	TCR signaling Pathway
TRBV11-2	28581	T cell receptor beta variable 11-2	TCRBV11S2|TCRBV21S3A2N2T|TRBV112	7	TCR signaling Pathway
TRBV11-3	28580	T cell receptor beta variable 11-3	TCRBV11S3|TCRBV21S2A2|TRBV113	7	TCR signaling Pathway

TRBV12-3	28577	T cell receptor beta variable 12-3	TCRBV12S3|TCRBV8S1|TRBV123	7	TCR signaling Pathway
TRBV12-4	28576	T cell receptor beta variable 12-4	TCRBV12S4|TCRBV8S2A1T|TRBV124	7	TCR signaling Pathway
TRBV12-5	28575	T cell receptor beta variable 12-5	TCRBV12S5|TCRBV8S3|TRBV125	7	TCR signaling Pathway
TRBV13	28574	T cell receptor beta variable 13	TCRBV13S1|TCRBV23S1A2T	7	TCR signaling Pathway
TRBV14	28573	T cell receptor beta variable 14	TCRBV14S1|TCRBV16S1A1N1	7	TCR signaling Pathway
TRBV15	28572	T cell receptor beta variable 15	TCRBV15S1|TCRBV24S1A3T	7	TCR signaling Pathway
TRBV16	28571	T cell receptor beta variable 16 (gene/pseudogene)	TCRBV16S1|TCRBV25S1A2PT	7	TCR signaling Pathway
TRBV17	28570	T cell receptor beta variable 17 (non-functional)	TCRBV17S1|TCRBV26S1P	7	TCR signaling Pathway
TRBV18	28569	T cell receptor beta variable 18	TCRBV18S1	7	TCR signaling Pathway
TRBV19	28568	T cell receptor beta variable 19	TCRBV17S1A1T|TCRBV19S1	7	TCR signaling Pathway
TRBV20-1	28567	T cell receptor beta variable 20-1	TCRBV20S1|TCRBV2S1|TRBV201	7	TCR signaling Pathway
TRBV24-1	28563	T cell receptor beta variable 24-1	TCRBV15S1|TCRBV24S1|TRBV241	7	TCR signaling Pathway
TRBV25-1	28562	T cell receptor beta variable 25-1	TCRBV11S1A1T|TCRBV25S1|TRBV251	7	TCR signaling Pathway
TRBV27	28560	T cell receptor beta variable 27	FLJ35984|TCRBV14S1|TCRBV27S1	7	TCR signaling Pathway
TRBV28	28559	T cell receptor beta variable 28	TCRBV28S1|TCRBV3S1	7	TCR signaling Pathway
TRBV29-1	28558	T cell receptor beta variable 29-1	TCRBV29S1|TCRBV4S1A1T|TRBV291	7	TCR signalingPathway

TRBV30	28557	T cell receptor beta variable 30 (gene/pseudogene)	TCRBV20S1A1N2|TCRBV30S1	7	TCR signaling Pathway
TRDC	28526	T cell receptor delta constant	-	14	TCR signaling Pathway
TRDD1	28525	T cell receptor delta diversity 1	-	14	TCR signaling Pathway
TRDD2	28524	T cell receptor delta diversity 2	-	14	TCR signaling Pathway
TRDD3	28523	T cell receptor delta diversity 3	TCRD	14	TCR signaling Pathway
TRDJ1	28522	T cell receptor delta joining 1	TCRD	14	TCR signalingPathway
TRDJ2	28521	T cell receptor delta joining 2	-	14	TCR signaling Pathway
TRDJ3	28520	T cell receptor delta joining 3	-	14	TCR signaling Pathway
TRDJ4	28519	T cell receptor delta joining 4	-	14	TCR signaling Pathway
TRDV1	28518	T cell receptor delta variable 1	hDV101S1	14	TCR signaling Pathway
TRDV2	28517	T cell receptor delta variable 2	MGC117421|hDV102S1	14	TCR signaling Pathway
TRDV3	28516	T cell receptor delta variable 3	hDV103S1	14	TCR signaling Pathway
TRGV9	6983	T cell receptor gamma variable 9	MGC47828|TCRGV9|V2	7	TCR signaling Pathway
TRGV8	6982	T cell receptor gamma variable 8	TCRGV8|V1S8	7	TCR signaling Pathway
TRGV5	6978	T cell receptor gamma variable 5	TCRGV5|V1S5	7	TCR signaling Pathway
TRGV4	6977	T cell receptor gamma variable 4	TCRGV4|V1S4	7	TCR signaling Pathway
TRGV3	6976	T cell receptor gamma variable 3	TCRGV3|V1S3	7	TCR signaling Pathway
TRGV2	6974	T cell receptor gamma variable 2	MGC42817|TCRGV2|VIS2	7	TCR signaling Pathway

TRGJP2	6972	T cell receptor gamma joining P2	JP2|TCRGJP2	7	TCR signaling Pathway
TRGJP1	6971	T cell receptor gamma joining P1	JP1|TCRGJP1	7	TCR signaling Pathway
TRGJP	6970	T cell receptor gamma joining P	JP|TCRGJP	7	TCR signaling Pathway
TRGJ2	6969	T cell receptor gamma joining 2	J2|TCRGJ2	7	TCR signaling Pathway
TRGJ1	6968	T cell receptor gamma joining 1	J1|TCRGJ1	7	TCR signaling Pathway
TRGC2	6967	T cell receptor gamma constant 2	TCRGC2|TRGC2(2X)|TRGC2(3X)	7	TCR signaling Pathway
TRGC1	6966	T cell receptor gamma constant 1	C1|TCRGC1	7	TCR signaling Pathway
TRAV6	6956	T cell receptor alpha variable 6	TCRAV5S1|TCRAV6S1	14	TCR signalingPathway
BMP1	649	bone morphogenetic protein 1	FLJ44432|PCOLC|PCP|TLD|pCP-2	8	TGFb_Family_Member
BMP10	27302	bone morphogenetic protein 10	MGC126783	2	TGFb_Family_Member
BMP15	9210	bone morphogenetic protein 15	GDF9B|ODG2|POF4	X	TGFb_Family_Member
BMP2	650	bone morphogenetic protein 2	BMP2A	20	TGFb_Family_Member
BMP3	651	bone morphogenetic protein 3	BMP-3A	4	TGFb_Family_Member
BMP4	652	bone morphogenetic protein 4	BMP2B|BMP2B1|MCOPS6|OFC11|ZYME	14	TGFb_Family_Member
BMP5	653	bone morphogenetic protein 5	MGC34244	6	TGFb_Family_Member
BMP6	654	bone morphogenetic protein 6	VGR|VGR1	6	TGFb_Family_Member
BMP7	655	bone morphogenetic protein 7	OP-1	20	TGFb_Family_Member
BMP8A	353500	bone morphogenetic protein 8a	FLJ14351|FLJ45264	1	TGFb_Family_Member
BMP8B	656	bone morphogenetic protein 8b	BMP8|MGC131757|OP2	1	TGFb_Family_Member
GDF1	2657	growth differentiation factor 1	-	19	TGFb_Family_Member
GDF10	2662	growth differentiation factor 10	BMP-3b|BMP3B	10	TGFb_Family_Member
GDF11	10220	growth differentiation factor 11	BMP-11|BMP11	12	TGFb_Family_Member

GDF15	9518	growth differentiation factor 15	GDF-15|MIC-1|MIC1|NAG-1|PDF|PLAB|PTGFB	19	TGFb_Family_Member
GDF2	2658	growth differentiation factor 2	BMP-9|BMP9	10	TGFb_Family_Member
GDF3	9573	growth differentiation factor 3	-	12	TGFb_Family_Member
GDF5	8200	growth differentiation factor 5	BMP14|CDMP1|LAP4|OS5|SYNS2	20	TGFb_Family_Member
GDF6	392255	growth differentiation factor 6	BMP13|CDMP2|KFS|KFSL|MGC158100|MGC158101|SGM1	8	TGFb_Family_Member
GDF7	151449	growth differentiation factor 7	BMP12	2	TGFb_Family_Member
GDF9	2661	growth differentiation factor 9	-	5	TGFb_Family_Member
GDNF	2668	glial cell derived neurotrophic factor	ATF1|ATF2|HFB1-GDNF	5	TGFb_Family_Member
INHA	3623	inhibin, alpha	-	2	TGFb_Family_Member
INHBA	3624	inhibin, beta A	EDF|FRP	7	TGFb_Family_Member
INHBB	3625	inhibin, beta B	MGC157939	2	TGFb_Family_Member
INHBC	3626	inhibin, beta C	IHBC	12	TGFb_Family_Member
INHBE	83729	inhibin, beta E	MGC4638	12	TGFb_Family_Member
LEFTY1	10637	left-right determination factor 1	LEFTB|LEFTYB	1	TGFb_Family_Member
LEFTY2	7044	left-right determination factor 2	EBAF|LEFTA|LEFTYA|MGC46222|TGFB4	1	TGFb_Family_Member
NODAL	4838	nodal homolog (mouse)	MGC138230	10	TGFb_Family_Member
TGFB1	7040	transforming growth factor, beta 1	CED|DPD1|TGFB|TGFbeta	19	TGFb_Family_Member
TGFB2	7042	transforming growth factor, beta 2	MGC116892|TGF-beta2	1	TGFb_Family_Member
TGFB3	7043	transforming growth factor, beta 3	ARVD|FLJ16571|TGF-beta3	14	TGFb_Family_Member
ACVR1B	91	activin A receptor, type IB	ACTRIB|ACVRLK4|ALK4|SKR2	12	TGFb_Family_Member_ Receptor
ACVR1C	130399	activin A receptor, type IC	ACVRLK7|ALK7	2	TGFb_Family_Member_ Receptor

ACVR2A	92	activin A receptor, type IIA	ACTRII|ACVR2	2	TGFb_Family_Member_ Receptor
ACVR2B	93	activin A receptor, type IIB	ACTRIIB|ActR-IIB|MGC116908	3	TGFb_Family_Member_ Receptor
ACVRL1	94	activin A receptor type II-like 1	ACVRLK1|ALK-1|ALK1|HHT|HHT2|ORW2|SKR3|TSR-I	12	TGFb_Family_Member_ Receptor
AMHR2	269	anti-Mullerian hormone receptor, type II	AMHR|MISR2|MISRII	12	TGFb_Family_Member_ Receptor
BMPR1A	657	bone morphogenetic protein receptor, type IA	10q23del|ACVRLK3|ALK3|CD292|SKR5	10	TGFb_Family_Member_ Receptor
BMPR1B	658	bone morphogenetic protein receptor, type IB	ALK-6|ALK6|CDw293	4	TGFb_Family_Member_ Receptor
BMPR2	659	bone morphogenetic protein receptor, type II (serine/threonine kinase)	BMPR-II|BMPR3|BMR2|BRK-3|FLJ41585|FLJ76945|PPH1|T-ALK	2	TGFb_Family_Member_ Receptor
TGFBR1	7046	transforming growth factor, beta receptor 1	AAT5|ACVRLK4|ALK-5|ALK5|LDS1A|LDS2A|SKR4|TGFR-1	9	TGFb_Family_Member_ Receptor
TGFBR2	7048	transforming growth factor, beta receptor II (70/80kDa)	AAT3|FAA3|LDS1B|LDS2B|MFS2|RIIC|TAAD2|TGFR-2|TGFbeta-RII	3	TGFb_Family_Member_ Receptor
TGFBR3	7049	transforming growth factor, beta receptor III	BGCAN|betaglycan	1	TGFb_Family_Member_ Receptor
TNFRSF11B	4982	tumor necrosis factor receptor superfamily, member 11b	MGC29565|OCIF|OPG|TR1	8	TNF_Family_Members
TNFSF10	8743	tumor necrosis factor (ligand) superfamily, member 10	APO2L|Apo-2L|CD253|TL2|TRAIL	3	TNF_Family_Members
TNFSF11	8600	tumor necrosis factor (ligand) superfamily, member 11	CD254|ODF|OPGL|OPTB2|RANKL|TRANCE|hRANKL2|sOdf	13	TNF_Family_Members

TNFSF12	8742	tumor necrosis factor (ligand) superfamily, member 12	APO3L|DR3LG|MGC129581|MGC20669|TWEAK	17	TNF_Family_Members
TNFSF13	8741	tumor necrosis factor (ligand) superfamily, member 13	APRIL|CD256|TALL2|TRDL-1|UNQ383/PRO715|ligand	17	TNF_Family_Members
TNFSF13B	10673	tumor necrosis factor (ligand) superfamily, member 13b	BAFF|BLYS|CD257|DTL|TALL-1|TALL1|THANK|TNFSF20|ZTNF4	13	TNF_Family_Members
TNFSF14	8740	tumor necrosis factor (ligand) superfamily, member 14	CD258|HVEML|LIGHT|LTg|TR2	19	TNF_Family_Members
TNFSF15	9966	tumor necrosis factor (ligand) superfamily, member 15	MGC129934|MGC129935|TL1|TL1A|VEGI|VEGI192A	9	TNF_Family_Members
TNFSF18	8995	tumor necrosis factor (ligand) superfamily, member 18	AITRL|GITRL|MGC138237|TL6|hGITRL	1	TNF_Family_Members
TNFSF4	7292	tumor necrosis factor (ligand) superfamily, member 4	CD134L|CD252|GP34|OX-40L|OX4OL|TXGP1	1	TNF_Family_Members
TNFSF8	944	tumor necrosis factor (ligand) superfamily, member 8	CD153|CD30L|CD30LG|MGC138144	9	TNF_Family_Members
TNFSF9	8744	tumor necrosis factor (ligand) superfamily, member 9	4-1BB-L|CD137L	19	TNF_Family_Members
TNFRSF10B	8795	tumor necrosis factor receptor superfamily, member 10b	CD262|DR5|KILLER|KILLER/DR5|TRAIL-R2|TRAILR2|TRICK2|TRICK2A|TRICK2B|TRICKB|ZTNFR9	8	TNF_Family_Members_ Receptors
TNFRSF10C	8794	tumor necrosis factor receptor superfamily, member 10c, decoy without an intracellular domain	CD263|DCR1|LIT|MGC149501|MGC149502|TRAILR3|TRID	8	TNF_Family_Members_ Receptors

TNFRSF10D	8793	tumor necrosis factor receptor superfamily, member 10d, decoy with truncated death domain	CD264|DCR2|TRAILR4|TRUNDD	8	TNF_Family_Members_ Receptors
TNFRSF11A	8792	tumor necrosis factor receptor superfamily, member 11a, NFKB activator	CD265|FEO|LOH18CR1|ODFR|OFE|OPTB7|OSTS|PDB2|RANK|TRANCER	18	TNF_Family_Members_ Receptors
TNFRSF12A	51330	tumor necrosis factor receptor superfamily, member 12A	CD266|FN14|TWEAKR	16	TNF_Family_Members_ Receptors
TNFRSF13B	23495	tumor necrosis factor receptor superfamily, member 13B	CD267|CVID|FLJ39942|MGC133214|MGC39952|TACI|TNFRSF14B	17	TNF_Family_Members_ Receptors
TNFRSF13C	115650	tumor necrosis factor receptor superfamily, member 13C	BAFF-R|BAFFR|CD268|MGC138235	22	TNF_Family_Members_ Receptors
TNFRSF14	8764	tumor necrosis factor receptor superfamily, member 14 (herpesvirus entry mediator)	ATAR|HVEA|HVEM|LIGHTR|TR2	1	TNF_Family_Members_ Receptors
TNFRSF17	608	tumor necrosis factor receptor superfamily, member 17	BCM|BCMA|CD269	16	TNF_Family_Members_ Receptors
TNFRSF18	8784	tumor necrosis factor receptor superfamily, member 18	AITR|GITR|GITR-D	1	TNF_Family_Members_ Receptors
TNFRSF19	55504	tumor necrosis factor receptor superfamily, member 19	TAJ|TAJ-alpha|TRADE|TROY	13	TNF_Family_Members_ Receptors
TNFRSF1A	7132	tumor necrosis factor receptor superfamily, member 1A	CD120a|FPF|MGC19588|TBP1|TNF-R|TNF-R-I|TNF-R55|TNFAR|TNFR1|TNFR55|TNFR60|p55|p55-R|p60	12	TNF_Family_Members_ Receptors

TNFRSF1B	7133	tumor necrosis factor receptor superfamily, member 1B	CD120b|TBPII|TNF-R-II|TNF-R75|TNFBR|TNFR1B|TNFR2|TNFR80|p75|p75TNFR	1	TNF_Family_Members_ Receptors
TNFRSF21	27242	tumor necrosis factor receptor superfamily, member 21	BM-018|DR6|MGC31965	6	TNF_Family_Members_ Receptors
TNFRSF25	8718	tumor necrosis factor receptor superfamily, member 25	APO-3|DDR3|DR3|LARD|TNFRSF12|TR3|TRAMP|WSL-1|WSL-LR	1	TNF_Family_Members_ Receptors
TNFRSF4	7293	tumor necrosis factor receptor superfamily, member 4	ACT35|CD134|OX40|TXGP1L	1	TNF_Family_Members_ Receptors
TNFRSF6B	8771	tumor necrosis factor receptor superfamily, member 6b, decoy	DCR3|DJ583P15.1.1|M68|TR6	20	TNF_Family_Members_ Receptors
TNFRSF8	943	tumor necrosis factor receptor superfamily, member 8	CD30|D1S166E|Ki-1	1	TNF_Family_Members_ Receptors
TNFRSF9	3604	tumor necrosis factor receptor superfamily, member 9	4-1BB|CD137|CDw137|ILA|MGC2172	1	TNF_Family_Members_ Receptors
